# Si─Si Bonding in an Unsupported N‐Heterocyclic Silylene Dimer Stabilized by an Iminophosphorane‐Based Scorpionate Ligand Versus Head‐to‐Tail Coordination in the Sn and Pb Tetrylenes

**DOI:** 10.1002/chem.70980

**Published:** 2026-04-17

**Authors:** Huanhuan Dong, Rochelle Ferns, Luke W. Giles, Lea Fohlmeister, Connor Bourne, Samuel R. Lawrence, Aidan P. McKay, Alexandra M. Z. Slawin, David B. Cordes, Tanja van Mourik, Andreas Stasch

**Affiliations:** ^1^ EaStCHEM School of Chemistry University of St Andrews St Andrews UK; ^2^ School of Chemistry Monash University Melbourne Victoria Australia

**Keywords:** alkene analogs, group 14 elements, iminophosphoranes, multiple bonds, N‐heterocylic silylenes

## Abstract

N,N‐chelating ligands disfavor element–element bonding in heavier alkene analogs of group 13 and group 14 elements. Here, we present a series of group 14 element(II) compounds of a new sterically demanding iminophosphorane‐based scorpionate ligand. The molecular structures of the Si and Ge analogs form element–element bonded interactions in the solid state in a head‐to‐head fashion, whereas the Sn and Pb analogs form one‐dimensional coordination polymers in a head‐to‐tail fashion. Analysis of the Si─Si bonding by experimental, for example, X‐ray diffraction, and computational methods suggests that a significant unsupported bonding interaction is present and enabled by the ligand that can be easily perturbed by the ligand sterics, including dispersion, and broken by entropic effects.

## Graphical abstract

A new iminophosphorane‐based scorpionate ligand enabled the characterization of a head‐to‐head silylene dimer with unsupported Si─Si bonding, whereas the tetrylene species of Sn and Pb afforded head‐to‐tail coordination polymers in the solid state.

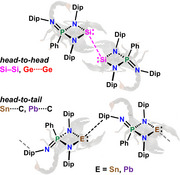

## 1 Introduction

**FIGURE 1:**
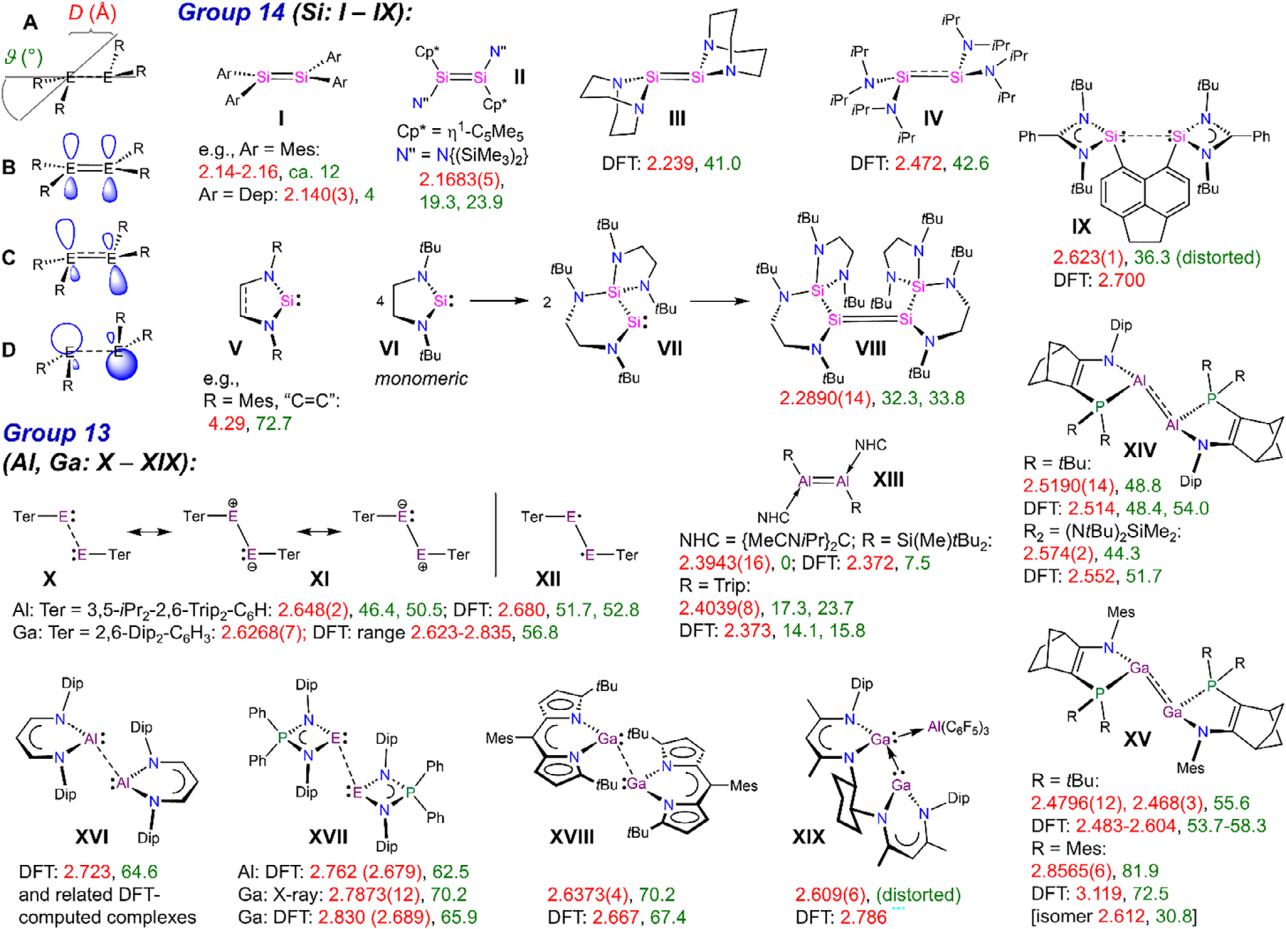
Sketch of bonding and *trans*‐bending in heavier alkene analogs and compound examples of group 14 (Si^II^) and group 13 (Al^I^, Ga^I^) elements with E–E distance (*D*, red) and *trans*‐bending angle (*ϑ*, green) from X‐ray diffraction and DFT computational studies. Note that the drawing interpretation of E–E bonding interactions is somewhat arbitrary. Mes = mesityl, Dep = 2,6‐diethylphenyl, Dip = 2,6‐diisopropylphenyl.

The bonding around the period 2 element carbon in planar alkenes is part of every chemist's knowledge, whereas our understanding of the complexities around bonding in heavier *p*‐block element alkene analogs is still developing [1, 2, 3, 4, 5]. These main group element–element bonding interactions are less understood, easier to perturb, and strongly influenced by the ligand entities bonded to the heavier main group centers, see Figure 1 for simplified bonding modes and relevant examples [1, 2, 3, 4, 5]. Of particular interest are alkene analogs with central aluminum and silicon centers due to their high elemental abundance on Earth, the new insights into element–element bonding they reveal, and the high reactivity of the low oxidation state species of these elements [3, 5, 6, 7]. The geometries in E═E (E = group 13 or 14 element) “double‐bonded” heavier alkene analogs show varying degrees of *trans*‐bending (Figure 1, **A–D**, selected group 14 (Si) examples **I**–**IX** and group 13 (Al, Ga) examples **X**‐**XIX**) depending on the nature of both E and R. This is seen to strongly affect the E─E bonding as judged by the wide range of E─E bond distances and *trans*‐bending angles [1, 2, 3, 4]. Disilenes with aryl substituents, Ar_2_Si═SiAr_2_ **I** [8, 9, 10, 11], feature a short and relatively strong Si═Si double bond, and their geometries generally range from planar to only slightly *trans*‐bent (*trans*‐bending angle *ϑ ca*. 0°–18°). The computed bond dissociation energies of disilenes can vary widely depending on the substituents [12]. Another class of silicon(II) compounds are N‐heterocyclic silylenes (NHSi), for example, **V** [13, 14, 15, 16, 17, 18]. These are, however, monomeric due to the two flanking N(R) groups that provide additional saturation and stabilization at the Si center from the N(R) lone pairs and from the electronegativity of the N atoms. This prompted the question of whether NHSi compounds can dimerize and a quest for an isolable NHSi dimer. It has been found that for electronic reasons such as lone pair repulsion at the N‐Si fragments, diaminosilylenes do not dimerize via short and strong Si═Si double bonds [19, 20, 21, 22, 23, 24, 25]. In contrast, hybrid organo‐amido silylenes such as Cp*SiN(SiMe_3_)_2_ or MesSiN(SiMe_3_)_2_, however, readily form Si═Si‐bonded dimers (disilene **II**) [26, 27], as do phosphino‐substituted silylenes [28], whereas diaminosilylenes were found to prefer dimerization via the N‐atoms according to DFT studies. Some Si─Si bonded dimers could be optimized in computational studies, however (e.g., **III** and **IV**) [19, 20, 21, 22, 23, 24, 25], and the long Si─Si bond predicted for **IV** was concluded to only show a weak (3–8 kcal mol^−1^) bonding interaction [23]. Another mechanism was elucidated where an N‐heterocyclic silylene (**VI**) dimerized to a ring‐expanded silylene via Si─N bond activation (**VII**), which is followed by dimerization to the tetrameric disilene **VIII** with a *trans*‐bent Si═Si bond [22]. Placing two three‐coordinate Si^II^ centers in close proximity led to an “enforced” bonding interaction in the case of compound **IX** that was found to react as a Si_2_ unit [29].

In group 13, terphenyl‐stabilized *trans*‐bent REER molecules (E = Al, Ga, **X**–**XII**) typically show relatively long and weak Al─Al and Ga─Ga bonds, respectively, with *ϑ* angles around 60°. These are found to dissociate in solution, however, and some reactivity has been reported to be favored to proceed via the dimeric forms over the lower‐coordinate monomeric form [30, 31, 32]. Additionally, some reactivity may proceed via appreciable contributions from (singlet) diradicaloid character (e.g., **XII**) in these compounds that is energetically accessible [33]. The NHC‐dialumene adducts **XIII** show short Al═Al double bonds with little *trans*‐bending [34, 35], whereas the N,P‐chelated dialumenes (**XIV**) [36, 37] and digallenes (**XV**) [38] show longer bonds and larger *trans*‐bending angles. The coordination ability of these N,P‐ligands shows some flexibility. The Al/Ga–P distances in some examples can vary significantly and, thus, these compounds could also be regarded as R_2_P donor‐stabilized amido complexes. For N,N‐chelating ligands, the DFT‐calculated β‐diketiminate aluminum(I) dimer (**XVI**) shows a significantly longer and more *trans*‐bent Al···Al interaction with some diradicaloid character from a low‐lying triplet state [39]. Very recently, a DFT study investigated the influence of various effects of N,N‐chelating and N,P‐chelating ligand classes on the Al–Al interaction in dimeric aluminum(I) compounds related to **XVI** [40]. For a diiminophosphinate‐supported gallium(I) dimer (**XVII**), both a weak Ga···Ga‐bonded isomer and a monomeric form have been structurally characterized. DFT studies on **XVII** for E ═ Al, Ga did reveal significant E─E bonding interactions and bond orders near one in the dimers [41]. A shorter Ga─Ga interaction than in **XVII** was found in a recently characterized dipyrromethene gallium(I) dimer **XVIII**, which shows bonding in the solid‐state and again a significant calculated bond index, but the Ga─Ga bonding is not retained in solution [42]. A bis(gallanediyl) coordinated by a bifunctional β‐diketiminate ligand forms a donor–acceptor Ga─Ga interaction only when coordinated to a strong Lewis acid (**XIX**), whereas the uncoordinated digallium(I) species shows an unbonded structure which, according to DFT calculations, is of similar energy compared to the bonded form (+1.7 kcal mol^−1^) [43, 44].

The relationship between the degree of *trans*‐bending in ditetrelenes (R_2_E═ER_2_, E = group 14 elements, **A**‐**D**), or if monomeric tetrylenes (tetrelenes, ER_2_) are afforded instead, has been described by the Carter–Goddard–Malrieu–Trinquier (CGMT) model [2, 45, 46, 47]. This model links distortions such as *trans*‐bending in the ditetrelenes or dissociation to monomeric tetrylenes to the singlet‐triplet energy separation (*ΔE*_s‐t_) of the ER_2_ fragments and the E═E bond strengths. For context in silicon chemistry, ab initio calculations on slightly *trans*‐bent H_2_Si═SiH_2_ suggest that *ca*. 87 kcal mol^−1^ are required to dissociate H_2_Si═SiH_2_ into two triplet state SiH_2_ fragments, whereas less energy (*ca*. 57 kcal mol^−1^) is required for dissociation into two singlet state SiH_2_ fragments, with a moderate *ΔE*_s‐t_ of *ca*. 20 kcal mol^−1^ [48]. All these types of energies are naturally heavily dependent on the properties of the ligands, R, and the nature of the central atom, E. N‐substituents strongly favor a singlet‐state silylene unit and disfavor Si═Si bonded species, and especially NHSis form generally stable monomeric species (**V**). The E─E bonding interactions in heavier main group alkene analogs become weaker down the group, and stabilizing effects from dispersion can often become the dominant effect in dimerizations, even when suitable orbital bonding interactions between the main group elements are present [3, 49, 50]. The influence of the ligand sterics, including dispersion effects, is especially important as sterically demanding ligands with often large hydrocarbyl groups are required to afford low‐coordinate and low oxidation state compounds of heavier *p*‐block elements. The degree of *trans*‐bending in heavier main group element alkene analogs (**A**–**D**) also influences the possible π‐bonding overlap between the main group elements. Increasing levels of *trans*‐bending lead to the former π‐bond from *p*‐orbital overlap (**B**) to adopt more lone pair (non‐bonding, n_‐_) character with higher *s*‐orbital content (**C**, to **D**) and increasing σ*‐like properties that are affected by orbital mixing with orbitals of the same symmetry (second‐order Jahn–Teller mixing) [3, 51]. These effects weaken the E─E bonding interaction and can increase the proportion of influence of the ligand sphere on geometry and bonding.

This series of examples (Figure 1) highlights the influence of two nitrogen substituents per element center on the nature of the E–E bonding interactions in heavier alkene analogs. It is evident that the π‐donating character and the electronegativity of the N‐substituents, especially if the central element is bonded to two N(R) groups, are weakening E─E bonding, leading to more *trans*‐bending with long E─E bonds, and most likely the formation of singlet‐state monomers. This has been explained by lone pair repulsion from the E‐bound N atoms with the *trans*‐bent π‐type orbital that weakens E─E bonding [52, 53]. In this work, we report on a new N‐ligand based on an iminophosphorane scorpionate scaffold and its group 14 element(II) complexes that include a Si─Si bonded N‐heterocyclic silylene dimer, its weaker Ge congener, and different metal–ligand interactions in the solid‐state structures of the Sn and Pb analogs.

## 2 Results and Discussion

### 2.1 Synthesis

**SCHEME 1:**
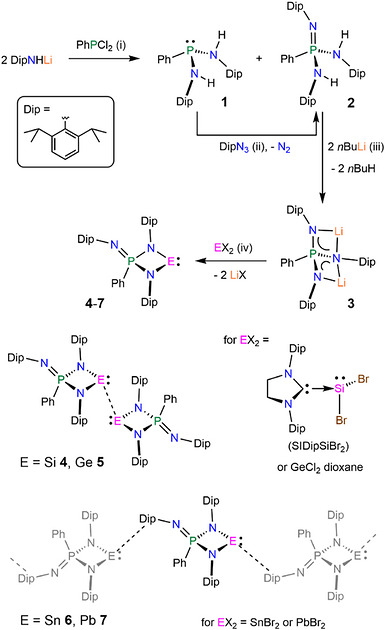
Synthesis of compounds **1**‐**7**; (i) THF, −78°C to r.t.; (ii) toluene, 0°C to 70°C, 30% isolated over 2 steps; (iii) toluene, −78°C to r.t., 94%; (iv) Moderate to high in situ yields in deuterated benzene; Si: benzene, 20% isolated (46% in situ); Ge: THF, −80°C to r.t., 27% isolated; Sn: Et_2_O, −30°C to r.t., 60% isolated; Pb: Et_2_O, −80°C to r.t., 19% isolated.

With the aim of accessing a potential dianionic, sterically demanding iminophosphorane‐based ligand system [54, 55, 56, 57] of the type RP(NAr)_3_^2−^, c.f. related monoanionic R_2_P(NAr)_2_^−^ [56, 57], we first targeted the P^III^ precursor diaminophosphine PhP(NHDip)_2_ **1** via a salt‐metathesis approach between two equivalents of DipNHLi and PhPCl_2_, in a similar reaction to the synthesis of Ph_2_PNHDip [58] (Scheme 1).

**FIGURE 2:**
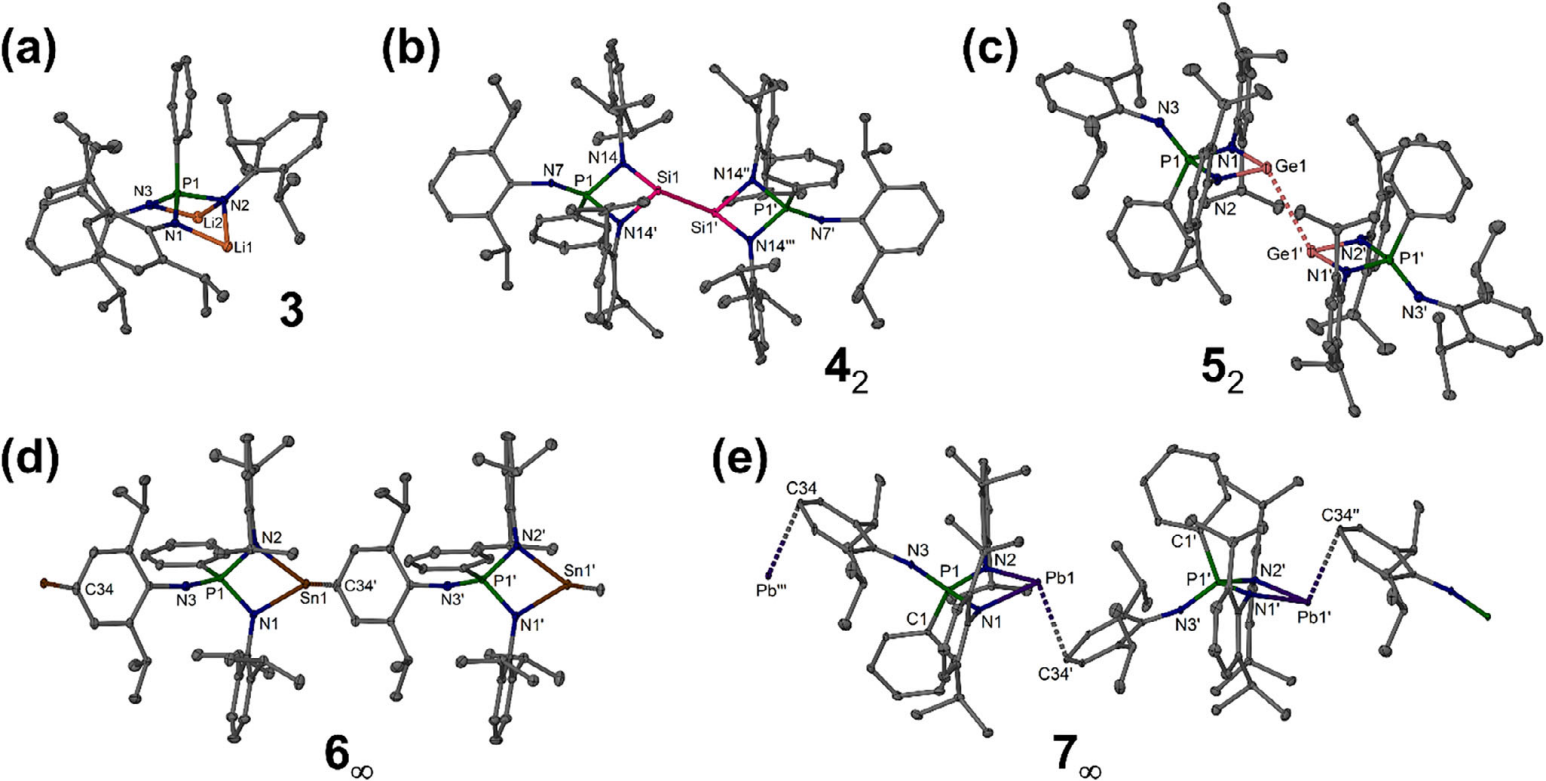
Molecular structures of **3**, **4**_2_, **5**_2_, **6_∞_**, and **7_∞_** (30% thermal ellipsoids). Hydrogen atoms and possible lattice solvent molecules are omitted from the image and formulae. Further information is given in the Supporting Information. Selected bond lengths (Å) and angles (°): **3**: P1–N7 1.598(3), P1–N19 1.650(3), P1–N31 1.628(3), N19–Li1 2.022(7), N31–Li1 1.965(6), N19–Li2 2.025(7), N7–Li2 1.957(6); **4**_2_: Si1–Si1′ 2.5916(9) (Si1–Si2 2.6879(13) for other solvate), Si1–N14 1.7510(11), P1–N14 1.7060(11), P1–N7 1.5333(15); N14–Si1–N14′ 82.51(7), C7–N7–P1 136.52(12), ϑ ca. 55.5 (56.1 mean for solvate); **5**_2_: Ge···Ge 3.1453(10) (Ge···Ge 3.1655(8) for other example), Ge1–N1 1.9044(19), Ge1–N2 1.8503(19), P1–N1 1.6850(19), P1–N2 1.7296(19), P1–N3 1.5661(19); N2–Ge1–N1 81.63(8), C31–N3–P1 144.07(16), *ϑ* ca. 59.45 (60.85 for other example); **6**_∞_: Sn1–N1 2.1380(18), Sn1–N2 2.136(2), Sn1···C34′ 2.793(2), P1–N1 1.6646(18), P1–N2 1.662(2), P1–N3 1.5346(19); N2–Sn1–N1 68.60(7), C31–N3–P1 161.27(16); **7**_∞_: Pb1–N1 2.230(3), Pb1–N2 2.257(3), Pb1···C34′ 2.826(4), P1–N1 1.658(3), P1–N2 1.657(3), P1–N3 1.553(3); N1–Pb1–N2 65.82(11), C31–N3–P1 160.4(3).

Crude compound **1** was obtained as a yellow oil in high in situ yield, could be crystallized from a pentane/THF mixture in one instance as **1**·THF, and was structurally characterized (Figure S70). To our surprise, the target P^V^ proligand PhP(═NDip)(NHDip)_2_ **2**, TipH_2_, was formed alongside **1** in a varying yield of up to 14% and could be crystallized from a hexane extract of the reaction mixture. Furthermore, crude **1** can be converted by reaction with DipN_3_ to **2** at room temperature to elevated temperatures (70°C). TipH_2_ **2** was obtained in a one‐pot procedure from PhPCl_2_ in good in situ yield and 30% crystallized yield on a large scale (Scheme 1). A molecular structure of compound **2** shows the expected connectivity and overall structure (Figure S71). The two acidic NH protons in **2** can be deprotonated with *n*‐butyllithium in toluene to afford [TipLi_2_] **3** as a colorless crystalline material in high yield (94%), see Scheme 1. The molecular structure of an arene solvate of **3** is shown in Figure 2a and can be regarded as an essentially monomeric, donor–solvent‐free dilithium complex of a dianionic ligand aided by the three bulky NDip substituents on one phosphorus atom. The Li centers in **3** show short intramolecular contacts to methyl groups from the Dip substituents, and intermolecular contacts to aryl substituents, leading to a one‐dimensional polymer (Figures S72 and S73). A THF adduct of **3** was also afforded, see the Supporting Information. Although [TipLi_2_] **3** could be readily prepared, the equivalent twofold deprotonation of TipH_2_ **2** with strong sodium or potassium base reagents has so far not been achieved, likely for steric reasons.

Salt‐metathesis of **3** with group 14 element dihalide species (EX_2_; E = Si [59, 60], Ge, Sn, Pb; X = Cl, Br), see Scheme 1, afforded a series of yellow to orange TipE‐type compounds, which were structurally characterized (Figure 2b–e). All structures show a divalent group 14 element center chelated by two nitrogen atoms and a terminal P═NDip and phenyl group on the P^V^ center. These structures revealed two classes of products; TipSi **4** and TipGe **5** were found to crystallize as (TipE)_2_ E─E‐bonded dimers, **4**_2_ and **5**_2_, whereas TipSn **6** and TipPb **7** show a one‐dimensional polymeric (TipE)_∞_ solid‐state structure with E···DipN coordination contacts (Scheme 1), **6**_∞_ and **7**_∞_. Considering these compounds as scorpionate complexes [61, 62] with the P═N─Dip groups as the tail and the terminal *para*‐carbon atom as the stinger, **4**_2_ and **5**_2_ can be regarded as forming head‐to‐head dimers with unengaged stingers, whereas **6**_∞_ and **7**_∞_ are head‐to‐tail coordination polymers linking via the stinger function of the tail.

For (TipSi)_2_, **4**_2_, two molecular structures of solvates ((TipSi)_2_·C_6_H_14_ in Figures 2b and S76, (TipSi)_2_·3 C_6_H_6_ in Figure S77) were afforded, both of which show an unsupported Si─Si bond of 2.5916(9) and 2.6879(13) Å, respectively. While the Si─Si distances between the two structures vary by around 0.1 Å, their *trans*‐bending angles (*ϑ*) are highly similar at ca. 56°. Both structures show highly similar Si─N bond lengths, but the P═N─C tail angle in **4**_2_ with the shorter Si─Si bond (136.52(12)°) is significantly more acute than those in the other (148.2° mean). The Si─Si bond length in **4**_2_ is greater than twice the single bond radius of Si (2.32 Å) [63], similar to that in the geometrically enforced species **IX** with three‐coordinate Si^II^ centers [29], and well within two Si van der Waals radii (4.20 Å) [64]. The Si─Si distances observed in **4**_2_ are slightly longer than those in amidinate silicon(I) dimers (1,2‐disilylenes) with overall related structures but different electron count showing strong σ‐bonds between both Si centers, shorter Si─Si distances (range ca. 2.413−2.489 Å) and larger *trans*‐bending angles (range ca. 66°−78°) [65, 66, 67]. Iminophosphorane‐based ligands have previously been employed to stabilise low oxidation state silicon compounds [68, 69, 70, 71, 72]. These are dominated by three‐coordinate Si systems, and one molecule contains a Si^III^─Si^III^ shared covalent bond of 2.3742(9) Å [70]. To the best of our knowledge, compound **4**_2_ represents the first structurally characterized N‐heterocyclic silylene dimer.

The molecular structure of TipGe **5** is also arranged in a dimeric form in the solid state, **5**_2_, but here the Ge─Ge distance in two structurally characterized examples (3.1453(10) Å with *ϑ* ca. 59.45°, see Figures 2c and S80, and 3.1655(8) Å with *ϑ* ca. 60.85°, see Figure S81) is much larger and well outside twice the Ge single bond radii (2.42 Å) [63] but still within two van der Waals radii (4.22 Å) [64]. Considering the similar radii of Si and Ge, this difference in bond length (ca. 0.5 Å) is considerable. Although the unsupported solid‐state Ge···Ge distances in **5**_2_ are presumed to be very weak, they are broadly similar in value compared to those in a few germanium complexes stabilized via supported N‐ligand systems (Figure S90) [73, 74, 75].

The molecular structures of TipSn **6_∞_** and TipPb **7_∞_** are shown in Figure 2d,e. The one‐dimensional coordination polymer structures are formed by coordination of the P═NDip *para*‐carbon atom to the metal center (E─C: **6_∞_** 2.793(2) Å; **7_∞_** 2.826(4) Å) in a head‐to‐tail fashion, alongside significant widening of the P═N–C tail angle (**6_∞_** 161.27(16)°; **7_∞_** 160.4(3)°) toward almost linear compared with the respective angles in **4**_2_ (136.52(12); 148.2° mean), **5**_2_ (144.07(16); 141.03(15)) or the DFT‐optimized monomers **6** (147.7°) and **7** (148.5°) (Figure S89), vide infra. This widened angle is accompanied by slight elongation of the P═N bond, shortening of the C─N bond, and elongation of the Dip‐C_ipso_–C_ortho_ bonds in the tail unit, and suggests a shift in electron density. The four‐membered chelates show N─E─N angles < 90° (**4**_2_: 82.51(7), **5**_2_: 81.63(8), **6_∞_**: 68.60(7), **7_∞_**: 65.82(11)).

In solution, complexes **4**–**7** all show one sharp singlet in their ^31^P{^1^H} NMR spectra (**4** −10.5; **5** −8.6; **6** −7.7; **7**: 0.4 ppm) and give ^1^H NMR spectra that show one very broad septet and two doublets for the methine and methyl hydrogens of the isopropyl groups of the E‐coordinated N‐Dip groups, respectively, and one sharp septet and one sharp doublet only for the P═NDip isopropyl hydrogen atoms. The broad resonances for **4** at room temperature somewhat sharpen when recorded at 65°C, but the compound retains the overall symmetry. The highly symmetric solution state nature of DipN‐iminophosphorane‐based ligands was also observed for related [Ph_2_P(NDip)_2_]^−^ ligands [57]. This symmetry suggests that complexes **4**–**7** are monomeric in solution. Downfield singlet resonances were found in the ^29^Si{^1^H} NMR spectrum of **4** (117.1 ppm) and the ^119^Sn{^1^H} NMR spectrum of **6** (248.3 ppm). ^1^H NMR spectra of **4** in deuterated benzene and deuterated cyclohexane are highly similar. ^29^Si{^1^H} and ^31^P{^1^H} NMR spectra of **4** show singlets of very similar chemical shift in deuterated benzene, deuterated cyclohexane, and protio‐*n*‐hexane, and ranging from 0°C to 65°C. A DOSY NMR experiment (Figure S69 and Table S1) of a mixture of TipSi **4** and TipH_2_ **2** with comparable molecular shape and size further supports that **4** is monomeric in deuterated benzene solution.

### 2.2 Computational Studies

**FIGURE 3:**
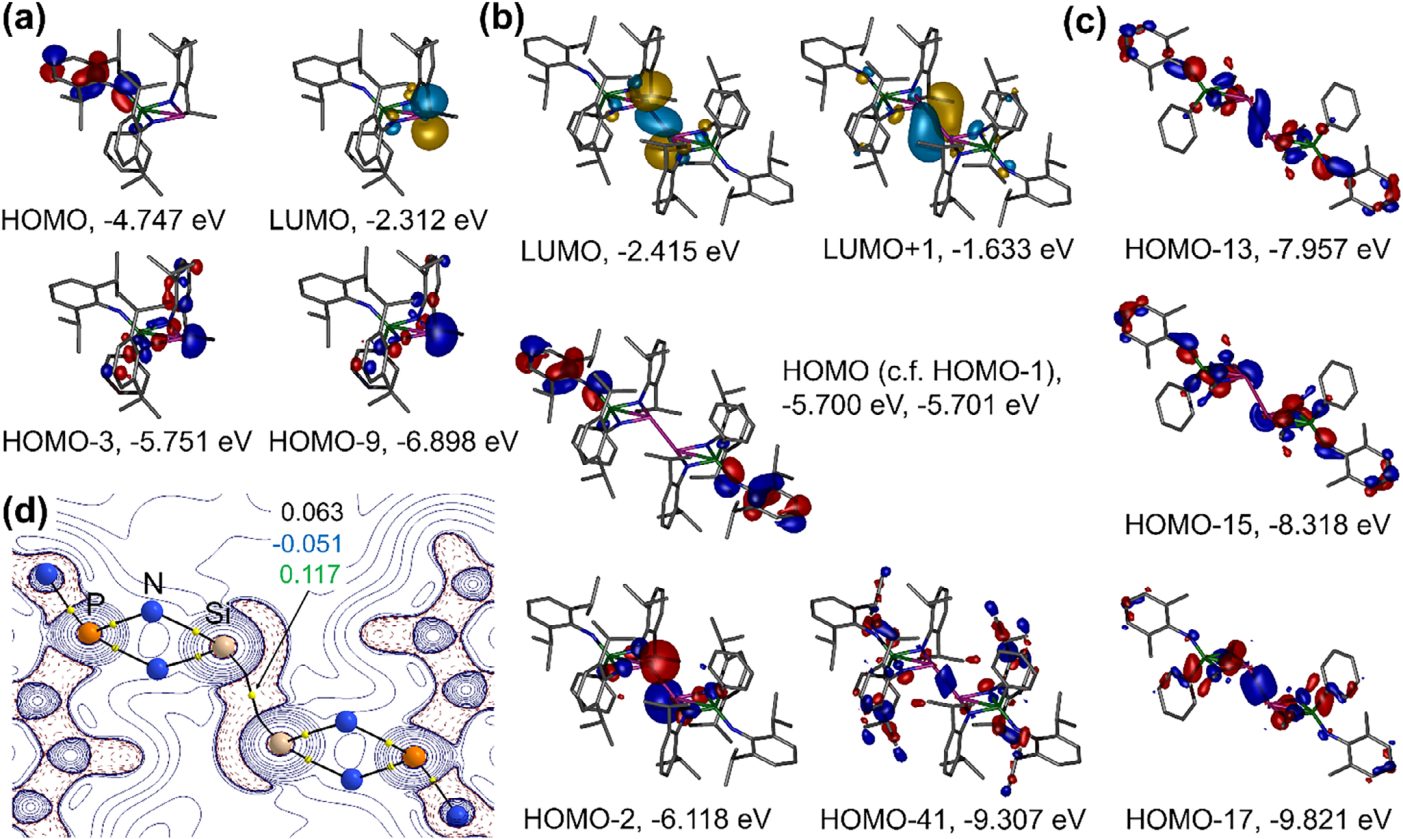
DFT calculations on **4**, **4**_2_ (M06‐D3/def2‐TZVP//M06‐L‐D3/def2‐TZVP level) and **4′**_2_ (B3LYP/def2‐TZVP level). (a) Selected molecular orbitals of TipSi **4** (isovalue 0.06) and (b), selected molecular orbitals of (TipSi)_2_ **4**_2_ (isovalue 0.04) showing the orbital phases in red/blue (occupied orbitals) or yellow/light blue (virtual orbitals). (c) Selected low‐lying orbitals (isovalue 0.04) of **4′**_2_ showing σ‐bonding contributions. (d) QTAIM contour plots showing the Laplacian of the electron density (solid lines: positive, dashed lines: negative) for **4**_2_ (through the PSiSi‐plane but angling the atoms of the (P)N_2_(Si) unit to show bond paths); bond critical points (bcps): yellow, bond paths: black lines, values for the electron density *ρ* [e bohr^−3^] (black), Laplacian, ∇*^2^ρ* [e bohr^−5^] (blue), and bond ellipticity *ε* (green) are given for the bcp between both Si atoms.

We carried out DFT calculations at the M06‐D3/def2‐TZVP//M06‐L‐D3/def2‐TZVP level of theory (from here on referred to as M06D3) for the monomeric TipE complexes **4**–**7** and for the dimers (TipSi)_2_ **4**_2_ and (TipGe)_2_ **5**_2_. The overall geometries of the optimized monomeric molecules agree well with those found by X‐ray diffraction, but the polymeric nature of the solid‐state interactions in TipSn **6_∞_** and TipPb **7_∞_** and the associated distortions were not modeled. The optimized dimer **4**_2_ afforded a Si─Si bonded species with an Si─Si bond of 2.479 Å and a *trans*‐bending angle of 56.3°; the latter agrees very well with values from X‐ray diffraction. The Si─Si bond length in optimized **4**_2_ is ca. 0.1−0.2 Å shorter compared with those obtained from X‐ray diffraction, which is influenced by packing effects including intra‐ and intermolecular dispersion forces, but is close to that found in DFT‐optimized **IV** [23]. Attempting to optimize **4**_2_ at the B3LYP/def2‐TZVP level without dispersion contributions (from here on referred to as B3LYP) led to the dissociation of the dimer beyond a distance of two silicon van der Waals radii [64]. Therefore, we studied the sterically unencumbered cut‐back model system (Tip'Si)_2_ **4′**_2_, where Tip′ = PhP(═NXyl)(NMe)_2_, Xyl = 2,6‐Me_2_C_6_H_3_ (B3LYP), that is, without dispersion. This did optimize **4′**_2_ to a Si─Si bonded species (Si─Si 2.661 Å, *ϑ* 57.2°) where both the Si─Si bond length and the *trans*‐bending angle agree remarkably well with X‐ray data obtained for **4**_2_ (vide supra). Similarly, the dimer (TipGe)_2_ **5**_2_ could be optimized with a Ge─Ge bond length of 2.892 Å (M06D3), which is again somewhat shorter compared to the distances found in the solid‐state. The cut‐back model (Tip'Ge)_2_ **5′**_2_ (B3LYP), c.f. **4′**_2_, provided a much larger Ge···Ge bond distance (3.614 Å), ca. 1 Å longer than the Si─Si bond in **4′**_2_, but with similar *ϑ*. Figure 3a shows selected DFT calculated orbitals of the TipE **4** monomer. The HOMO contains π‐electron density of the P═N─Dip tail, whereas the Si lone pair is part of lower‐lying orbitals (HOMO‐3, ‐9). The LUMO is a Si *p*‐orbital. In the DFT‐calculated dimer (Figure 3b), the ligand tail is again part of the HOMO and HOMO‐1, and the HOMO‐2 contains the out‐of‐phase combination of the Si lone pairs with largely non‐bonding character that shows σ*‐symmetry with respect to Si─Si bonding. Low‐lying orbitals contain contributions for the respective in‐phase σ‐electron density between both Si atoms as part of σ‐bonding orbitals, for example, the HOMO‐41, which is 3.2 eV lower in energy than the Si‐based HOMO‐2 (Figure 3b). Low‐lying orbitals in the related cutback model **4′**_2_ visualize the Si─Si bonding contributions better (Figure 3c). The NBO second‐order perturbation theory analysis indicates that the main Si─Si bonding interaction in **4**_2_ can be viewed as a twofold donor–acceptor interaction where a Si lone pair (78.6% s, 21.3% p) donates into an empty Si‐based orbital contribution (*p*‐orbital), which would support the simplified donor‐acceptor interaction model for bonding in heavier alkene analogs [1, 2, 3, 4, 5]. Element–element bonding in heavier main group alkene analogs is typically weakened by lone pair repulsion from the adjacent π‐donor N(R) substituents at the central main group element, which lowers acceptor properties of the empty main group element *p*‐orbital [40, 53]. No structurally characterized examples of heavier alkene analogs of Si, Ge, and Al with N,N‐chelating ligands and strong bonding are known, and for Ga, only the weakly bonded digallium structures **XVII** and **XVIII** have been reported. The virtual orbitals of **4**_2_ show the *p*–*p*–σ‐bond (LUMO) and a *p*–*p*–π‐bond (LUMO+1), see Figure 3b. These types of orbitals are reminiscent of the Si─Si bonding in amidinate silicon(I) dimers, for example, {*t*BuC_6_H_4_C(NDip)_2_Si}_2_ **8** [66], which, in effect, contain two more electrons in the Si_2_ unit compared with **4**_2_ and show a *p*–*p*–σ‐bond HOMO (88.7% *p*‐character) and a *p*–*p*–π‐bond LUMO [65, 66, 67]. For compound **8**, values for the bond dissociation energy varied significantly depending on the model and method used. We reoptimized **8** (M06D3) for comparison and found an Si─Si bond length (2.482 Å, c.f. 2.560 Å at the original RI‐BP86/def2‐TZVPP level [66]) that is in very good agreement with that obtained from X‐ray diffraction (2.4885(15) Å). Because the Si─Si bonding in compound **4**_2_ does not contain the main σ‐bonding components of the Si─Si bonding in **8** (the HOMO of **8** is analogous to the LUMO in **4**_2_), it is surprising that the Wiberg bond indices (WBIs) of **4**_2_ (1.03), **4′**_2_ (0.752), and **8** (0.803) are of similar magnitude despite **8** containing essentially two more bonding electrons (Table S7). The bonding in **4**_2_ can also be compared to that found for dimeric aluminum and gallium species **XVI**‐**XVIII** with N,N‐chelating ligands, which contain relatively weak σ‐bonding interactions that show similarly significant bond indices near 1 [40, 42].

Across the series **4**–**7** and the dimers **4**_2_ and **5**_2_, the HOMO constitutes π‐density on the electron‐rich P═N─Dip tail, and the LUMO is dominated by an out‐of‐(NEN)‐plane E *p*‐orbital for **4**–**7** or the respective *p*–*p*–σ‐bond for the dimers **4**_2_ and **5**_2_. The P═N─Dip angles are more acute in the DFT‐optimized monomeric molecules **6** (147.7°) and **7** (148.5°) compared with the angles in **6_∞_** (161.27(16)°) and **7_∞_** (160.4(3)°) as obtained from X‐ray diffraction, highlighting the geometric variability of the P═N─Dip tail unit involved in the head‐to‐tail coordination. The NPA atomic charges are summarized in Figure S112 and show that a significant anionic charge is located at the P═N─Dip *para*‐ (and *meta*‐) carbon centers (*ca*. −0.2 each), which are involved in the donation to the Sn^II^ and Pb^II^ centers in **6_∞_** and **7_∞_**; that is, the “stinger in the scorpionate tail”. The *ΔE*_s‐t_ (Table S6) decreases upon dimerization for **4** and may hint at appreciable diradicaloid contributions [33, 39, 40, 76] for Si─Si‐bonded **4**_2_, which was not probed in this work. The HOMO–LUMO gap (Figure S113) widens significantly upon dimerization for Si (from 2.435 eV in **4** to 3.285 eV in **4**_2_), largely due to a significant lowering of the HOMO energy, whereas for Ge it narrows upon dimerization (from 3.300 eV in **5** to 2.629 eV in **5**_2_) due to a decrease in the LUMO energy. This provides some explanation for the stabilization of a dimer for Si compared with Ge. Across the monomers **5**–**7**, the HOMO slightly increases in energy down the group, possibly due to poorer electrostatic stabilization of the dianionic Tip^2−^ ligand unit with a larger E^II^ center down the group (c.f. the decreasing N─E─N angles down the group), and the E *p*‐orbital (LUMO) energy decreases down the group. This leads to a low HOMO–LUMO gap (Figure S113) for Sn (2.63 eV) and Pb (2.32 eV), which likely contributes to the HOMO donating electron density into the LUMO as found in the solid‐state coordination polymer structures, in addition to electrostatic stabilization from the interaction of the electron‐rich tail‐end coordinating to a poorly saturated cationic E^II^ center.

A QTAIM study on the silicon(II) dimers **4**_2_ (see Figure 3d), and **4** **′**_2_ (Figure S97) shows a large region of negative Laplacian and concentration of electron density around the bond critical point (bcp) between the two Si centers, whereas the Ge─Ge bonded dimers show a positive Laplacian (Figures S104 and S106), and less (**5**_2_) to no (**5′**_2_) significant σ‐bonding orbital overlap (Figures S102, S103, and S105), as well as lower WBIs, see Table S7 for a comparison of the E─E bonding interactions. The computed electron density in **4**_2_ is similar to that determined for Driess’ compound **IX** with three‐coordinate Si^II^ centers [29]. The Laplacian plot of **4**_2_ looks broadly similar to that obtained for the amidinate silicon(I) species **8** with two more electrons at the Si_2_ unit although the values for the electron density (+21%) and the Laplacian (+90%) at the bcp are not surprisingly larger for **8** compared with **4_2_** (Table S7). For comparison, the digallium species **XVIII** only shows a very small area of negative Laplacian around the bcp and a low Laplacian value (−0.007 e bohr^−5^) [42].

The reaction of two monomers to one dimer (Table S5) was studied by DFT calculations (gas phase) and afforded exergonic dimerizations for **4**_2_ (*ΔG*_298_ = −17.2 kcal mol^−1^, *ΔH*_298_ = −34.9 kcal mol^−1^) and **5**_2_ (*ΔG*_298_ = −12.1 kcal mol^−1^, *ΔH*_298_ = −28.9 kcal mol^−1^) at the M06D3 level of theory, which will be strongly affected by a significant dispersion contribution [4, 49, 50]. For the cutback models studied at the B3LYP level without dispersion, we have found a positive *ΔG*_298_ (+9.8 kcal mol^−1^) but negative *ΔH*_298_ (−1.1 kcal/mol) for the dimerization to **4′**_2_ and a mildly endergonic reaction to form **5′**_2_ (*ΔG*_298_ = +7.1 kcal mol^−1^, *ΔH*_298_ = +1.5 kcal mol^−1^); the latter is not surprising when the very large Ge···Ge distance in this structure is considered. The zero‐point‐energy‐corrected internal energies show similar magnitudes compared with the *ΔH*_298_ values. An energy scan along the Si─Si bond of **4′**_2_ (Figure S98, B3LYP) from the equilibrium distance to approximately 2 Si van der Waals radii shows an increase of ca. 2.1 kcal mol^−1^and an expected curve profile; Si─Si bond stretching and compressing is a low‐energy process. The computational studies provide support for significant Si─Si bonding in **4**_2_, based on a combination of bond distance, orbital overlap, Laplacian of electron density distribution, WBI, and comparison to related molecules. As discussed, π‐donor substituents from N(R) groups weaken E─E bonding interactions in heavier alkene analogs and thus, experimentally observed species with two N‐substituents per E center as in **4**_2_ are very rare [41, 42, 53]. In the present case, the π‐donor effect may be decreased due to the directly N‐bonded, positively charged P^V^ center in the Tip‐ligand framework. Both the small chelate ring‐size and the sterics of the ligand contribute to a fortuitously balanced geometry in **4**_2_ (Si─Si bond length, *ϑ*) that allows isolation of the dimeric form. Because dimer formation is energetically dominated by dispersion forces and dissociation of **4**_2_ is possible without apparent kinetic hurdles, dissolution in solvents will add dispersive stabilization from molecule–solvent interactions [4, 49, 50]. In addition, entropic factors make dimer formation less viable (*ΔG* vs. *ΔH*) as has been pointed out [39], especially in solution, where monomer **4** is expected to form easily.

### 2.3 Reactivity of TipSi 4

**SCHEME 2:**
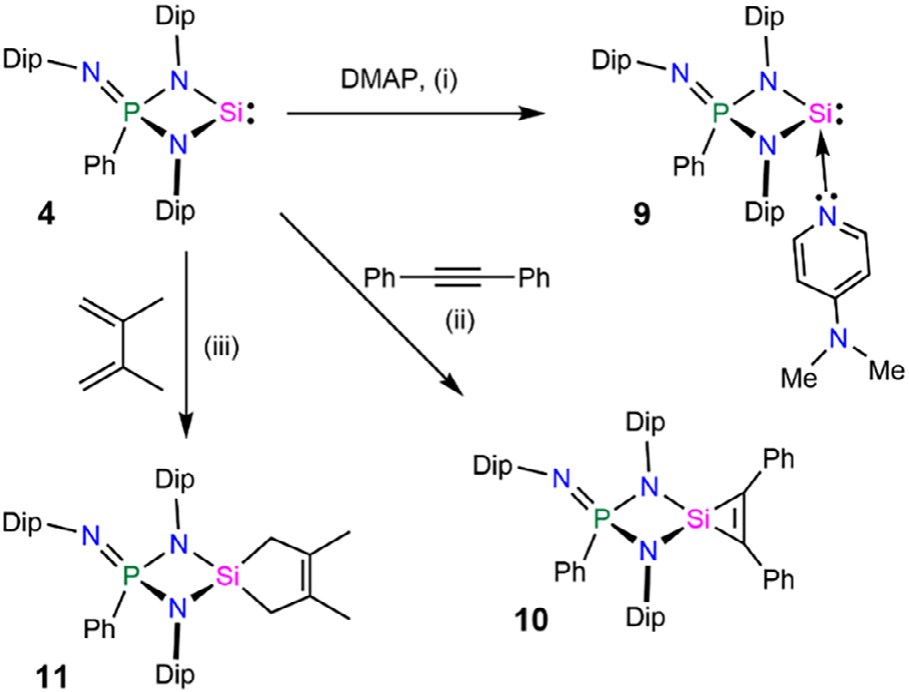
Synthesis of compounds **9**–**11**; (i) deuterated benzene, r.t., quantitative in situ; (ii) deuterated benzene, r.t., ca. 78% in situ; (iii) deuterated benzene, r.t., ca. 72% in situ.

**FIGURE 4:**
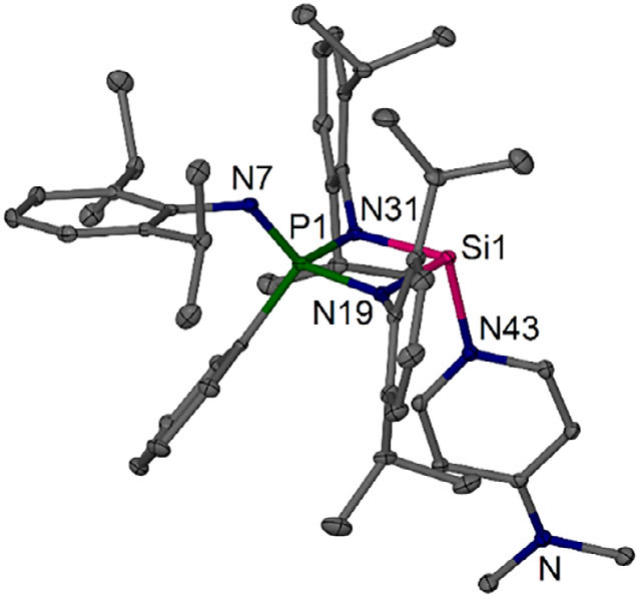
The molecular structure of TipSi(DMAP)·C_6_H_6_·0.25C_6_H_14_, **9**·C_6_H_6_·0.25C_6_H_14_, shown as 30% thermal ellipsoids. Only one of two independent molecules of **9** is shown; hydrogen atoms are omitted. Selected bond lengths (Å) and angles (°): shown molecule: Si1–N19 1.8126(16), Si1–N31 1.8338(17), Si1–N43 1.9499(16), P1–N7 1.5522(16), P1–N19 1.6711(17), P1–N31 1.6824(16), P1–C1 1.819(2), N7–C7 1.414(2); N19–Si1–N31 78.54(7), N19–Si1–N43 99.66(7), N31–Si1–N43 100.15(7), C7–N7–P1 127.80(13).

NMR spectroscopy indicated that TipSi **4** and its heavier congeners are monomeric in deuterated benzene solution. To further probe the properties of compound **4** and to assess whether it behaves as a dimer or monomer, some reactivity studies were undertaken. To probe the accessibility of the relatively low‐lying empty *p*‐orbital, a solution of **4** was treated with one equivalent of DMAP which immediately formed the bright yellow adduct TipSi(DMAP) **9**, see Scheme 2 and Figure 4. The molecular structure of TipSi(DMAP) **9** is a rare NHSi donor adduct, showing the expected three‐coordinate Si^II^ center with slightly elongated Si─N distances compared to its precursor **4**_2_, and is similar to the DMAP adduct of (nacnac′)Si:, where nacnac′ = H_2_CC(NDip)CHC(NDip)Me [77]. The DMAP ligand coordinates via the face of the PN_2_Si plane that bears the smaller (P)Ph group, which rotates to a more perpendicular position with respect to the DMAP plane to accommodate the donor ligand. The angle of the P═N─C tail, which houses the HOMO in this electron‐rich Si^II^ compound, is now much more acute (127.80(13)°) compared to those in **4**–**7** and may indicate that the tail geometry is affected by, and can aid in moderating, electron density at the PN_2_E plane. In a similar manner, treating TipSn **6** with THF afforded the formation of the adduct TipSn(THF) as suggested by NMR spectroscopy, and shows the electrophilic nature of these complexes.

We next treated TipSi **4** with either diphenylacetylene or 2,3‐dimethylbuta‐1,3‐diene in deuterated benzene at room temperature (Scheme 2). Both reactions were accompanied by a rapid color change and showed full conversion of **4** to one main product each within 1 h, as monitored by NMR spectroscopy. In both cases, the expected silacyclopropene TipSi(CPh)_2_ **10** and silacyclopentene TipSi{CH_2_C(Me)}_2_ **11** products were afforded in high in situ yields, and subsequently, single crystals of the expected products were obtained. The mild reaction conditions show the high reactivity of **4**, and the product outcomes support a typical reactivity pattern of a monomeric silylene species [14, 15, 16, 17, 18]. For comparison, the reactivity of compound **IX** with two geometrically enforced Si centers in close proximity to diphenyl acetylene afforded a Si^III^ product that involves both Si centers in substrate reactivity [29]. The molecular structures of compounds **10** (Figure S87) and **11** (Figure S88) were determined, showing the expected atom connectivity, but due to poor overall ordering, they are not discussed here in detail.

## 3 Conclusion

In conclusion, we have synthesized a new scorpionate‐like dianionic “triiminophosphorane”‐based ligand system (Tip^2−^) that forms stable, structurally characterized complexes with divalent group 14 element centers Si‐Pb. Compound (TipSi)_2_ **4**_2_ shows relatively short Si─Si bonding in the solid‐state and represents the first structurally characterized example of a heretofore elusive N‐heterocyclic silylene dimer. Si─Si bonding is not retained in benzene solution, and compound **4** behaves as a reactive monomeric silylene species. Computational studies show that dimerization to **4**_2_ is favorable, but energetically dominated by dispersion effects, and due to the stability of the monomers, dissociation is largely driven by entropic effects, for example, upon dissolution. At the observed Si─Si distance, significant Si─Si bonding is present in **4**_2_ and values for WBI, electron density, and, to a lesser extent, the Laplacian are surprisingly similar to those found for the amidinate silicon(I) dimer {*t*BuC_6_H_4_C(NDip)_2_Si}_2_ **8**, which contains two more bonding electrons and forms a robust Si─Si bond in contrast. The weak dimers observed in (TipGe)_2_ **5**_2_ show much longer and weaker Ge─Ge bonding despite similar radii of Si and Ge. The heavier congeners TipSn **6** and TipPb **7** do not form E─E interactions in the solid state but instead coordinate in a head‐to‐tail fashion to the *para*‐carbon atom of the electron‐rich DipN═P tail of a neighboring unit, leading to one‐dimensional coordination polymers (TipE)_∞_. The size, shape, and electronics of the dianionic ligand allow an Si─Si bonding interaction in **4**_2_ to form at both an appropriate distance (*D*) and *trans*‐bending angle (*ϑ*) that seems to be optimal for element‐element bonding, for example, when the core structure is compared to that of the DFT‐computed cutback model **4′**_2_ without dispersion contributions. The π‐donor substituents of the N(Dip) groups adjacent to Si are expected to weaken Si─Si bonding more generally, but this effect may be diminished in **4**_2_ because both N(Dip) groups also bond to the positively charged phosphazene P^V^ center in the four‐membered ring. By adjusting the angle of the electron‐rich P═N─Dip tail, the ligand appears to be able to respond to changes in electronic requirements or to offer an additional coordination site via engaging the scorpionate stinger in the polymeric structures (TipE)_∞_.

## Conflicts of Interest

The authors declare no conflicts of interest.

## Supporting information



The authors have cited additional references within the Supporting Information [78, 79, 80, 81, 82, 83, 84, 85, 86, 87, 88, 89, 90, 91, 92, 93, 94, 95, 96, 97, 98, 99, 100, 101, 102, 103, 104, 105, 106, 107, 108, 109, 110, 111].

## Data Availability

Selected data that support the findings of this study are available in the Supporting Information of this article. CCDC 2495397–2495416 contains the supplementary crystallographic data for this paper. These data can be obtained free of charge from The Cambridge Crystallographic Data Centre via www.ccdc.cam.ac.uk/structures. The research data (NMR spectroscopy, DFT computational studies) supporting this publication can be accessed at https://doi.org/10.17630/003ef35c‐bb10‐42af‐8cf8‐02555a3e9c3e.
