# Supplementary material for: Si─Si Bonding in an Unsupported N‐Heterocyclic Silylene Dimer Stabilized by an Iminophosphorane‐Based Scorpionate Ligand Versus Head‐to‐Tail Coordination in the Sn and Pb Tetrylenes
Source: Chemistry. 2026 Apr 17;32(25):e70980. doi: 10.1002/chem.70980 (PMC13331589; doi:10.1002/chem.70980)
Supplement: Supplementary file 1 — The authors have cited additional references within the Supporting Information [78, 79, 80, 81, 82, 83, 84, 85, 86, 87, 88, 89, 90, 91, 92, 93, 94, 95, 96, 97, 98, 99, 100, 101, 102, 103, 104, 105, 106, 107, 108, 109, 110, 111]. [file CHEM-32-e70980-s001.pdf]

Supplementary Information

for

Si–Si bonding in an unsupported N-heterocyclic silylene dimer stabilised by an iminophosphorane-based scorpionate ligand versus head-to-tail coordination in the Sn and Pb tetrylenes

*Huanhuan Dong, Rochelle Ferns, Luke W. Giles, Lea Fohlmeister, Connor Bourne, Samuel R. Lawrence, Aidan P. McKay, Alexandra M. Z. Slawin, David B. Cordes, Tanja van Mourik and Andreas Stasch**

Table of Contents

1	Experimental Section	2
2	NMR spectroscopy	14
3	X-ray crystallography	83
4	Computational studies	121
5	References	166

1 Experimental Section

1.1 General considerations

All manipulations were carried out using standard Schlenk and glove box techniques under a dry argon or dinitrogen atmosphere unless described below (for organic condensation reactions and aqueous organic workup steps). Benzene, toluene, diethyl ether, THF, *n*-hexane and *n*-pentane were either dried and distilled under inert gas over $LiAlH_4$, sodium or potassium, or taken from an MBraun solvent purification system and degassed prior to use. ^{1}H, ^{7}Li, $^{13}C\{^{1}H\}$, and $^{31}P\{^{1}H\}$ NMR spectra were recorded on a Bruker AV 300, Bruker AVII 400 or Bruker AV III 500 spectrometer in deuterated chloroform or benzene and were referenced to the residual ^{1}H or $^{13}C\{^{1}H\}$ resonances of the solvent used, or external aqueous LiCl or H_3PO_4 solutions, respectively. $^{29}Si\{^{1}H\}$ NMR and ^{119}Sn NMR were referenced to tetramethylsilane in $CDCl_3$ or Bu_3SnCl in $CDCl_3$. Chemical shifts are given in ppm. Abbreviations: s = singlet, d = doublet, q = quartet, sept = septet, br = broad, m = multiplet. IR spectra were obtained on a Shimadzu IR Affinity spectrometer with ATR attachment or using a Perkin–Elmer RXI FT-IR spectrometer as Nujol mulls between NaCl plates. Melting points were determined using a Gallenkamp apparatus in sealed glass capillaries under argon and are uncorrected. Yields or conversions in solution were determined by integration of ^{1}H NMR spectra against an internal standard (such as hexamethylbenzene or adamantane). The elemental analysis was performed by the Elemental Analysis Service at London Metropolitan University. $DipN_3$,[1] BzlK,[2] (SIDip)$SiBr_2$[3] and (IDip)SiX_2 (X = Br or Cl)[4] were synthesised as described in the literature. All other reagents were used as received. Although the synthesis of PhP(NHDip)$_2$ **1** has not been reported in the literature, the molecular structure of a dilithium complex, [PhP(NDip)$_2$(LiOEt$_2$)$_2$] has been reported.[5] Bulky P^{III}-N compounds similar to **1**, and Li and group 14 element complexes of these, are known.[6]

1.2 Syntheses

Synthesis of PhP(NHDip)$_2$ 1

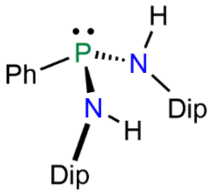

To a cold (-78 °C) stirring solution of 2,6-diisopropylaniline (DipNH$_2$, 12.1 mL, 73.6 mmol, 2 equiv.) in THF (50 mL), was added *n*BuLi (48.3 mL of a 1.6 M solution in hexane, 77.3 mmol, 2.05 equiv.) dropwise and the reaction solution was stirred at this temperature for 0.5 h before being allowed to warm to room temperature whilst stirring for an additional 0.5 h. The afforded colourless solution was added dropwise to a cold (-78 °C) solution of PhPCl$_2$ (5.06 mL, 6.67 g, 37.3 mmol, 1 equiv.) in THF (20 mL), and the mixture was vigorously stirred for one hour, during which a colour change from orange to bright yellow was observed. The reaction mixture was allowed to warm to room temperature and stirred for another hour before all volatiles were removed. The yellow residue was extracted with *n*-pentane (30 mL). (Note: If all volatiles are removed, an oily residue is obtained that contains predominantly PhP(NHDip)$_2$ **1** as the main product and varying quantities of TipH$_2$, PhP(=NDip)(NHDip)$_2$, **2** as a by-product. This mixture can be used for further conversion with DipN$_3$ to afford compound **2**.) THF (ca. 2 mL) was added and large colourless crystals were observed after storing the solution at -40 °C for two weeks, which was isolated as PhP(NHDip)$_2$·THF, **1**·THF. Yield = 10.4 g (64%). A yellow sticky oil of **1** was obtained when attempting to remove THF from the product under vacuum. ^{1}H NMR (benzene-*d*$_6$, 400.1 MHz, 298 K): δ = 1.12 (d, J_{HH} = 6.8 Hz, 12H, NHDip-*i*Pr-C*H*$_3$), 1.13 (d, J_{HH} = 6.9 Hz, 12H, NHDip-*i*Pr-C*H*$_3$), 3.40 (sept, J_{HH}= 6.8 Hz, 2H, NHDip-*i*Pr-C*H*), 3.41 (sept, J_{HH}= 6.8 Hz, 2H, NDip-*i*Pr-C*H*), 4.45 (d, J_{HP} = 6.2 Hz, 2H, 2 × N*H*), 7.04 – 7.11 (m, 7H, Ar-C*H*), 7.18 – 7.22 (m, 2H, Ar-C*H*), 7.76 – 7.81 (m, 2H, Ph-*o*-C*H*). ^{13}C{^{1}H} NMR (benzene-*d*$_6$, 125.7 MHz, 298 K): δ = 24.1 (*i*Pr-*C*H$_3$), 24.3 (*i*Pr-*C*H$_3$), 28.7 (*i*Pr-*C*H), 28.8 (*i*Pr-*C*H), 124.1 (Dip-Ar-*m*-*C*H), 129.03 (d, J_{CP} = 5.7 Hz, Ph-*m*-*C*H), 129.4 (Ph-*p*-*C*H), 129.7 (d, J_{CP} = 20.0 Hz, Ph-*o*-*C*H), 139.1 (d, J_{CP} = 9.8 Hz, Dip-Ar-*ipso*-*C*), 141.9 (d, J_{CP} = 2.3 Hz, Dip-Ar-*o*-*C*), 145.2 (d, J_{CP} = 8.3 Hz, Ph-*ipso*-*C*). ^{31}P{^{1}H} NMR (benzene-*d*$_6$, 162.0 MHz, 295 K): δ = 69.4 ppm (s). IR (ATR), *v*~/cm^{-1}: 3059w, 2959m, 2928m, 2866m, 1589w, 1437s, 1360s, 1329m, 1252m, 1101s, 891m, 868m, 741s, 698s.

Synthesis of $TipH_2$, $PhP(=NDip)(NHDip)_2$, 2

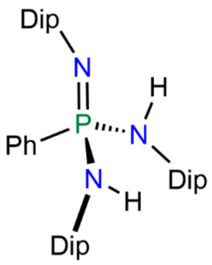

Method one:

To a Schlenk flask containing a cold solution (0 °C) of $DipNH_2$ (17.0 mL, 90.1 mmol, 2.0 equiv.) in diethyl ether (30 mL) was added *n*BuLi (62.0 mL of a 1.6 M solution in hexane, 99.1 mmol, 2.2 equiv.) dropwise, and the reaction mixture was warmed to room temperature and stirred for one hour. The reaction mixture was cooled again to 0 °C, and $PhPCl_2$ (6.50 mL, 8.57 g, 47.9 mmol, 1.0 equiv.) was added dropwise. The resulting solution was stirred at room temperature for 18 hours before it was filtered. All volatiles were removed from the filtrate, and the residue was extracted with *n*-hexane (ca. 150 mL), under reduced pressure to ca. 30 mL and stored at -35 °C overnight to yield colourless crystals of $TipH_2$ **2**. Yield = 2.61 g (14%).

Method two:

To a Schlenk flask containing a cold solution (0 °C) of $DipNH_2$ (32.9 g, 186 mmol, 2.05 equiv.) in diethyl ether (30 mL) was added *n*BuLi (116 mL of a 1.6 M solution in hexane, 186 mmol, 2.05 equiv.) dropwise and the reaction mixture was warmed to room temperature and stirred for one hour. The reaction mixture was cooled again to 0 °C, and $PhPCl_2$ (12.5 mL, 16.5 g, 92.1 mmol, 1 equiv.) was added dropwise. The yellow solution was warmed to room temperature and stirred for an additional 15 hours. The solution was filtered, all volatiles were removed under reduced pressure, and the oily residue was taken up in toluene (30 mL). To this, a solution of $DipN_3$ (14.2 g, 69.5 mmol, 1.15 equiv.) in toluene (ca. 100 mL) was slowly added at -20 °C. The reaction mixture was warmed to room temperature and slowly heated (open to inert gas) to 70 °C and stirred for three hours. After cooling, all volatiles were removed under vacuum, and the honey-like residue was extracted into *n*-hexane (50 mL) (or *n*-pentane alternatively). Storing the solution at 5 °C afforded successive crops (after further concentration of the supernatant solution) of crystalline colourless $TipH_2$ **2**. Yield = 17.6 g (30%). Note: NMR spectroscopy suggests higher *in-situ* yields, and the low to moderate isolated yield is due to the requirement to crystallise the product for isolation. M.p.: 142-144 °C. ^{1}H NMR (benzene-d_6, 400.1 MHz, 298 K): δ = 0.90 (d, J_{HH} = 6.8 Hz, 12H, NHDip-*i*Pr-CH_3), 1.02 (d, J_{HH} = 6.8 Hz, 12H, NHDip-*i*Pr-CH_3), 1.23 (d, J_{HH} = 6.8 Hz, 12H, NDip-*i*Pr-CH_3), 3.47 (sept, J_{HH}= 6.9 Hz, 4H, NHDip-*i*Pr-C*H*), 3.77 (sept, J_{HH}= 6.9 Hz, 2H, NDip-*i*Pr-C*H*), 4.37 (d, J_{HP} = 7.5 Hz, 2H, 2 × N*H*), 6.94 (m, 3H, Ph-C*H*), 7.00 (m, 6H, NHDip-Ar-C*H*), 7.05 (m, 1H, NDip-Ar-*p*-C*H*), 7.22 (d, J_{HH} = 1.6 Hz, 2H, NDip-Ar-*m*-C*H*), 7.78 – 7.95 (m, 2H, Ph-*o*-C*H*). $^{13}C\{^{1}H\}$ NMR (benzene-d_6, 100.1 MHz,

300 K): δ = 23.7 (*i*Pr-*C*H_3), 24.0 (*i*Pr-*C*H_3), 24.3 (*i*Pr-*C*H_3), 28.5 (*i*Pr-*C*H), 28.7 (*i*Pr-*C*H), 120.1 (NDip-Ar-*C*H), 123.3 (NDip-Ar-*C*H), 123.9 (NHDip-Ar-*C*H), 126.8 (NHDip-Ar-*C*H), 127.5 (NHDip-Ar-*C*H), 127.7 (NHDip-Ar-*C*H), 131.0 (d, J_{CP} = 2.4 Hz, Ph-*C*H), 132.6 (d, J_{CP} = 161.6 Hz, Ph-*ipso*-*C*), 133.5 (d, J_{CP} = 8.9 Hz, Ph-*o*-*C*H), 134.6 (d, J_{CP} = 4.6 Hz, NHDip-Ar-*o*-*C*), 141.8 (d, J_{CP} = 7.6 Hz, NDip-Ar-*o*-*C*), 144.2 (NDip-Ar-*ipso*-*C*), 146.5 (d, J_{CP} = 3.2 Hz, NHDip-Ar-*ipso*-*C*). $^{31}P\{^1H\}$ NMR (benzene-d_6, 162.0 MHz, 295 K): δ = -20.2 ppm (s). IR (nujol), ν~/cm^{-1}: 3377m, 3060w, 2958m, 2923m, 2863m, 1588w, 1433s, 1360s, 1333m, 1252m, 1106s, 921m, 907m, 789s, 751s.

Synthesis of [TipLi$_2$] 3

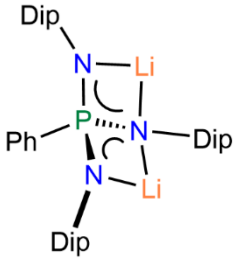

To a cold (-78 °C) stirring solution of TipH$_2$ **2** (1.50 g, 2.36 mmol, 1 equiv.) in toluene (30 ml) was added *n*BuLi (1.6 M in *n*-hexane, 3.17 mL, 5.07 mmol, 2.15 equiv.) dropwise and the reaction mixture was stirred at low temperature for one hour, during which the formation of a white precipitate was observed. The yellow reaction mixture was slowly warmed to room temperature and stirred overnight. The resulting solution was filtered, and the residue was dried *in vacuo*, yielding the crude product as a white powder that was poorly soluble in benzene-d_6. The NMR spectra were thus obtained at elevated temperature or after a drop of THF was added. Colourless crystals of solvates of [TipLi$_2$] **3** suitable for single crystal X-ray diffraction were obtained by cooling a warm, concentrated benzene or toluene solution of **3**. Crystals of [Tip(LiTHF)$_2$], **3**(THF)$_2$, were obtained from mixtures in toluene/THF. Yield = 2.10 g (94%). Mp.: decomposes to a brownish oil above 211 °C. ^1H NMR (400.1 MHz, benzene-d_6/THF, 294 K): δ = 1.15 (d, J_{HH} = 6.7 Hz, 36H, *i*Pr-C*H*$_3$), 4.09 (sept, J_{HH} = 6.5 Hz, 6H, *i*Pr-C*H*), 6.88–7.15 (m, 12H, Ar-*H*), 7.13 (m, 2H, Ar-*H*). $^{13}C\{^1H\}$ NMR (benzene-d_6/THF, 125.7 MHz, 298 K): δ = 23.9 (*i*Pr-*C*H_3), 28.2 (*i*Pr-*C*H), 118.9 (d, J_{CP} = 3.3 Hz, Dip-Ar-*C*H), 123.2 (d, J_{CP} = 2.7 Hz, Dip-Ar-*C*H), 126.4 (d, J_{CP} = 2.4 Hz, Ph-C*H*), 126.8 (d, J_{CP} = 10.8 Hz, Ph-*C*H), 130.5 (d, J_{CP} = 7.2 Hz, Ph-*CH*), 143.9 (d, J_{CP} = 6.3 Hz, Dip-Ar-*C*), 144.7 (d, J_{CP} = 114.7 Hz, Ph-*ipso*-C), 149.9 (d, J_{CP} = 4.9 Hz, Dip-Ar-*C*). $^{31}P\{^1H\}$ (202.4 MHz, benzene-d_6/THF, 298 K): δ = -3.7 ppm (s). $^7Li\{^1H\}$ (155.5 MHz, benzene-d_6/THF, 300 K): δ = 0.63 ppm (s). IR (nujol), ν~/cm^{-1}: 3049w, 2954m, 1586w, 1413s, 1345m, 1315s, 1272s, 1243s, 1196s, 1139w, 1112m, 1044m, 1001m, 970s, 941m, 769s.

Synthesis of [(TipH)K]

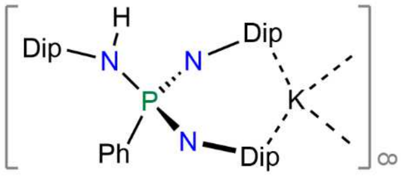

Toluene was added (20 mL) to a solid mixture of $TipH_2$ **2** (100 mg, 0.157 mmol, 1 equiv.) and benzyl potassium (20.5 g, 0.157 mmol, 1 equiv.) at -30 °C, and the reaction mixture was stirred for 15 minutes. It was then slowly warmed to room temperature and stirred overnight, during which a colourless solution formed (after ca. 10 minutes). All volatiles were removed under vacuum, and the waxy residue was washed with *n*-hexane (3 × 10 mL). The resulting white solid was dried *in vacuo*, affording the crude product as a white powder, which was partially soluble in benzene-d_6. Yield = 61.5 mg (58%). M.p.: 256-259 °C (melts to a pale-yellow oil). ^{1}H NMR (benzene-d_6, 400 MHz, 298 K): δ = 1.16 (d, J_{HH} = 6.8 Hz, 12H, *i*Pr-CH_3), 1.21 (d, J_{HH} = 6.2 Hz, 12H, *i*Pr-CH_3), 1.24 (d, J_{HH} = 6.4 Hz, 12H, *i*Pr-CH_3), 4.01 (sept, J_{HH} = 7.2 Hz, 2H, *i*Pr-C*H*), 4.08 (sept, J_{HH} = 7.2 Hz, 4H, *i*Pr-C*H*), 5.03 (s, N*H*), 6.62 (m, 2H, J_{HH} = 7.3 Hz, Ar-C*H*), 6.85 (m, 1H, Ar-C*H*), 6.96 (m, 7H, Ar-C*H*), 7.10 (m, 2H, Ar-C*H*), 8.04 (m, 2H, Ar-C*H*). ^{31}P{^{1}H} NMR (benzene-d_6, 121 MHz, 298 K): δ = -15.5 ppm (s). ^{13}C{^{1}H} NMR (benzene-d_6, 125.7 MHz, 298 K): δ = 23.9 (*i*Pr-*C*H_3), 24.5 (*i*Pr-*C*H_3), 24.6 (*i*Pr-*C*H_3), 27.8 (*i*Pr-*C*H), 28.1 (*i*Pr-*C*H), 116.6 (d, J_{CP} = 2.7 Hz, Ar-*C*H), 122.7 (d, J_{CP} = 1.4 Hz, Ar-*C*H), 122.9 (Ar-*C*H), 123.0 (Ar-*C*H), 126.2 (d, J_{CP} = 13.0 Hz, Ar-*C*H), 128.2 (Ar-*C*H), 128.3 (Ar-*C*H), 133.2 (d, J_{CP} = 6.5 Hz, Ar-*C*), 139.1 (d, J_{CP} = 158.0 Hz, Ph-*ipso*-*C*), 139.1 (d, J_{CP} = 3.5 Hz, Ar-*C*), 142.3 (d, J_{CP} = 2.0 Hz, Ar-*C*), 143.3 (d, J_{CP} = 8.3 Hz, Ar-*C*), 150.0 (d, J_{CP} = 4.1 Hz, Ar-*C*). IR (ATR), $\tilde{v}$/cm^{-1}: 2961w, 2922w, 2864w, 1582w, 1456s, 1360m, 1333s, 1290s, 1254s, 1107s, 756s, 696m.

Synthesis of TipSi 4/(TipSi)$_2$ 4$_2$

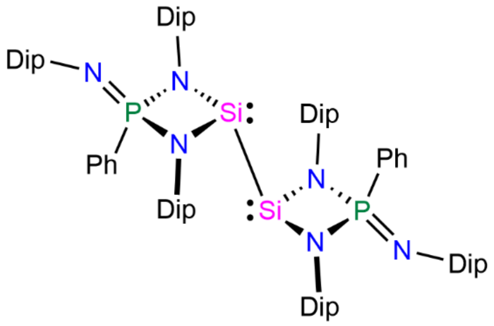

To a Schlenk flask charged with a mixture of [$TipLi_2$] **3** (100 mg, 0.155 mmol, 1.05 equiv.) and (SIDip)$SiBr_2$ (85.4 mg, 0.148 mmol, 1 equiv.) was added benzene (20 ml) at ca. 10 °C. The resulting orange solution was warmed to room temperature and stirred for two hours before all the volatiles were removed under vacuum. The yellow foamy residue was extracted with *n*-hexane (10 ml), and the solution was stored at -40 °C to yield a crop of yellow crystalline solid, which was analysed by

multinuclear NMR spectroscopies to be TipSi **4** (20 mg). The mother liquor was pumped down under vacuum, and the yellow residue was analysed to be a mixture of **4**, $TipH_2$ **2** and SIDip, from which pure TipSi **4** could not be separated off. Yield = 20 mg (20%). ^{1}H NMR (400.1 MHz, benzene-d_6, 339 K): δ = 0.72 (d, J_{HH} = 6.8 Hz, 12H, P=NDip-*i*Pr-C*H*3), 1.27 (d, J_{HH} = 6.8 Hz, 24H, P-NDip-*i*Pr-C*H*3), 2.89 (sept, J_{HH} = 6.9 Hz, 2H, P=NDip-*i*Pr-C*H*), 3.77 (s, br, 4H, P-NDip-*i*Pr-C*H*), 6.88 (m, 3H, Ph-C*H*), 6.95-7.06 (m, 3H, Ar-C*H*), 7.12 (s, 6H, Ar-C*H*), 7.15 (m, 2H, Ph-C*H*); ^{1}H NMR (499.9 MHz, cyclohexane-d_{12}, 338 K): δ = 0.45 (d, J_{HH} = 6.7 Hz, 12H, P=NDip-*i*Pr-C*H*3), 1.24 (d, J_{HH} = 6.5 Hz, 24H, P-NDip-*i*Pr-C*H*3), 2.65 (sept, J_{HH} = 6.7 Hz, 2H, P=NDip-*i*Pr-C*H*), 3.67 (s, br, 4H, P-NDip-*i*Pr-C*H*), 6.69 (m, 3H, Ph-C*H*), 7.10 (s, Ar-C*H*, 6H), 7.13-7.26 (m, 3H, Ar-C*H*), 7.66 (m, 2H, Ph-C*H*); ^{13}C{^{1}H} NMR (101.1 MHz, benzene-d_6, 295 K): δ = 22.5 (s, P=NDip-*i*Pr-*C*H3), 24.0 (s, P-NDip-*i*Pr-*C*H3), 27.5 (s, P=NDip-*i*Pr-*C*H), 29.3 (s, P-NDip-*i*Pr-*C*H), 120.4 (s, NDip-Ar-*C*H), 123.2 (s, P=NDip-Ar-*C*H), 123.9 (d, J_{CP} = 3.0 Hz, P-NDip-Ar-*C*H), 126.8 (d, J_{CP} = 3.0 Hz, P-NDip-Ar-*C*H), 127.2 (s, P-NDip-Ar-*C*H), 130.8 (d, J_{CP} = 3.0 Hz, Ph-*C*H), 133.0 (d, J_{CP} = 105.0 Hz, Ph-*ipso-C*), 133.9 (d, J_{CP} = 10.6 Hz, Ph-*C*H), 134.2 (d, J_{CP} = 4.6 Hz, P-NDip-Ar-*C*), 139.3 (d, J_{CP} = 6.0 Hz, P=NDip-Ar-*C*), 142.0 (P=NDip-Ar-*ipso-C*), 147.1 (d, J_{CP} = 4.1 Hz, P-NDip-Ar-*ipso-C*); ^{31}P{^{1}H} (162.1 MHz, benzene-d_6, 294 K): δ = -10.5 ppm (s); ^{31}P{^{1}H} (162.1 MHz, benzene-d_6, 338 K): δ = -10.5 ppm (s); ^{31}P{^{1}H} (202.4 MHz, cyclohexane-d_{12}, 300 K): δ = -9.45 ppm (s); ^{31}P{^{1}H} (202.4 MHz, cyclohexane-d_{12}, 338 K): δ = -10.5 ppm (s); ^{31}P{^{1}H} (202.4 MHz, *n*-hexane/cyclohexane-d_{12} (ca. 10:1), 243 K: note, at this temperature, the compound had mainly precipitated, but the ^{31}P NMR resonance could be recorded): δ = -11.2 ppm (s); ^{31}P{^{1}H} (202.4 MHz, *n*-hexane/cyclohexane-d_{12} (ca. 10:1), 273 K): δ = -10.4 ppm (s); ^{29}Si{^{1}H} (79.5 MHz, benzene-d_6, 338 K): δ = 117.1 ppm (s); ^{29}Si{^{1}H} (99.3 MHz, cyclohexane-d_{12}, 300 K): δ = 117.8 ppm (s); ^{29}Si{^{1}H} (99.3 MHz, cyclohexane-d_{12}, 338 K): δ = 117.8 ppm (s); ^{29}Si{^{1}H} (99.3 MHz, *n*-hexane/cyclohexane-d_{12} (ca. 10:1), 243 K): no signal (at this temperature, the compound had precipitated); ^{29}Si{^{1}H} (99.3 MHz, *n*-hexane/cyclohexane-d_{12} (ca. 10:1), 273 K): δ = 115.9 ppm (s).

Notes: Using (IDip)$SiCl_2$ in the reaction with [$TipLi_2$] **3** also formed some TipSi **4** *in-situ*, together with by-products including some $TipH_2$ **2**. However, this also repeatedly afforded colourless crystals of [(TipH)Li(IDip)] (δ_{31P} = -7.0 ppm) which was structurally characterised, see the X-ray crystallography section.

For alternative access to a precursor to TipSi **4**, we studied the reactions of [$TipLi_2$] **3** with SiX_4 (X = Cl, Br), which afforded intractable product mixtures under various conditions. In one instance, a crystal of [(TipH)Li{N(Dip)PN(Ar')Li}]·2 C_6H_{14}, Ar' = 2-*i*Pr-6(Me_2PhC)C_6H_3 (see the X-ray crystallography section) was afforded, highlighting that the combination of highly basic [$TipLi_2$] **3** and Lewis-acidic SiX_4 can lead to reactions that modify and decompose the ligand framework.

Synthesis of TipGe 5/(TipGe)$_2$ 5$_2$, and (TipH)Ge(NHDip), (TipH)GeCl, and (TipHGe)$_2$O

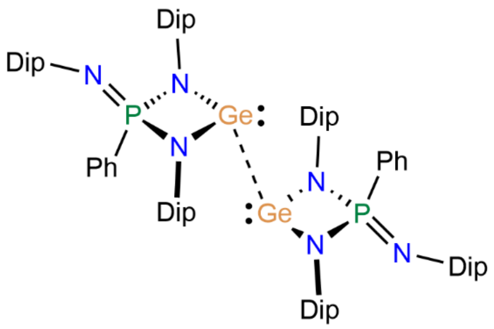

To a stirring solution of germanium(II) chloride-dioxane adduct, $GeCl_2$·dioxane (71.6 mg, 0.309 mmol, 1 equiv.) in THF (20 mL) was added a solution of [$TipLi_2$] **3** (200 mg, 0.309 mmol, 1 equiv.) in THF (20 mL) at -80 °C. The mixture was stirred at room temperature for 18 h before all the volatiles were removed under vacuum, and the residue was extracted with *n*-pentane (25 mL). Yellow to orange crystals of TipGe **5** suitable for single crystal X-ray diffraction were obtained after storing the solution at -25 °C for two days. Yield: 65 mg (27%). Notes: *in-situ* preparations of TipGe **5**, e.g. generated in deuterated benzene, provided higher yields, see the NMR section. In one instance, a crystal of TipGe·(TipH)Ge(NHDip)·C_5H_{14}, **5**·(TipH)Ge(NHDip)·C_5H_{14}, was obtained and crystallographically characterised (see the X-ray crystallography section), which was likely formed from the reaction of TipGe **5** with $DipNH_2$ as an impurity in the ligand. Later crops in the synthesis of TipGe **5** contained (TipH)GeCl on occasion, which was characterised by X-ray diffraction and NMR spectroscopy, see below. Traces of moisture and TipGe **5** afforded (TipHGe)$_2$O, which was structurally characterised, please see the X-ray diffraction section. M.p.: 192 °C (melts). ^{1}H-NMR (benzene-*d*$_6$, 300.1 MHz, 298 K): δ = 0.74 (d, J_{HH} = 6.9 Hz, 12H, *i*Pr-C*H*$_3$), 0.91 (d, J_{HH} = 6.9 Hz, 12 H, *i*Pr-C*H*$_3$), 1.25 (d, J_{HH} = 6.9 Hz, 12H, *i*Pr-C*H*$_3$), 2.91 (sept, J_{HH} = 6.9 Hz, 2 H, *i*Pr-C*H*), 3.70 (br sept, J_{HH} = 6.9 Hz, 4H, *i*Pr-C*H*), 7.04 (m, 12H, Ar-C*H*), 7.67 (m, 2H, Ar-C*H*). ^{31}P{^{1}H} NMR (benzene-*d*$_6$, 121.0 MHz, 298 K): δ = -8.6 ppm (s). ^{13}C{^{1}H} NMR (101.0 MHz, benzene-*d*$_6$, 300 K): δ = 24.7 (*i*Pr-*C*H$_3$), 27.9 (*i*Pr-*C*H$_3$), 29.5 (*i*Pr-*C*H), 120.4 (Ar-*C*), 123.6 (Ar-*C*), 124.2 (d, J_{CP} = 2.4 Hz, Ar-*C*H), 126.8 (d, J_{CP} = 2.8 Hz, Ar-*C*H), 130.6 (d, J_{CP} = 2.8 Hz, Ar-*C*H), 134.1 (d, J_{CP} = 10.2 Hz, Ar-*C*H), 134.4 (Ar-*C*), 134.9 (d, J_{CP} = 109.6 Hz, Ar-*C*), 137.2 (d, J_{CP} = 5.3 Hz, Ar-*C*H), 139.8 (d, J_{CP} = 5.6 Hz, Ar-*C*H), 143.0 (d, J_{CP} = 4.0 Hz, Ar-*C*), 147.2 (Ar-*C*). IR (nujol), $\tilde{v}$/cm^{-1}: 3058w, 2960m, 2924m, 2865w, 1590w, 1433m, 1382m, 1360m, 1254s, 1191m, 1103s, 794s, 748s. EI-MS (solid state, 70 eV) m/z (%): 707.5 (22, [TipGe]$^+$), 664.5 (12, [TipGe-*i*Pr]$^+$), 592.5 (9, [$TipH_2$-*i*Pr]$^+$). Elemental analysis: calculated for $C_{42}H_{56}GeN_3P$: C 71.40; H 7.99; N 5.95%; found: C 71.32; H 8.10; N 5.90%.

NMR data for [(TipH)GeCl]

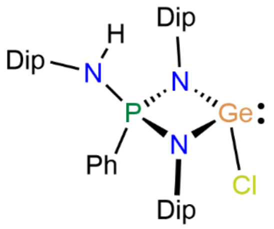

^{1}H-NMR (499.9 MHz, benzene-d_6, 298 K): δ = 0.38 (d, J_{HH} = 6.7 Hz, 6H, *i*Pr-C*H*$_3$), 0.70 (d, J_{HH} = 6.7 Hz, 6H, *i*Pr-C*H*$_3$), 0.82 (d, J_{HH} = 6.9 Hz, 6H, *i*Pr-C*H*$_3$), 1.26 (d, J_{HH} = 6.7 Hz, 6H, *i*Pr-C*H*$_3$), 1.38 (d, J_{HH} = 7.0 Hz, 6H, *i*Pr-C*H*$_3$), 1.40 (d, J_{HH} = 6.7 Hz, 6H, *i*Pr-C*H*$_3$), 2.62 (sept, J_{HH} = 6.7 Hz, 2H, *i*Pr-C*H*), 4.10 (sept, J_{HH} = 7.1 Hz, 2H, *i*Pr-C*H*), 4.26 (sept, J_{HH} = 6.6 Hz, 2H, *i*Pr-C*H*), 4.72 (s, NH), 6.75-6.93 (m, 6H, Ar-C*H*), 7.08-7.11 (m, 6H, Ar-C*H*), 8.16 (m, 2H, Ar-C*H*). ^{31}P{^{1}H} NMR (202.4 MHz, benzene-d_6, 121.0 MHz, 298 K): δ = 28.1 ppm (s). ^{13}C{^{1}H} NMR (125.7 MHz, benzene-d_6, 300 K): δ = 22.7 (*i*Pr-*C*H$_3$), 22.9 (*i*Pr-*C*H$_3$), 23.1 (*i*Pr-*C*H$_3$), 24.8 (*i*Pr-*C*H$_3$), 27.7 (*i*Pr-*C*H$_3$), 28.0 (*i*Pr-*C*H), 28.3 (*i*Pr-*C*H), 28.6 (*i*Pr-*C*H), 30.0 (*i*Pr-*C*H), 123.7 (Ar-*C*H), 124.7 (Ar-*C*H), 124.8 (Ar-*C*H), 125.9 (Ar-*C*H), 127.4 (Ar-*C*H), 127.8 (Ar-*C*H), 129.9 (d, J_{CP} = 129.5 Hz, Ph-*ipso*-*C*), 132.1 (d, J_{CP} = 2.9 Hz, Ar-*C*H), 133.8 (d, J_{CP} = 11.1 Hz, Ar-*C*H), 136.1 (d, J_{CP} = 3.1 Hz, Ar-*C*), 144.4 (d, J_{CP} = 5.6 Hz, Ar-*C*), 146.7 (d, J_{CP} = 5.4 Hz, Ar-*C*), 149.1 (d, J_{CP} = 2.5 Hz, Ar-*C*).

Synthesis of TipSn 6

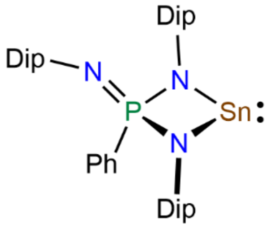

To a stirring solution/slurry of tin(II) bromide ($SnBr_2$) (64.4 mg, 0.232 mmol, 1 equiv.) in Et_2O (20 mL) at -30 °C, was added a solution of [$TipLi_2$] **3** (150 mg, 0.232 mmol, 1 equiv.) in Et_2O (20 mL). The reaction mixture was slowly warmed to room temperature and stirred for 18 h, during which the formation of a white precipitate was observed. The yellow solution was then filtered, and all volatiles from the filtrate were removed under vacuum. The foamy yellow residue was extracted with toluene (30 mL), concentrated and stored at -25 °C for two days to yield crystals of **6** suitable for single crystal X-ray diffraction. Yield: 105 mg (60%). M.p.: 240-242 °C (melts). ^{1}H-NMR (benzene-d_6, 499.9 MHz, 343 K): δ = 0.74 (d, J_{HH} = 6.6 MHz, 12H, *i*Pr-C*H*$_3$), 0.89 (br d, J_{HH} = 6.6 MHz, 12H, *i*Pr-C*H*$_3$), 1.25 (d, J_{HH} = 6.6 MHz, 12H, *i*Pr-C*H*$_3$), 2.91 (sept, J_{HH} = 6.6 MHz, 2H, *i*Pr-C*H*), 3.74 (br sept, 4H, *i*Pr-C*H*), 6.93 (m, 4H, Ar-C*H*), 7.06 (m, 4H, Ar-C*H*), 7.14 (m, 4H, Ar-C*H*), 7.61 (m, 2H, Ar-C*H*); ^{13}C{^{1}H} NMR (benzene-d_6, 125.7 MHz, 343 K): δ = 24.5 (*i*Pr-*C*H$_3$), 27.2 (*i*Pr-*C*H$_3$), 28.6 (*i*Pr-*C*H), 119.3 (Ar-*C*H), 123.1 (Ar-*C*H), 123.5 (d, J_{CP} = 2.7 Hz, Ar-*C*H), 125.6 (d, J_{CP} = 3.1 Hz, Ar-*C*H),

127.1 (d, J_{CP} = 3.0 Hz, Ar-*C*H), 129.2 (d, J_{CP} = 3.0 Hz, Ar-*C*H), 133.2 (d, J_{CP} = 9.5 Hz, Ar-*C*H), 138.1 (d, J_{CP} = 114.3 Hz, Ar-*C*), 139.0 (d, J_{CP} = 5.0 Hz, Ar-*C*), 139.7 (d, J_{CP} = 5.6 Hz, Ar-*C*), 143.7 (d, J_{CP} = 4.5 Hz, Ar-*C*), 146.6 (d, J_{CP} = 3.6 Hz, Ar-*C*). $^{31}P\{^1H\}$ NMR (benzene-d_6, 202.1 MHz, 343 K): δ = -7.7 ppm (s, with possible combined $^{117/119}$Sn satellites, $^2J_{117/119Sn\text{-}31P} \approx$ 46.5 Hz). $^{119}Sn\{^1H\}$ NMR (benzene-d_6, 149.3 MHz, 298 K): δ = 248.3 ppm (s). IR (nujol), ν~/cm^{-1}: 3060w, 2959m, 1588w, 1433m, 1361m, 1334m, 1290w, 1258s, 1186w, 1090s, 1015s, 922m, 907m, 790s, 752s. EI-MS (solid state, 70 eV) m/z (%): 635.5 (2, $[TipH_2]^+$), 592.5 (7, [TipH$_2$-*i*Pr]$^+$), 459.3 (90, [TipH$_2$-DipNH]$^+$), 177.1 (100, $[DipNH_3]^+$).

THF addition to TipSn **6**.

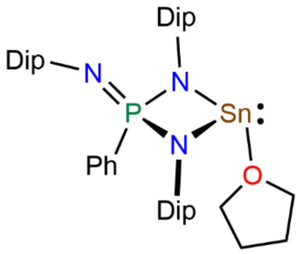

To a J. Young NMR tube charged with a benzene-d_6 solution (0.5 mL) of TipSn **6** (50.0 mg, 66.4 µmol, 1 equiv.) was added THF (16.2 µL, 198 µmol, ca. 3 equiv.) at ambient temperature. The clean conversion of TipSn **6** to a new phosphorus-containing species was observed via $^{31}P\{^1H\}$ NMR spectroscopy. The resulting product was characterised by multinuclear NMR spectroscopy and believed to be TipSn(THF), **6**(THF). Small yellow crystals were obtained from the above benzene-d_6 solution at -4 °C, but these redissolved too quickly and easily for characterisation by X-ray diffraction. ^{1}H-NMR (benzene-d_6, 499.9 MHz, 298 K): δ = 0.61 (br d, 6H, *i*Pr-CH_3), 0.82 (d, J = 6.6 Hz, 12H, *i*Pr-CH_3), 1.17 (br d, 12H, *i*Pr-CH_3), 1.53 (br d, 6H, *i*Pr-CH_3), 2.97 (sept, J_{HH} = 6.7 Hz, 2H, *i*Pr-C*H*), 3.45 (br sept, 2H, *i*Pr-C*H*), 4.41 (br sept, 2H, *i*Pr-C*H*), 6.91 (m, 3H, Ph-C*H*), 6.96 (m, 1H, Ar-C*H*), 7.09 (m, 2H, Ar-C*H*), 7.13 (m, 3H, Ar-C*H*), 7.19 (m, 2H, Ar-C*H*), 7.90 (m, 2H, Ph-C*H*). $^{13}C\{^1H\}$ NMR (benzene-d_6, 125.8 MHz, 298 K): δ = 25.0 (*i*Pr-*C*H$_3$), 25.4 (*i*Pr-*C*H$_3$), 27.2 (*i*Pr-*C*H), 28.8 (br, *i*Pr-*C*H), 118.9 (Ar-*C*H), 123.3 (Ar-*C*H), 123.8 (d, J_{CP} = 2.4 Hz, Ar-*C*H), 125.2 (d, J_{CP} = 2.7 Hz, Ar-*C*H), 126.9 (d, J_{CP} = 12.3 Hz, Ar-*C*H), 128.0 (Ar-*C*H), 129.2 (Ar-*C*H), 133.2 (d, J_{CP} = 9.7 Hz, Ar-*C*H), 139.7 (d, J_{CP} = 5.6 Hz, Ar-*C*), 139.9 (d, J_{CP} = 115 Hz, Ar-*C*), 144.4 (d, J_{CP} = 5.2 Hz, Ar-*C*), 147.0 (Ar-*C*). $^{31}P\{^1H\}$ NMR (benzene-d_6, 202.4 MHz, 298 K): δ = -6.5 (s, with possible combined $^{117/119}$Sn satellites, $^2J_{117/119Sn\text{-}31P} \approx$ 24.8 Hz) $^{119}Sn\{^1H\}$ NMR (benzene-d_6, 149.3 MHz, 298 K): δ = 113.9 ppm (s).

Synthesis of TipPb 7

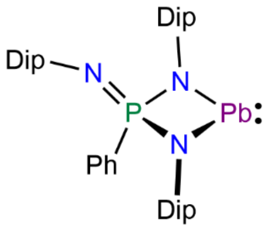

To a stirring solution/slurry of lead(II) chloride ($PbCl_2$) (110 mg, 0.396 mmol, 1.3 equiv.) in Et_2O (20 mL) was added a solution of [$TipLi_2$] **3** (200 mg, 0.309 mmol, 1.0 equiv.) in Et_2O (20 mL) at -80 °C. The reaction mixture was slowly warmed to room temperature and stirred for 18 hours, during which the formation of a white precipitate was observed. The orange solution was then filtered, and all the volatiles were removed from the filtrate under vacuum. The residue was extracted with toluene (30 mL), and the solution was stored at -25 °C to yield orange crystals for single crystal X-ray diffraction analysis. Yield: 50 mg (19%). M.p.: decomposes above 210 °C. ^{1}H-NMR (benzene-d_6, 300.0 MHz, 338 K): δ = 0.76 (d, J_{HH} = 6.6 Hz, 12H, *i*Pr-CH_3), 0.90 (d, J_{HH} = 6.6 Hz, 12H, *i*Pr-CH_3), 1.26 (d, J_{HH} = 6.6 Hz, 12H, *i*Pr-CH_3), 2.95 (sept, J_{HH} = 6.6 Hz, 2H, *i*Pr-C*H*), ca. 3.9 (very br, 4H, *i*Pr-C*H*), 6.95 (m, 7H, Ar-C*H*), 7.20 (m, 5H, Ar-C*H*), 7.68 (m, 2H, Ar-C*H*). ^{13}C{^{1}H} NMR data (benzene-d_6, 100.1 MHz, 300 K): δ = 23.6 (*i*Pr-*C*H_3), 24.8 (*i*Pr-*C*H_3), 27.1 (*i*Pr-*C*H), 28.5 (*i*Pr-*C*H), 118.8 (Ar-*C*H), 122.6 (Ar-*C*H), 123.3 (Ar-*C*H), 126.2 (d, J_{CP} = 2 Hz, Ar-*C*H), 128.7 (d, J_{CP} = 9.0 Hz, Ar-*C*H), 132.8 (d, J_{CP} = 9.0 Hz, Ar-*C*H), 138.2 (Ar-*C*), 140.2 (d, J_{CP} = 6.8 Hz, Ar-*C*), 144.3 (d, J_{CP} = 118.0 Hz, Ar-*C*), 148.3 (d, J_{CP} = 4.7 Hz, Ar-*C*). ^{31}P{^{1}H} NMR (benzene-d_6, 121.1 MHz, 338 K): δ = 0.44 (s, with possible satellites, $^{2}J_{207Pb-31P}$ ≈ 90.8 Hz). IR (nujol), $\tilde{\nu}$/cm^{-1}: 3053w, 2958m, 1588w, 1458s, 1428s, 1301m, 1241m, 1194s, 1139w, 1104m, 1073m, 953m, 929s, 814m, 781s, 755s.

Reactivity of TipSi 4

Synthesis of TipSi(DMAP) 9

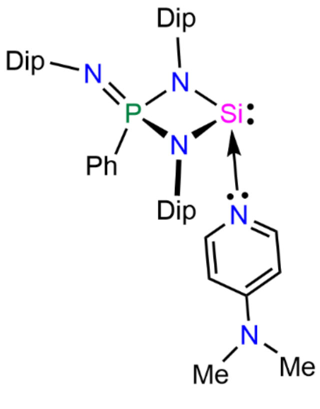

To a J. Young NMR tube charged with a solution of TipSi **4** (10.0 mg, 15.2 µmol, 1 equiv.) in benzene-d_6 (0.5 mL) was added 4-(dimethylamino)pyridine (DMAP, 1.85 mg, 15.2 µmol, 1 equiv.)

at ambient temperature to immediately give a bright yellow solution. The full conversion of TipSi **4** to TipSi(DMAP) **9** was observed via $^{31}P\{^1H\}$ NMR spectroscopy. Yellow crystals suitable for single crystal X-ray diffraction analysis were obtained by layering *n*-hexane (0.2 mL) onto the above concentrated benzene-d_6 solution (0.2 mL) of **9**. ^1H-NMR (benzene-d_6, 499.9 MHz, 298 K): δ = 0.79 (br d, 6H, *i*Pr-CH_3), 0.92 (br d, 12H, *i*Pr-CH_3), 1.24 (br d, 6H, *i*Pr-CH_3), 1.75 (br d, 12H, *i*Pr-CH_3), 1.88 (s, 6H, DMAP-N(CH_3)$_2$), 3.12 (sept, J_{HH} = 6.7 Hz, 2H, *i*Pr-C*H*), 3.65 (br sept, 2H, *i*Pr-C*H*), 5.20 (br sept, 2H, *i*Pr-C*H*), 5.85 (d, J_{HH} = 7.1 Hz, DMAP-C*H*), 6.88 (m, 3H, Ph-C*H*), 6.93 (m, 1H, Ar-C*H*), 7.11 (m, 2H, Ar-C*H*), 7.18 (m, 2H, Ar-C*H*), 7.26 (m, 2H, Ar-C*H*), 7.44 (br m, 2H, Ar-C*H*), 7.75 (m, 2H, Ph-C*H*), 8.32 (d, J_{HH} = 7.1 Hz, DMAP-C*H*). $^{13}C\{^1H\}$ NMR (benzene-d_6, 125.8 MHz, 298 K): δ = 23.7 (*i*Pr-*C*H$_3$), 24.6 (*i*Pr-*C*H$_3$), 25.4 (*i*Pr-*C*H$_3$), 27.1(*i*Pr-*C*H), 28.4 (*i*Pr-*C*H), 30.6 (*i*Pr-*C*H), 37.8 (DMAP-*C*H$_3$), 106.0 (DMAP-*C*H), 118.8 (Ar-*C*H), 123.1 (Ar-*C*H), 123.8 (Ar-*C*H), 125.5 (Ar-*C*H), 125.4 (Ar-*C*H), 126.9 (d, J_{CP} = 12.3 Hz, Ar-*C*H), 129.5 (Ar-*C*H), 132.3 (d, J_{CP} = 10.5 Hz, Ar-*C*H), 138.2 (d, J_{CP} = 4.4 Hz, Ar-*C*), 138.8 (d, J_{CP} = 106.0 Hz, Ar-*C*), 140.0 (d, J_{CP} = 6.0 Hz, Ar-*C*), 144.2 (d, J_{CP} = 4.2 Hz, Ar-*C*), 145.8 (DMAP-*C*H), 149.9 (Ar-*C*), 154.7 (Ar-*C*). $^{31}P\{^1H\}$ NMR (benzene-d_6, 162.0 MHz, 298 K): δ = -4.3 ppm.

Synthesis of TipSi(CPh)$_2$ 10

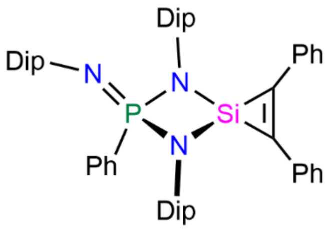

To a J. Young NMR tube charged with a solution of TipSi **4** (10.0 mg, 15.2 µmol, 1.0 equiv.) in benzene-d_6 (0.5 mL), was added diphenylacetylene (2.71 mg, 15.2 µmol, 1.0 equiv.) to yield a pale-yellow solution immediately. The full conversion of TipSi **4** to TipSi(CPh)$_2$ **10** was observed via $^{31}P\{^1H\}$ NMR spectroscopy within one hour. Estimated *in-situ* NMR yield: 78% (equates to 9.9 mg). Colourless crystals of TipSi(CPh)$_2$ **10** suitable for single crystal X-ray diffraction analysis were obtained from a solvent mixture of benzene-d_6 and *n*-hexane at -4 °C overnight. ^1H-NMR (400.1 MHz, benzene-d_6, 298 K): δ = 0.64 (d, J_{HH} = 6.2 Hz, 6H, P-NDip-*i*Pr-CH_3), 0.90 (br, 6H, P=NDip-*i*Pr-CH_3), 1.05 (d, J_{HH} = 7.2 Hz, 6H, P-NDip-*i*Pr-CH_3), 1.24 (br, 6H, P=NDip-*i*Pr-CH_3), 1.39 (d, J_{HH} = 6.5 Hz, 6H, P-NDip-*i*Pr-CH_3), 1.62 (d, J_{HH} = 6.2 Hz, 6H, P-NDip-*i*Pr-CH_3), 3.02 (sept, J_{HH} = 5.5 Hz, 2H, P-NDip-*i*Pr-C*H*), 3.81 (sept, J_{HH} = 7.0 Hz, 2H, P=NDip-*i*Pr-C*H*), 4.83 (sept, J_{HH} = 6.5 Hz, 2H, P=NDip-*i*Pr-C*H*), 6.96 (m, Ar-C*H*), 7.11 (m, Ar-C*H*), 7.19 (m, Ar-C*H*), 7.52 (d, Ar-C*H*), 7.63 (d, Ar-C*H*), 8.23 (m, Ar-C*H*). $^{13}C\{^1H\}$ NMR (101.1 MHz, benzene-d_6, 295 K): δ = 22.2 (*i*Pr-*C*H$_3$), 23.3 (*i*Pr-*C*H$_3$), 24.3 (*i*Pr-*C*H$_3$), 24.1 (*i*Pr-*C*H$_3$), 25.8 (*i*Pr-*C*H$_3$), 27.0 (*i*Pr-*C*H$_3$), 27.4 (*i*Pr-*C*H), 28.8

(*i*Pr-*C*H), 30.3 (*i*Pr-*C*H), 31.6 (Ar-*C*), 89.8 (Ar-*C*), 120.5 (Ar-*C*H), 123.6 (Ar-*C*H), 123.9 (Ar-*C*H), 134.1 (d, J_{CP} = 10.2 Hz, Ph-*C*H), 135.6 (d, J_{CP} = 119.0 Hz, Ph-*C*), 139.7 (d, J_{CP} = 6.3 Hz, Ph-*CH*), 147.7 (Ar-*C*), 150.0 (Ar-*C*). ^{31}P{^{1}H} NMR (162.0 MHz, benzene-*d*$_6$, 294 K): δ = -22.6 ppm (s). IR (ATR), *v*~/cm^{-1}: 2961w, 2926w, 2866w, 1441s, 1381s, 1258s, 1200m, 1101s, 1007s, 932s, 795s.

Synthesis of TipSi{CH$_2$C(Me)}$_2$ 11

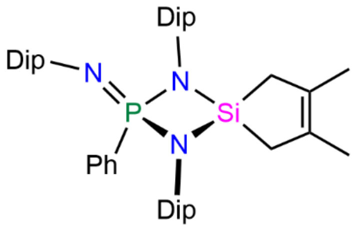

To a J. Young NMR tube charged with a solution of TipSi **4** (10.0 mg, 15.2 µmol, 1.0 equiv.) in benzene-*d*$_6$ (0.5 mL), was added 2,3-dimethyl-1,3-butadiene (ca. 3.47 µL, 30.4 µmol, 2.0 equiv.) at room temperature to yield a bright yellow solution once mixed. The full conversion of TipSi **4** to TipSi{CH$_2$C(Me)}$_2$ **11** was observed via ^{31}P{^{1}H} NMR spectroscopy within one hour. Pale-yellow crystals suitable for single crystal X-ray diffraction analysis were obtained from a solvent mixture of benzene-*d*$_6$ and *n*-hexane at -4 °C for 2 days. Estimated *in-situ* NMR yield: 72% (equates to 8.1 mg). ^{1}H-NMR (499.9 MHz, benzene-*d*$_6$, 298 K): δ = 0.54 (d, J_{HH} = 6.9 Hz, 6H, *i*Pr-C*H*$_3$), 1.13 (d, J_{HH} = 6.9 Hz, 12H, *i*Pr-C*H*$_3$), 1.28 (d, J_{HH} = 6.8 Hz, 12H, *i*Pr-C*H*$_3$), 1.58 (d, J_{HH} = 6.5 Hz, 6H, *i*Pr-C*H*$_3$), 1.40 (s, (C*H*$_3$)CC(CH$_3$)), 1.67 (s, (CH$_3$)CC(C*H*$_3$)), 1.82 (s, Si-C*H*$_2$), 2.19 (s, Si-C*H*$_2$), 2.87 (sept, J_{HH} = 6.7 Hz, 2H, *i*Pr-C*H*), 3.72 (sept, J_{HH} = 6.4 Hz, 2H, *i*Pr-C*H*), 4.35 (sept, J_{HH} = 6.9 Hz, 2H, *i*Pr-C*H*), 6.97-7.00 (m, 3H, Ar-C*H*), 7.12 (m, 6H, Ar-C*H*), 7.19 (m, 3H, Ar-C*H*), 7.93-7.98 (m, 2H, Ar-C*H*). ^{31}P{^{1}H} NMR (162.0 MHz, benzene-*d*$_6$, 295 K): δ = -18.5 ppm (s). Note: a high-quality ^{13}C NMR spectrum for compound **11** was not obtained owing to insufficient isolated product and impurities in the *in-situ* generated sample.

2 NMR Spectroscopy

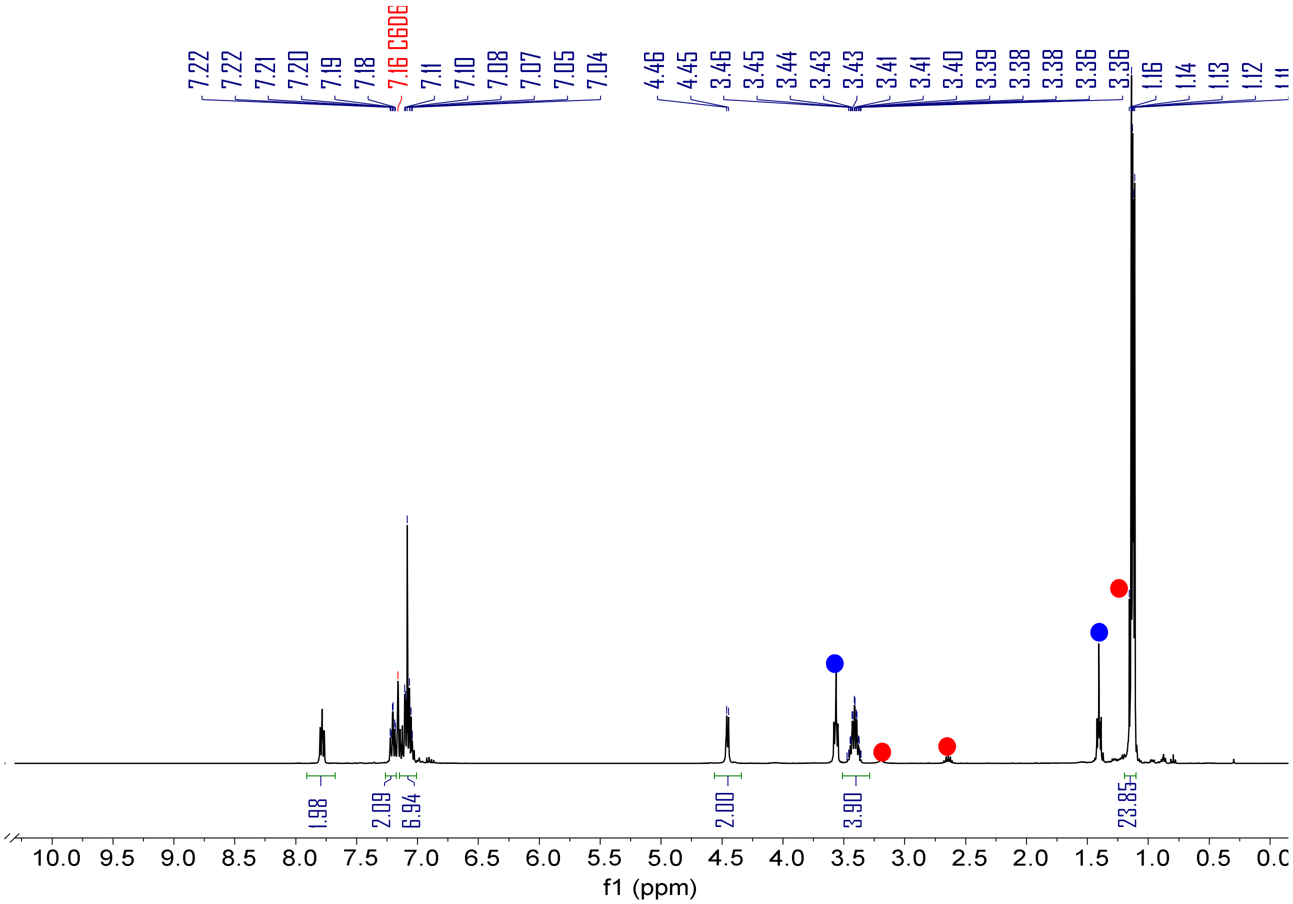

Figure S1. ¹H-NMR spectrum (400.1 MHz, C_6D_6, 294 K) of isolated PhP(NHDip)$_2$·THF **1**. The blue circles denote the chemical resonances of THF. The red circles denote the resonances of a trace amount of DipNH$_2$.

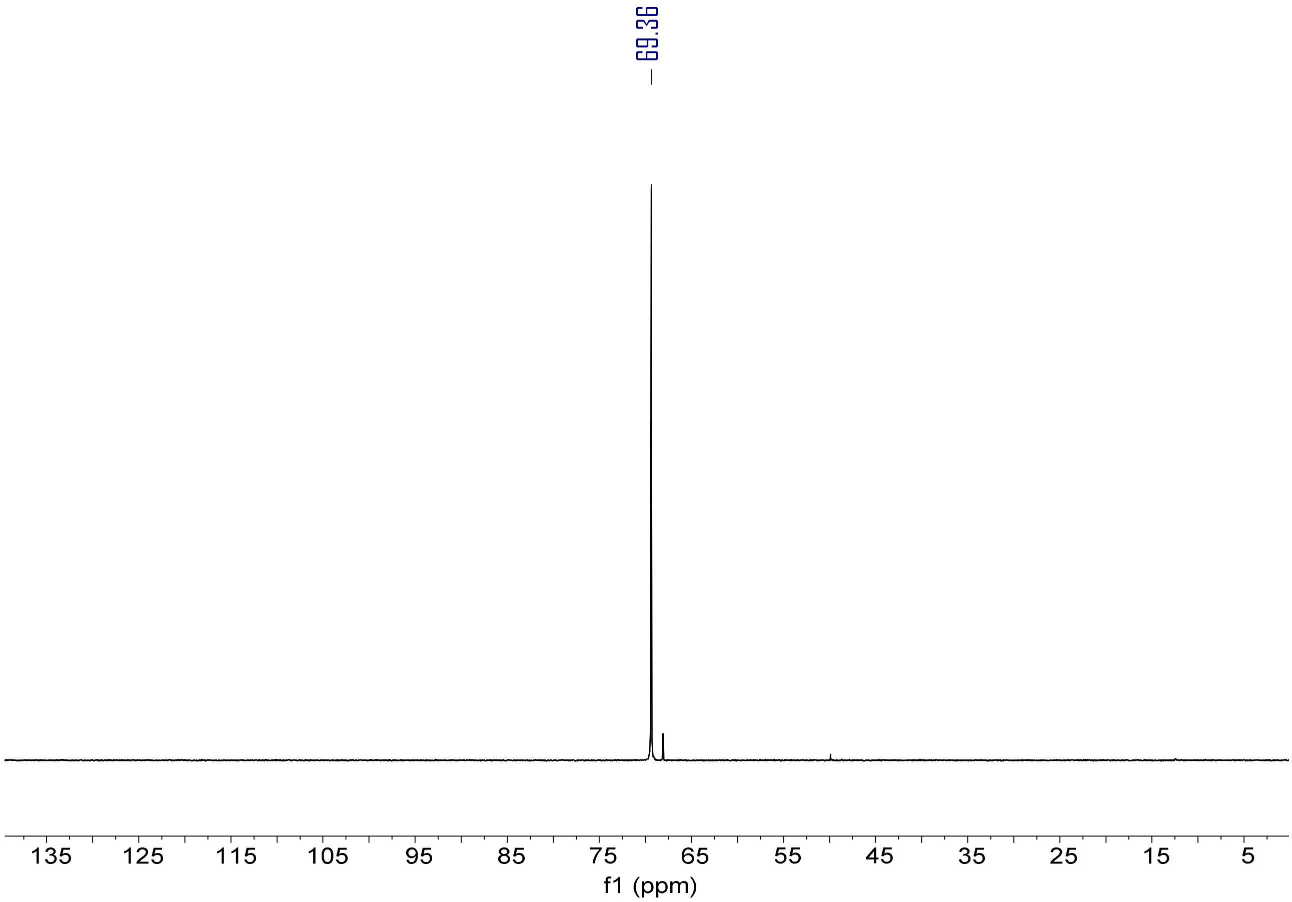

Figure S2. $^{31}P\{^{1}H\}$ NMR spectrum (162.9 MHz, C_6D_6, 295 K) of PhP(NHDip)$_2$·THF **1**.

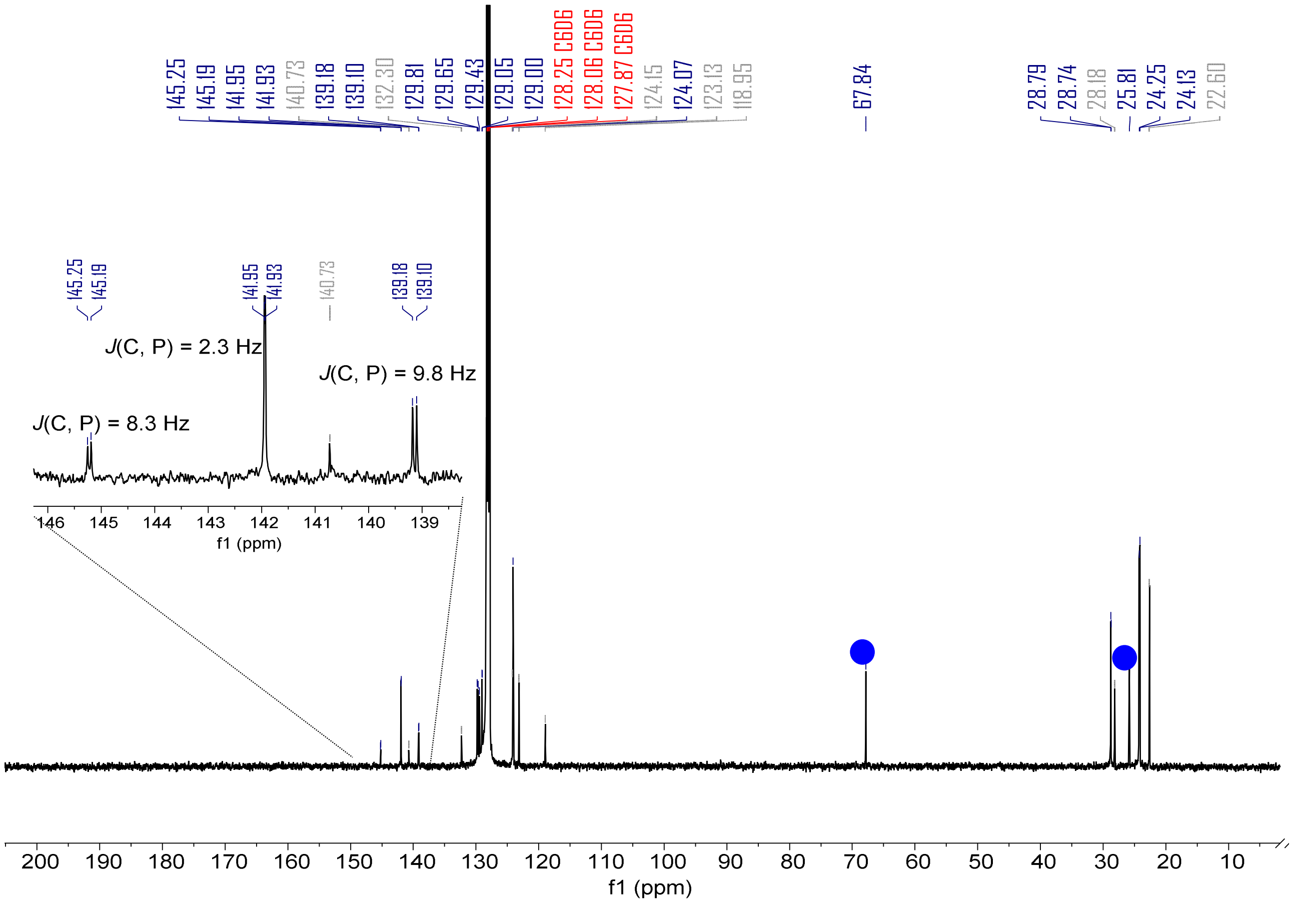

Figure S3. $^{13}C\{^{1}H\}$ NMR spectrum (125.7 MHz, C_6D_6, 298 K) of $PhP(NHDip)_2\cdot THF$ **1**. The blue circles denote the chemical resonances of THF. The grey-labelled peaks denote the chemical shift of impurities (e.g. $DipNH_2$). The zoomed region shows several representative doublets corresponding to C-P coupling.

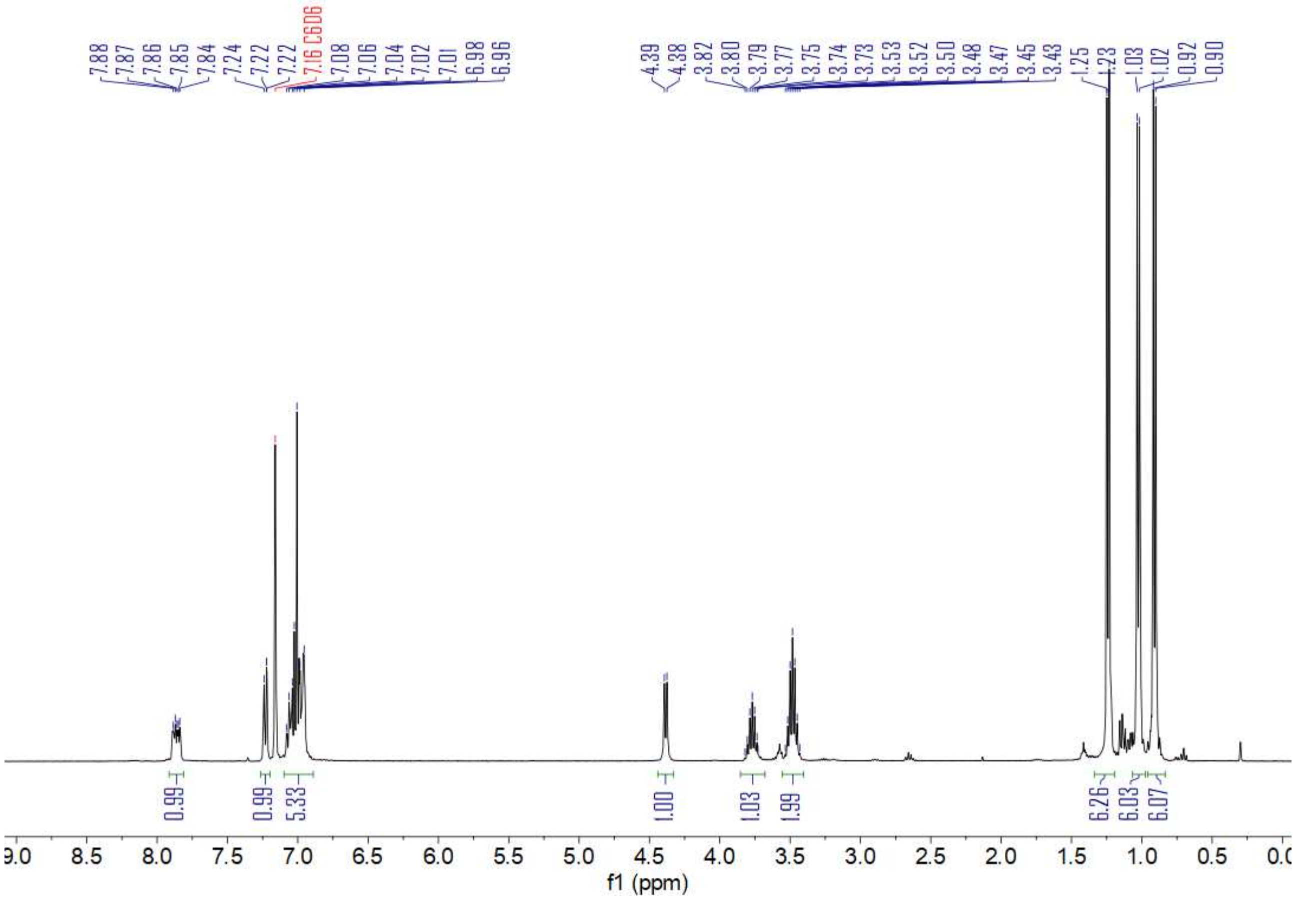

Figure S4. ^{1}H-NMR spectrum (400.1 MHz, C_6D_6, 298 K) of $TipH_2$ **2**.

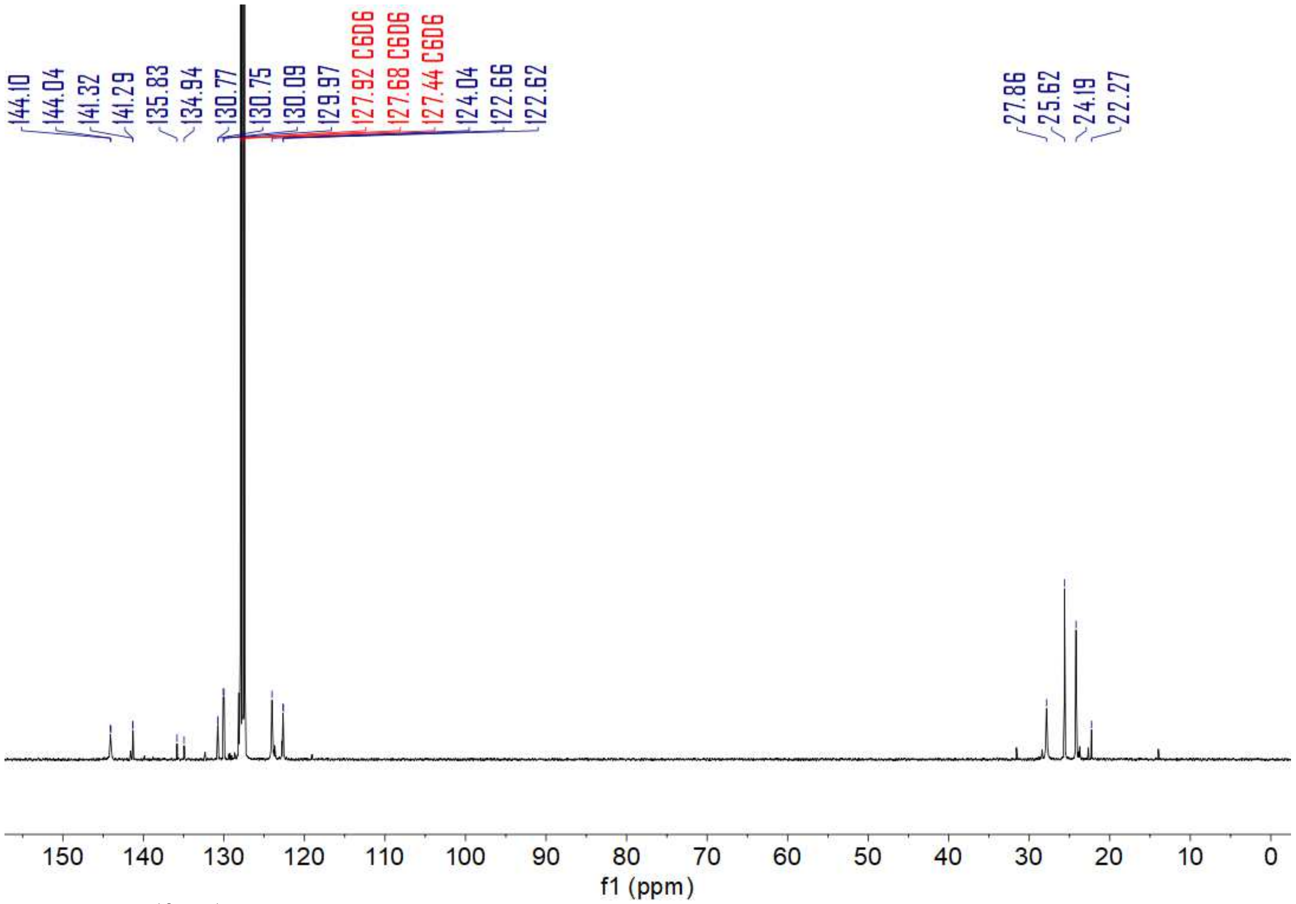

Figure S5. $^{13}C\{^{1}H\}$ NMR spectrum (100.6 MHz, C_6D_6, 300 K) of $TipH_2$ **2**.

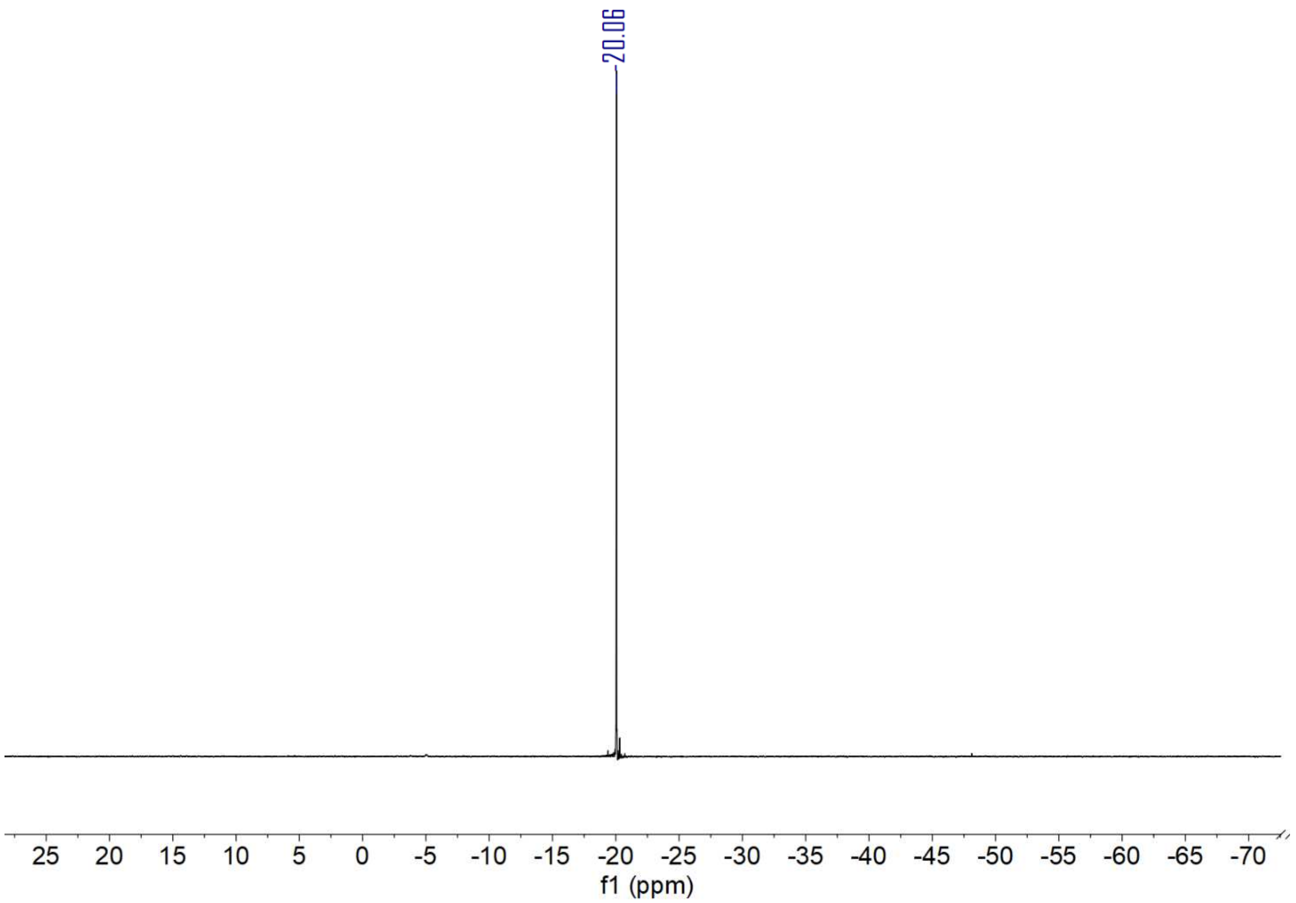

Figure S6. $^{31}P\{^{1}H\}$ NMR spectrum (121.5 MHz, C_6D_6, 298 K) of $TipH_2$ **2**.

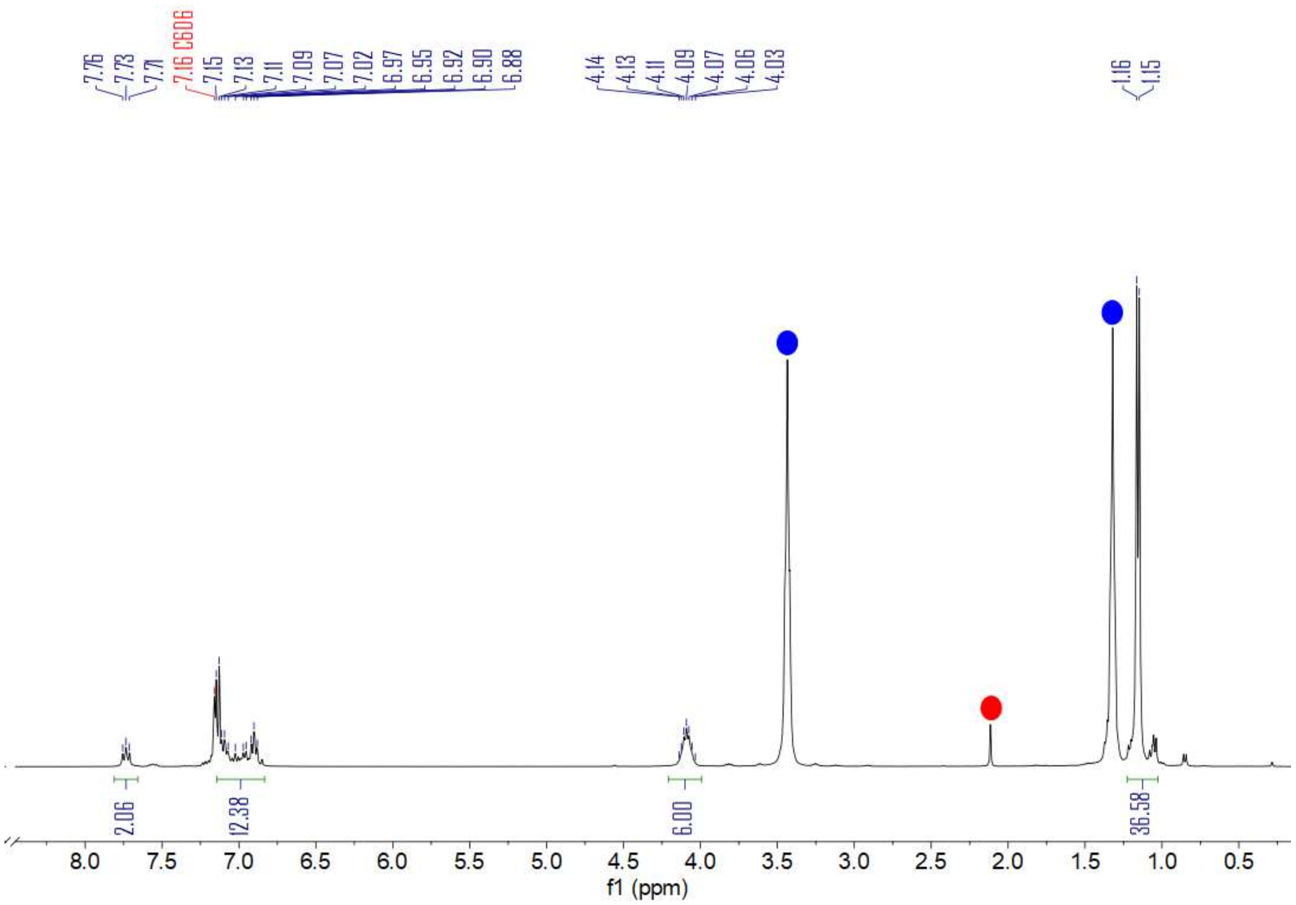

Figure S7. ^{1}H-NMR spectrum (400.1 MHz, C_6D_6, 298 K) of [$TipLi_2$] **3** (obtained after adding a drop of THF to the deuterated benzene solution of **3**). The blue circles denote the resonances of THF. The red circle denotes the resonance of residual toluene.

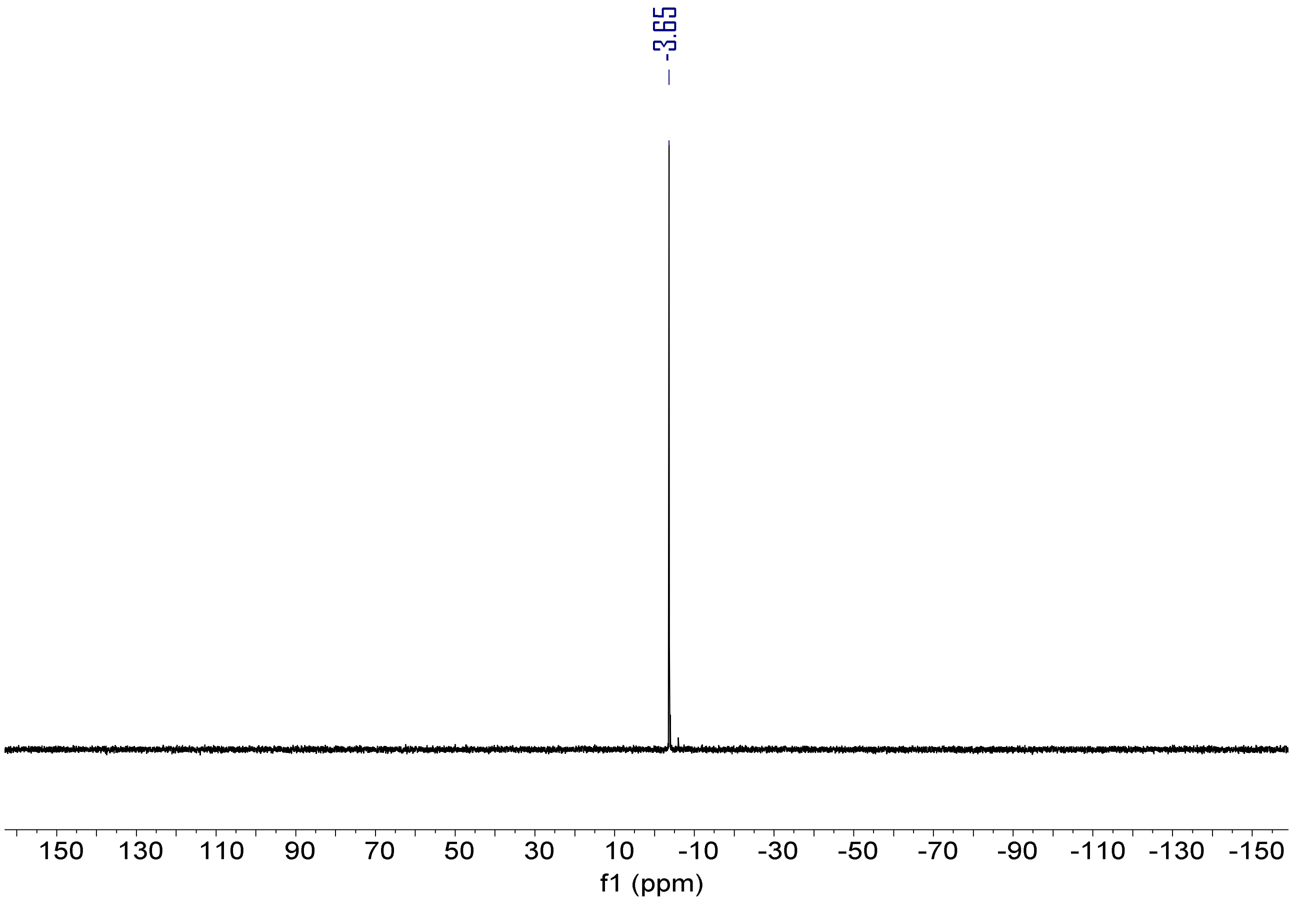

Figure S8. $^{31}P\{^{1}H\}$ NMR spectrum (202.4 MHz, C_6D_6, 298 K) of [$TipLi_2$] **3** (was obtained after adding a drop of THF to the deuterated benzene solution of **3**).

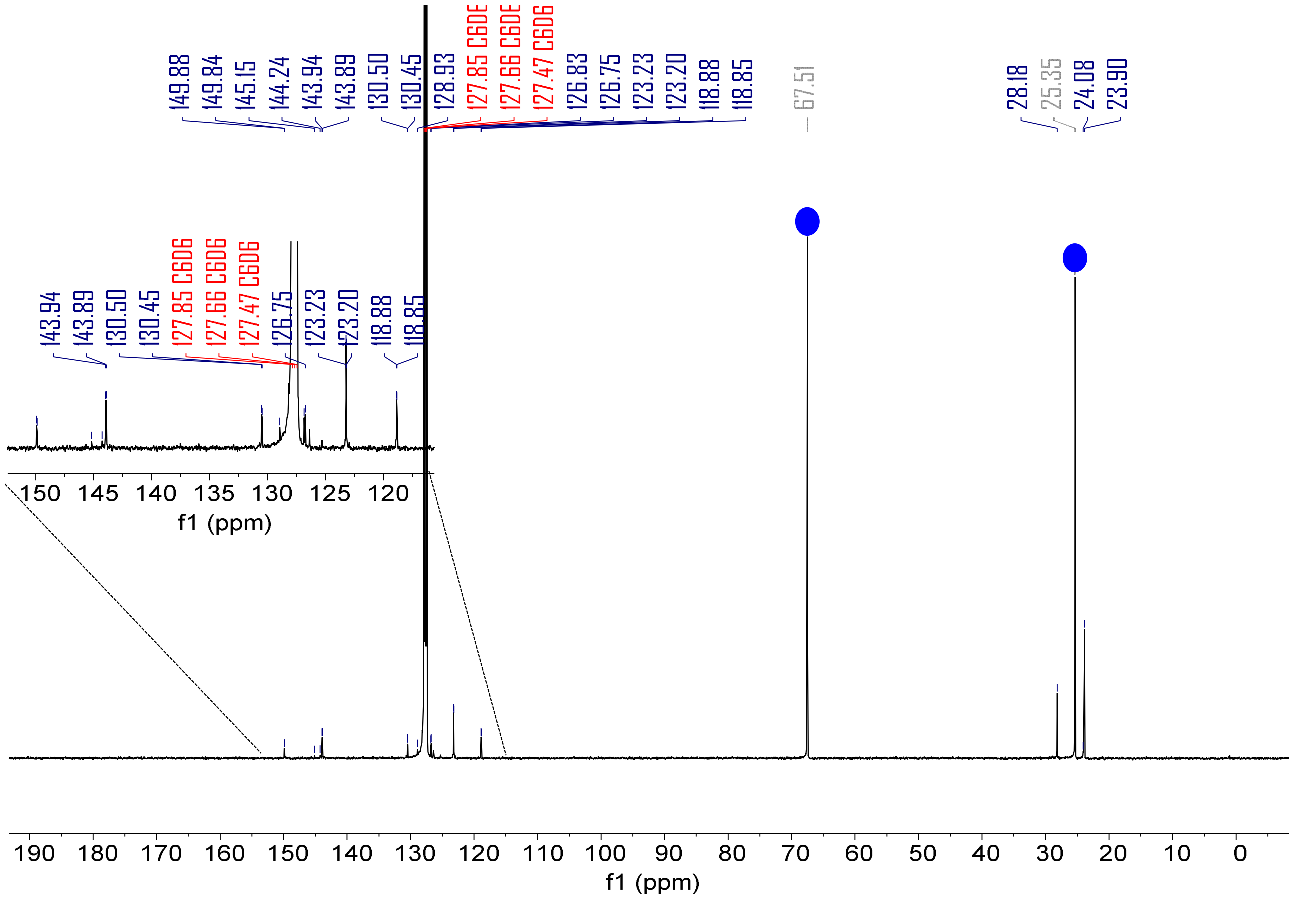

Figure S9. $^{13}C\{^{1}H\}$-NMR spectrum (125.7 MHz, C_6D_6, 298 K) of [TipLi$_2$] **3** (obtained after adding a drop of THF to a deuterated benzene solution of **3**). The blue circles denote the resonances of THF.

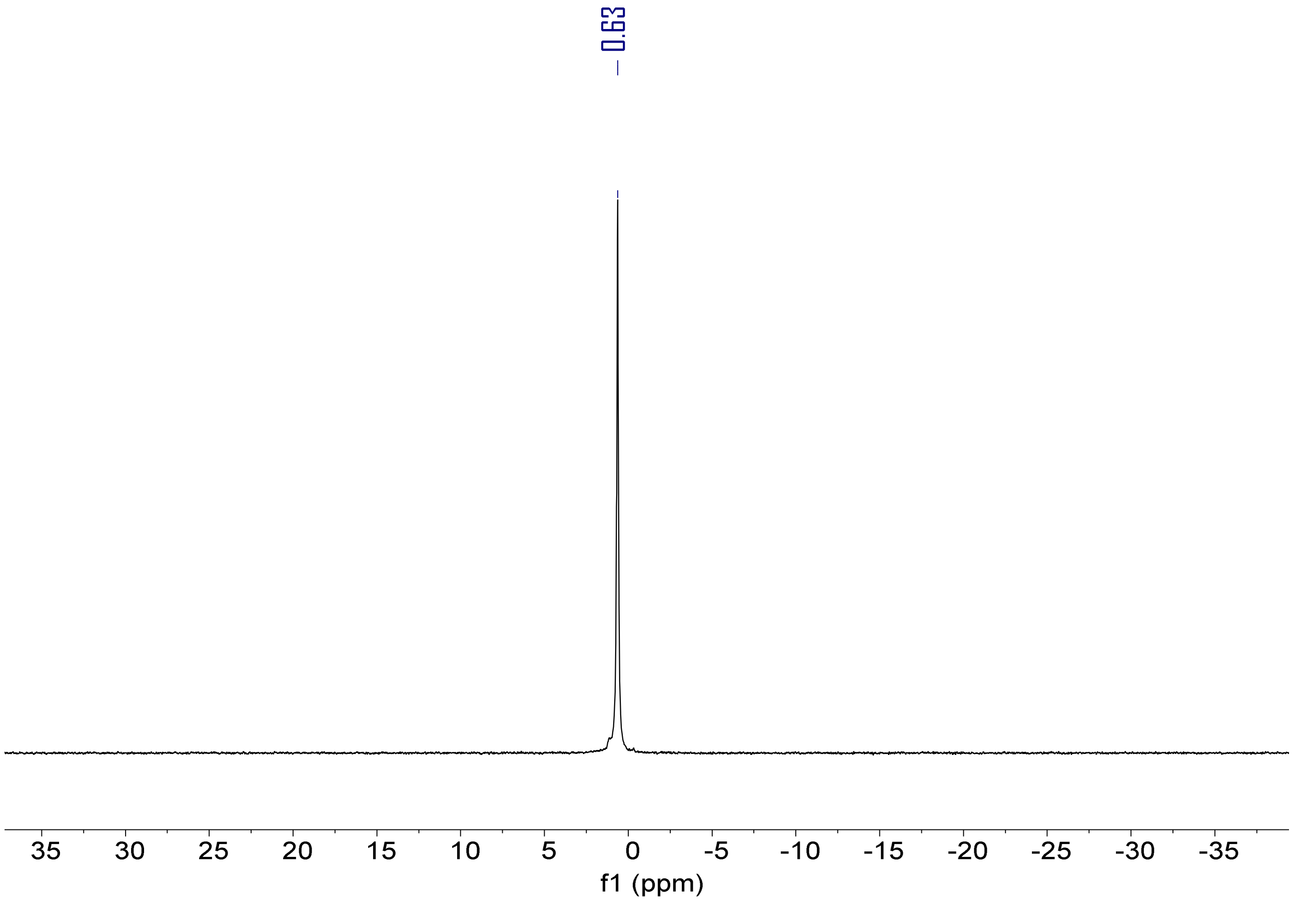

Figure S10. $^{7}Li\{^{1}H\}$-NMR spectrum (155.5 MHz, C_6D_6, 300 K) of [TipLi$_2$] **3** (obtained after adding a drop of THF to the deuterated benzene solution of **3**).

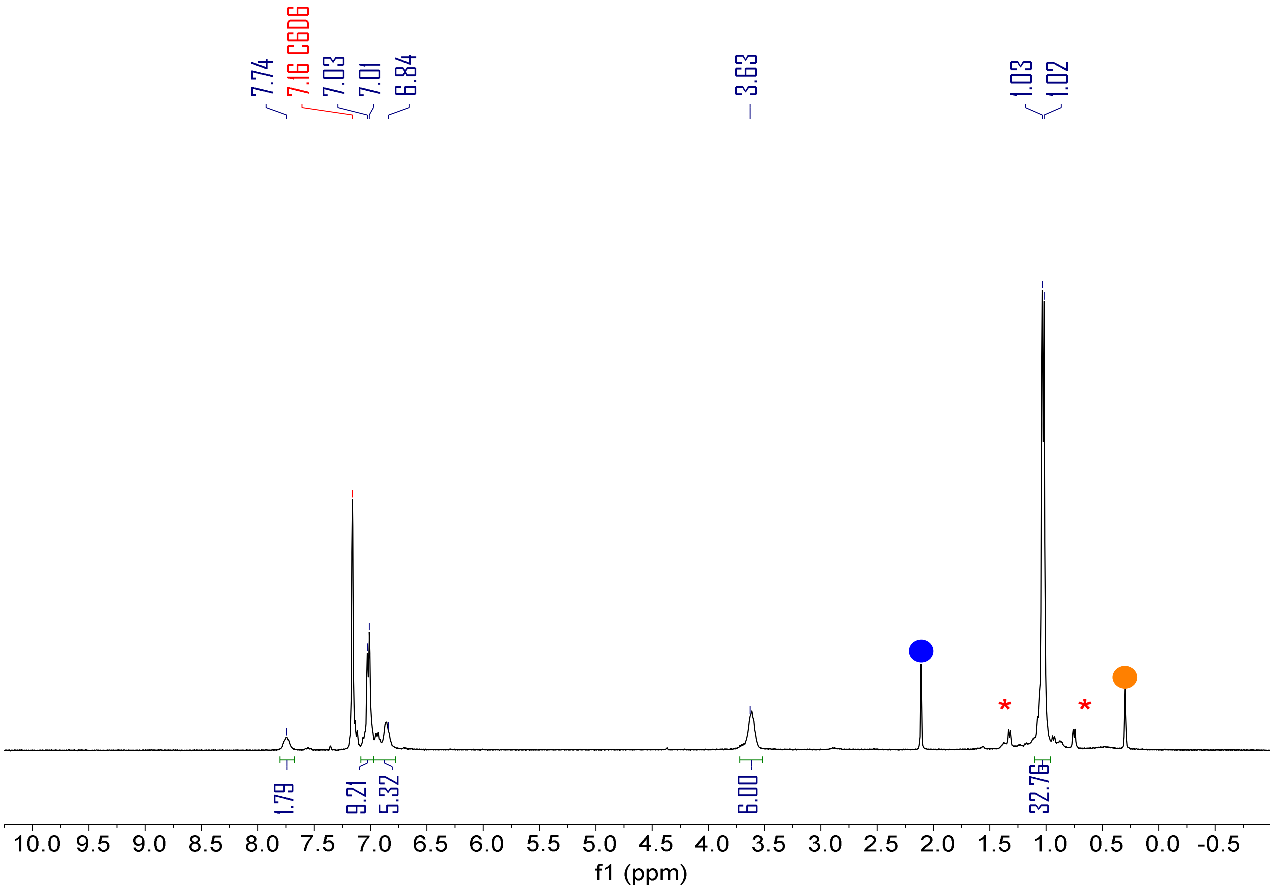

Figure S11. ^{1}H-NMR spectrum (400.1 MHz, C_6D_6, 300 K) of [$TipLi_2$] **3** without the addition of THF. Note that the sample was poorly dissolved. The blue circles denote the resonances of toluene. The orange circle denotes the resonance of silicone grease. The red asterisks denote the chemical resonances of a trace amount of [HTipLi].

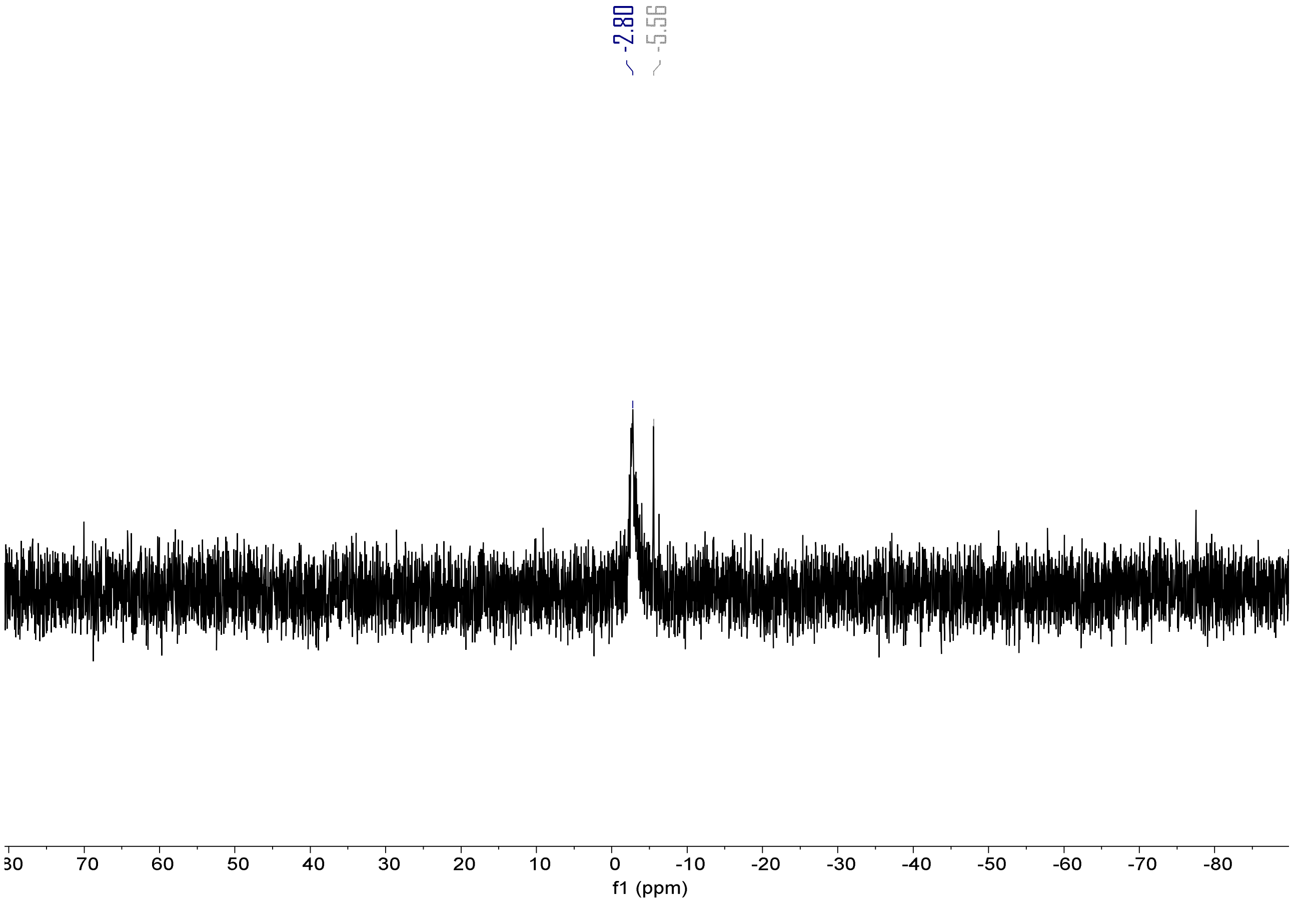

Figure S12. $^{31}P\{^{1}H\}$ NMR spectrum (162.0 MHz, C_6D_6, 300 K) of [$TipLi_2$] **3** without the addition of THF (the sample was poorly dissolved), showing a broad signal at around δ_p = -2.80 ppm. The gey-labelled peak corresponds to the monolithiated intermediate [HTipLi].

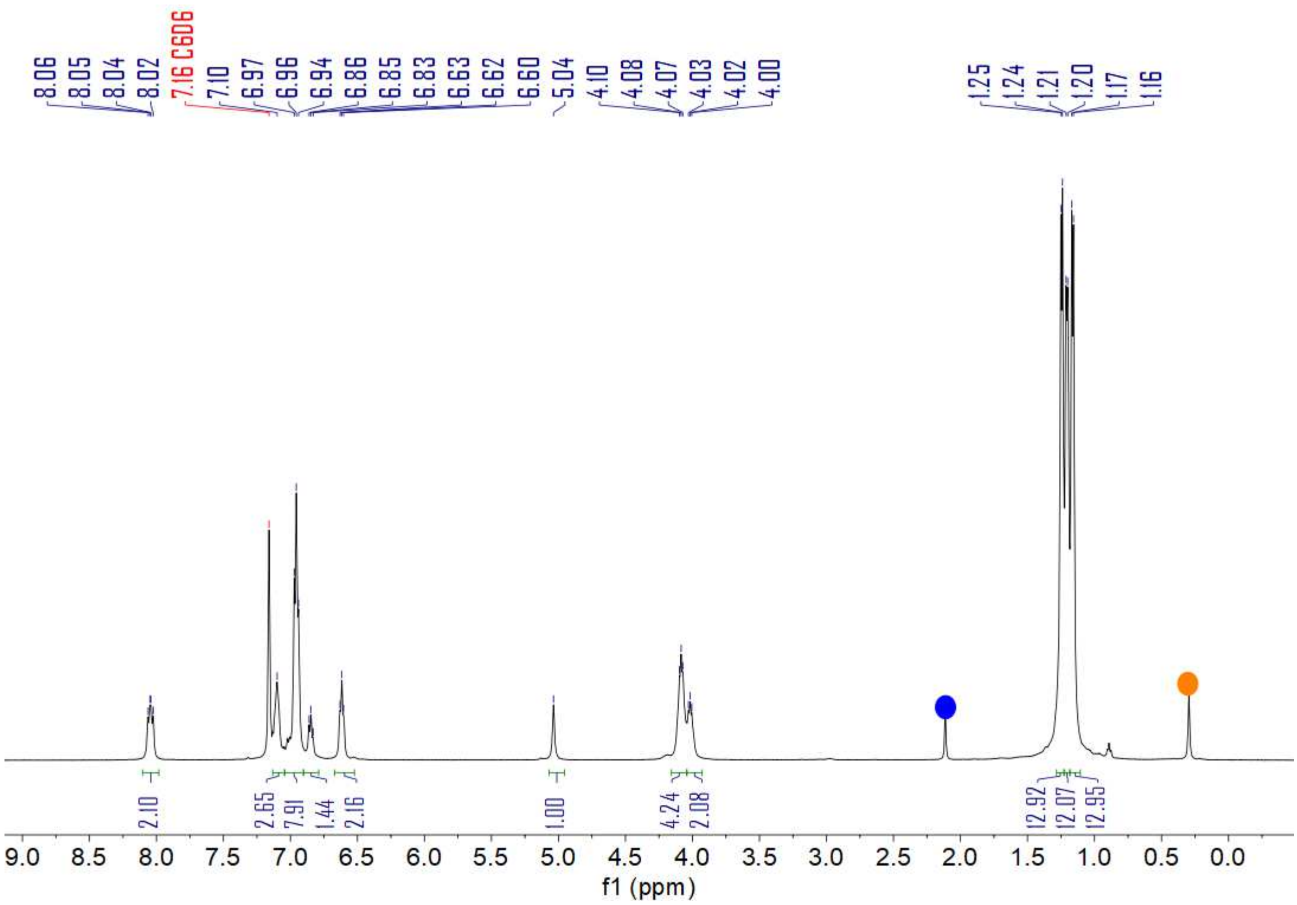

Figure S13. ^{1}H-NMR spectrum (499.9 MHz, C_6D_6, 298 K) of [(TipH)K]. The blue circle denotes the resonance of toluene. The orange circle denotes the resonance of silicone grease.

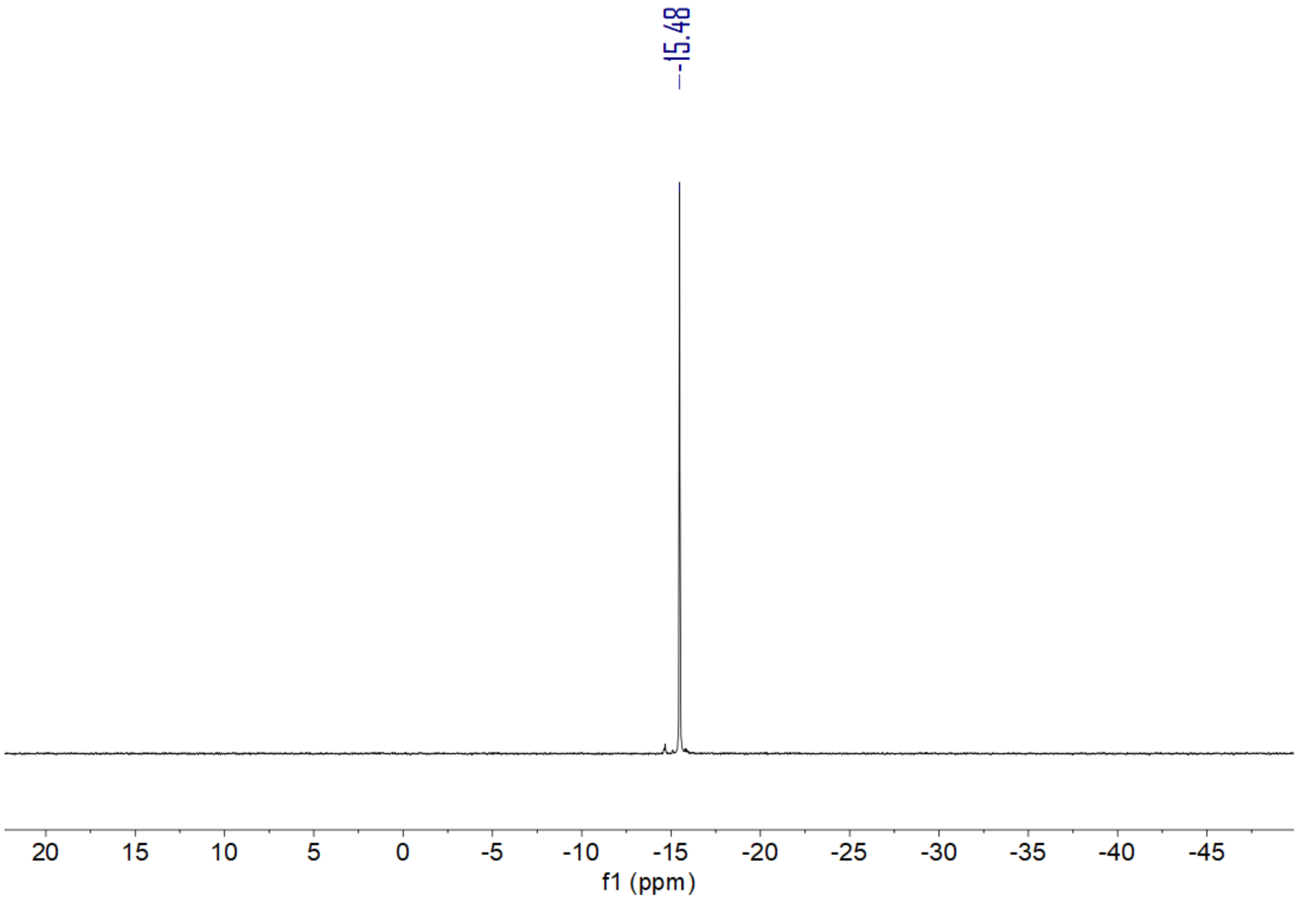

Figure S14. $^{31}P\{^{1}H\}$ NMR spectrum (202.4 MHz, C_6D_6, 298 K) of [(TipH)K].

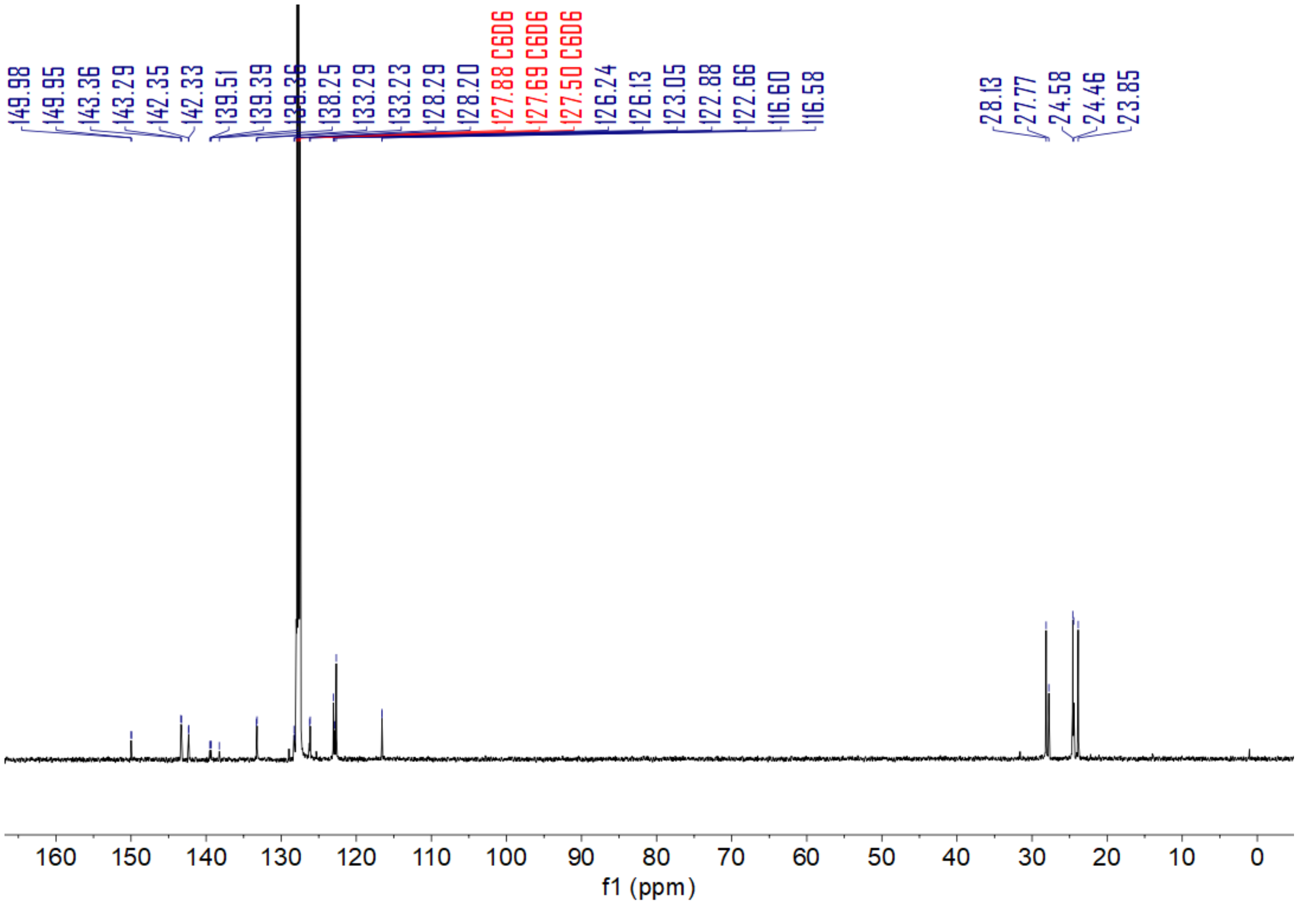

Figure S15. $^{13}C\{^1H\}$ NMR spectrum (101.0 MHz, C_6D_6, 300 K) of [(TipH)K].

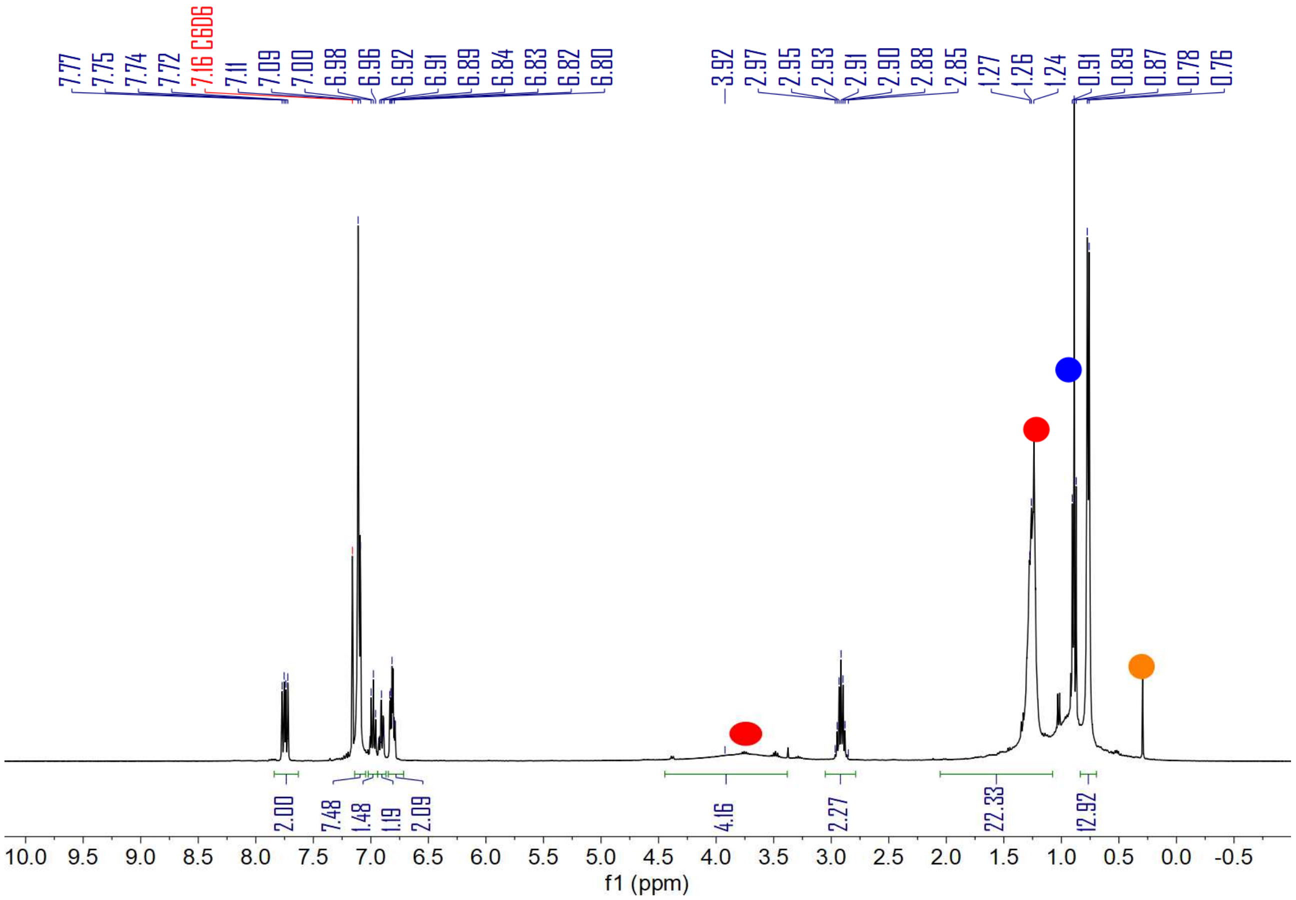

Figure S16. ^{1}H-NMR spectrum (400.1 MHz, C_6D_6, 298 K) of TipSi **4** at ambient temperature. The blue circle denotes the resonance of residual *n*-hexane. The orange circle denotes the resonance of silicone grease. The broad resonances (red circles) are assigned to the protons of the Dip-isopropyl groups that are extremely broad at this temperature.

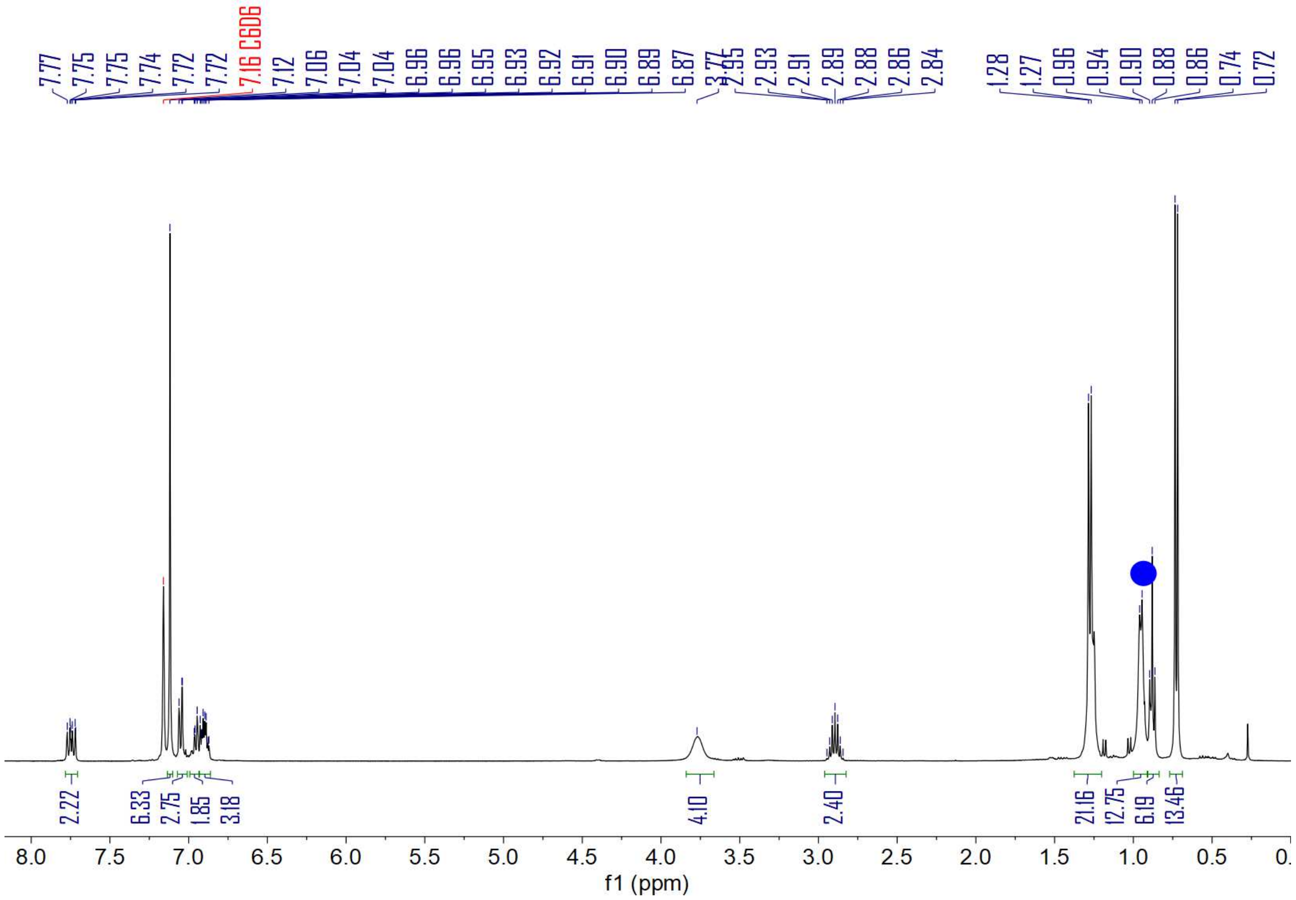

Figure S17. ^{1}H-NMR spectrum (400.0 MHz, C_6D_6, 339 K) of TipSi **4** at 65 °C. The blue circle denotes the resonance of residual *n*-hexane.

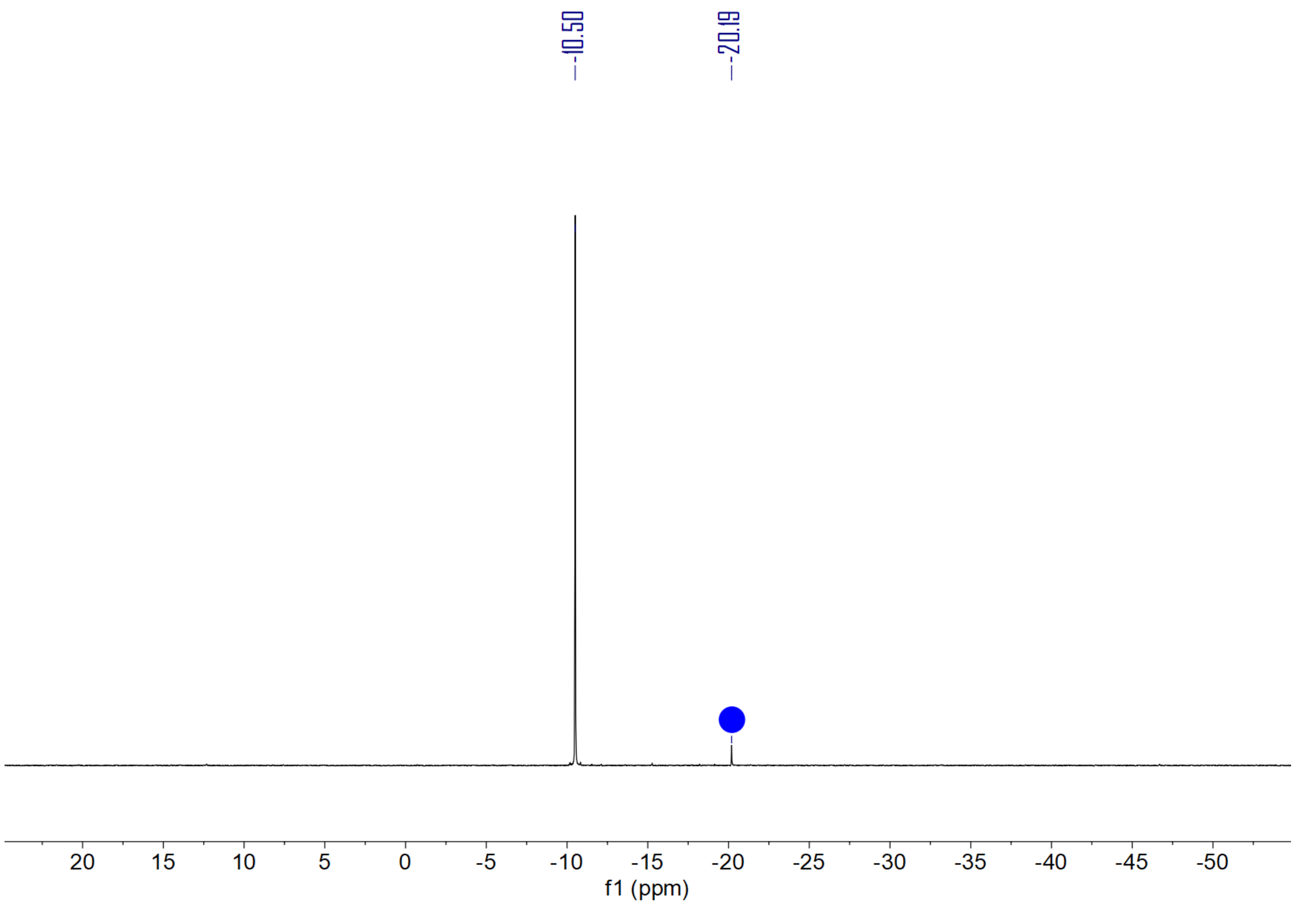

Figure S18. $^{31}P\{^{1}H\}$ NMR spectrum (162.3 MHz, C_6D_6, 338 K) of TipSi **4**. The blue circle denotes the chemical resonance of proligand $TipH_2$ **2**.

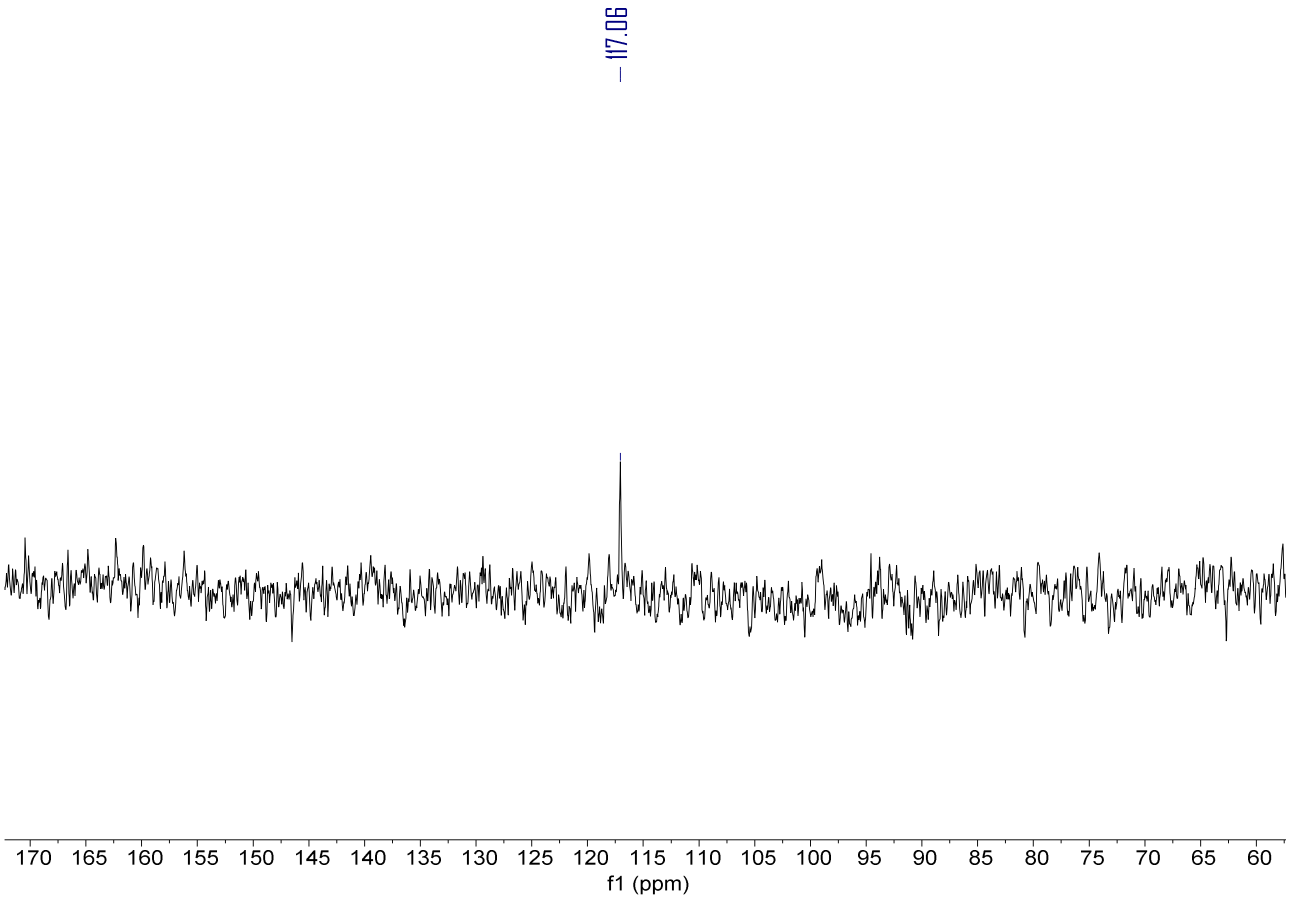

Figure S19. $^{29}Si\{^{1}H\}$ NMR spectrum (79.5 MHz, C_6D_6, 338 K) of TipSi **4**. Note that this resonance could not be detected via $^{1}H,^{29}Si$ HMBC NMR experiments.

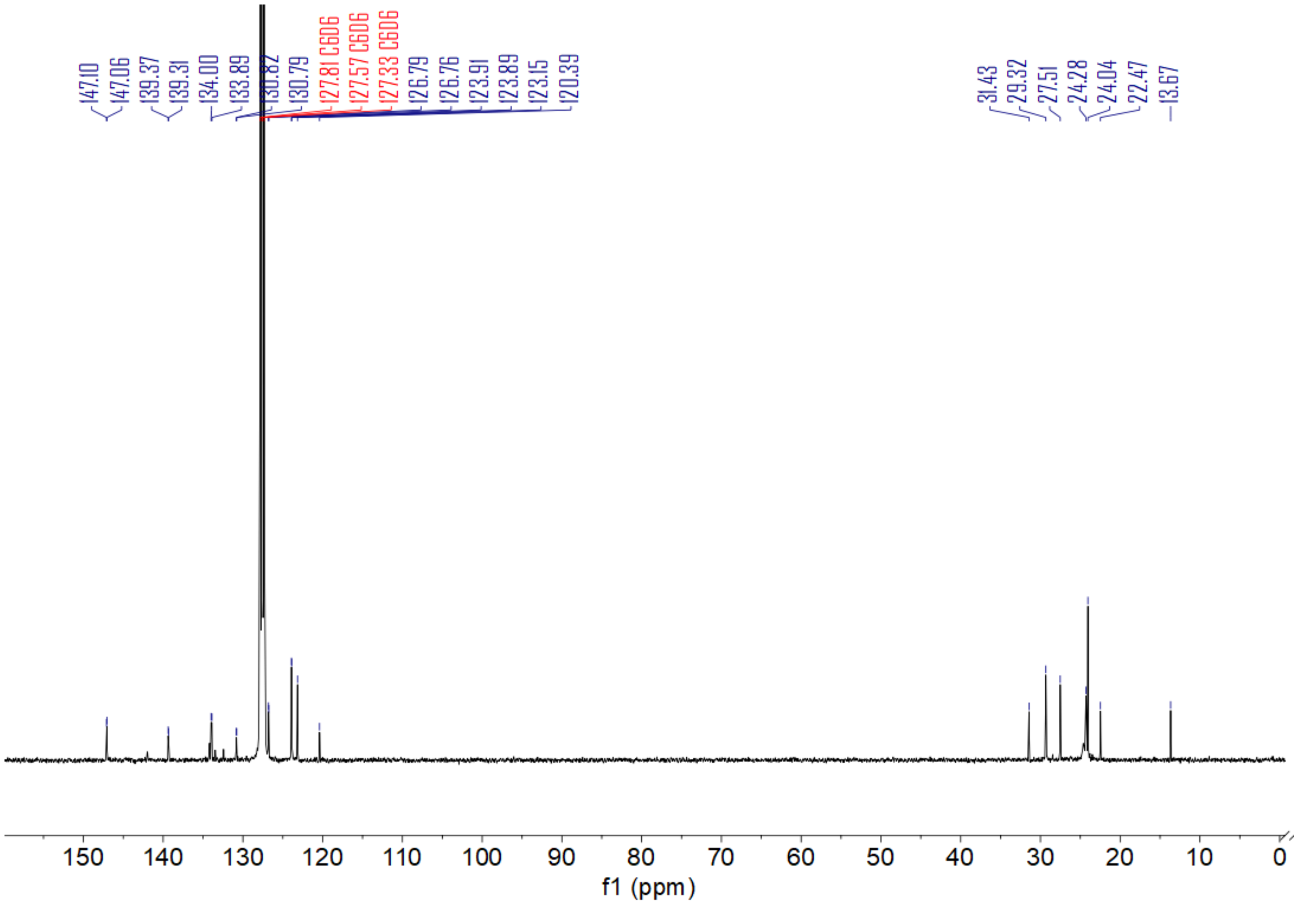

Figure S20. $^{13}C\{^{1}H\}$ NMR spectrum (100.6 MHz, C_6D_6, 338 K) of TipSi **4**.

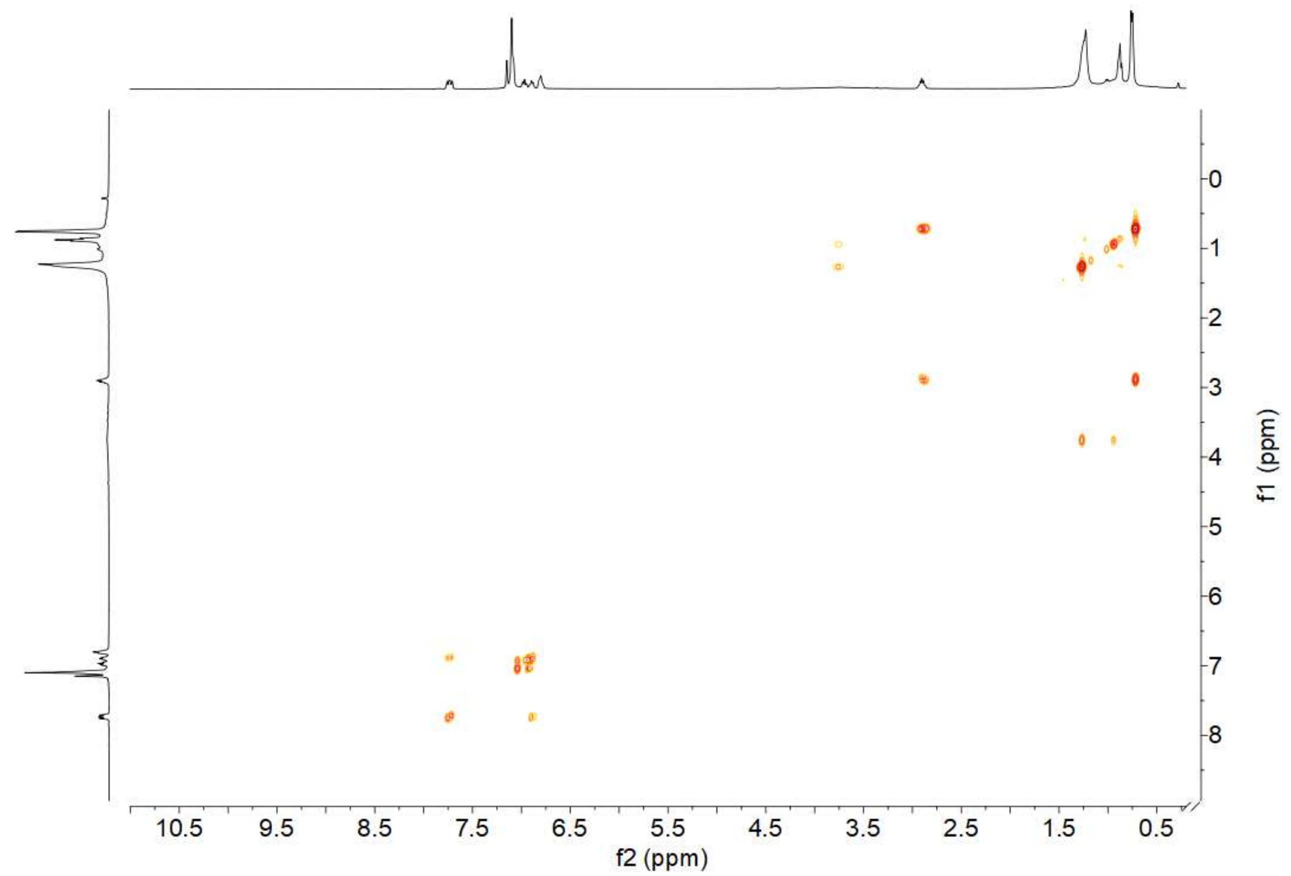

Figure S21. ^{1}H-^{1}H COSY NMR spectrum of TipSi **4**.

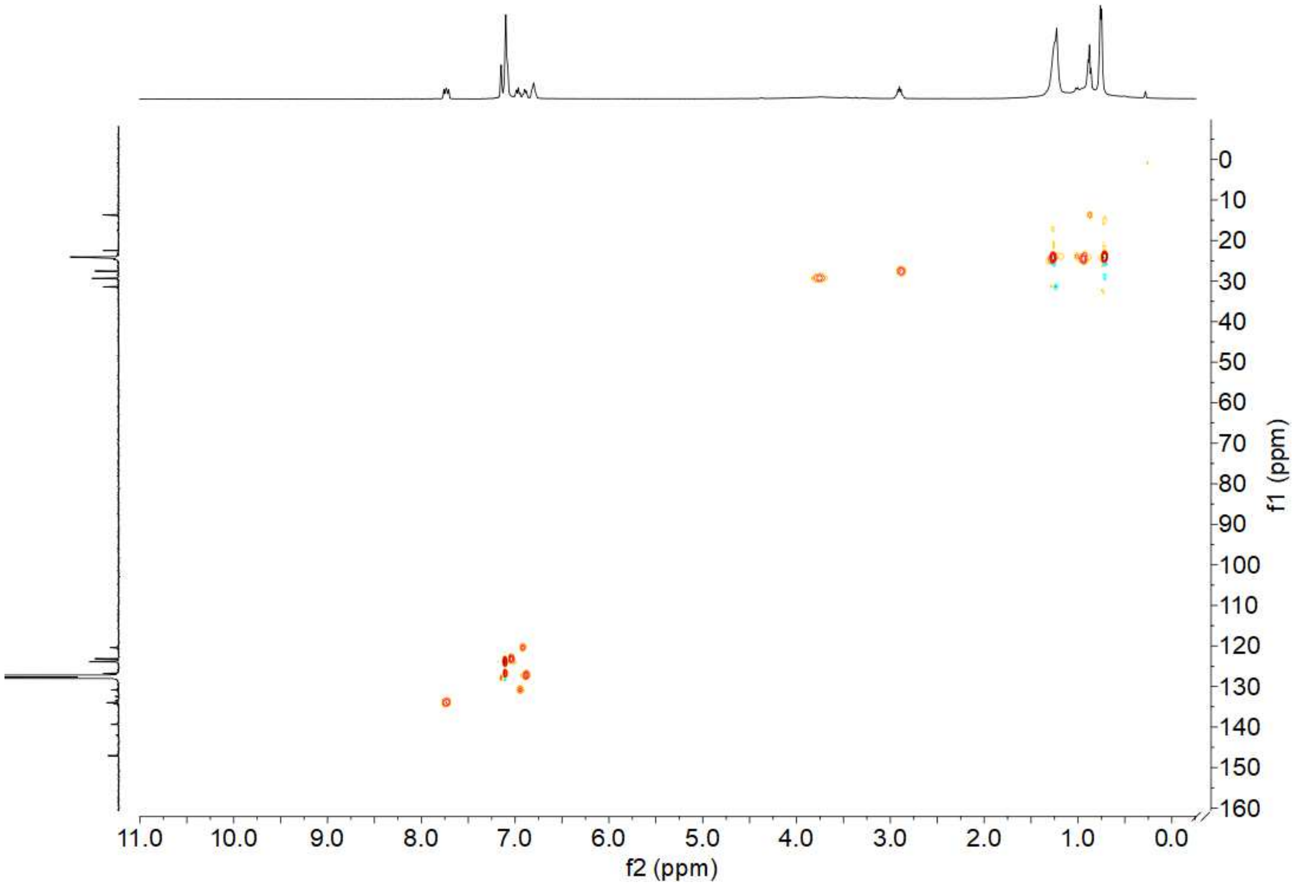

Figure S22. ^{1}H-^{13}C HSQC NMR spectrum of TipSi **4**.

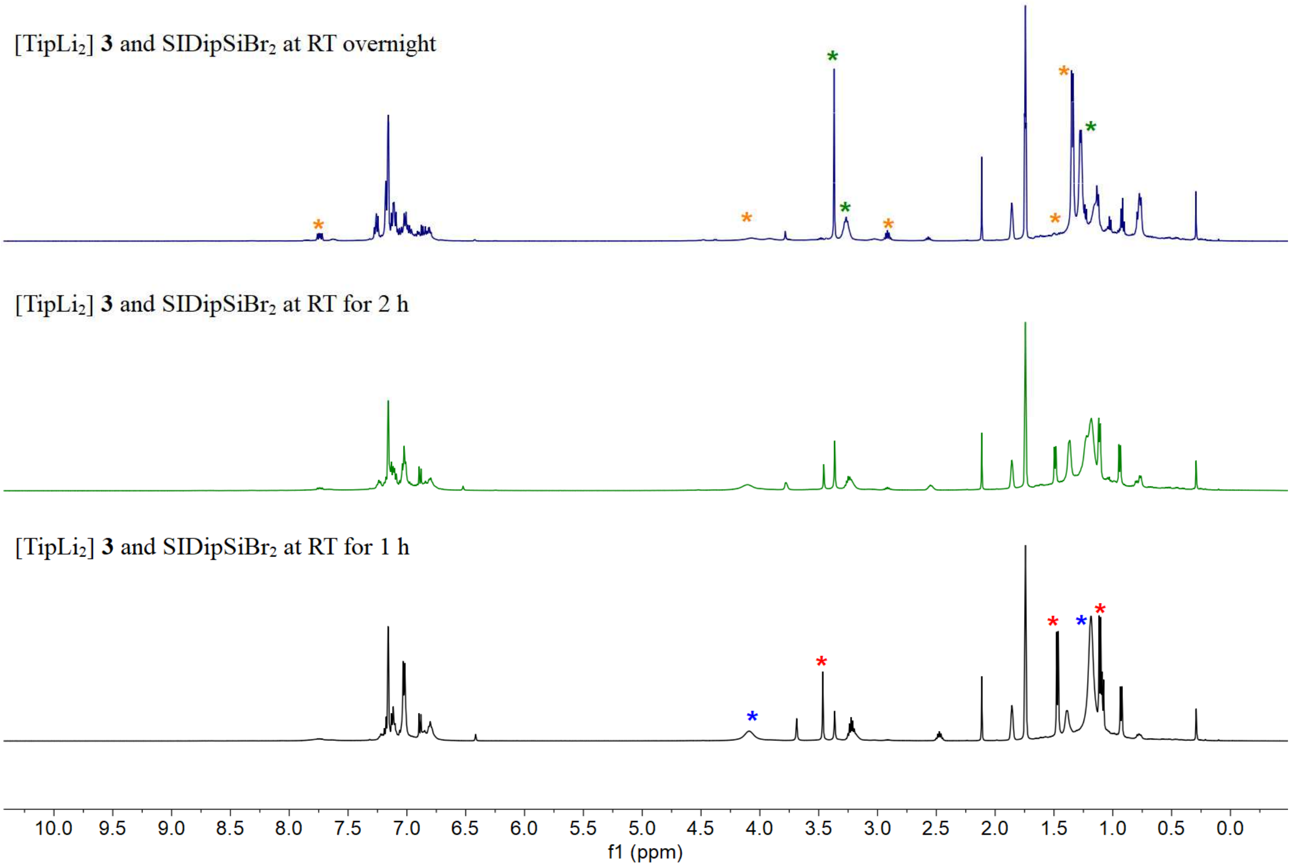

Figure S23. Stacked ^{1}H-NMR spectra (499.9 MHz, C_6D_6, 298 K) of the reaction progress between [TipLi$_2$] **3** (15 mg, 23.16 µmol, 1 equiv.) and SIDipSiBr$_2$ (13.4 mg, 23.16 µmol, 1 equiv.) in C_6D_6 (0.6 mL) at room temperature. Adamantane (2.2 mg, 16.15 µmol) was added as an internal standard. In the bottom spectrum, the blue asterisks denote the resonances of [TipLi$_2$] **3**, and the red asterisks denote the resonances of SIDipSiBr$_2$. In the top spectrum, the full consumption of two starting materials and the formation of TipSi **4** (orange asterisks) and free SIDip (green asterisks) were observed.

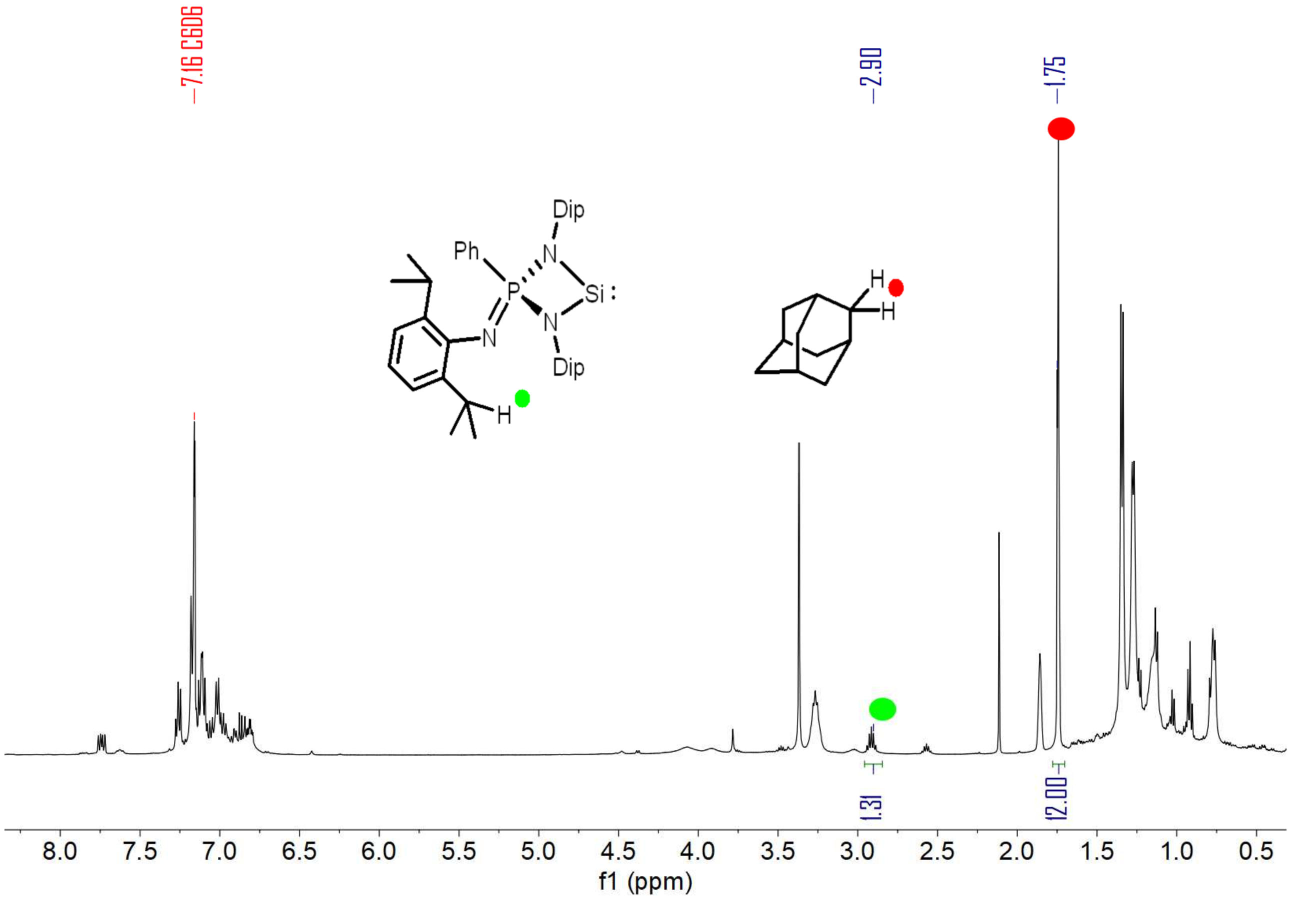

Figure S24. ^{1}H-NMR spectrum (499.9 MHz, C_6D_6, 298 K) of the *in-situ* NMR reaction between $[TipLi_2]$ **3** (15 mg, 23.16 µmol, 1 equiv.) and $SIDipSiBr_2$ (13.4 mg, 23.16 µmol, 1 equiv.) in C_6D_6 (0.6 mL) at room temperature. Adamantane (2.2 mg, 16.15 µmol) was added as an internal standard. An *in-situ* NMR yield of 46% for TipSi **4** was determined by integration against the resonances of adamantane.

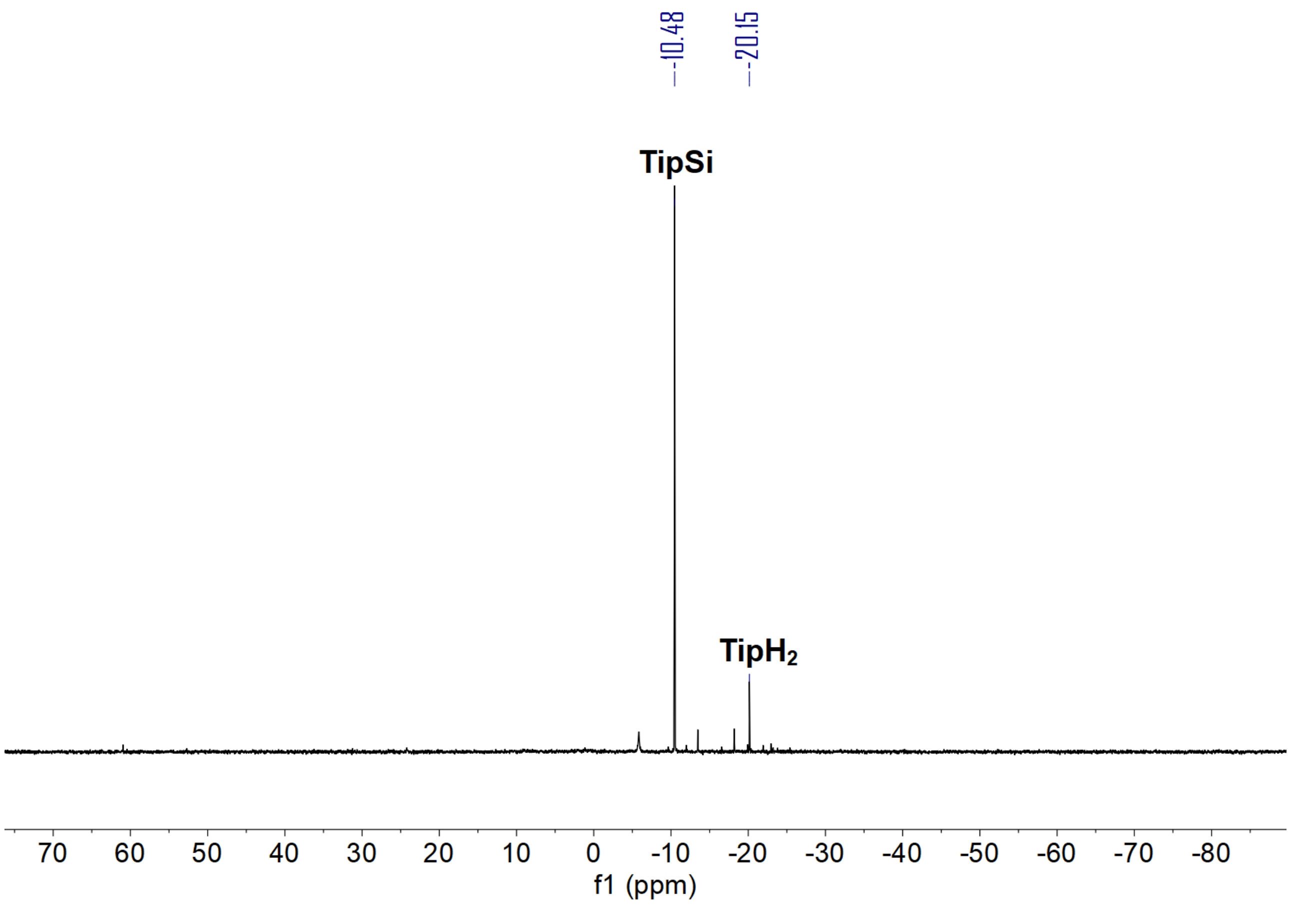

Figure S25. $^{31}P\{^{1}H\}$ NMR spectrum (202.4 MHz, C_6D_6, 338 K) of the *in-situ* NMR reaction between [$TipLi_2$] **3** and $SIDipSiBr_2$, showing formation of TipSi **4** and a small amount of the proligand $TipH_2$ **2**.

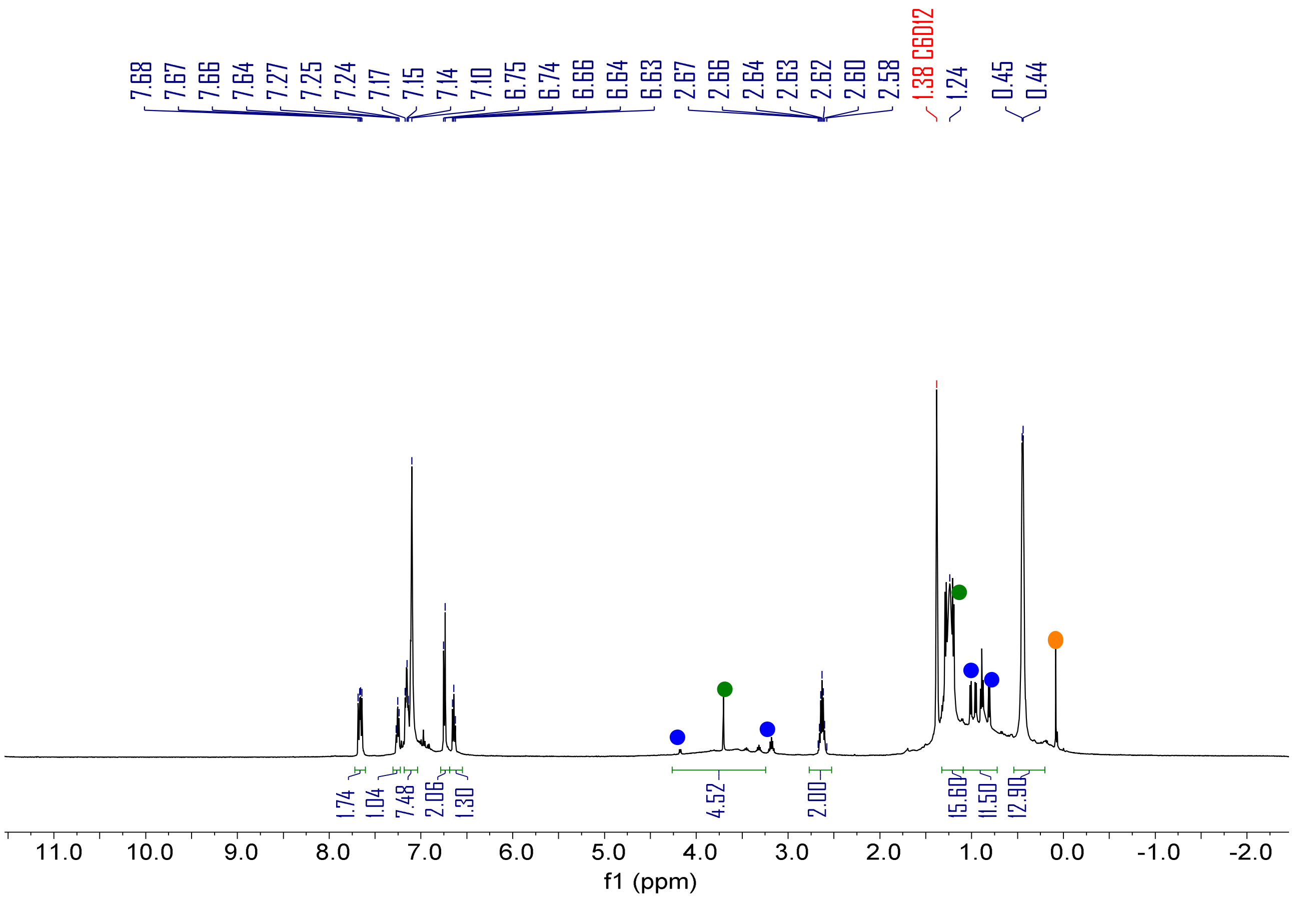

Figure S26. ^{1}H-NMR spectrum (499.9 MHz, C_6D_{12}, 300 K) of TipSi **4**. The sample contains ca. 15 mg of the compound fully dissolved in ca. 0.6 mL of C_6D_{12}. The blue circles denote the chemical resonances of $TipH_2$, and the green circles denote the chemical resonances of traceable SIPr. The orange circle denotes the resonance of silicone grease.

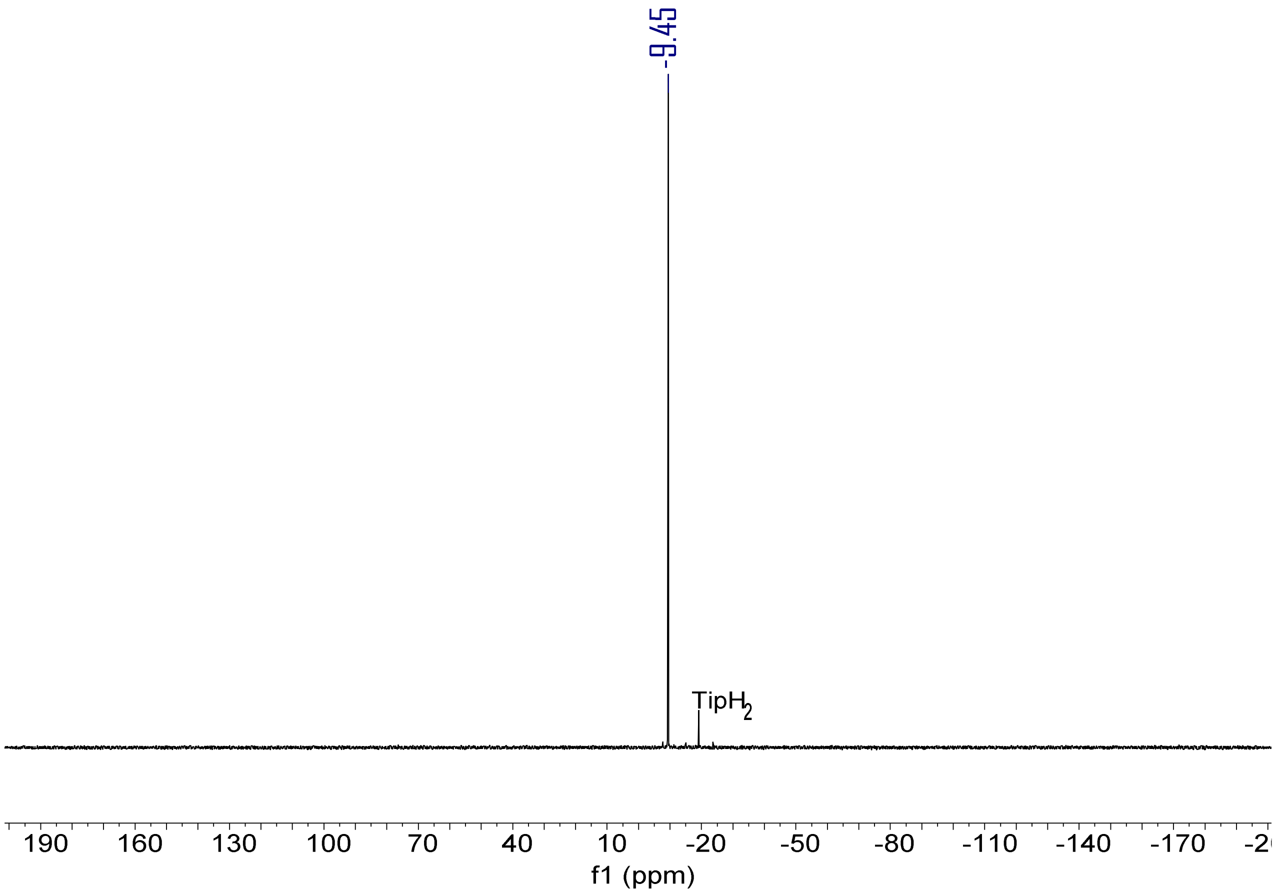

Figure S27. $^{31}P\{^{1}H\}$ NMR spectrum (202.4 MHz, C_6D_{12}, 300 K) of TipSi **4**. The sample contains ca. 15 mg of the compound fully dissolved in ca. 0.6 mL of C_6D_{12}.

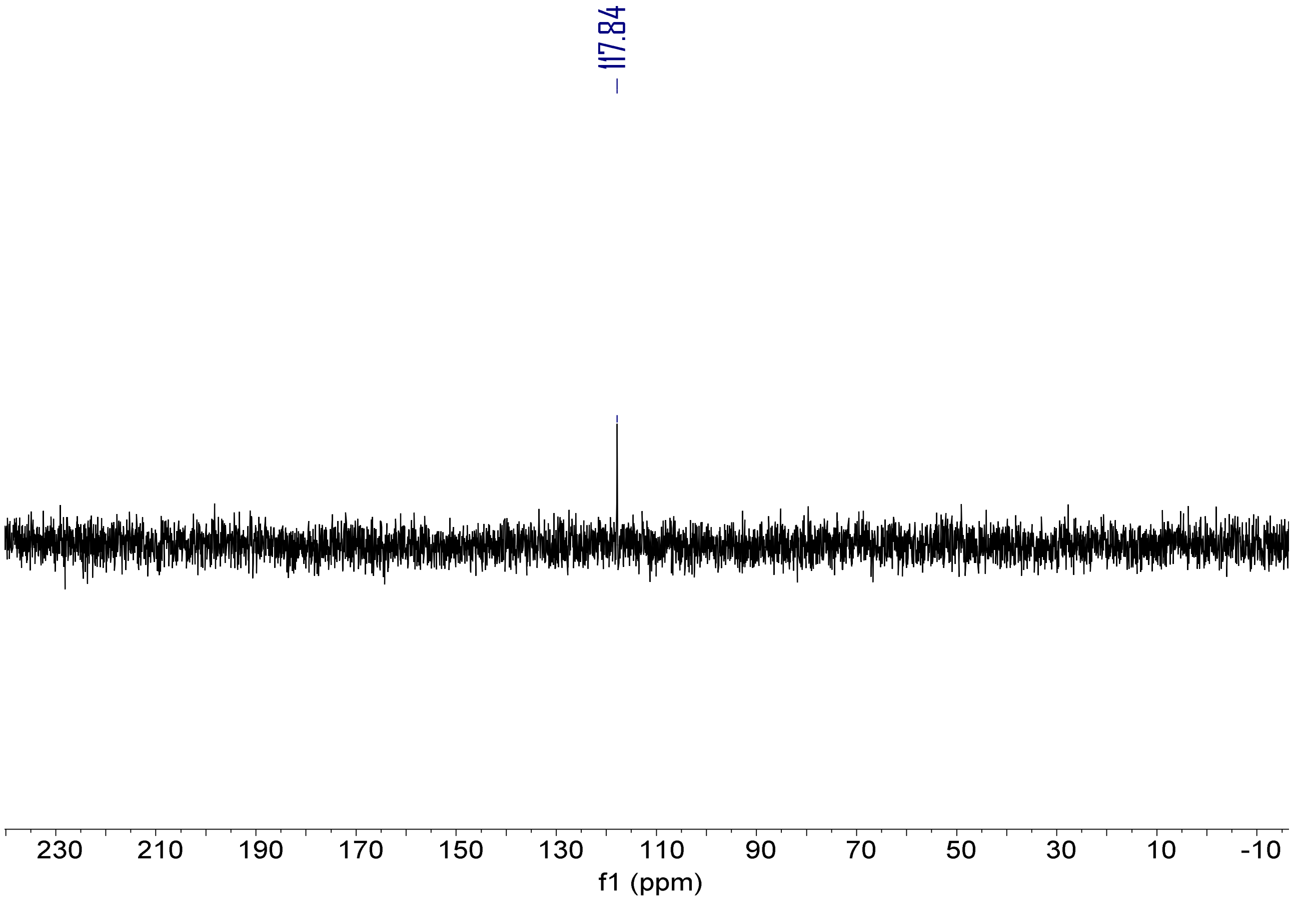

Figure S28. $^{29}Si\{^{1}H\}$ NMR spectrum (99.3 MHz, C_6D_{12}, 300 K) of TipSi **4**. The sample contains ca. 15 mg of the compound fully dissolved in ca. 0.6 mL of C_6D_{12}.

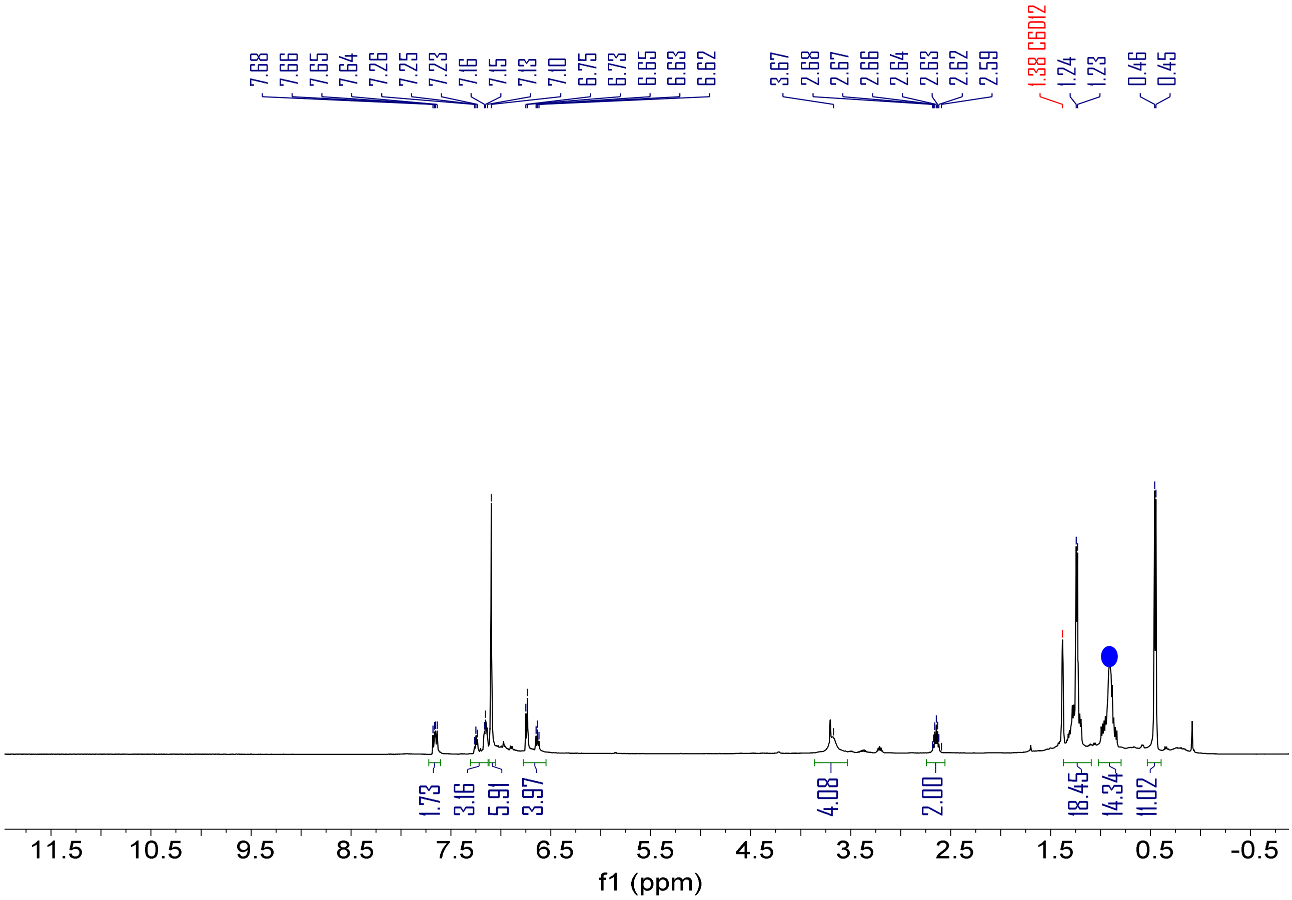

Figure S29. ^{1}H-NMR spectrum (499.9 MHz, C_6D_{12}, 338 K) of TipSi **4**. The sample contains ca. 20 mg of the compound fully dissolved in ca. 0.6 mL of C_6D_{12}. The blue circles denote the chemical resonances of residual *n*-hexane.

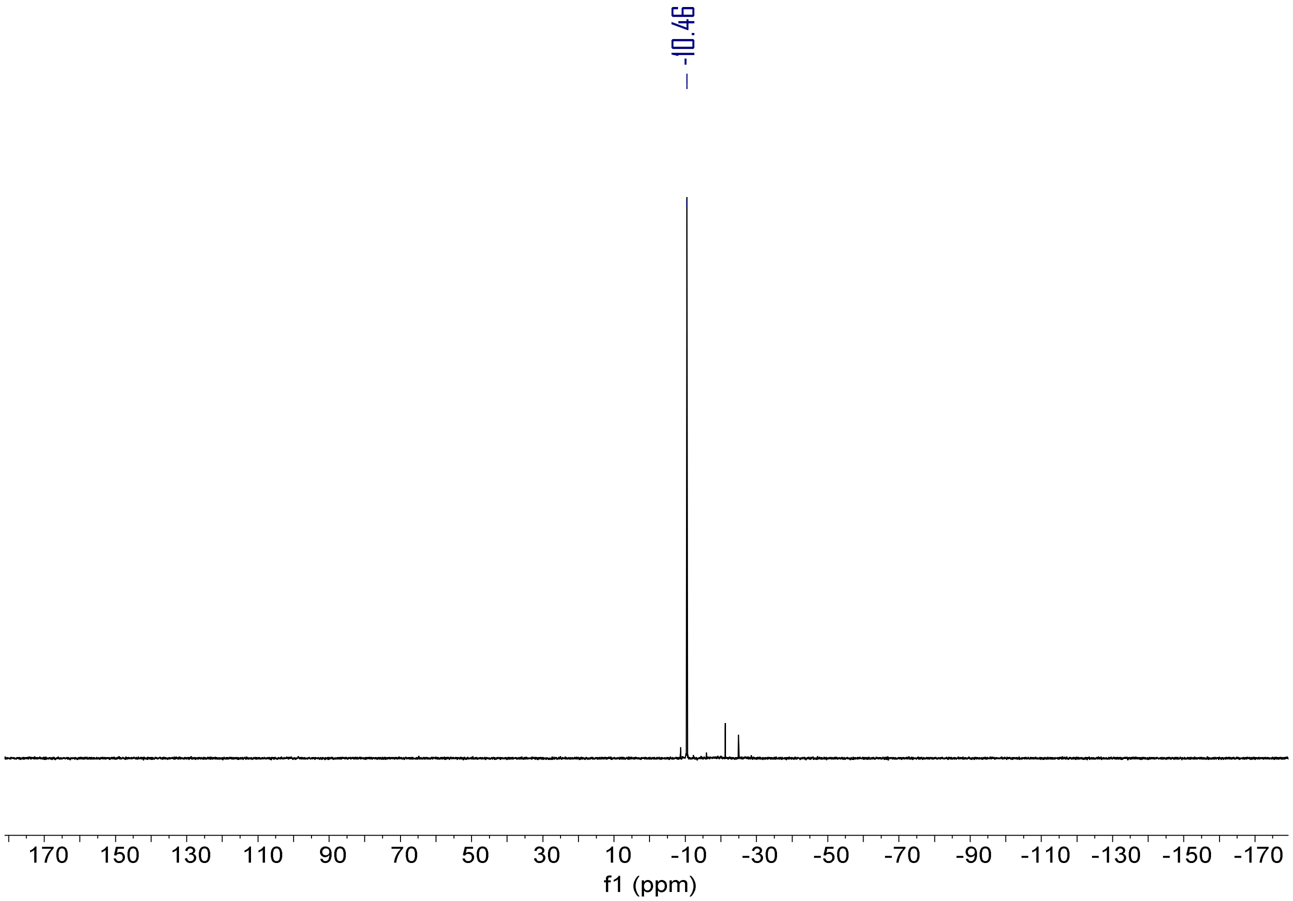

Figure S30. $^{31}P\{^{1}H\}$ NMR spectrum (202.4 MHz, C_6D_{12}, 338 K) of TipSi **4**. The sample contains ca. 20 mg of the compound fully dissolved in ca. 0.6 mL of C_6D_{12}.

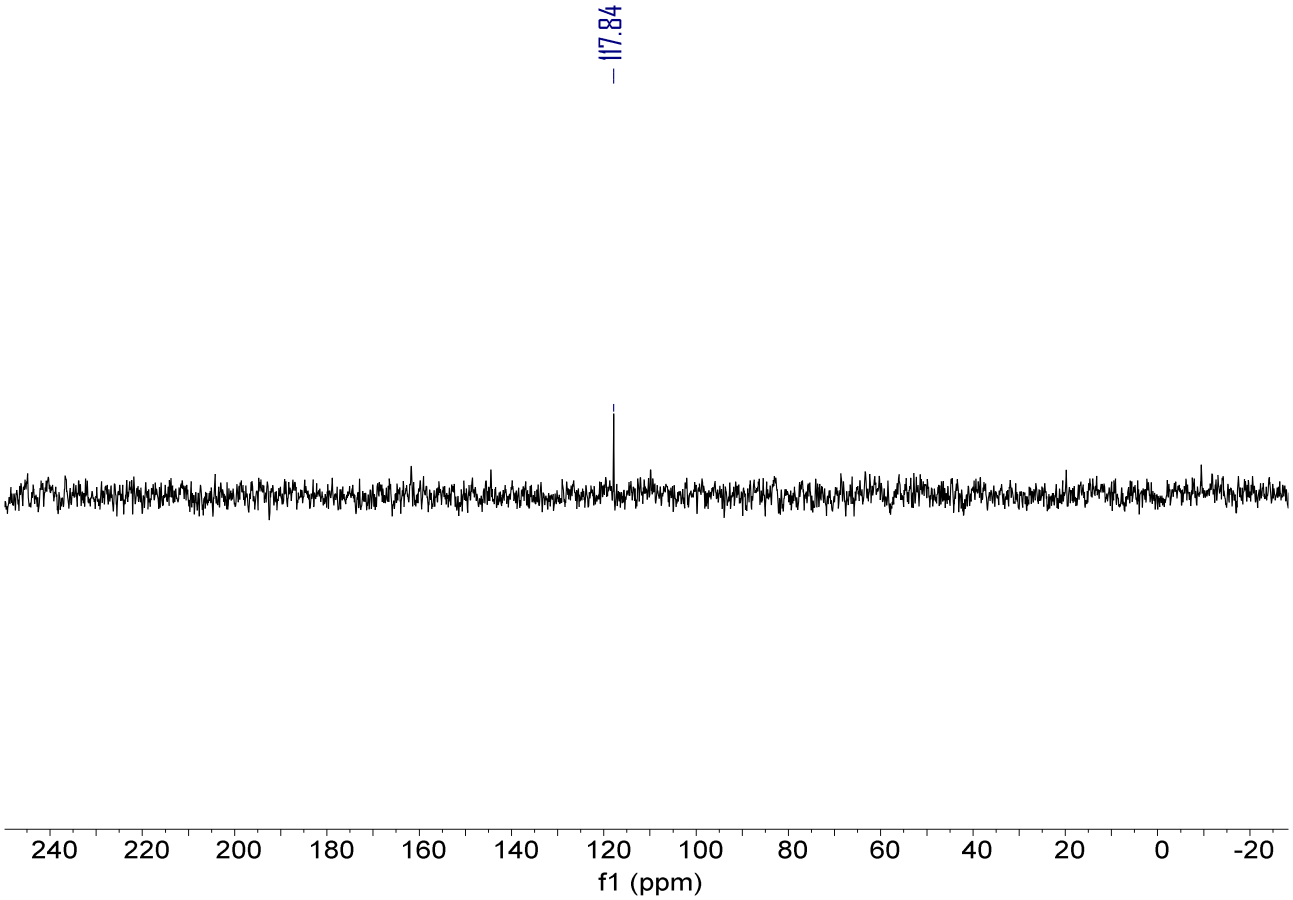

Figure S31. ^{29}Si{^{1}H} NMR spectrum (99.3 MHz, C_6D_{12}, 338 K) of TipSi **4**. The sample contains ca. 20 mg of the compound fully dissolved in ca. 0.6 mL of C_6D_{12}.

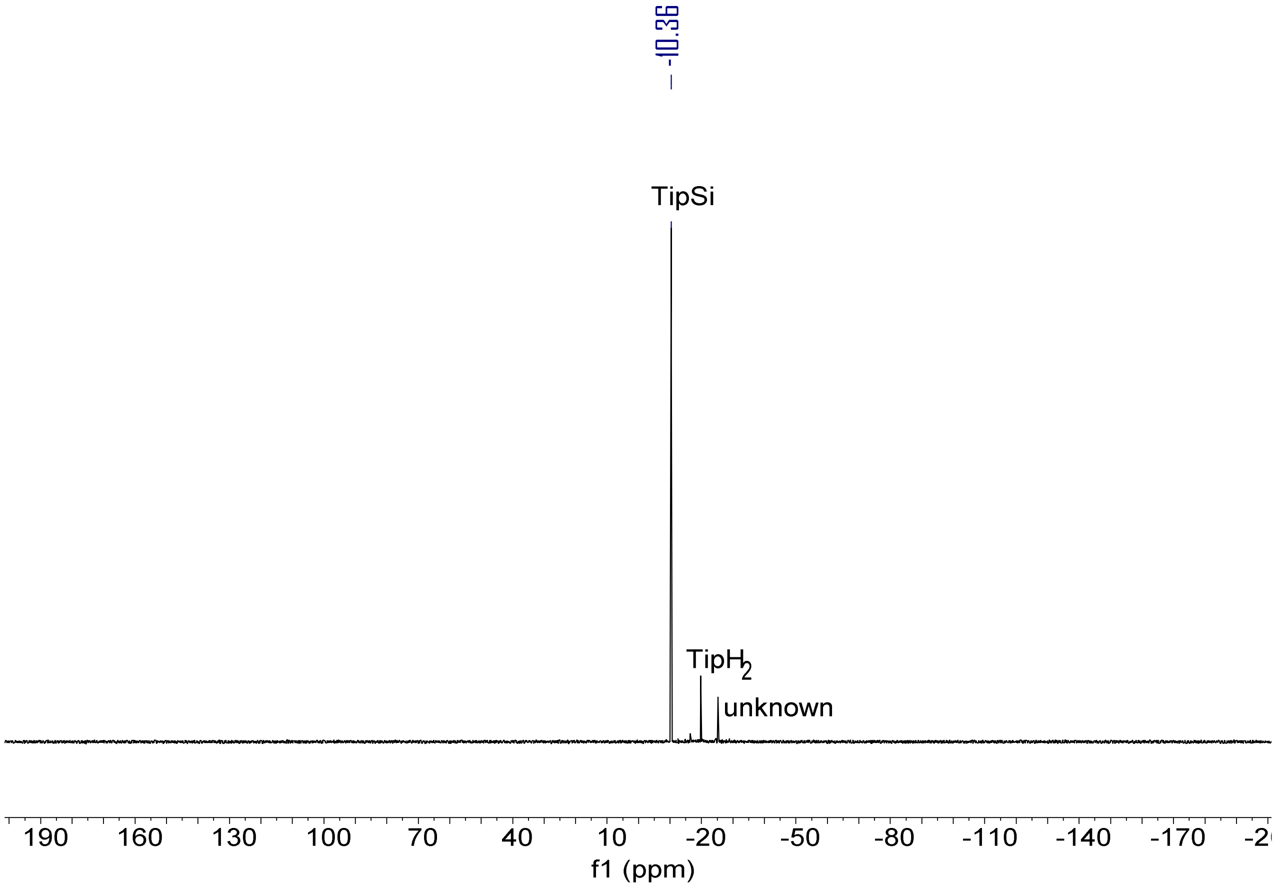

Figure S32. $^{31}P\{^1H\}$ NMR spectrum (202.4 MHz, *n*-hexane/C_6D_{12}, 273 K) of TipSi **4**. The sample contains ca. 30 mg of the compound predominantly dissolved in a solvent mixture (*n*-hexane/C_6D_{12} = 10:1 (v/v)).

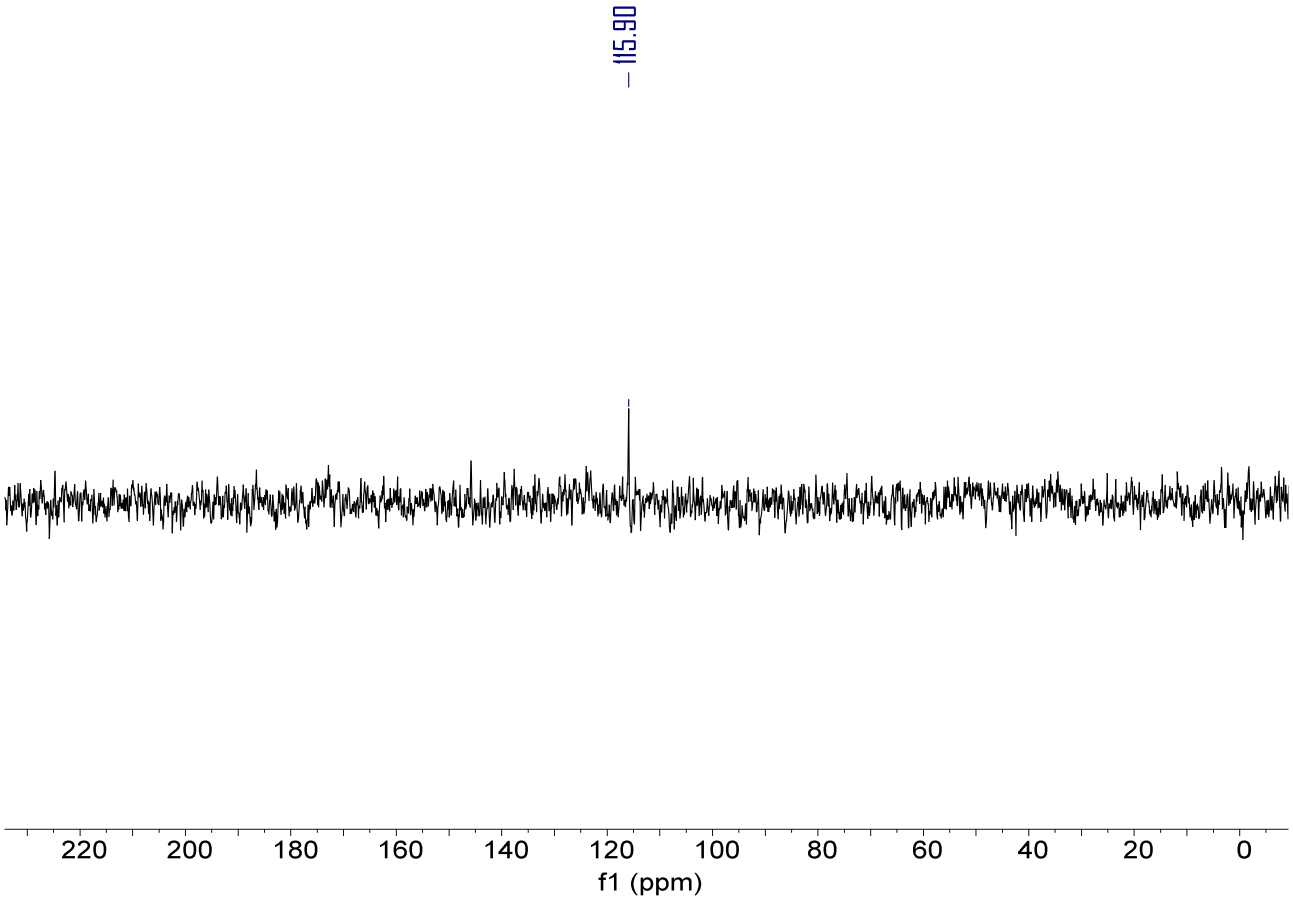

Figure S33. $^{29}Si\{^1H\}$ NMR spectrum (99.3 MHz, *n*-hexane/C_6D_{12}, 273 K) of TipSi **4**. The sample contains ca. 30 mg of the compound predominantly dissolved in a solvent mixture (*n*-hexane/C_6D_{12} = 10:1 (v/v)).

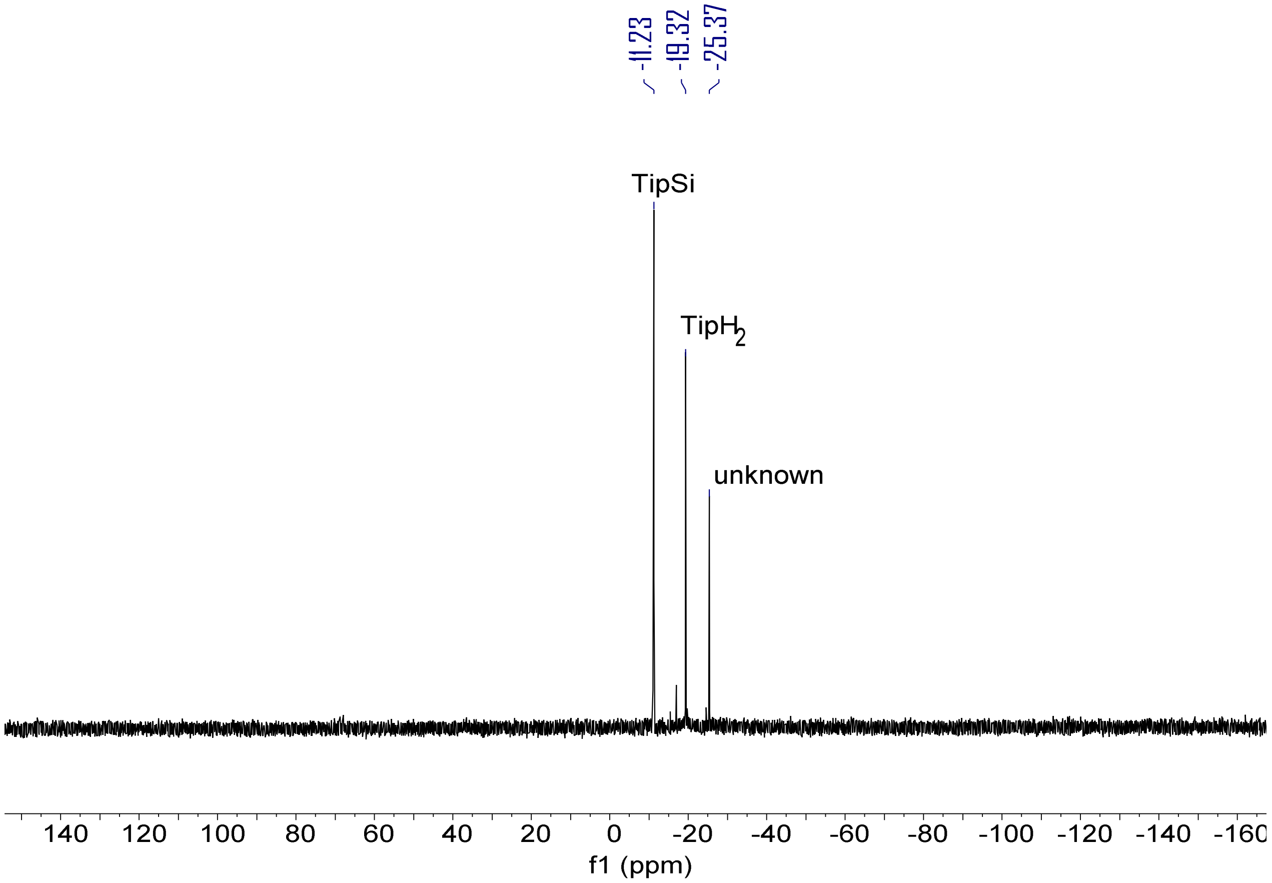

Figure S34. $^{31}P\{^1H\}$ NMR spectrum (202.4 MHz, *n*-hexane/C_6D_{12}, 243 K) of TipSi **4**. The sample contains ca. 30 mg of the compound and is only poorly dissolved in the solvent mixture (*n*-hexane/C_6D_{12} = 10:1 (v/v)) at this temperature. When compared to the spectrum in Figure S32, TipSi **4** is predominantly precipitated at this low temperature, resulting in low signal intensity for **4** in the spectrum. No $^{29}Si\{^1H\}$ NMR resonance could be detected at this temperature.

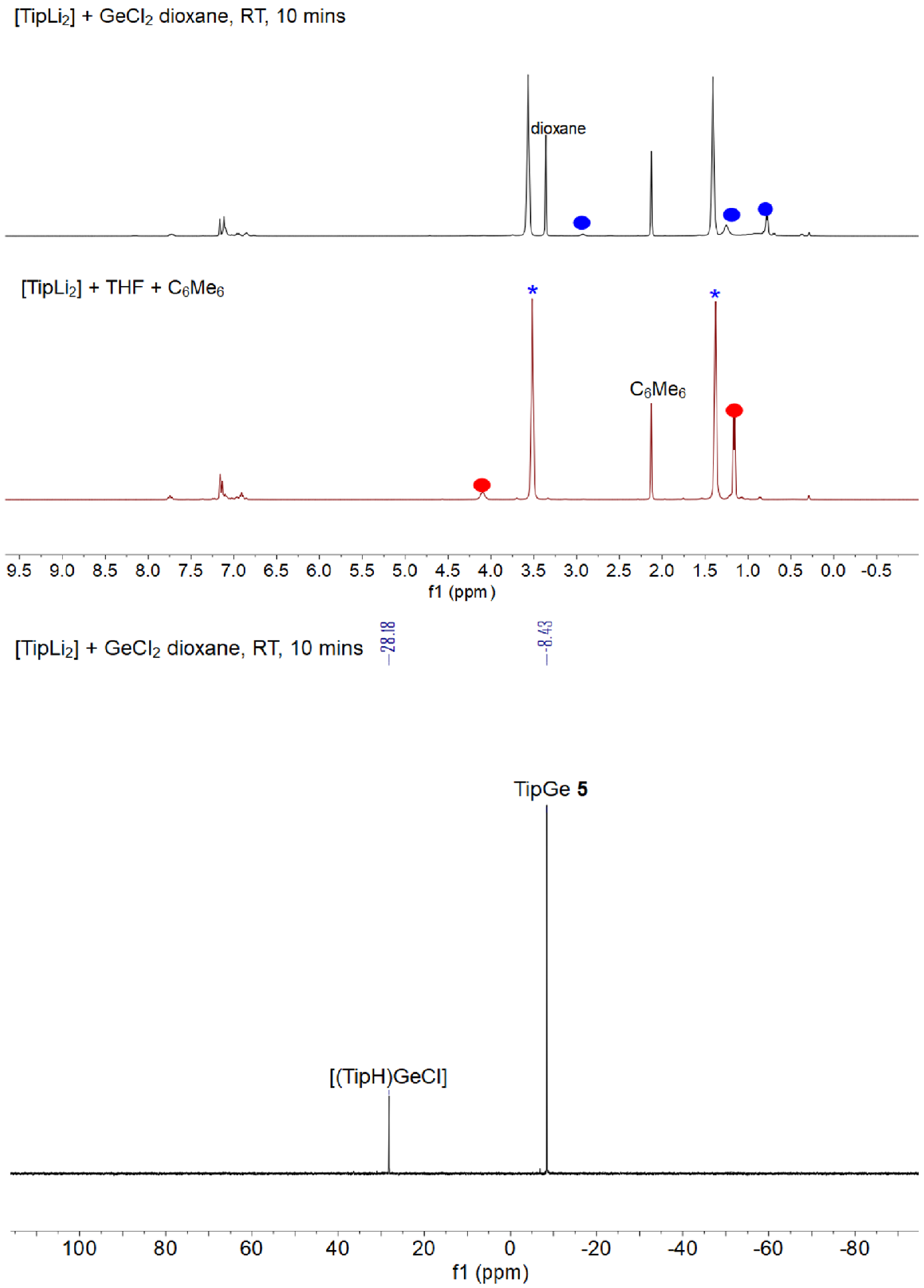

Figure S35. ^{1}H-NMR (400.1 MHz, C_6D_6, 300 K) and $^{31}P\{^{1}H\}$ NMR spectrum (121.5 MHz, C_6D_6, 300 K) of an *in-situ* reaction between [$TipLi_2$] **3** (6.50 mg, 10.1 µmol, 1 equiv.) and $GeCl_2$-dioxane adduct (2.85 mg, 12.3 µmol, 1.2 equiv.) in C_6D_6 (0.6 mL) and THF (ca. 20 µL), hexamethylbenzene (1.5 mg, 9.24 µmol, 1 equiv.) was added as an internal standard. Full consumption of [$TipLi_2$] **3** and formation of TipGe **5** were observed at ambient temperature for 10 minutes. The red circles denote the resonances of [$TipLi_2$] **3**, and the blue circles denote the resonances of TipGe **5**. The blue asterisks denote resonances of THF. The *in-situ* yield could not be accurately determined due to the broadness of NMR resonances.

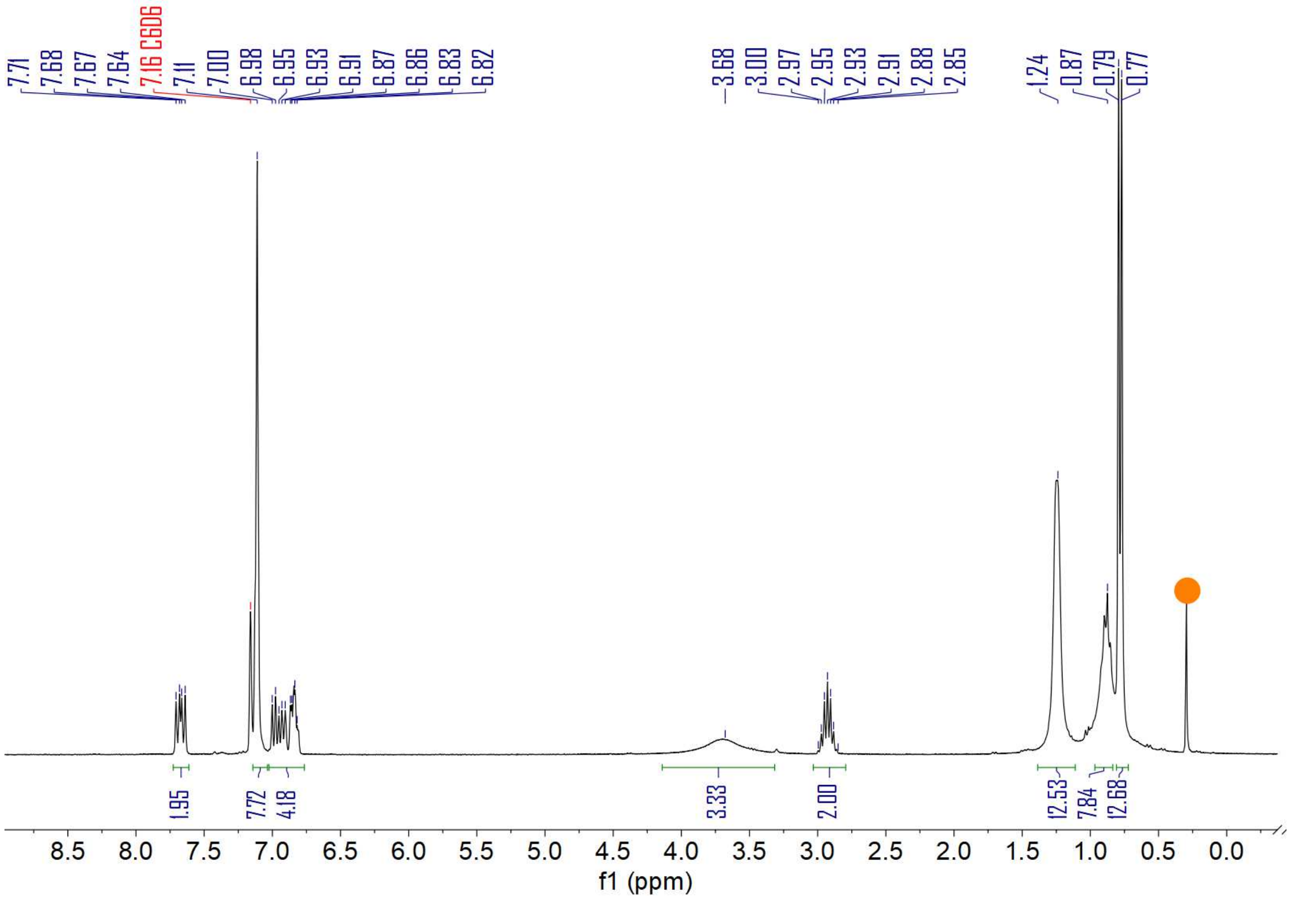

Figure S36. ^{1}H-NMR spectrum (300.0 MHz, C_6D_6, 298 K) of TipGe **5**. The orange circle denotes the resonance of silicone grease.

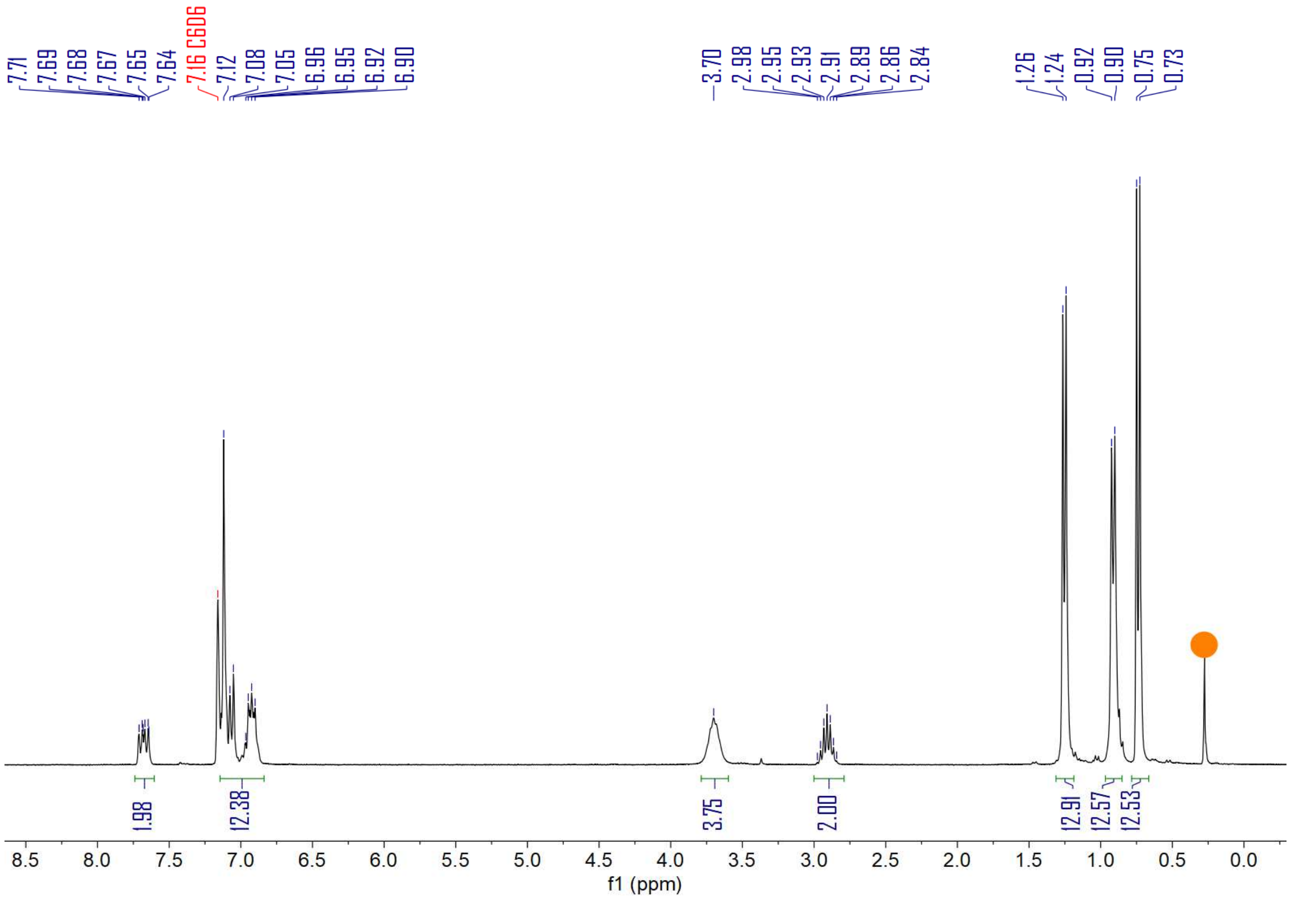

Figure S37. ^{1}H-NMR spectrum (300.0 MHz, C_6D_6, 338 K) of TipGe **5**. The orange circle denotes the resonance of silicone grease.

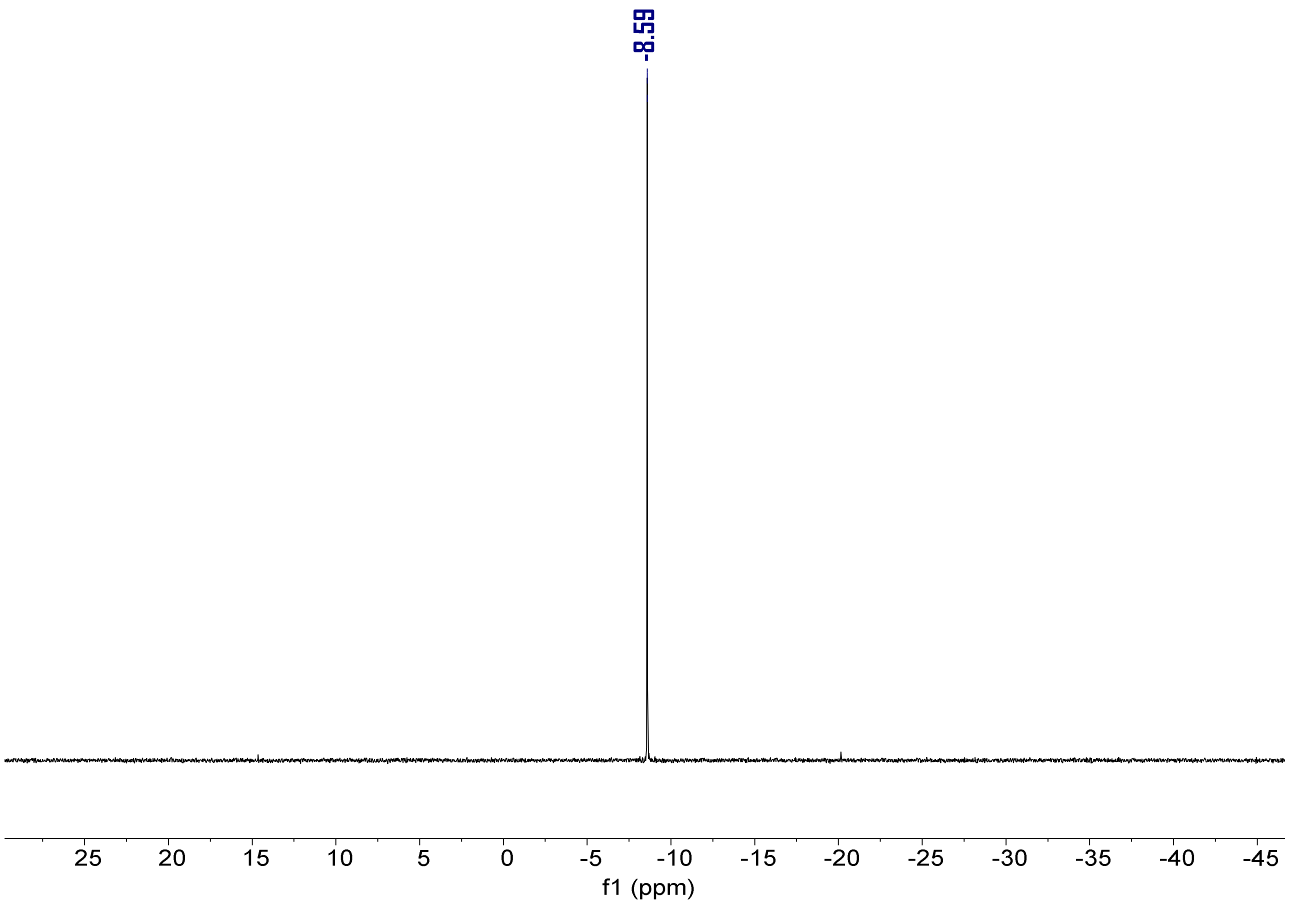

Figure S38. $^{31}P\{^{1}H\}$ NMR spectrum (202.4 MHz, C_6D_6, 298 K) of TipGe **5**.

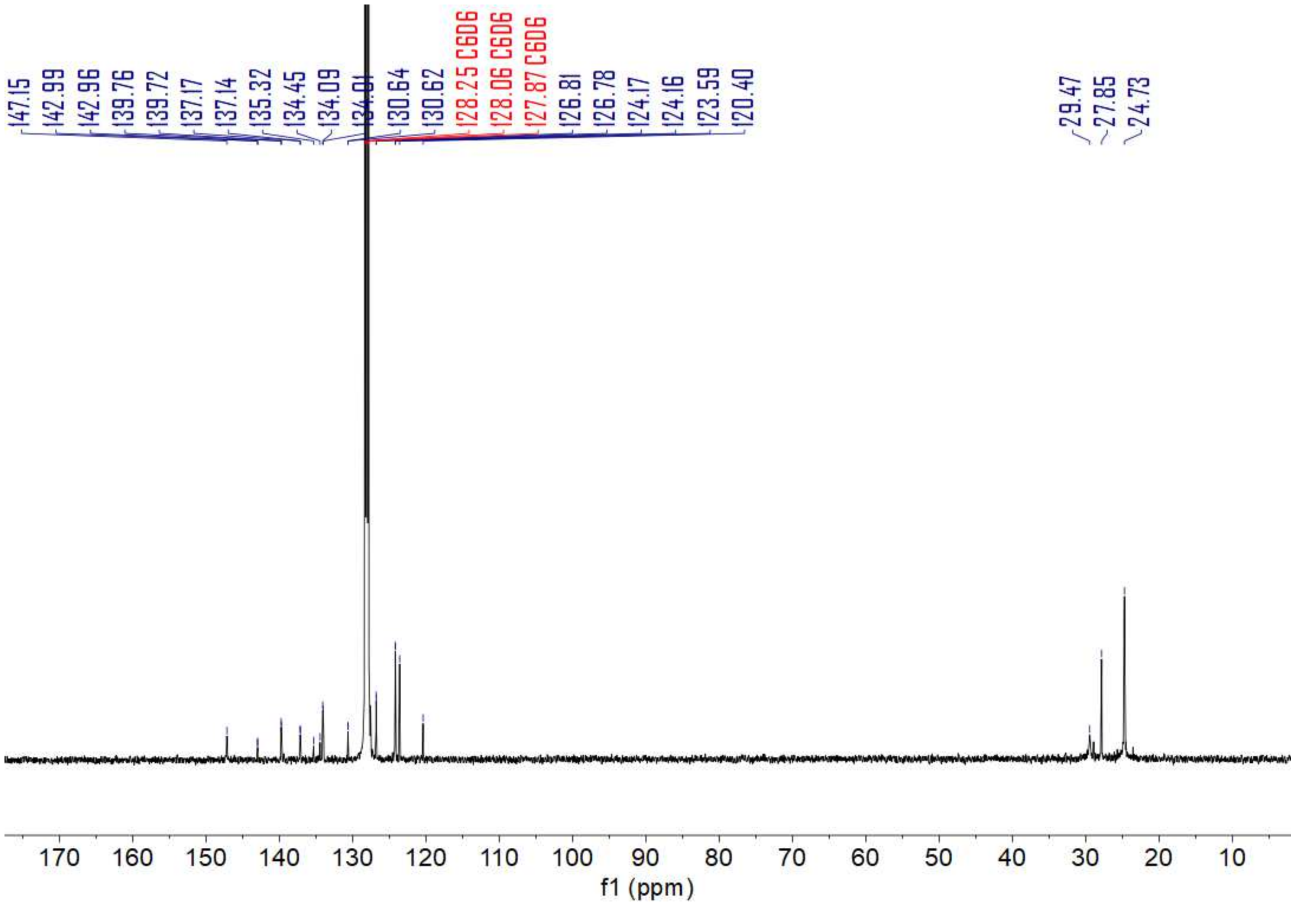

Figure S39. $^{13}C\{^{1}H\}$ NMR spectrum (125.7 MHz, C_6D_6, 298 K) of TipGe **5**.

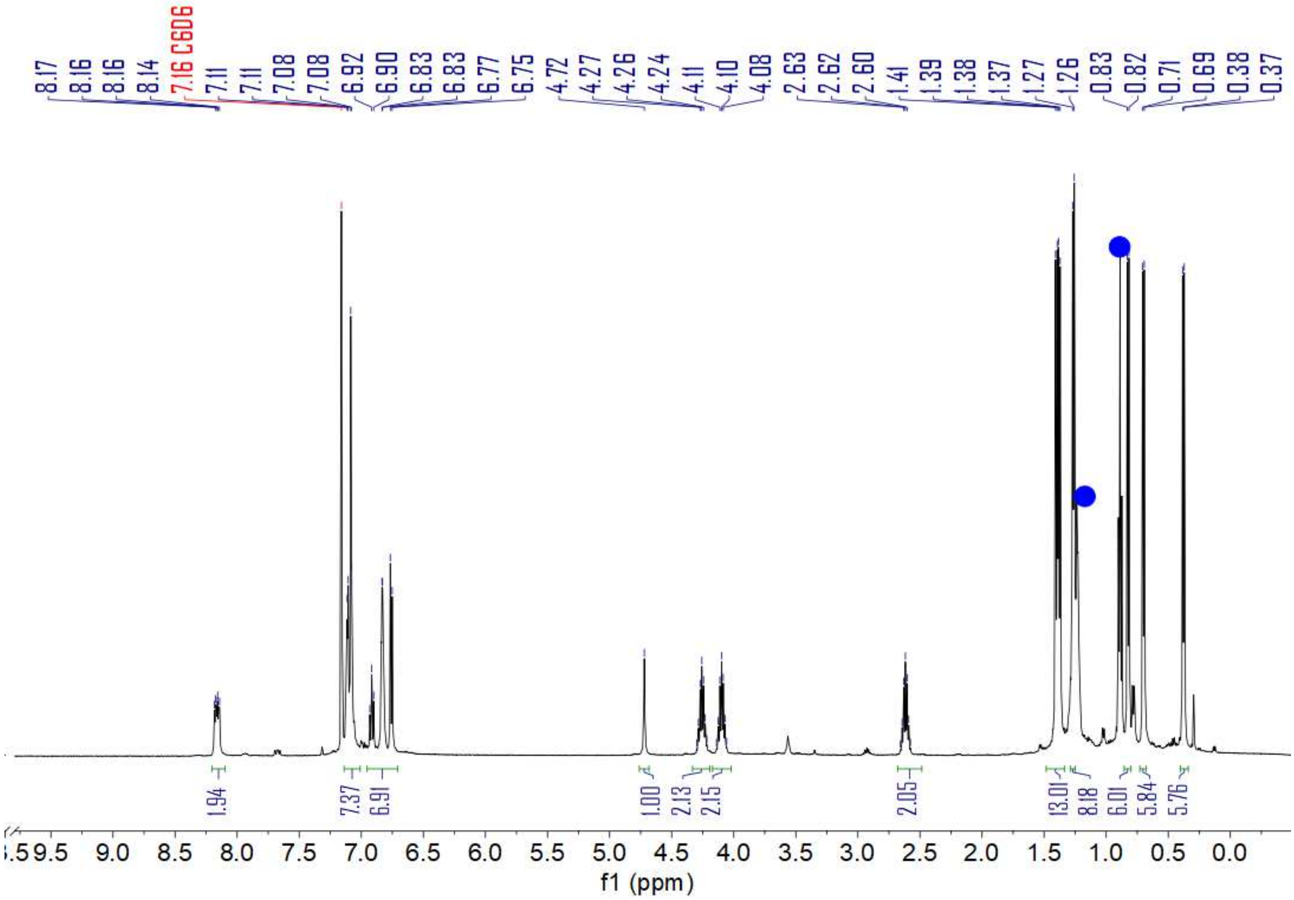

Figure S40. ^{1}H-NMR spectrum (499.9 MHz, C_6D_6, 298 K) of [(TipH)GeCl]. The sample was isolated as a side-product during the salt metathesis reaction of [$TipLi_2$] **3** with germanium(II) chloride-dioxane adduct. The blue circles denote the chemical resonances of residual *n*-hexane.

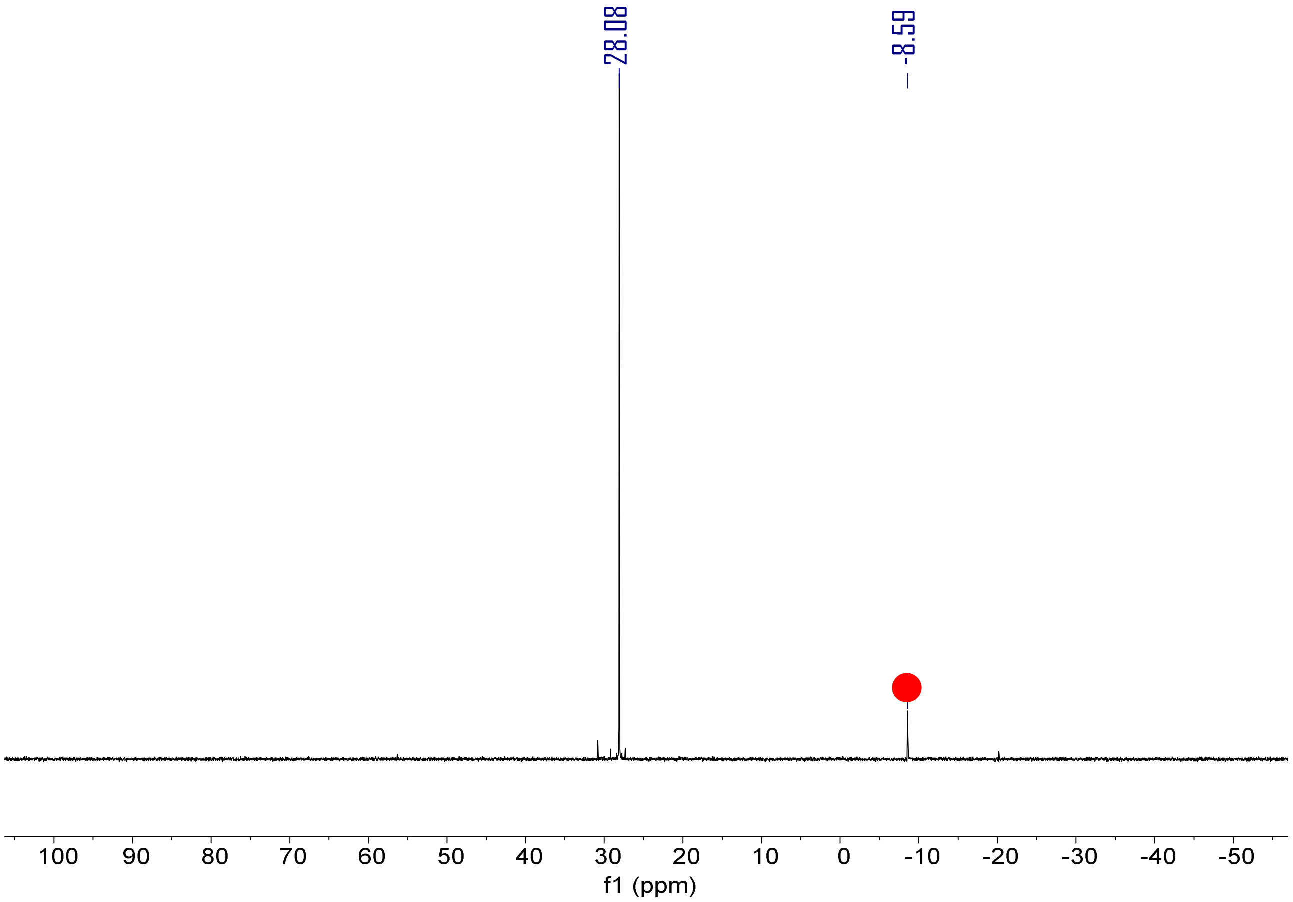

Figure S41. $^{31}P\{^{1}H\}$ NMR spectrum (202.4 MHz, C_6D_6, 298 K) of (TipH)GeCl. The sample was isolated as a side-product during the salt metathesis reaction of [TipLi$_2$] **3** with germanium(II) chloride-dioxane adduct. The red circle denotes the chemical resonance of TipGe **5**.

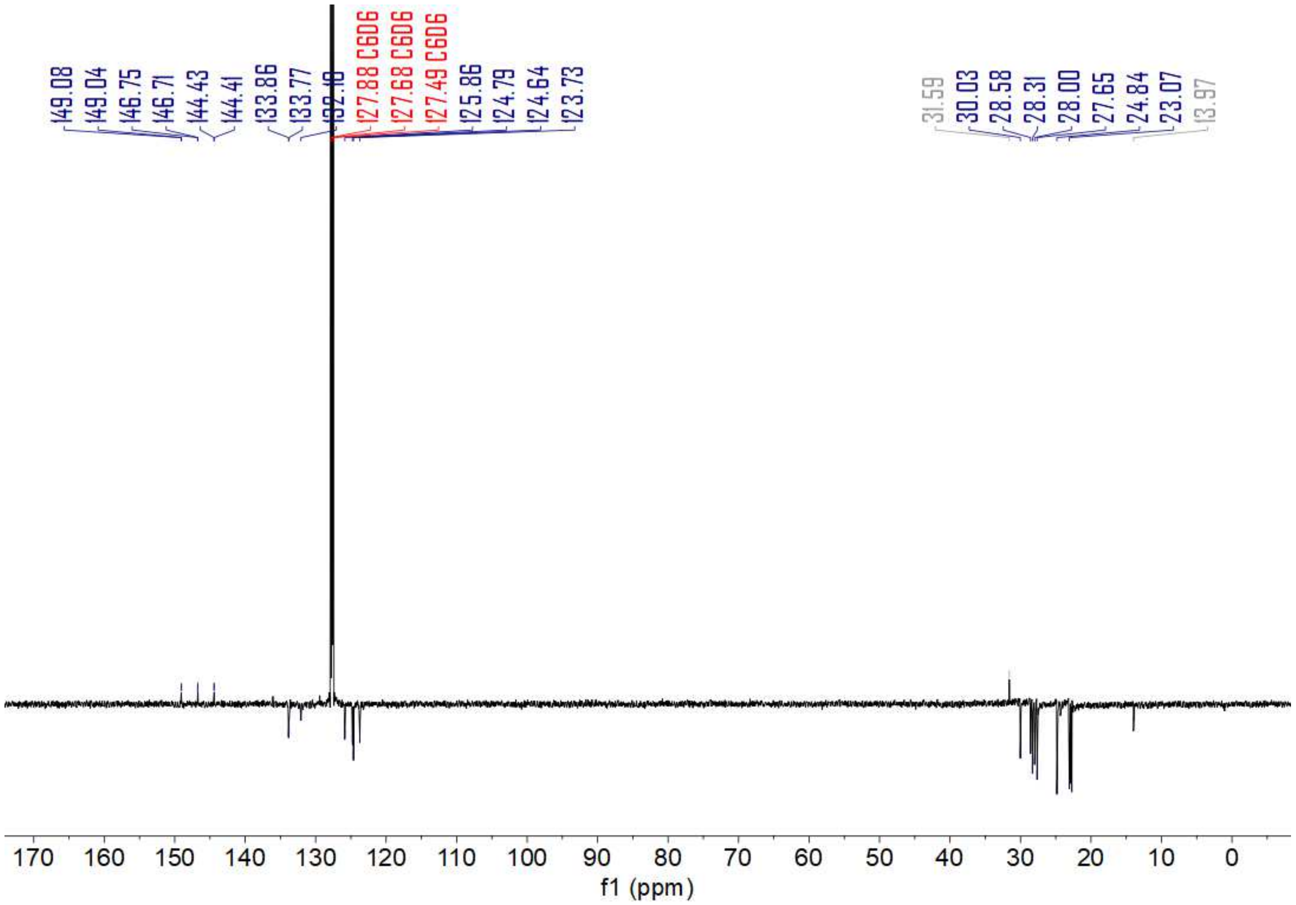

Figure S42. $^{13}C\{^{1}H\}$ DEPT NMR spectrum (125.7 MHz, C_6D_6, 298 K) of (TipH)GeCl. The sample was isolated as a side-product during the salt metathesis reaction of [TipLi$_2$] **3** with germanium(II) chloride-dioxane adduct. The grey-labelled peaks denote the chemical resonances of residual *n*-hexane.

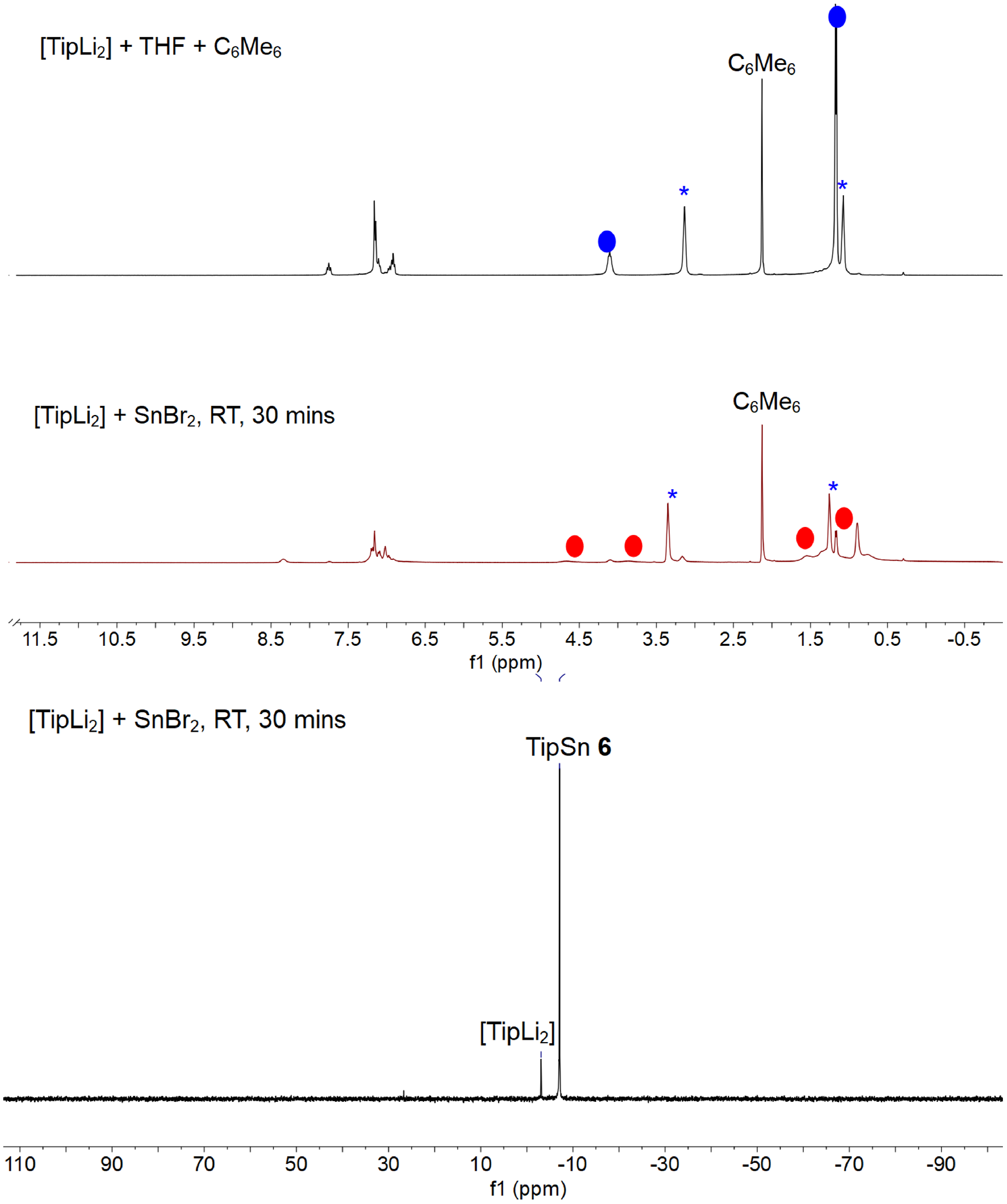

Figure S43. ^{1}H-NMR (400.1 MHz, C_6D_6, 300 K) and $^{31}P\{^{1}H\}$ NMR spectrum (121.5 MHz, C_6D_6, 300 K) of an *in-situ* reaction between [$TipLi_2$] **3** (6.5 mg, 10.1 µmol, 1 equiv.) and $SnBr_2$ (2.80 mg, 10.1 µmol, 1 equiv.) in C_6D_6 (0.6 mL) and THF (ca. 20 µL), hexamethylbenzene (1.5 mg, 9.24 µmol, 1 equiv.) was added as an internal standard. The formation of TipSn **6** was observed at ambient temperature for 20 minutes. The blue circles denote the resonances of [$TipLi_2$] **3**, and the red circles denote the resonances of TipSn **6**. The blue asterisks denote resonances of THF. The *in-situ* yield could not be determined due to the broadness of NMR resonances.

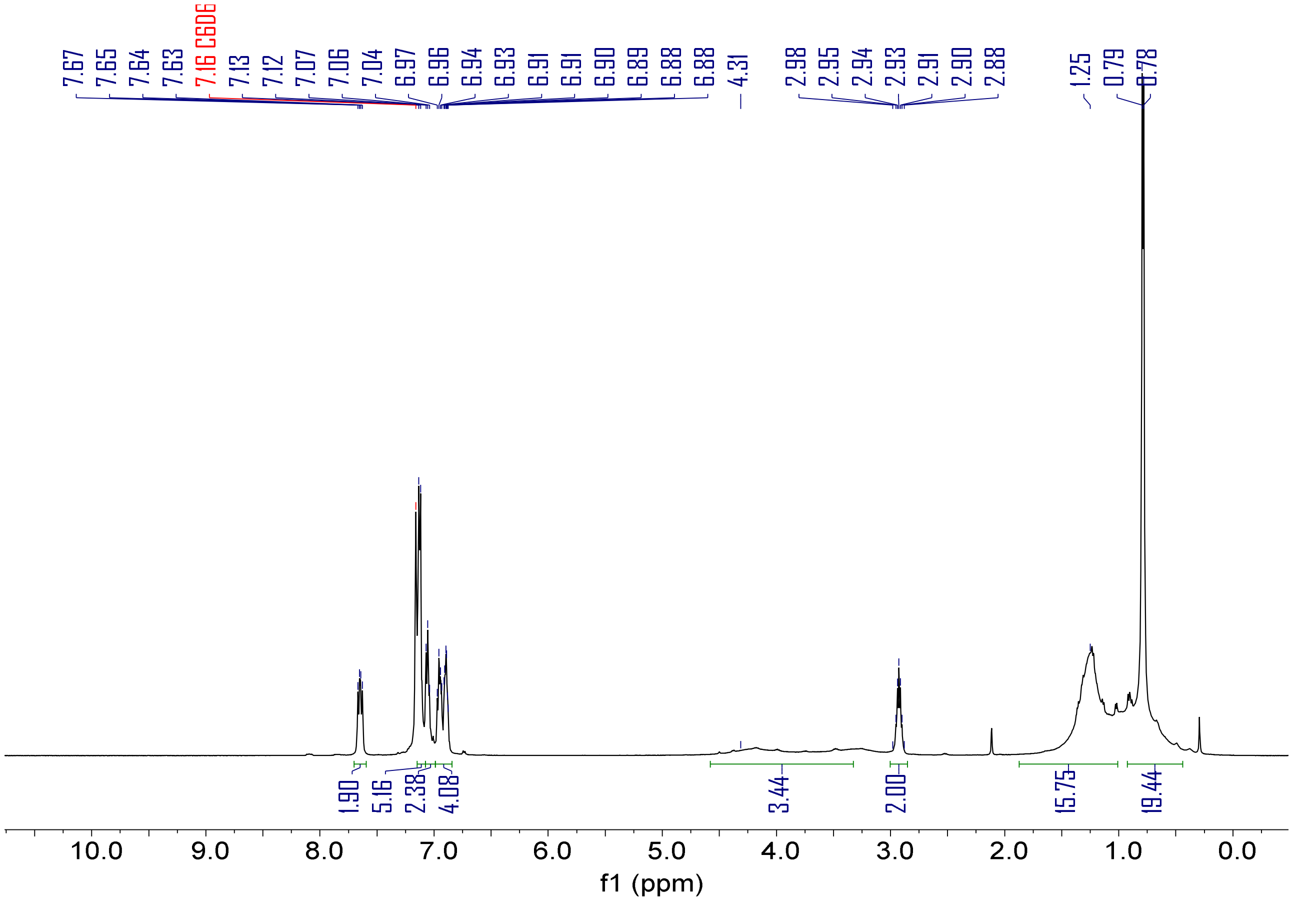

Figure S44. ^{1}H-NMR spectrum (499.9 MHz, C_6D_6, 298 K) of TipSn **6** at ambient temperature, showing some very broad resonances.

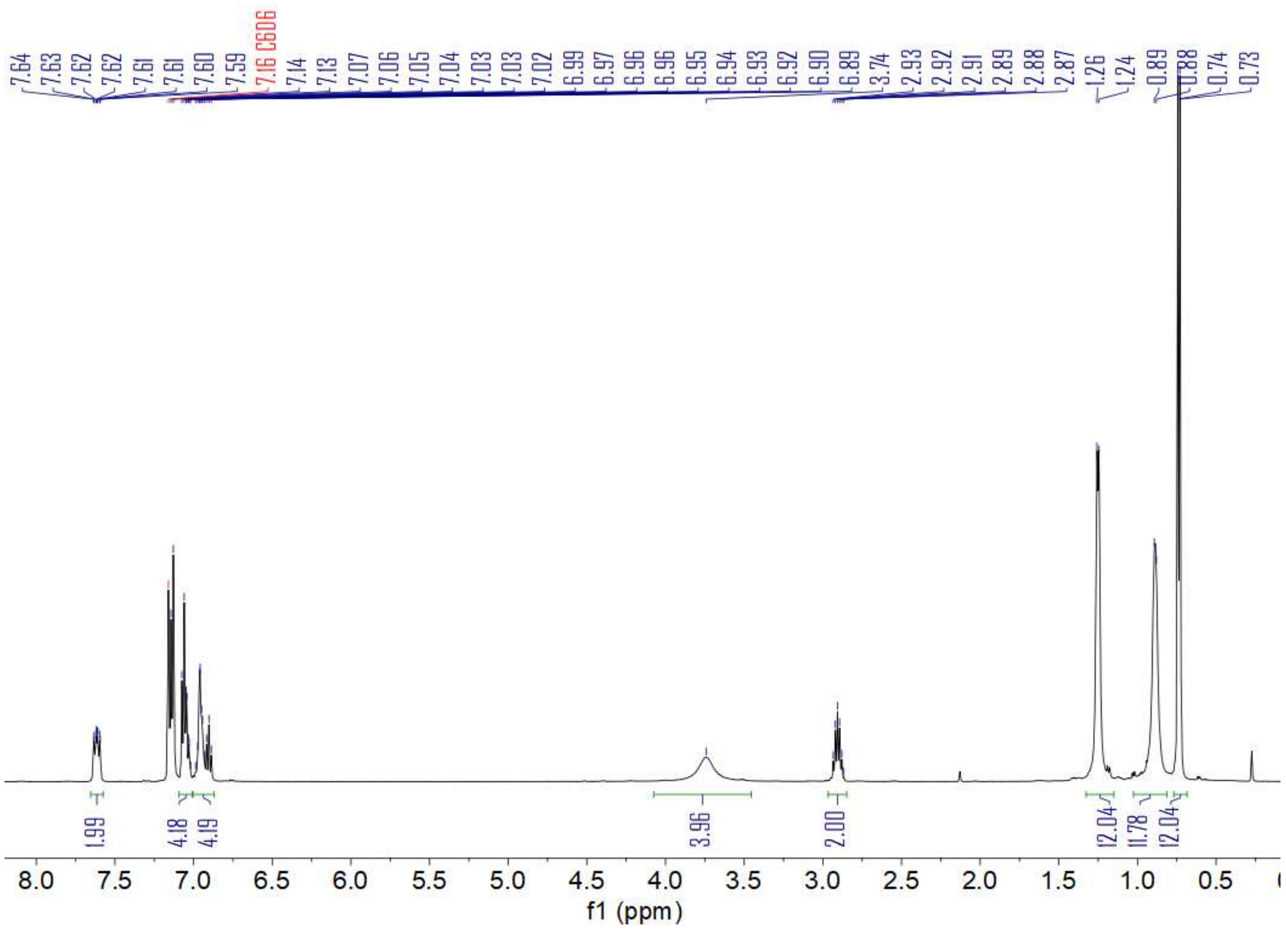

Figure S45. ^{1}H-NMR spectrum (499.9 MHz, C_6D_6, 343 K) of TipSn **6**.

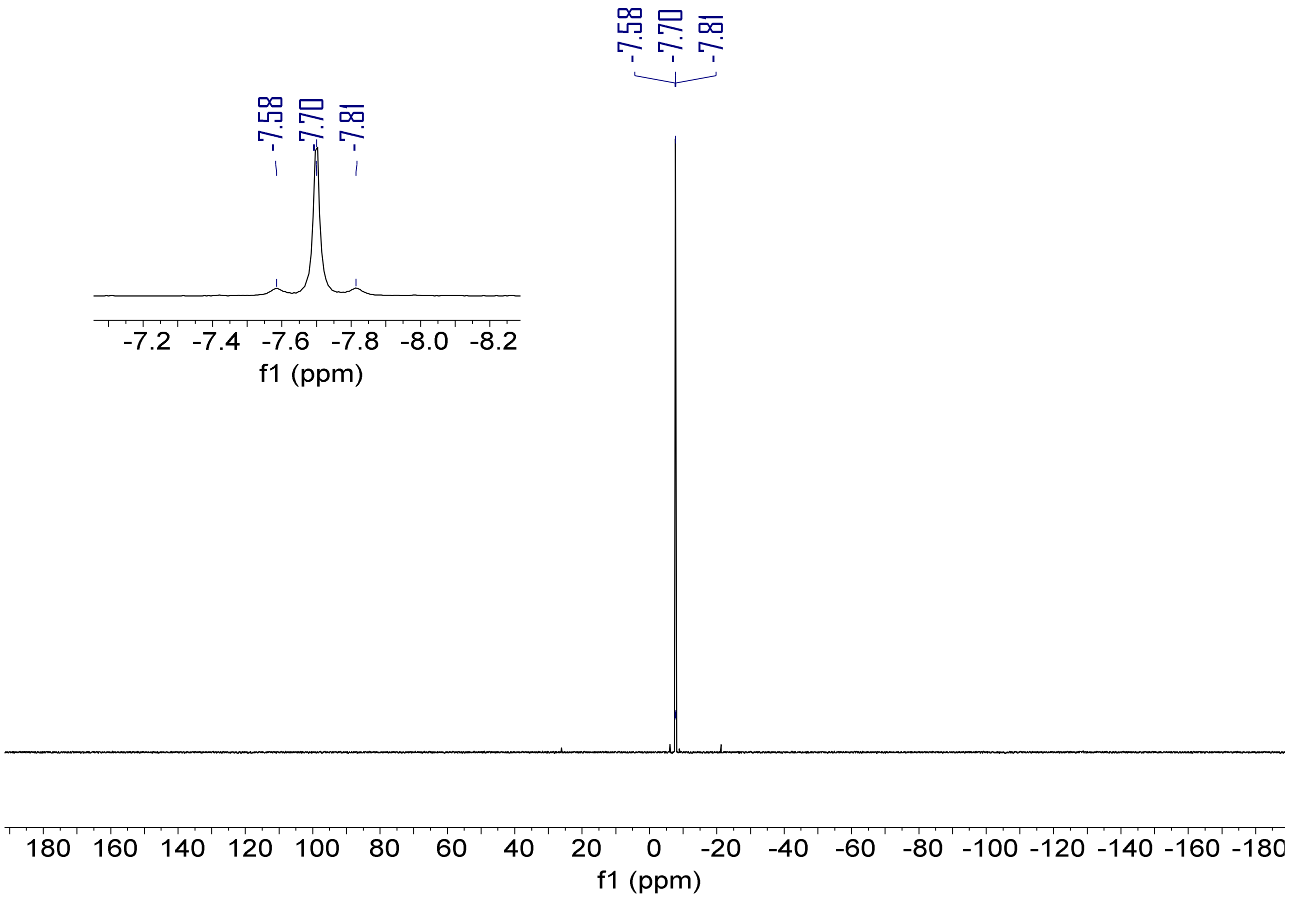

Figure S46. $^{31}P\{^{1}H\}$ NMR spectrum (202.4 MHz, C_6D_6, 298 K) of TipSn **6**.

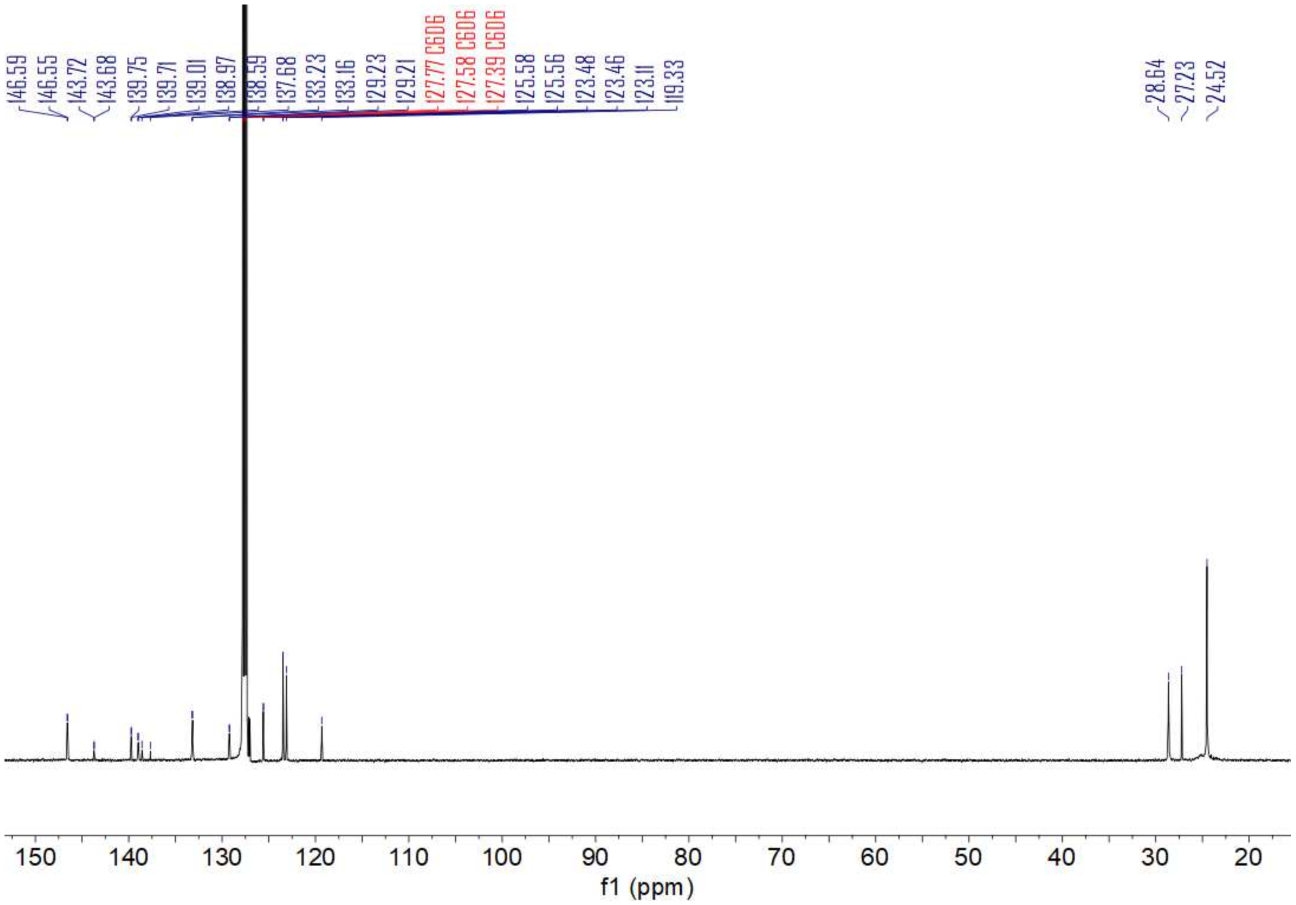

Figure S47. $^{13}C\{^{1}H\}$ NMR spectrum (125.7 MHz, C_6D_6, 343 K) of TipSn **6**.

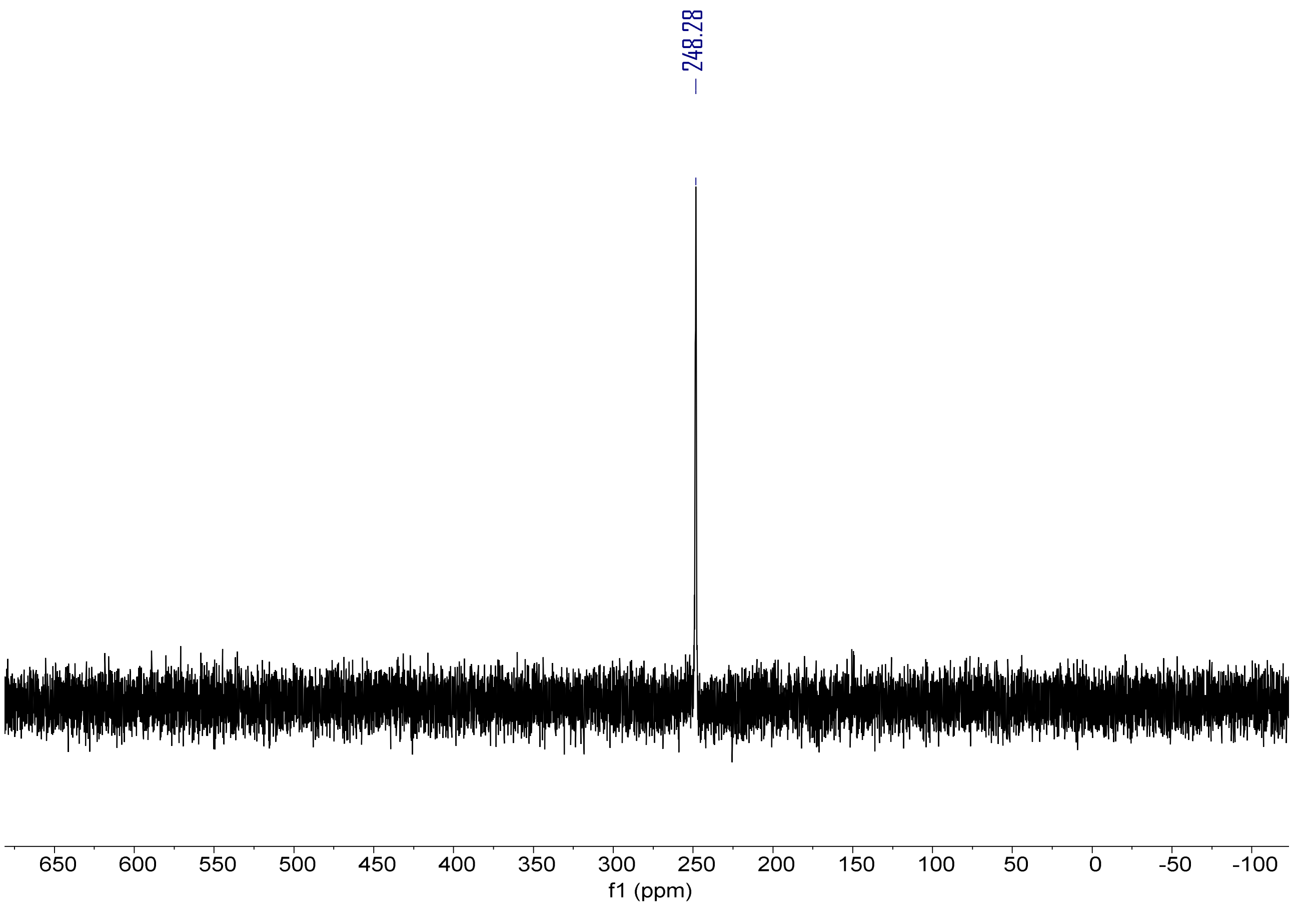

Figure S48. $^{119}Sn\{^{1}H\}$ NMR spectrum (149.3 MHz, C_6D_6, 298 K) of TipSn **6**.

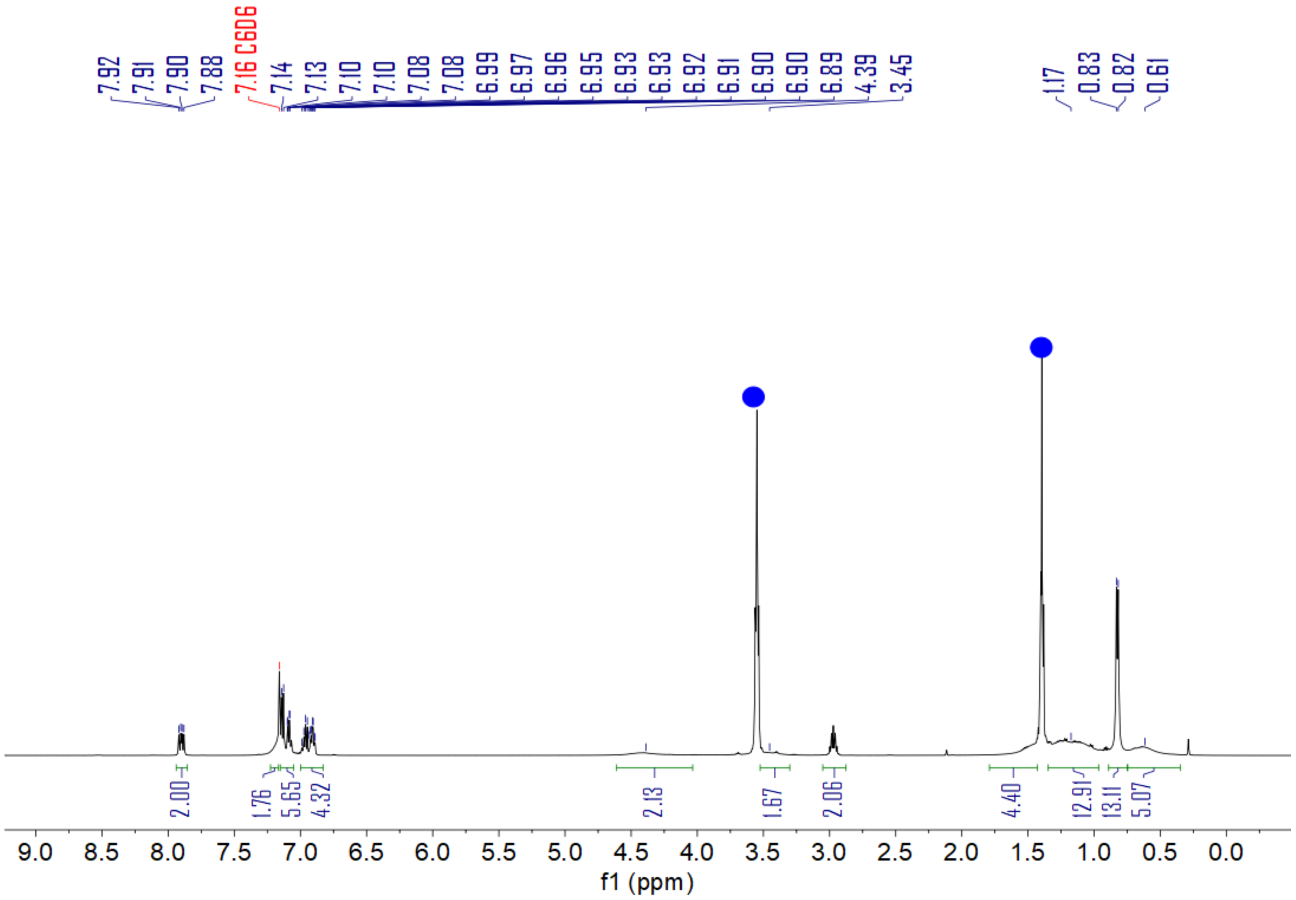

Figure S49. ^{1}H-NMR spectrum (499.9 MHz, C_6D_6, 298 K) of the *in-situ* reaction of TipSn **6** with THF, indicating the formation of the THF adduct TipSn(THF). The blue circles denote the resonances of free THF.

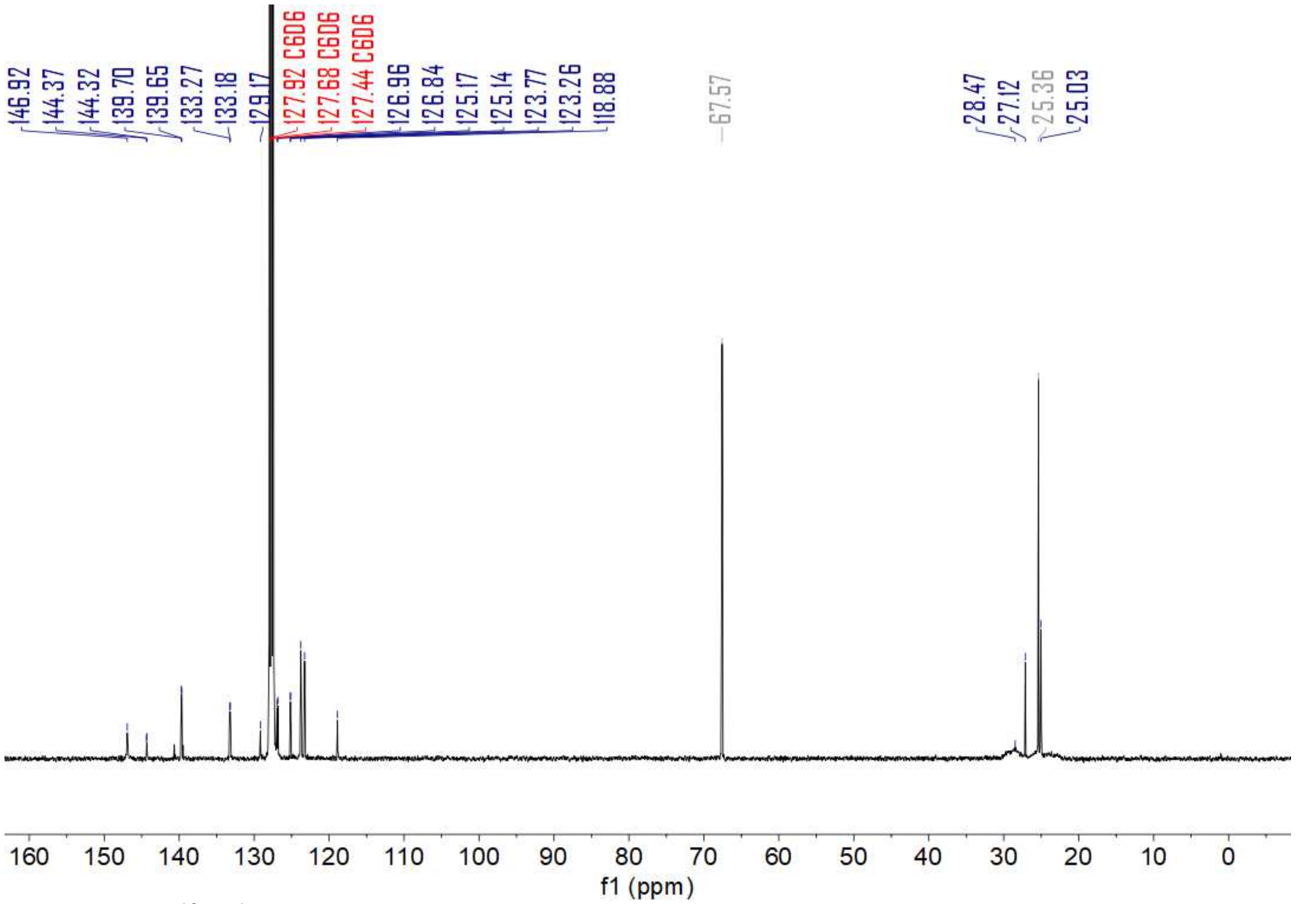

Figure S50. $^{13}C\{^{1}H\}$ NMR spectrum (100.6 MHz, C_6D_6, 298 K) of the *in-situ* reaction between TipSn **6** and THF, indicating the formation of the THF adduct TipSn(THF). The grey-labelled peaks denote the resonances of free THF.

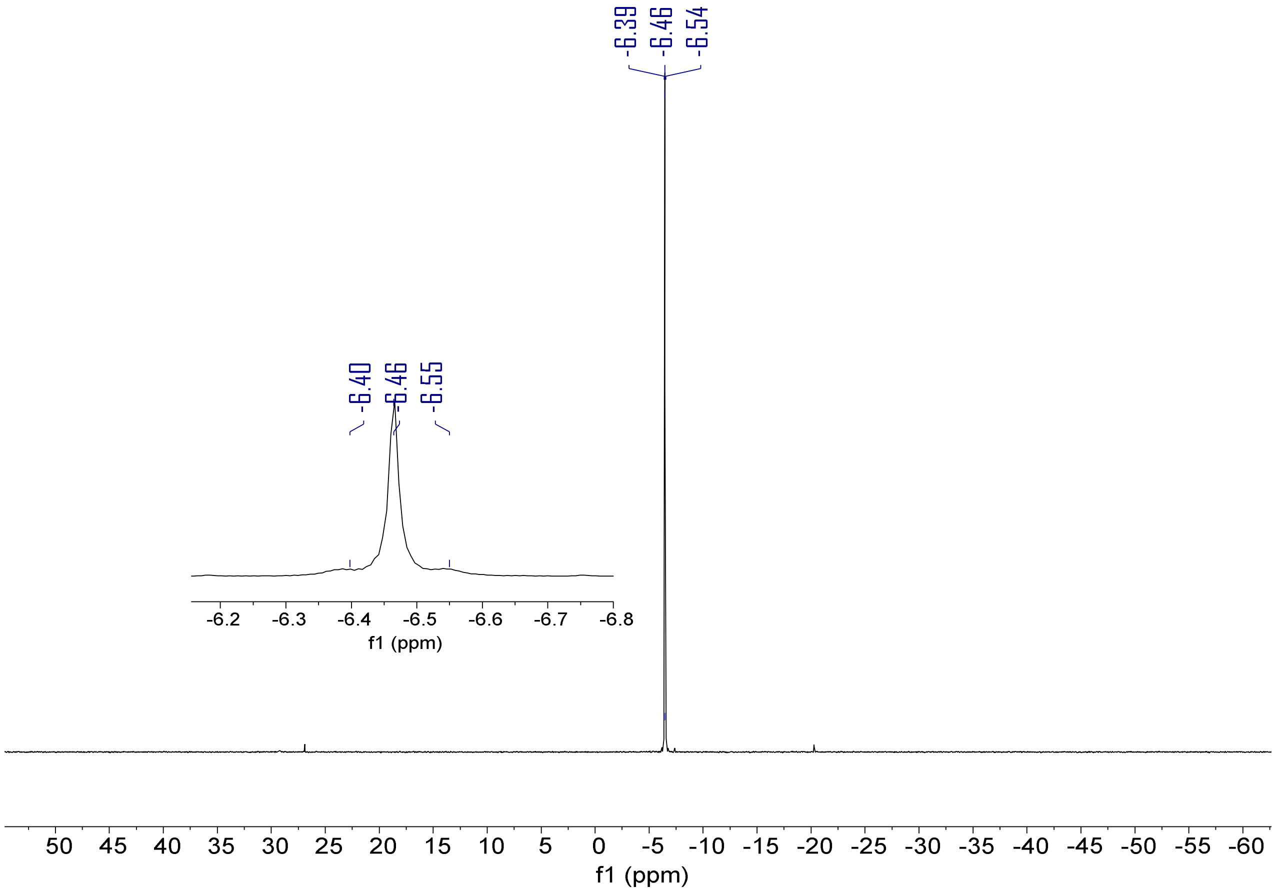

Figure S51. $^{31}P\{^{1}H\}$ NMR spectrum (202.4 MHz, C_6D_6, 298 K) of the *in-situ* reaction between TipSn **6** and THF, indicating the formation of the THF adduct TipSn(THF).

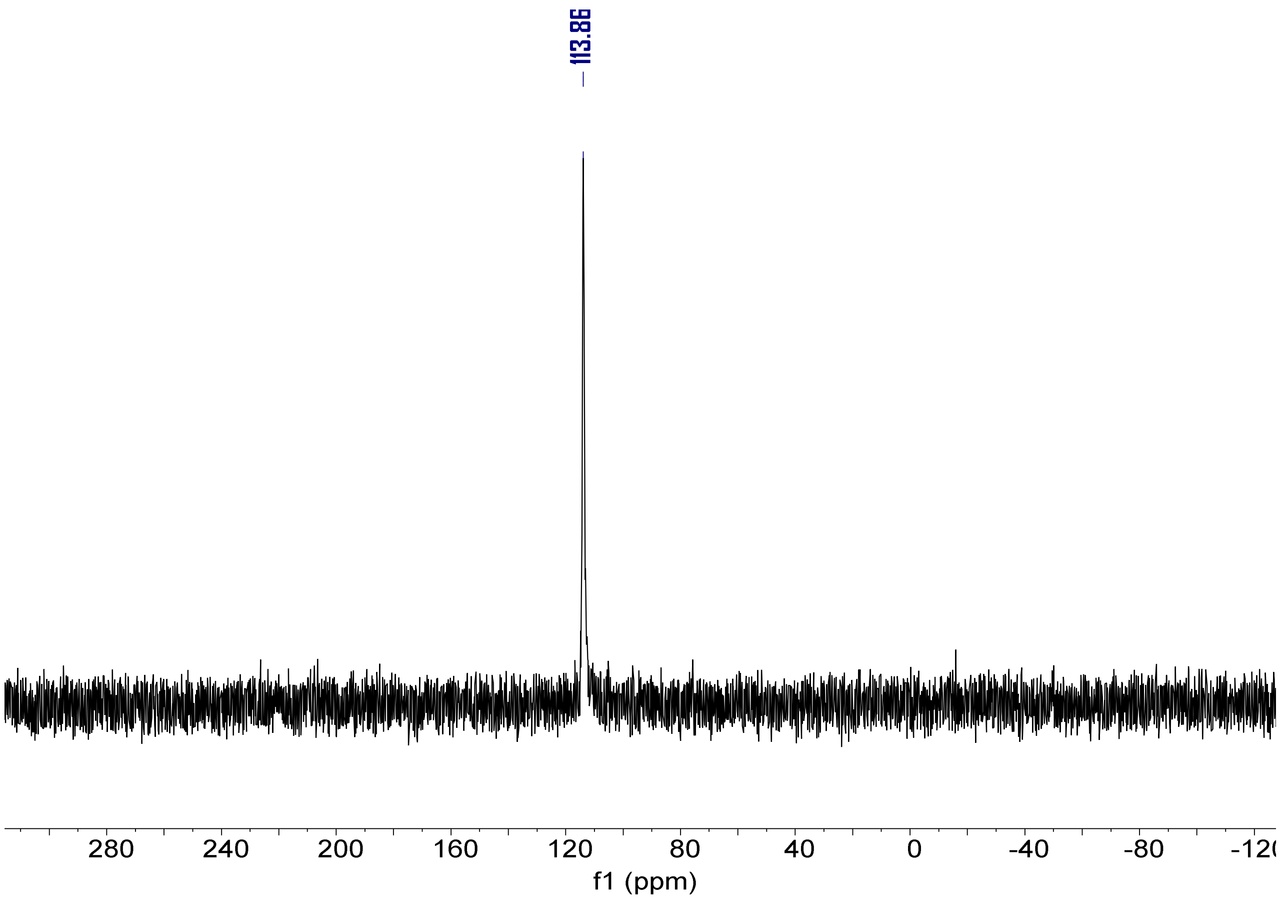

Figure S52. $^{119}Sn\{^{1}H\}$ NMR spectrum (149.3 MHz, C_6D_6, 298 K) of the *in-situ* reaction between TipSn **6** and THF, indicating the formation of the THF adduct TipSn(THF).

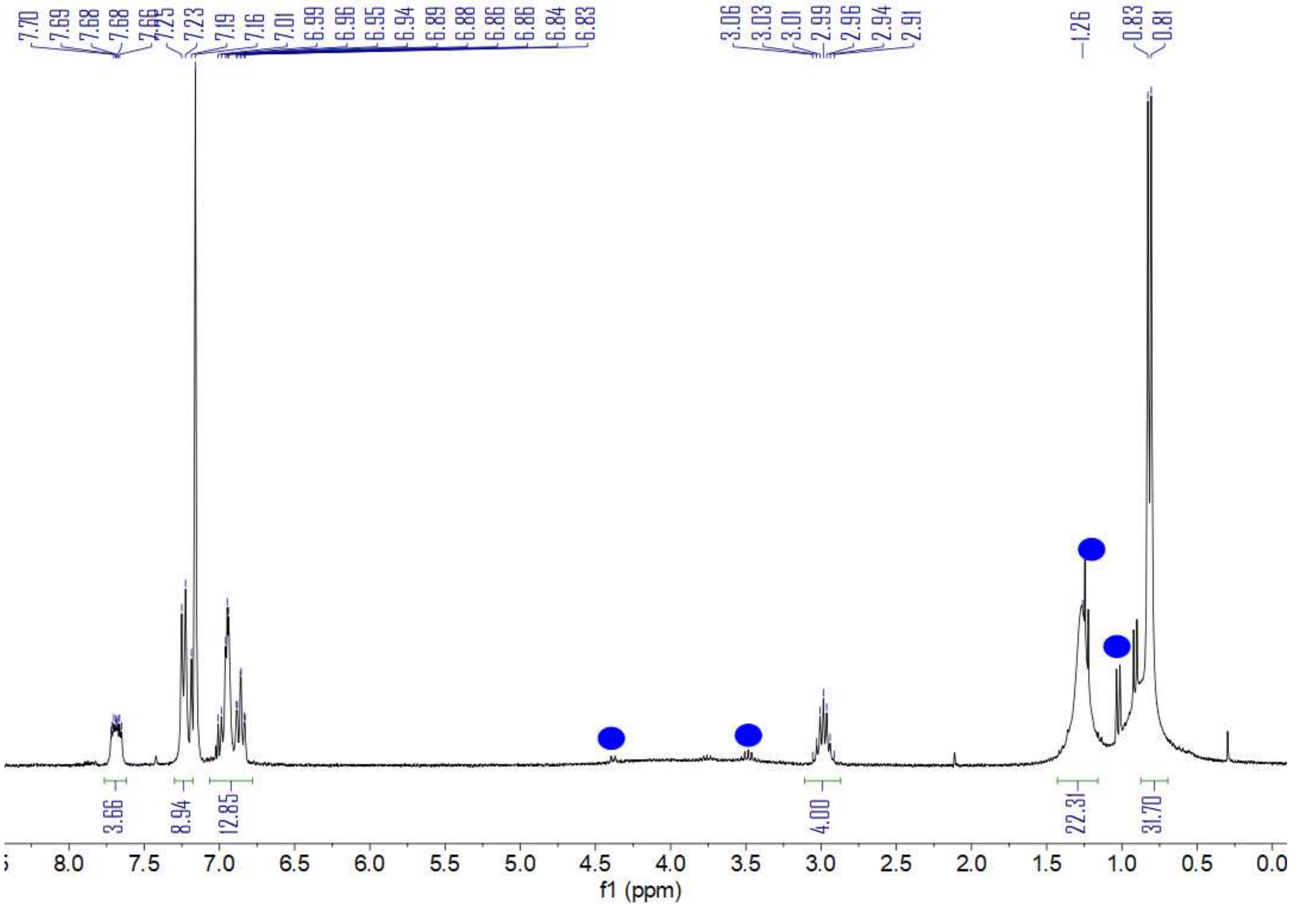

Figure S53. ^{1}H-NMR spectrum (300.0 MHz, C_6D_6, 298 K) of TipPb **7**. The blue circles denote the resonances of $TipH_2$ **2**.

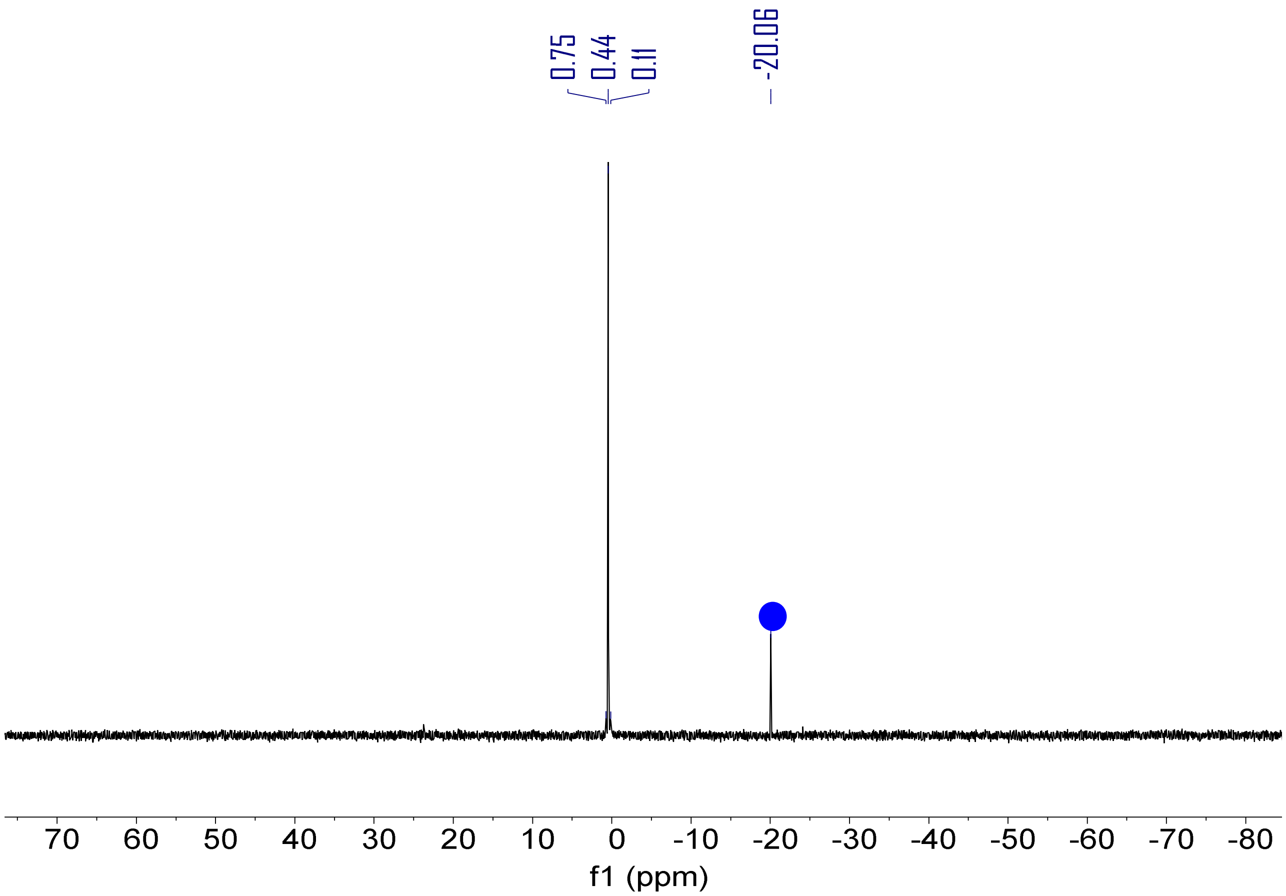

Figure S54. $^{31}P\{^1H\}$ NMR spectrum (162.1 MHz, C_6D_6, 300 K) of TipPb **7**. The blue circle denotes the chemical resonance of $TipH_2$ **2**.

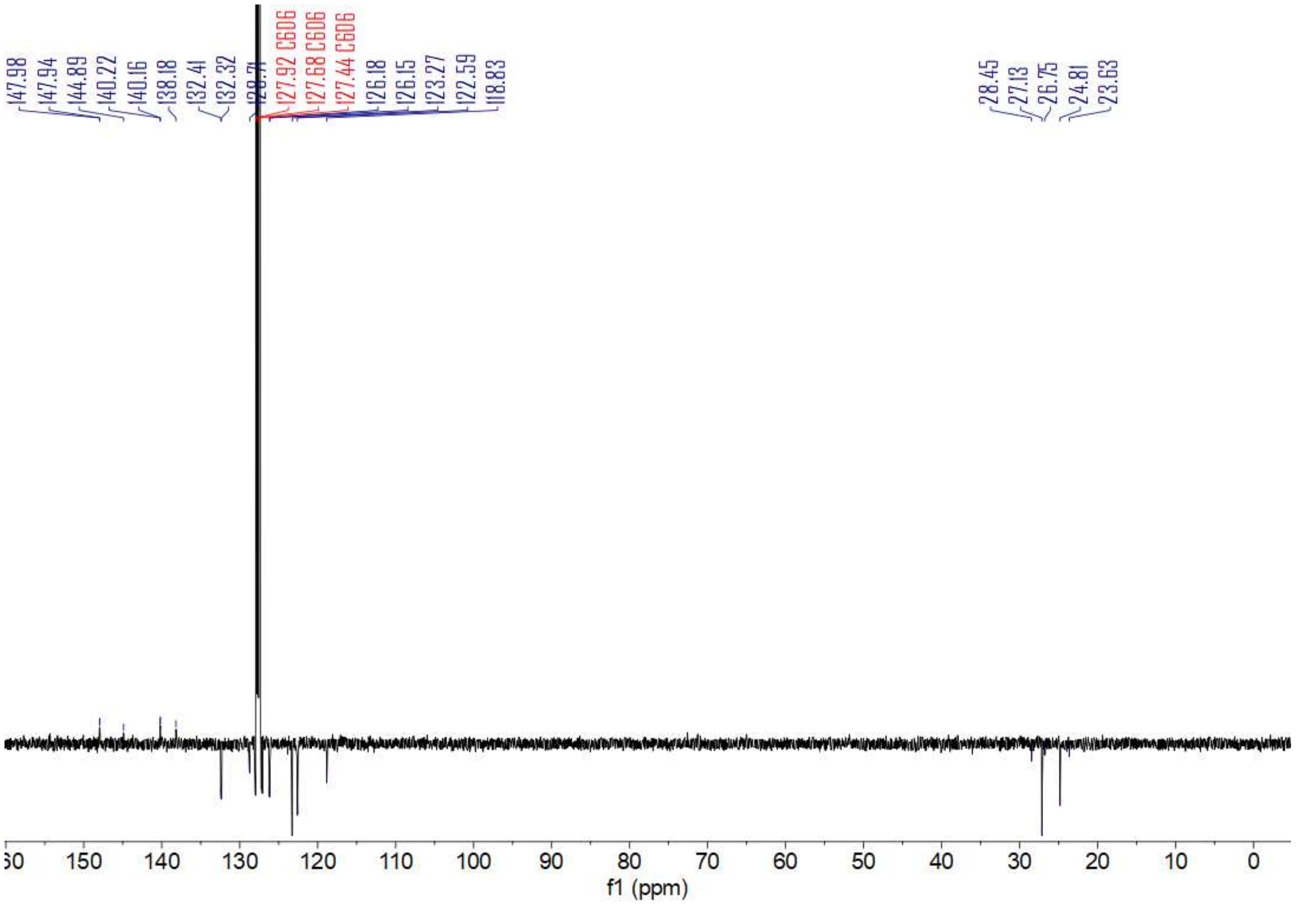

Figure S55. $^{13}C\{^{1}H\}$ DEPT NMR spectrum (101.0 MHz, C_6D_6, 300 K) of TipPb **7**.

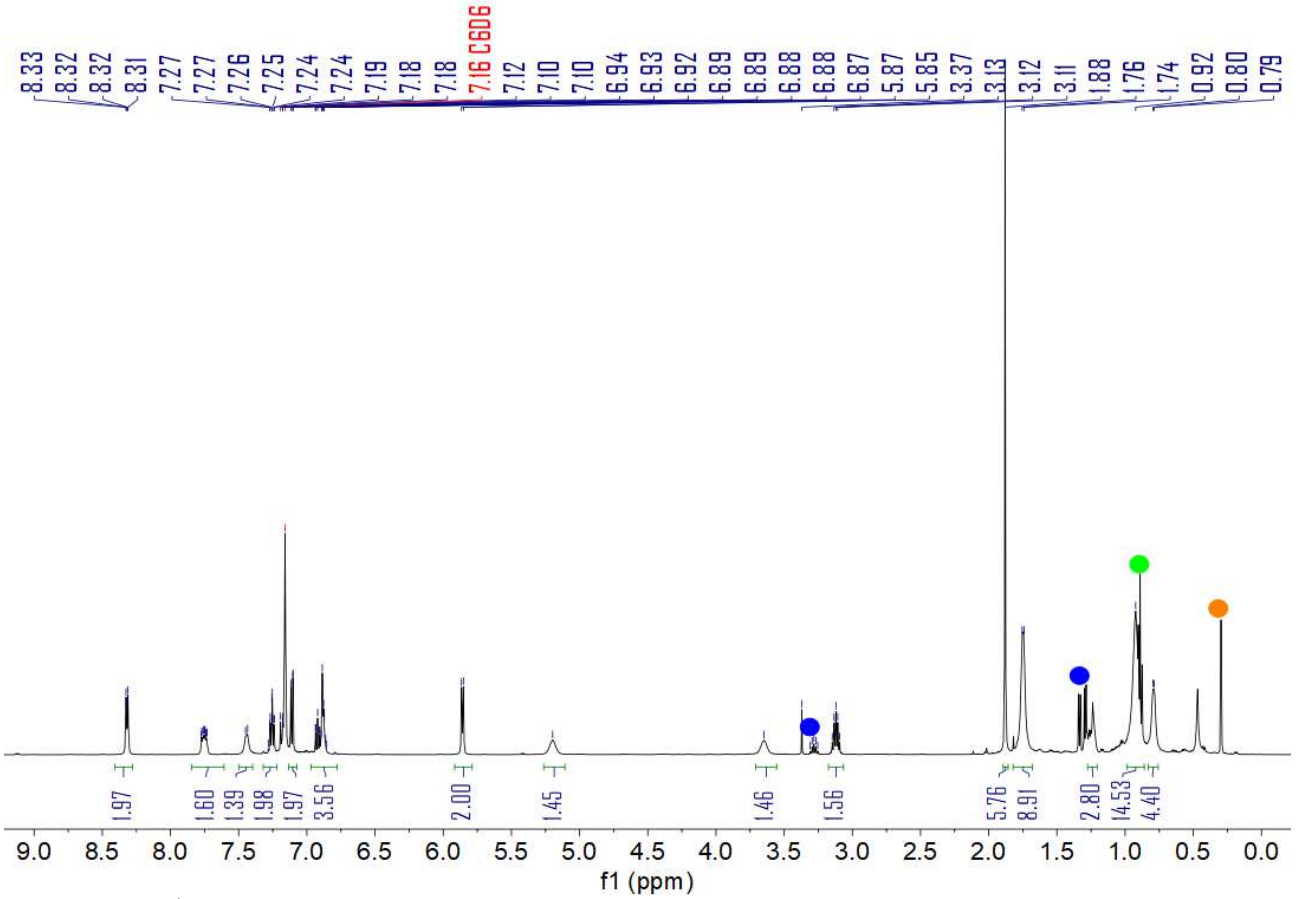

Figure S56. ^{1}H-NMR spectrum (500.1 MHz, C_6D_6, 298 K) of the *in-situ* reaction between TipSi **4** and DMAP, indicating the formation of the adduct TipSi(DMAP) **9**. The blue circles denote the resonances of free SIDip. The green circle denotes the resonance of *n*-hexane. The orange circle denotes the resonance of silicone grease.

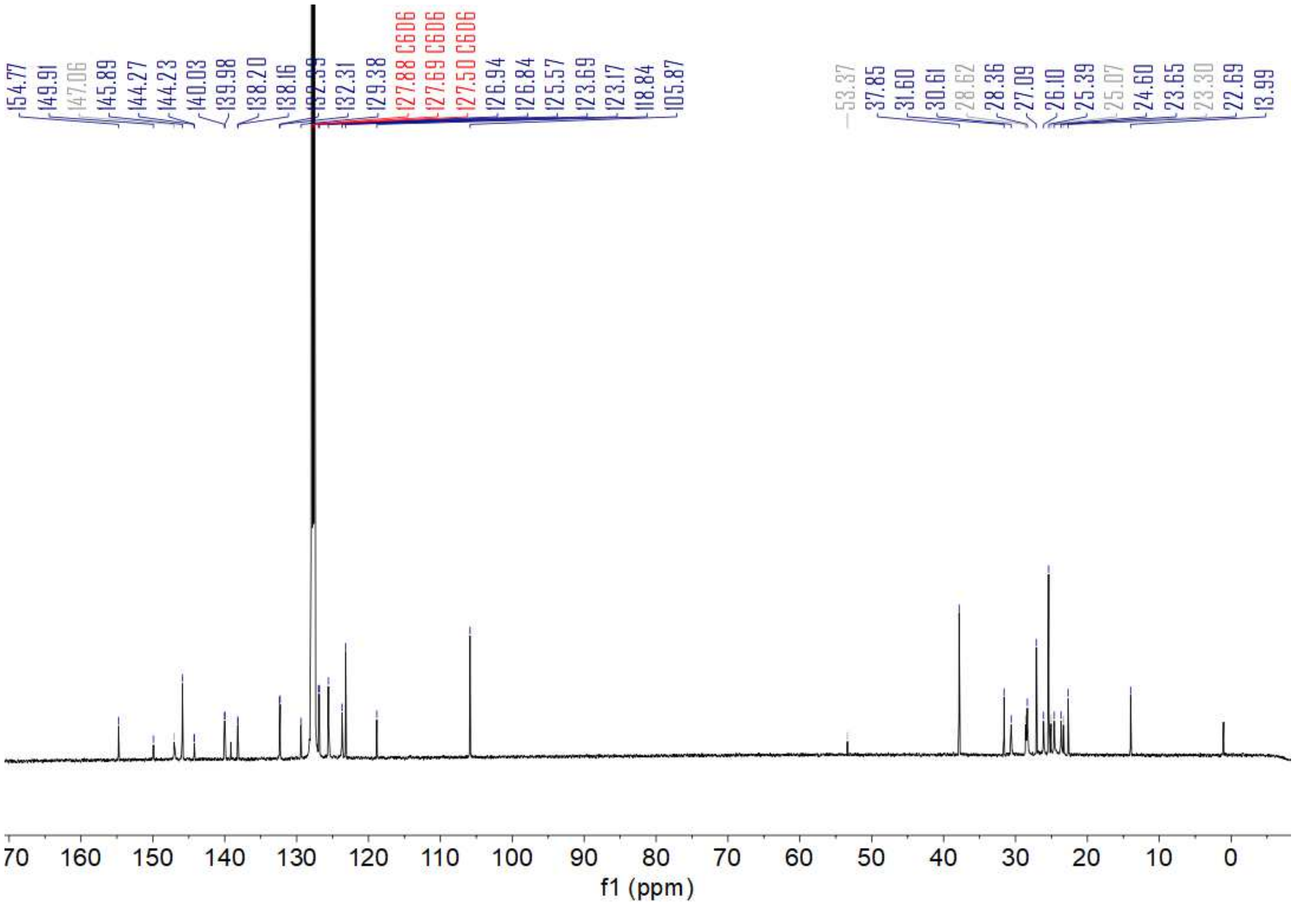

Figure S57. $^{13}C\{^{1}H\}$ NMR spectrum (125.8 MHz, C_6D_6, 298 K) of TipSi(DMAP) **9**, formed from the *in-situ* reaction between TipSi **4** and DMAP. The grey-labelled peaks correspond to the resonances of free SIDip.

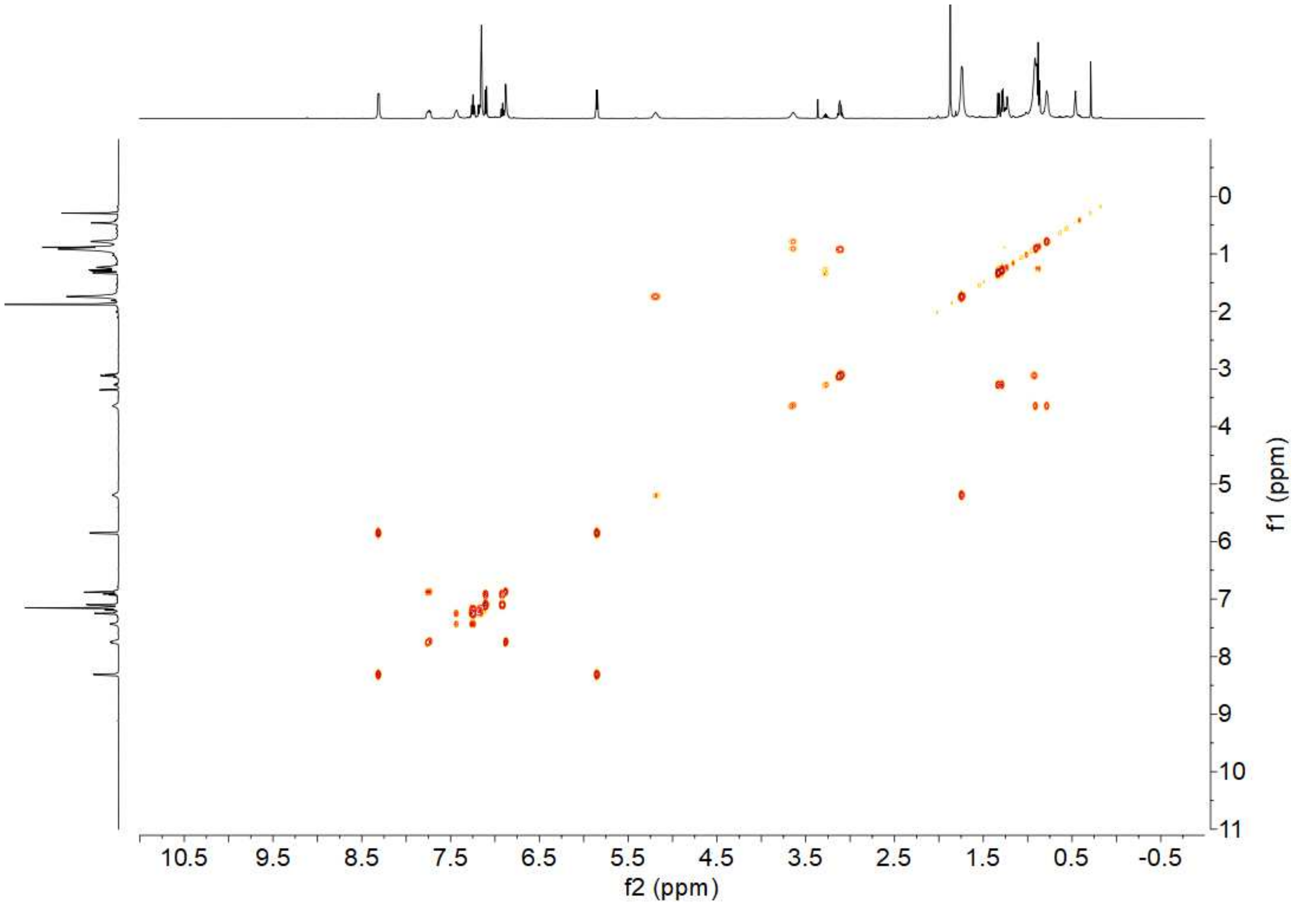

Figure S58. ^{1}H-^{1}H COSY NMR spectrum of TipSi(DMAP) adduct **9**, formed from the *in-situ* reaction between TipSi **4** and DMAP.

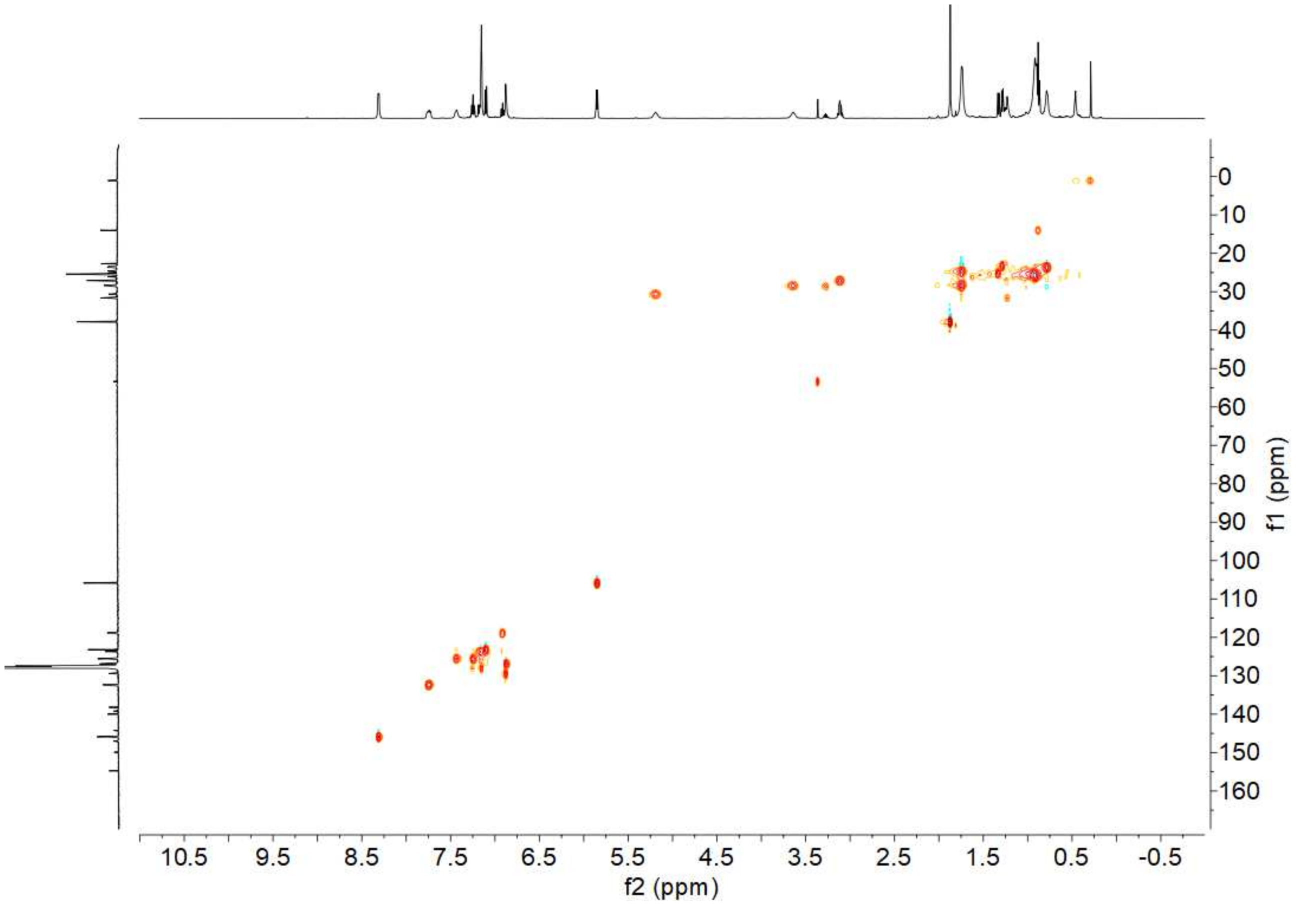

Figure S59. ^{1}H-^{13}C HSQC NMR spectrum TipSi(DMAP) adduct **9**, formed from the *in-situ* reaction between TipSi **4** and DMAP.

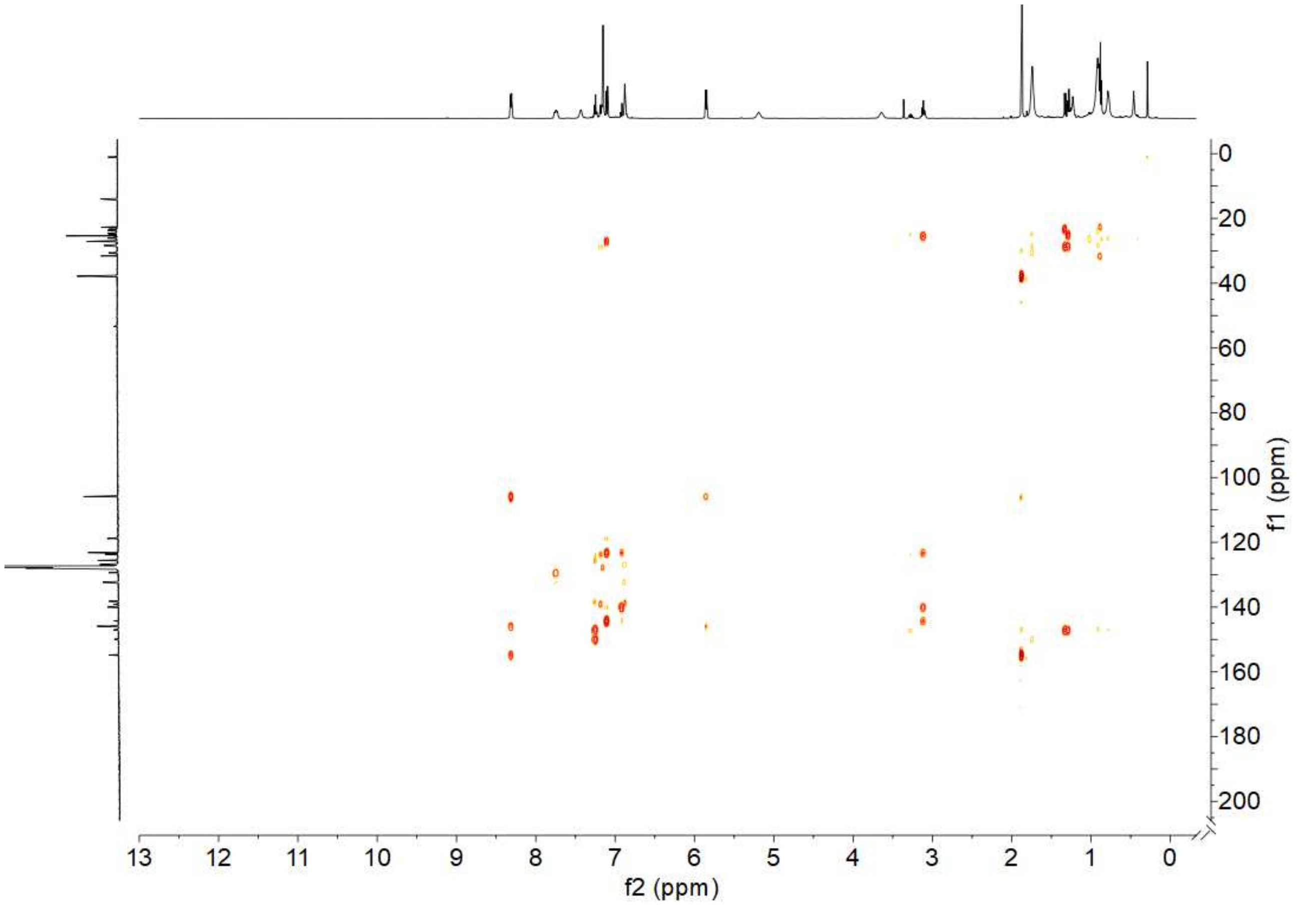

Figure S60. ^{1}H-^{13}C HMBC NMR spectrum of TipSi(DMAP) adduct **9**, formed from the *in-situ* reaction between TipSi **4** and DMAP.

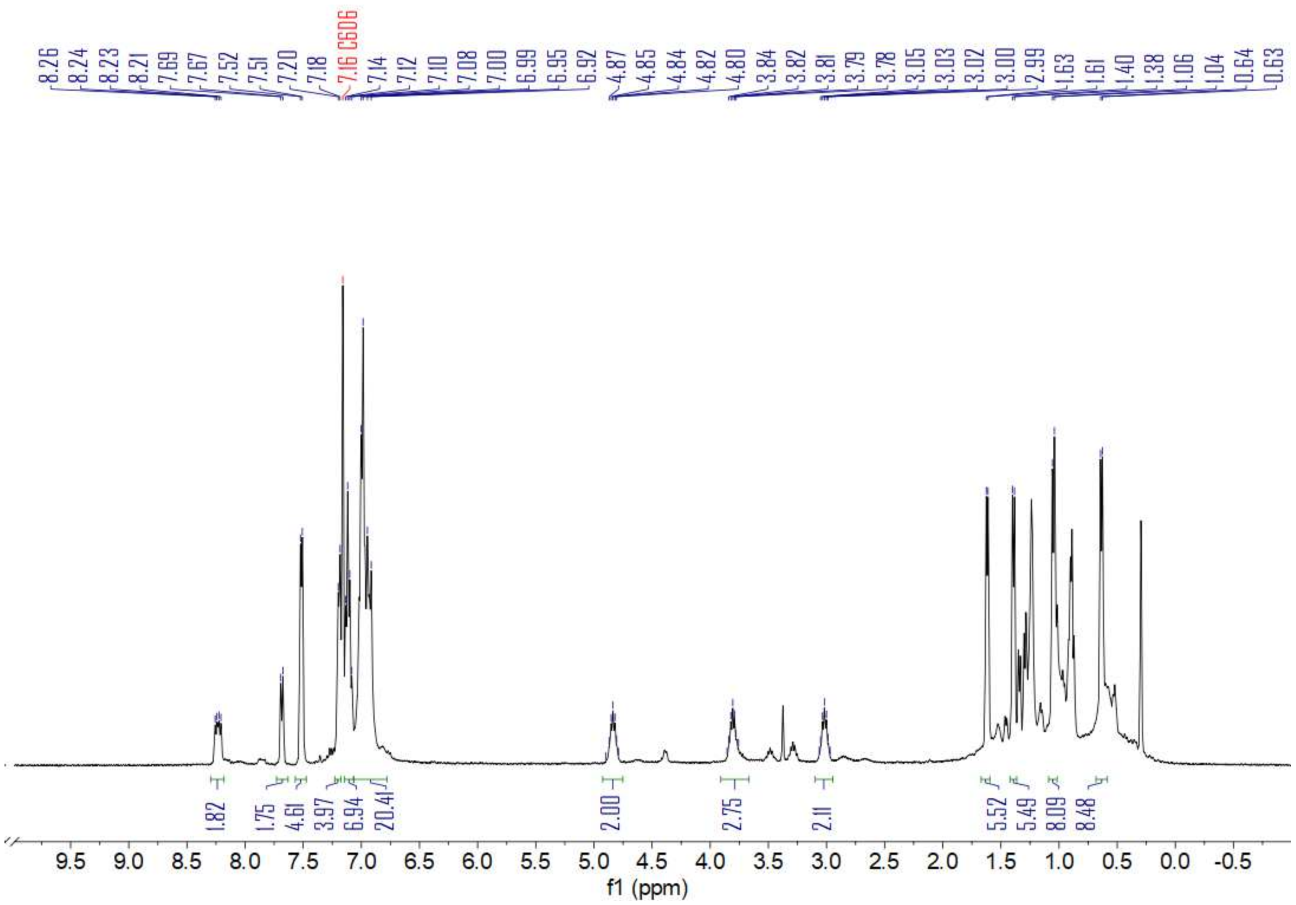

Figure S61. ^{1}H-NMR spectrum (400.1 MHz, C_6D_6, 298 K) of TipSi(CPh)$_2$ **10**, formed from the *in-situ* reaction between TipSi **4** and diphenylacetylene.

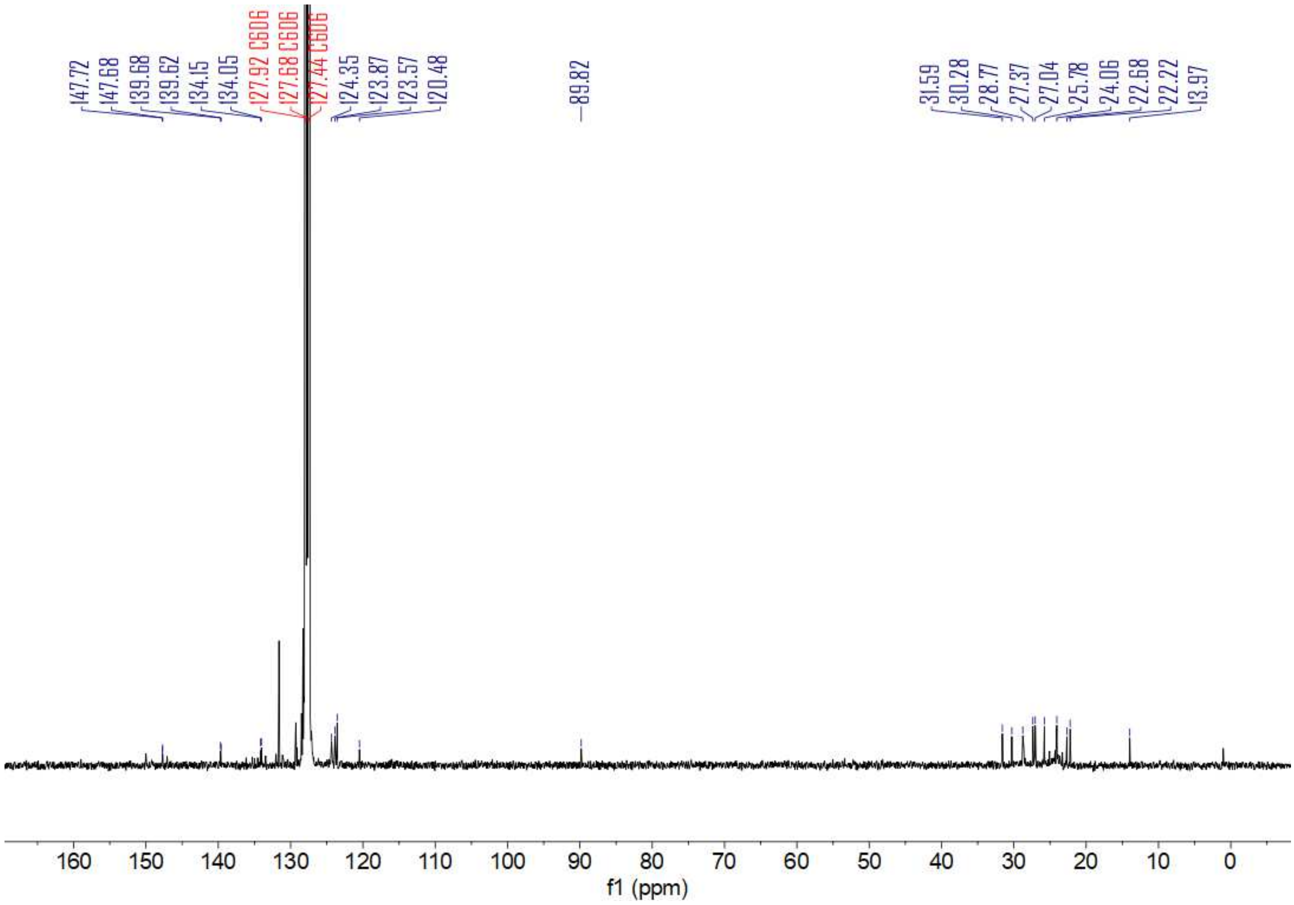

Figure S62. $^{13}C\{^{1}H\}$ NMR spectrum (100.6 MHz, C_6D_6, 298 K) of $TipSi(CPh)_2$ **10**, formed from the *in-situ* reaction between TipSi **4** and diphenylacetylene.

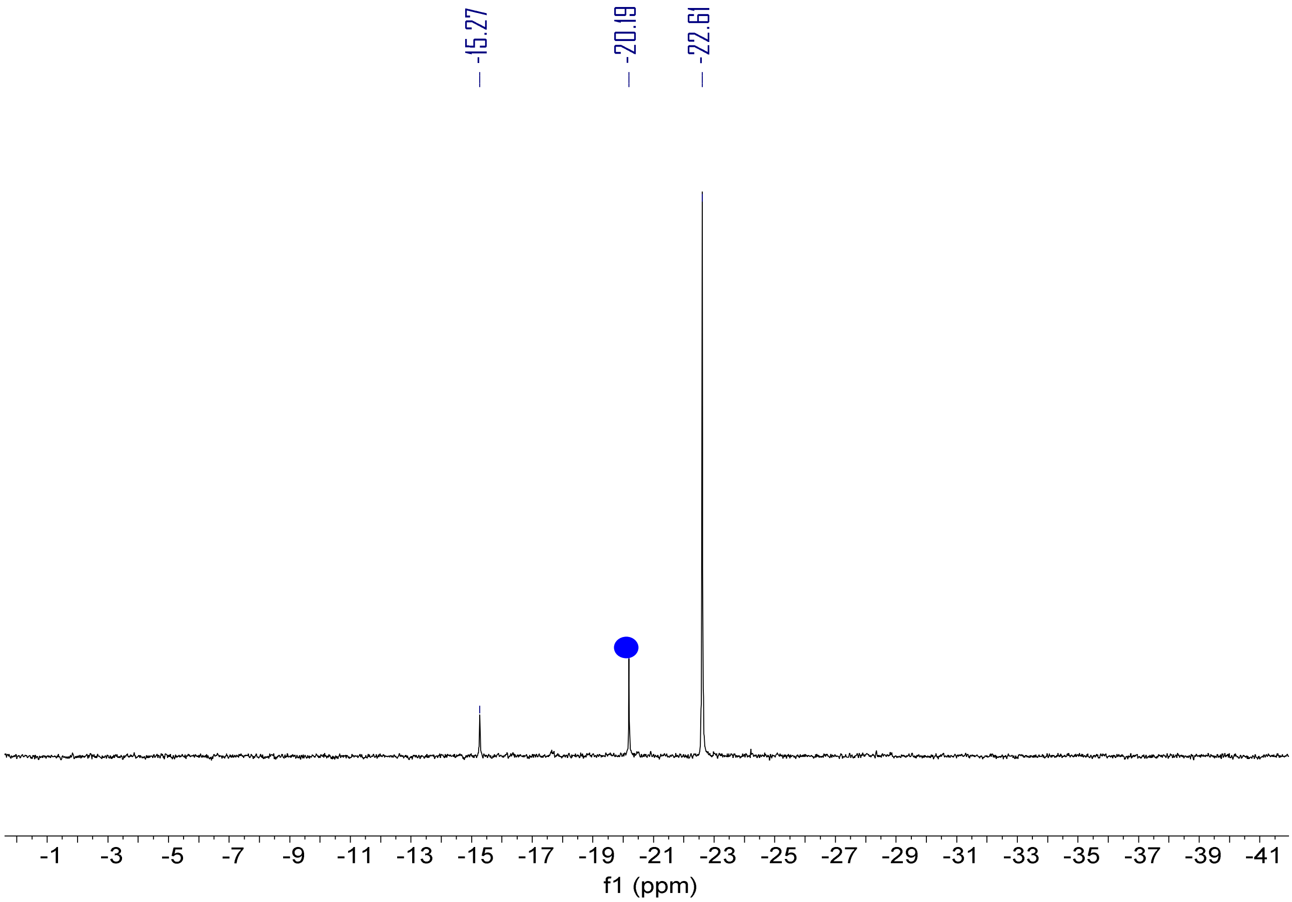

Figure S63. $^{31}P\{^{1}H\}$ NMR spectrum (162.1 MHz, C_6D_6, 298 K) of TipSi(CPh)$_2$ **10** (δ= -22.6 ppm), formed from the *in-situ* reaction between TipSi **4** and diphenylacetylene. The blue circle denotes the resonance of proligand TipH$_2$ **2**.

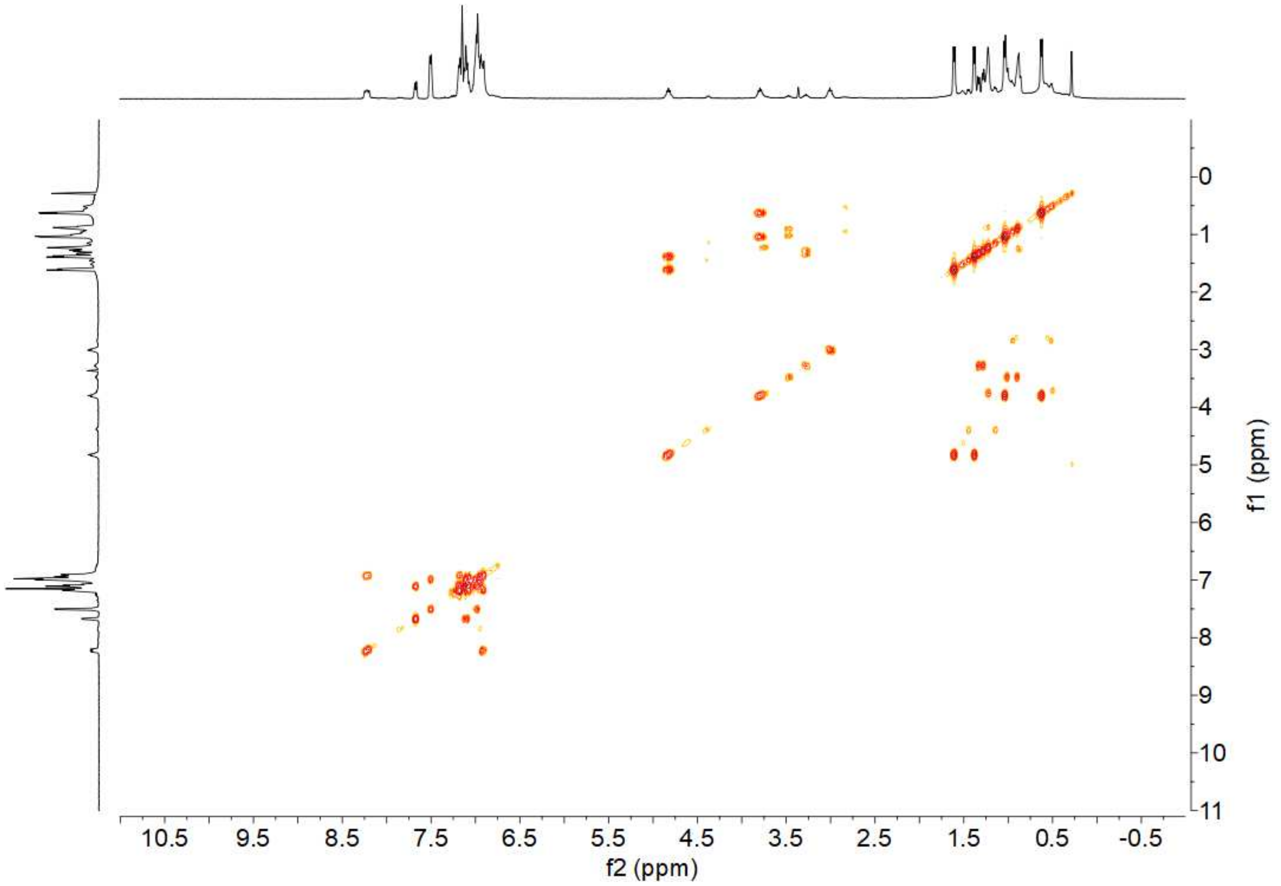

Figure S64. ^{1}H-^{1}H COSY spectrum of TipSi(CPh)$_2$ **10**, formed from the *in-situ* reaction between TipSi **4** and diphenylacetylene.

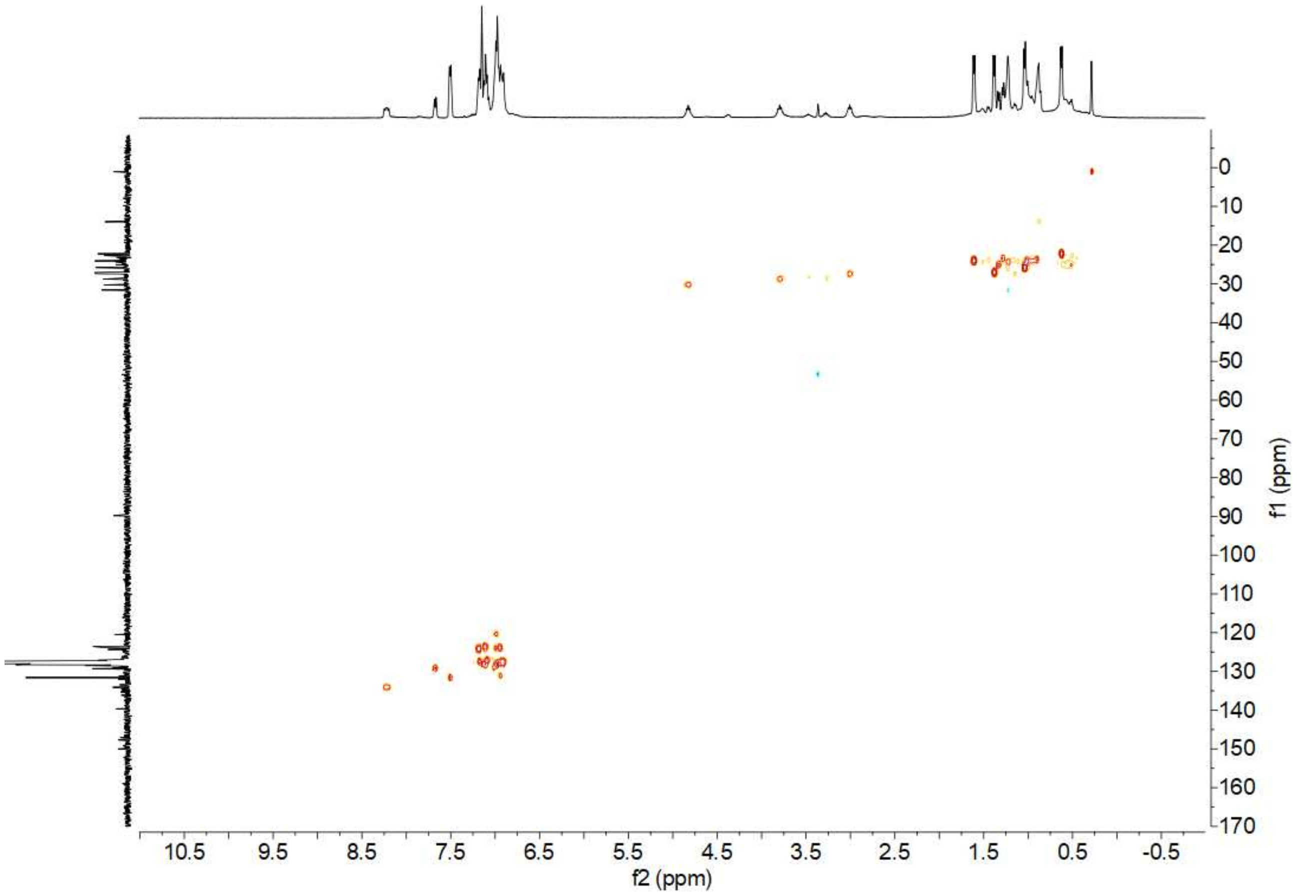

Figure S65. ^{1}H-^{13}C HSQC spectrum of TipSi(CPh)$_2$ **10**, formed from the *in-situ* reaction between TipSi **4** and diphenylacetylene.

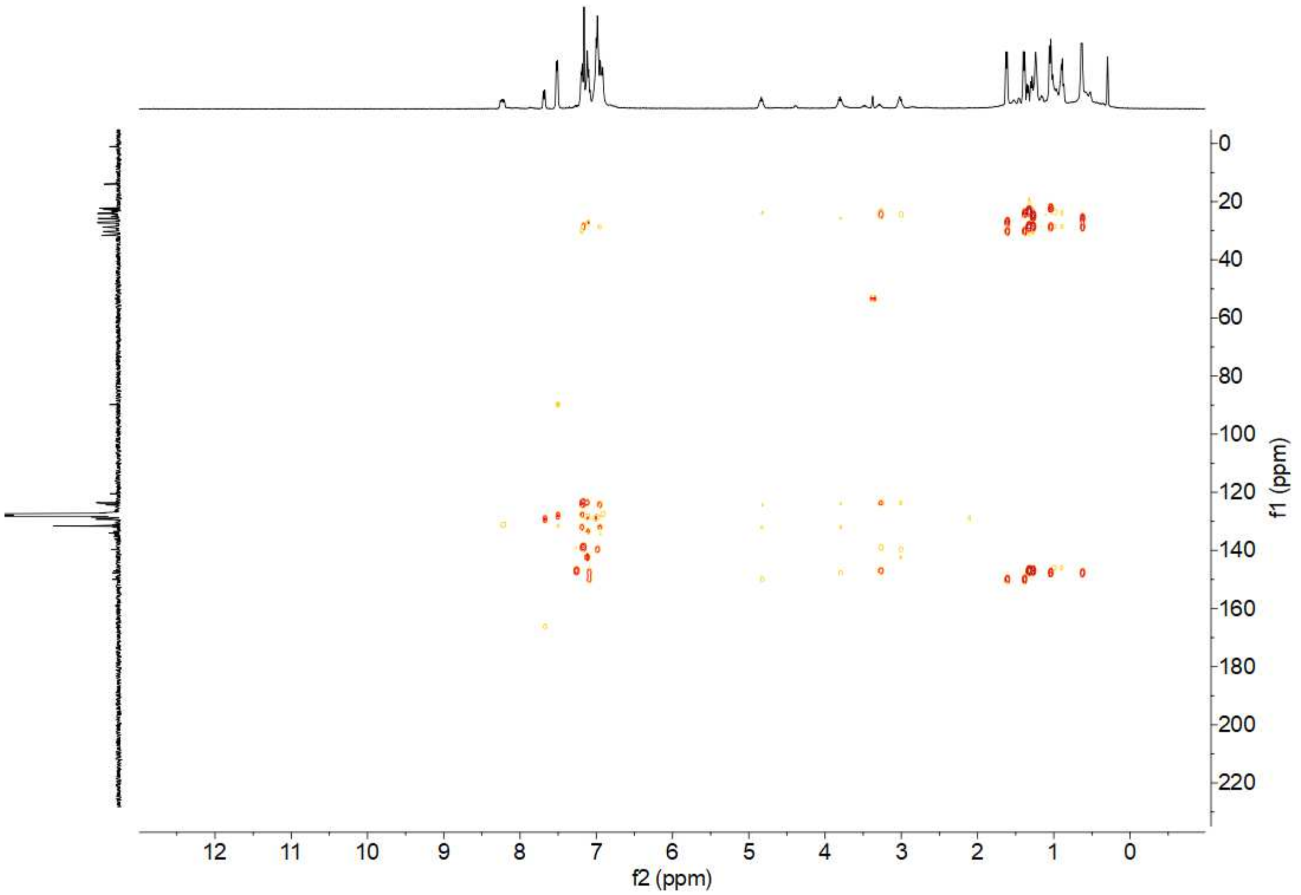

Figure S66. ^{1}H-^{13}C HMBC spectrum of TipSi(CPh)$_2$ **10**, formed from the *in-situ* reaction between TipSi **4** and diphenylacetylene.

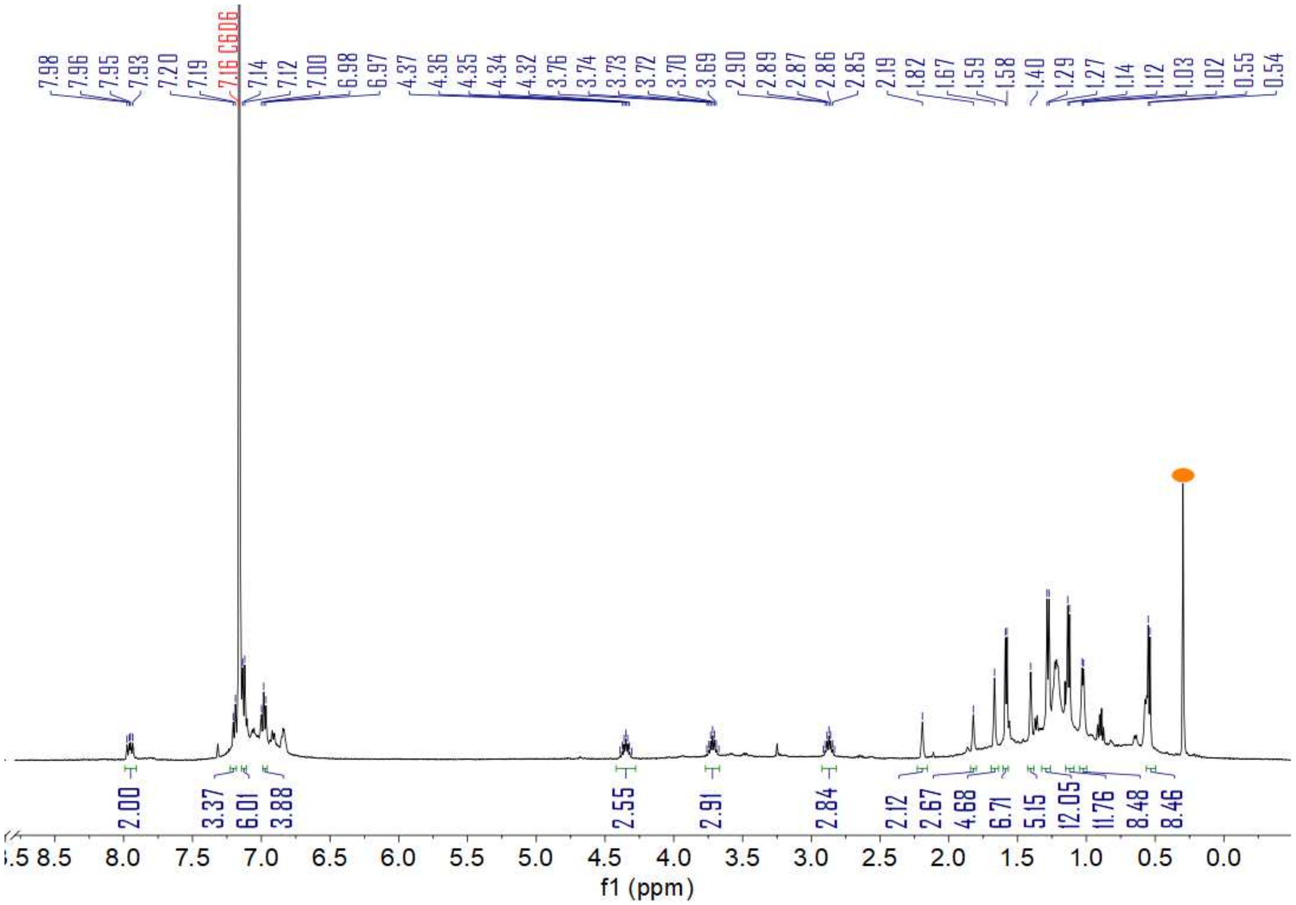

Figure S67. ^{1}H-NMR spectrum (499.9 MHz, C_6D_6, 298 K) of the *in-situ* reaction between TipSi **4** and 2,3-dimethyl-1,3-butadiene, indicating the formation of TipSi{$CH_2C(Me)$}$_2$ **11**. Crystals of **11** were subsequently afforded from this mixture.

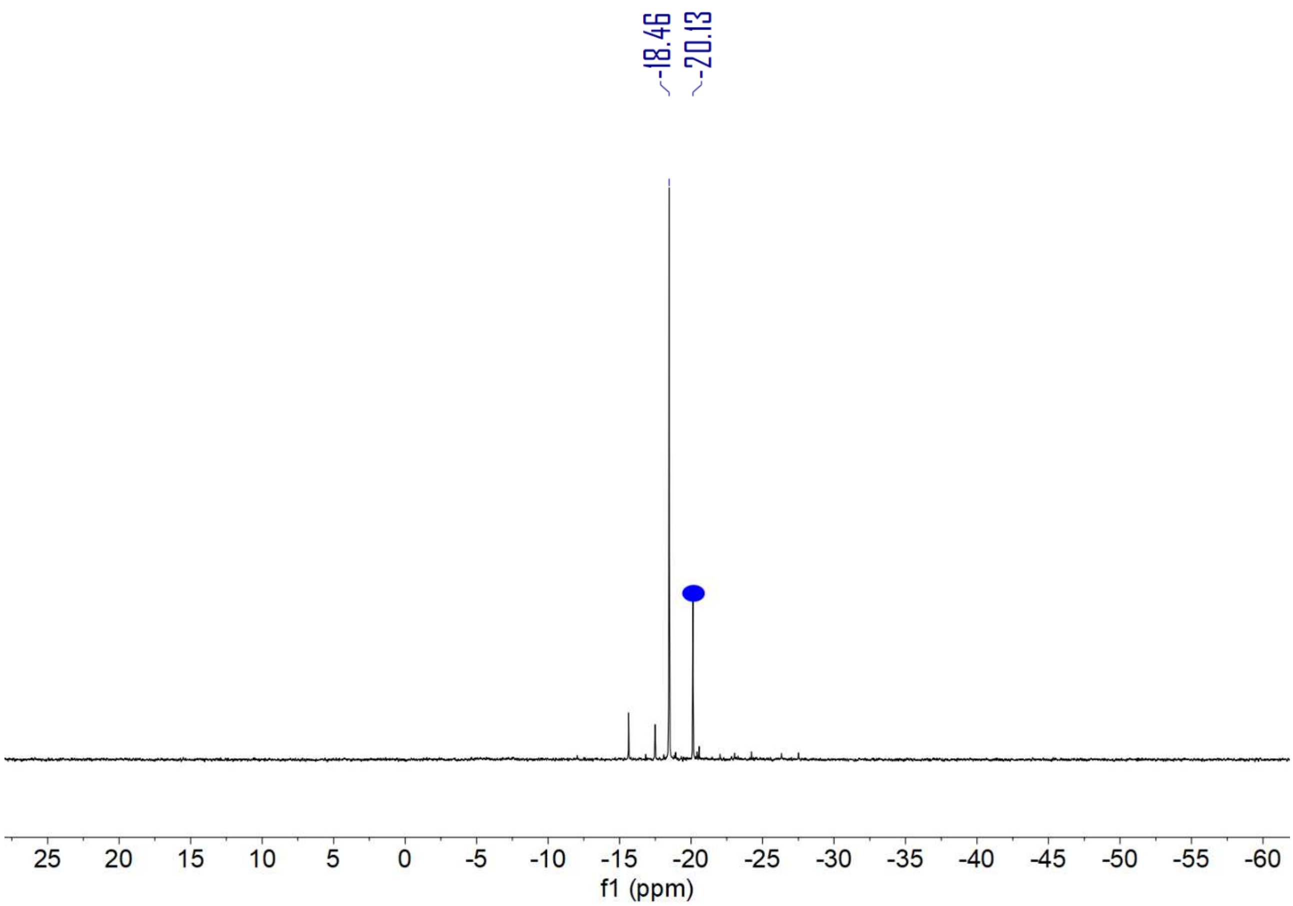

Figure S68. $^{31}P\{^{1}H\}$ NMR spectrum (121.5 MHz, C_6D_6, 298 K) of the *in-situ* reaction between TipSi **4** and 2,3-dimethyl-1,3-butadiene, indicating the formation of TipSi{$CH_2C(Me)$}$_2$ **11** (δ= -18.5 ppm). The blue circle denotes the resonance of proligand $TipH_2$ **2**.

DOSY NMR spectroscopic study of TipSi 4

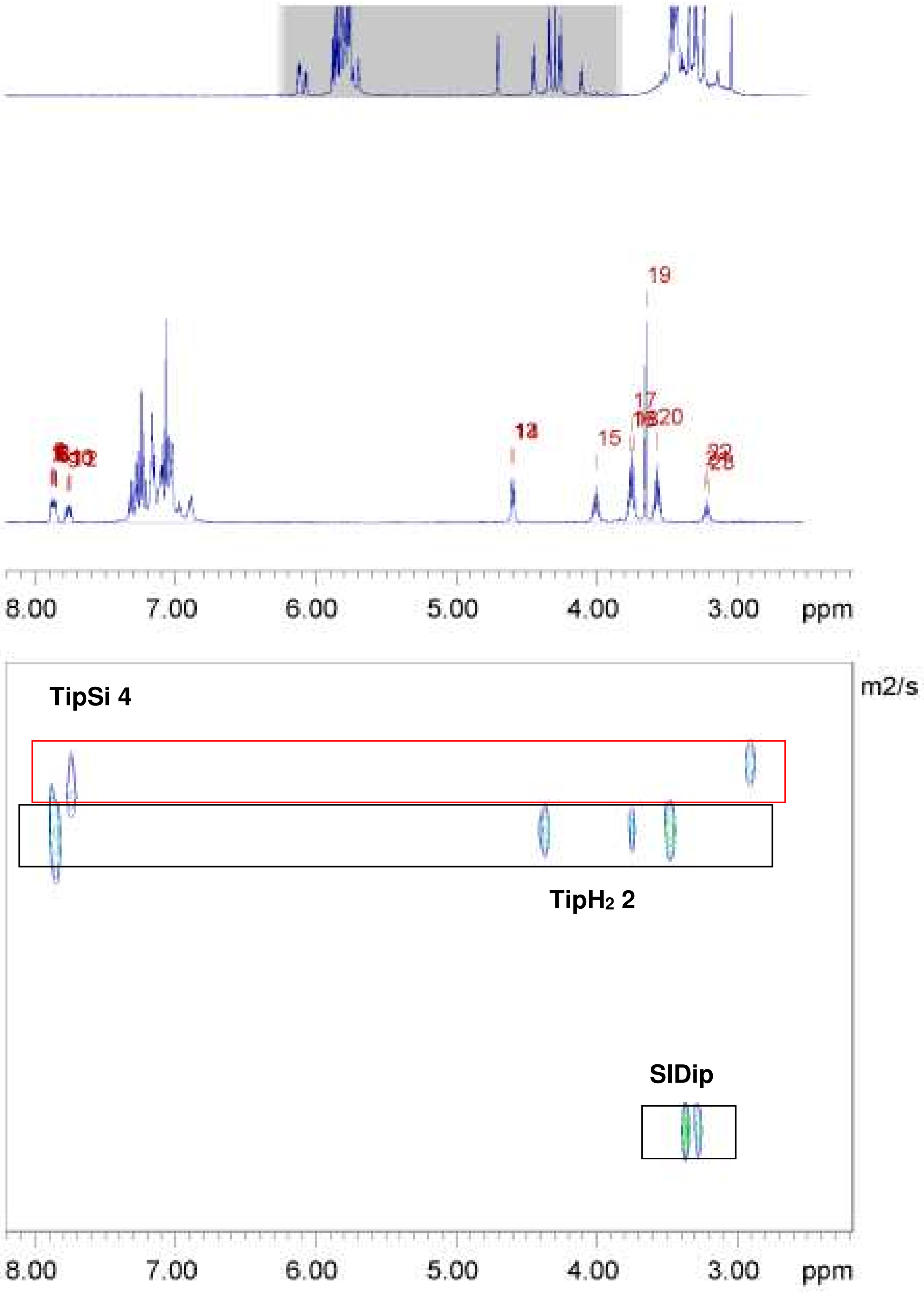

Figure S69. ^{1}H DOSY NMR spectrum (500 MHz, C_6D_6, 298 K) of TipSi **4**, $TipH_2$ **2** and SIDip in deuterated benzene.

Table S1. ^{1}H DOSY NMR determined parameters of TipSi **4**.

D [10^{-10} m^2 s^{-1}]	M_{calc} [g mol^{-1}]	M_{theo} [g mol^{-1}]	Deviation [%]	Assignment
6.17	733	635.76	+15.3	$TipH_2$ **2**
6.04	757	658.96	+14.9	TipSi **4**
7.09	541	390.61	+38.5	SIDip

The average values of the extracted diffusion coefficients from the above spectrum (Figure S60) were used to calculate the estimated molecular weights of the free ligand $TipH_2$ **2** and the corresponding silylene TipSi **4** by using the SEGWE calculator.[7,8]

To qualitatively judge if TipSi **4** is present as a dimer or monomer in deuterated benzene solution, a mixture of TipSi **4**, $TipH_2$ **2** and SIDip was investigated by DOSY NMR spectroscopy. This mixture was studied with the knowledge that $TipH_2$ **2** is a monomeric molecule with a comparable size and shape to monomeric TipSi **4** for direct comparison, see Table S1. The determined molecular weight value for TipSi **4** is only ca. 15% higher than the expected one, an error that is essentially identical to that of $TipH_2$ **2** (Table S1). The (^{1}H) NMR spectroscopic features of TipSi **4** are also similar to those of the heavier congeners **5-7** and together support that TipSi **4** is monomeric in deuterated benzene solution.

3 X-ray Crystallography

X-ray diffraction data for compounds **3**$(THF)_2 \cdot C_6H_6$, $\mathbf{4}_2 \cdot 3\ C_6H_6$, and **11** were collected using a Rigaku MM-007HF High Brilliance RA generator/confocal optics with XtaLAB P100 or P200 diffractometers [Cu Kα radiation (λ = 1.54187 Å)]. X-ray diffraction data for compounds **1**·THF, **3**·1.5 C_7H_8, $\mathbf{4}_2 \cdot C_6H_{14}$, [(TipH)Li(IPr)]·$C_6H_{14}$, [(TipH)Li(IPr)]·2 C_6H_6, [(TipH)Li{N(Dip)PN(Ar')Li}]·2 C_6H_{14} (Ar' = 2-*i*Pr-6(Me_2PhC)C_6H_3), TipHGeCl, $(TipHGe)_2O$, **9**·C_6H_6·0.25 C_6H_{14}, and **10** were collected using a Rigaku FR-X Ultrahigh Brilliance Microfocus RA generator/confocal optics with XtaLAB P200 diffractometer [Mo Kα radiation (λ = 0.71073 Å)]. Data for compounds **1**·THF, **3**$(THF)_2 \cdot C_6H_6$, [(TipH)Li(IPr)]·C_6H_{14}, [(TipH)Li(IPr)]·2 C_6H_6, and $(TipHGe)_2O$) were collected using CrystalClear[9] (using either just ω steps, or both ω and φ steps, accumulating area detector images spanning at least a hemisphere of reciprocal space) and processed (including correction for Lorentz, polarization and absorption) using either CrystalClear or CrysAlisPro.[10] Data for compounds **3**·1.5 C_7H_8, $\mathbf{4}_2 \cdot C_6H_{14}$, [(TipH)Li(IPr)]·2 C_6H_6, TipHGeCl, **8**·C_6H_6·0.25 C_6H_{14}, **10**, and **11** were collected (using a calculated strategy) and processed using CrysAlisPro. Data for $TipH_2$ **2**, **3**·1.5 C_6H_6, [(TipH)K], **5**, **5**·(TipH)Ge(NHDip)·C_5H_{14}, **6**·1.5 C_6H_6, and **7**·C_6H_6 were collected at the MX1[11] and MX2[12] beamlines at the Australian Synchrotron using BluIce[13] for data collection, XDS[14] for data reduction and SADABS[15] for absorption correction.
Structures were solved by dual space (SHELXT[16]) or direct (SIR2011[17]) methods and refined by full-matrix least-squares against F^2 (SHELXL-2019/3[18]). Non-hydrogen atoms were refined anisotropically, and hydrogen atoms were refined using a riding model, except for NH hydrogen atoms in **1**·THF, $TipH_2$ **2**, [(TipH)Li(IPr)]·C_6H_{14}, [(TipH)Li(IPr)]·2 C_6H_6, [(TipH)Li{N(Dip)PN(Ar')Li}]·2 C_6H_{14}, TipHGeCl, and $(TipHGe)_2O$, which were located from the difference Fourier map and refined isotropically with a distance restraint (with thermal motion riding on the parent nitrogen for [(TipH)Li(IPr)]·C_6H_{14}, and [(TipH)Li{N(Dip)PN(Ar)Li}]·2 C_6H_{14}) or in [(TipH)K], and **5**·(TipH)Ge(NHDip)·C_5H_{14}, which were located from the difference Fourier map and refined isotropically without a distance restraint.
Crystals of **10** were affected by non-merohedral twinning, showing a twin law of [-0.9998 -0.0002 -0.0005 -0.0001 -0.9999 -0.0001 -0.6842 -0.0008 -0.9999], and a refined twin fraction of 0.4772(17). The structures **9**·C_6H_6·0.25 C_6H_{14}, and **10** showed void space containing diffuse electron density (1301 and 402 $Å^3$, respectively) and the SQUEEZE[19] routine implemented in PLATON[20] was used to remove the contribution to the diffraction pattern of the unordered electron density in the void spaces. All calculations except SQUEEZE were performed using the CrystalStructure[21] or Olex2[22] interfaces. Selected crystallographic data are presented in Tables S2. CCDC 2495397–2495416

contains the supplementary crystallographic data for this paper. These data can be obtained free of charge from The Cambridge Crystallographic Data Centre via www.ccdc.cam.ac.uk/structures.

Table S2. Crystallographic data.

Compound reference	PhP(NHDip)$_2$·THF, **1**·THF	TipH$_2$ **2**	[TipLi$_2$]·1.5 C_6H_6, **3**·1.5 C_6H_6
formula	$C_{34}H_{49}N_2OP$	$C_{42}H_{58}N_3P$	$C_{51}H_{65}Li_2N_3P$
formula weight	532.75	635.88	764.91
crystal description	Colourless prism	Colourless block	Colourless rod
temperature [K]	173	100	100
wavelength [Å]	0.71075	0.7108	0.7108
space group	$P\bar{1}$	$P\bar{1}$	$P2_1/n$
a [Å]	11.0815(2)	9.1590(18)	12.412(3)
b [Å]	11.4734(3)	11.767(2)	23.768(5)
c [Å]	14.8485(3)	17.194(3)	15.741(3)
α [°]	110.450(2)	90.87(3)	90
β [°]	99.8933(18)	97.76(3)	109.07(3)
γ [°]	107.899(2)	100.14(3)	90
vol [Å]^3	1598.63(7)	1806.0(7)	4389.0(17)
Z	2	2	4
ρ (calc) [g/cm^3]	1.107	1.169	1.158
μ [mm^{-1}]	0.113	0.109	0.100
F(000)	580	692	1652
theta range for data collection [°]	1.541 to 25.376	1.196 to 30.380	1.615 to 27.887
reflections collected	23845	66690	39475
independent reflections (R_{int})	5813 (0.0368)	10401 (0.0901)	10381 (0.0841)
parameters, restraints	426, 168	439, 3	582, 24
GoF on F^2	1.041	1.050	1.013
R_1 [$I > 2\sigma(I)$]	0.0477	0.0588	0.0543
wR_2 (all data)	0.1435	0.1659	0.1417
largest diff. peak/hole [e/Å^3]	0.31, -0.31	0.538, -0.563	0.399, -0.352
Flack x parameter	-	-	-
CCDC number	2495397	2495409	2495410

Table S2 continued 2. Crystallographic data.

Compound reference	[$TipLi_2$]·1.5 C_7H_8, **3**·1.5 C_7H_8	[Tip($LiTHF)_2$]·C_6H_6, **3**$(THF)_2$·C_6H_6	[(TipH)K]
formula	$C_{52.50}H_{68}Li_2N_3P$	$C_{53}H_{75}Li_2N_3O_2P$	$C_{42}H_{57}KN_3P$
formula weight	785.94	831.05	673.97
crystal description	Colourless prism	Colourless chunk	Colourless rod
temperature [K]	100	173	100
wavelength [Å]	0.71073	1.54187	0.7108
space group	$P2_1/n$	$P2_1$/c	$P2_1/n$
a [Å]	12.5559(3)	11.6991(5)	10.666(2)
b [Å]	23.8762(7)	24.7026(15)	19.194(4)
c [Å]	15.8665(5)	18.4560(5)	18.690(4)
α [°]	90	909	90
β [°]	110.053(3)	92.628(3)	91.35(3)
γ [°]	90	90	90
vol [Å]^3	4468.2(2)	5328.1(4)	3825.2(13)
Z	4	4	4
ρ (calc) [g/cm^3]	1.168	1.036	1.170
μ [mm^{-1}]	0.100	0.739	0.213
F(000)	1700	1804	1456
theta range for data collection [°]	1.989 to 29.049	3.579 to 68.429	1.521 to 31.888
reflections collected	80997	53934	37056
independent reflections (R_{int})	10682 (0.0391)	9623 (0.1108)	10722 (0.0941)
parameters, restraints	640, 157	590, 186	440, 0
GoF on F^2	1.163	1.034	1.021
R_1 [$I > 2\sigma(I)$]	0.0889	0.0902	0.0582
wR_2 (all data)	0.2558	0.2873	0.1527
largest diff. peak/hole [e/Å^3]	0.733, -0.609	0.91, -0.29	0.374, -0.588
Flack x parameter	-	-	-
CCDC number	2495403	2495399	2495414

Table S2 continued 3. Crystallographic data.

Compound reference	$(TipSi)_2 \cdot C_6H_{14}$, **4**$_2 \cdot C_6H_{14}$	$(TipSi)_2 \cdot 3\ C_6H_6$, **4**$_2 \cdot 3\ C_6H_6$	$[(TipH)Li(IPr)] \cdot C_6H_{14}$
formula	$C_{90}H_{126}N_6P_2Si_2$	$C_{102}H_{130}N_6P_2Si_2$	$C_{72}H_{100}LiN_5P$
formula weight	1410.08	1558.23	1073.53
crystal description	Pale yellow plate	Yellow plate	Colourless prism
temperature [K]	100	173	173
wavelength [Å]	0.71073	1.54184	0.71073
space group	*Cmca*	$P2_1$	$P\bar{1}$
a [Å]	21.3502(6)	11.6316(2)	12.57840(18)
b [Å]	24.9121(6)	24.1679(6)	14.5085(2)
c [Å]	15.5715(4)	16.5376(3)	18.8002(3)
α [°]	90	90	85.4812(12)
β [°]	90	93.771(2)	88.2767(12)
γ [°]	90	90	86.9071(11)
vol $[Å]^3$	8282.2(4)	4638.84(17)	3414.12(9)
Z	4	2	2
ρ (calc) $[g/cm^3]$	1.131	1.116	1.044
μ $[mm^{-1}]$	0.129	1.034	0.082
F(000)	3064	1684	1170
theta range for data collection [°]	2.094 to 29.112	2.678 to 66.648	1.622 to 28.510
reflections collected	35979	82503	103181
independent reflections (R_{int})	5105 (0.0315)	15590 (0.0502)	15015 (0.0296)
parameters, restraints	290, 85	1123, 333	761, 56
GoF on F^2	1.043	1.021	1.042
R_1 $[I > 2\sigma(I)]$	0.0418	0.0449	0.0658
wR_2 (all data)	0.1036	0.1261	0.2050
largest diff. peak/hole $[e/Å^3]$	0.371, -0.433	0.365, -0.222	1.24, -0.28
Flack *x* parameter	-	0.095(10)	-
CCDC number	2495407	2495415	2495413

Table S2 continued 4. Crystallographic data.

Compound reference	[(TipH)Li(IPr)]·2 C_6H_6,	[(TipH)Li{N(Dip)PN(Ar)Li}]·2 C_6H_{14},	TipGe, **5**
formula	$C_{81}H_{105}LiN_5P$	$C_{82}H_{119}Li_2N_5P_2$	$C_{42}H_{56}GeN_3P$
formula weight	1186.60	1250.71	706.45
crystal description	Colourless prism	Colourless prism	Orange-yellow plate
temperature [K]	100	93	100
wavelength [Å]	0.71073	0.71073	0.7108
space group	$P2_1/n$	$P2_1/c$	$P2_1/n$
a [Å]	13.2276(2)	14.909(4)	11.355(2)
b [Å]	38.0655(6)	28.540(5)	23.029(5)
c [Å]	14.09229(19)	18.284(4)	15.474(3)
α [°]	90	90	90
β [°]	91.5919(13)	95.709(5)	100.99(3)
γ [°]	90	90	90
vol [Å]^3	7092.96(18)	7741(3)	3972.2(14)
Z	4	4	4
ρ (calc) [g/cm^3]	1.111	1.073	1.181
μ [mm^{-1}]	0.085	0.100	0.843
F(000)	2576	2728	1504
theta range for data collection [°]	1.875 to 29.096	2.239 to 25.391	2.464 to 31.369
reflections collected	142183	111627	39472
independent reflections (R_{int})	16906 (0.0443)	14136 (0.0502)	11268 (0.0803)
parameters, restraints	932, 356	936, 131	466, 6
GoF on F^2	1.034	1.041	1.035
R_1 [$I > 2\sigma(I)$]	0.0539	0.0424	0.0549
wR_2 (all data)	0.1432	0.1240	0.1519
largest diff. peak/hole [e/Å^3]	0.599, -0.311	0.56, -0.32	1.650, -1.124
Flack x parameter	-	-	-
CCDC number	2495408	2495405	2495398

Table S2 continued 5. Crystallographic data.

Compound reference	**5**·(TipH)Ge(NHDip)·C_5H_{14}	TipHGeCl	$(TipHGe)_2O$
formula	$C_{98.50}H_{137}Ge_2N_7P_2$	C42 H57 Cl Ge N3 P	$C_{84}H_{114}Ge_2N_6OP_2$
formula weight	1626.26	742.91	1430.99
crystal description	Yellow block	Colourless prism	Colourless block
temperature [K]	123	100	173
wavelength [Å]	0.71073	0.71073	0.71073
space group	$P\bar{1}$	$P2_1/n$	$P\bar{1}$
a [Å]	11.6469(5)	12.26847(19)	11.1932(3)
b [Å]	20.2513(8)	12.3073(2)	12.6219(3)
c [Å]	21.2764(9)	26.8273(5)	16.4649(6)
α [°]	107.310(2)	90	69.003(6)
β [°]	105.447(2)	98.7113(16)	75.001(7)
γ [°]	94.488(2)	90	65.603(6)
vol $[Å]^3$	4549.9(3)	4003.97(12)	1960.91(15)
Z	2	4	1
ρ (calc) [g/cm^3]	1.187	1.232	1.212
μ [mm^{-1}]	0.744	0.904	0.855
F(000)	1742	1576	762
theta range for data collection [°]	2.129 to 28.896	1.824 to 29.075	1.849 to 25.380
reflections collected	87890	76319	40292
independent reflections (R_{int})	22139 (0.0440)	9514 (0.0354)	7154 (0.0572)
parameters, restraints	1099, 29	449, 1	446, 1
GoF on F^2	1.070	1.066	1.045
R_1 [$I > 2\sigma(I)$]	0.0441	0.0283	0.0248
wR_2 (all data)	0.1130	0.0692	0.0688
largest diff. peak/hole [e/Å^3]	1.249, -0.833	0.373, -0.320	0.33, -0.31
Flack *x* parameter	-		-
CCDC number	2495406	2495404	2495412

Table S2 continued 6. Crystallographic data.

Compound reference	TipSn·1.5 C_6H_6, **6**·1.5 C_6H_6	TipPb·C_6H_6, **7**·C_6H_6	TipSi(DMAP)·C_6H_6·0.25 C_6H_{14}, **9**·C_6H_6·0.25 C_6H_{14}
formula	$C_{51}H_{65}N_3PSn$	$C_{48}H_{62}N_3PPb$	$C_{56.50}H_{75.50}N_5PSi$
formula weight	869.72	919.16	883.77
crystal description	Orange-yellow plate	Yellow block	Yellow plate
temperature [K]	100	100	100
wavelength [Å]	0.71073	0.7108	0.71073
space group	*C*2/*c*	*P*2_1/*c*	*P*2_1/*n*
a [Å]	25.751(5)	12.474(3)	21.7059(7)
b [Å]	20.104(4)	20.279(4)	22.0058(5)
c [Å]	17.632(4)	17.693(4)	23.8990(9)
α [°]	90	90	90
β [°]	95.68(3)	101.84(3)	113.200(4)
γ [°]	90	90	90
vol $[Å]^3$	9084(3)	4380.5(16)	10492.4(7)
Z	8	4	8
ρ (calc) [g/cm^3]	1.272	1.394	1.119
μ [mm^{-1}]	0.635	3.923	0.116
F(000)	3656	1872	3828
theta range for data collection [°]	1.287 to 28.000	1.546 to 27.869	1.961 to 29.100
reflections collected	59818	38971	118113
independent reflections (R_{int})	10863 (0.1079)	10369 (0.0724)	24356 (0.0691)
parameters, restraints	544, 45	490, 0	1228, 120
GoF on F^2	1.039	1.029	1.016
R_1 [$I > 2\sigma(I)$]	0.0438	0.0404	0.0571
wR_2 (all data)	0.1106	0.1070	0.1255
largest diff. peak/hole [e/Å^3]	1.035, -2.444	1.649, -3.352	0.485, -0.331
Flack *x* parameter	-	-	-
CCDC number	2495401	2495400	2495416

Table S2 continued 7. Crystallographic data.

Compound reference	TipSi(CPh)$_2$ **10**	TipSi{CH$_2$C(Me)}$_2$ **11**
formula	$C_{56}H_{66}N_3PSi$	$C_{48}H_{66}N_3PSi$
formula weight	840.17	744.09
crystal description	Colourless prism	Colourless prism
temperature [K]	100	125
wavelength [Å]	0.71073	1.54184
space group	*I*2	$P2_1/n$
a [Å]	21.3600(8)	10.08661(11)
b [Å]	15.6979(4)	41.5414(5)
c [Å]	32.3634(9)	11.49255(13)
α [°]	90	90
β [°]	102.818(3)	115.1909(13)
γ [°]	90	90
vol [Å]^3	10581.2(6)	4357.54(9)
Z	8	4
ρ (calc) [g/cm^3]	1.055	1.134
μ [mm^{-1}]	0.111	1.077
F(000)	3616	1616
theta range for data collection [°]	1.624 to 29.262	2.127 to 75.656
reflections collected	152366	81900
independent reflections (R_{int})	37777 (0.0737)	8875 (0.0472)
parameters, restraints	1401, 2002	806, 52
GoF on F^2	1.081	1.101
R_1 [$I > 2\sigma(I)$]	0.0892	0.0564
wR_2 (all data)	0.2784	0.1538
largest diff. peak/hole [e/Å^3]	0.631, -0.950	0.468, -0.792
Flack *x* parameter	0.36(6)	-
CCDC number	2495402	2495411

PhP(NHDip)$_2$·THF, **1**·THF

Compound **1** is an oil, but in one instance, **1**·THF could be crystallised from an *n*-pentane/THF mixture. This compound crystallised with one full molecule in the asymmetric unit. The structure shows disorder in one isopropyl group which was modelled using two positions for the terminal methyls. The THF solvate also shows disorder which was modelled using two positions and refined with geometric and thermal restraints.

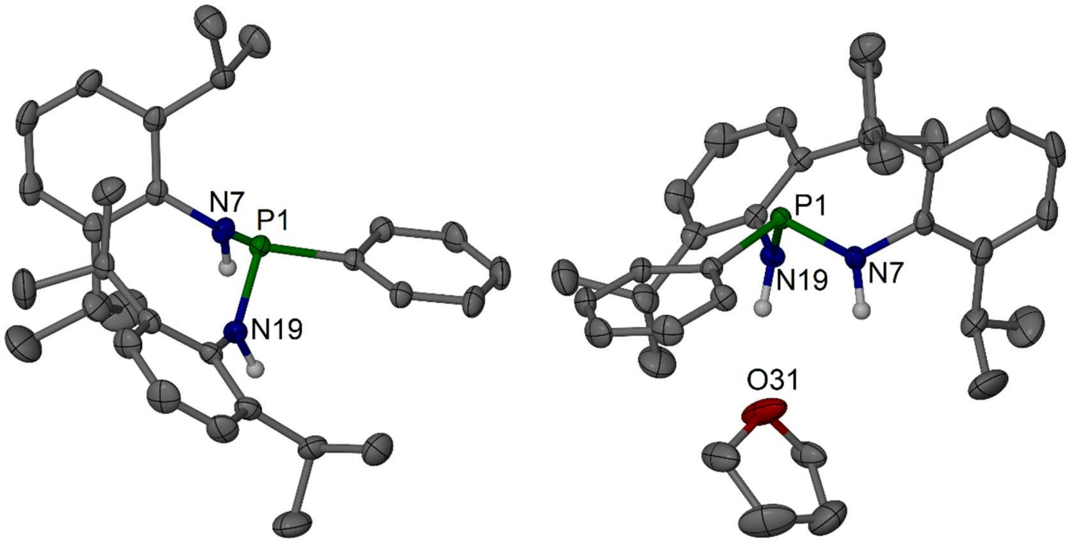

Figure S70. Molecular structure of PhP(NHDip)$_2$·THF, **1**·THF, shown as 30% thermal ellipsoids. Hydrogen atoms except for NH are omitted for clarity. Minor components of disorder are not shown. The left image shows only compound **1** and the right image of **1**·THF shows the position of THF with hydrogen-bonded contacts to **1**. Selected bond lengths (Å) and angles (°): P1–N7 1.7152(13), P1–N19 1.7016(14), P1–C1 1.8360(16); N19–P1–N7 105.82(7), N19–P1–C1 101.36(7), N7–P1–C1 97.54(7).

TipH$_2$, PhP(=NDip)(NHDip)$_2$, **2**

Compound **2** crystallised with a full molecule in the asymmetric unit. There is no clear distinction between and ordering of the P=N and P–N bonds in the structure and three (N)H positions were found and refined with 66.7% (two-thirds) occupancy each.

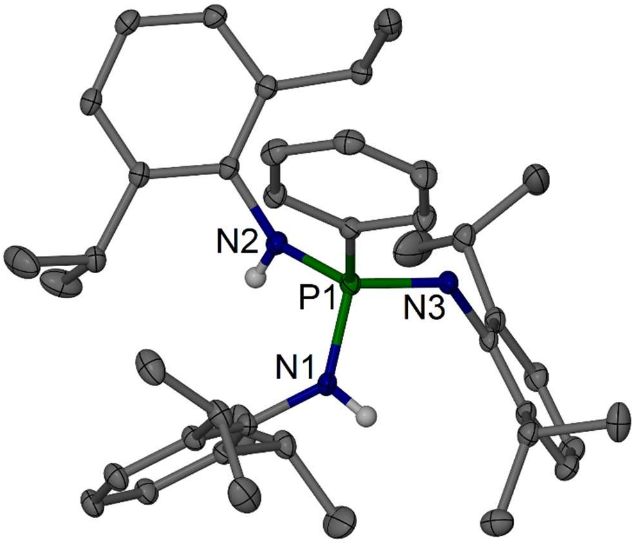

Figure S71. Molecular structure of TipH$_2$ **2** shown as 30% thermal ellipsoids. Hydrogen atoms except for two of the three NH positions are omitted for clarity. Selected bond lengths (Å) and angles (°): P1–N1 1.6282(15), P1–N2 1.6052(15), P1–N3 1.6049(13), P1–C1 1.7918(15); N2–P1–N1 105.49(8), N3–P1–N1 104.30(7), N3–P1–N2 122.25(8), N1–P1–C1 113.86(8), N2–P1–C1 107.67(7), N3–P1–C1 103.60(7), C7–N1–P1 128.62(11), C19–N2–P1 135.23(11), C31–N3–P1 126.86(10).

$[TipLi_2]\cdot 1.5\ C_6H_6$, **3**$\cdot$1.5 C_6H_6 and $[TipLi_2]\cdot 1.5\ C_7H_8$, **3**$\cdot$1.5 C_7H_8

Both solvates of $[TipLi_2]$ **3** crystallised with a full monomeric unit and lattice solvent in the asymmetric unit, and their unit cells are similar. The monomeric units are arranged in one-dimensional chains via intermolecular Li····C interactions (via Li2) and the low-coordinate Li centres show weak contacts to hydrogen atoms of C–H bonds.

The structure of $[TipLi_2]\cdot 1.5\ C_6H_6$, **3**$\cdot$1.5 C_6H_6 shows a disordered isopropyl group which was modelled using two positions and refined with geometric restraints. One of the benzene solvate molecules is disordered and was modelled using two positions and refined with geometric restraints.

The structure of $[TipLi_2]\cdot 1.5\ C_7H_8$, **3**$\cdot$1.5 C_7H_8 shows a disordered isopropyl group which was modelled using two positions and refined with geometric and thermal restraints. One of the toluene solvates was disordered over a symmetry element and was placed with FragmentDB[23,24] using an idealised geometry and refined with an occupancy fixed at 0.5.

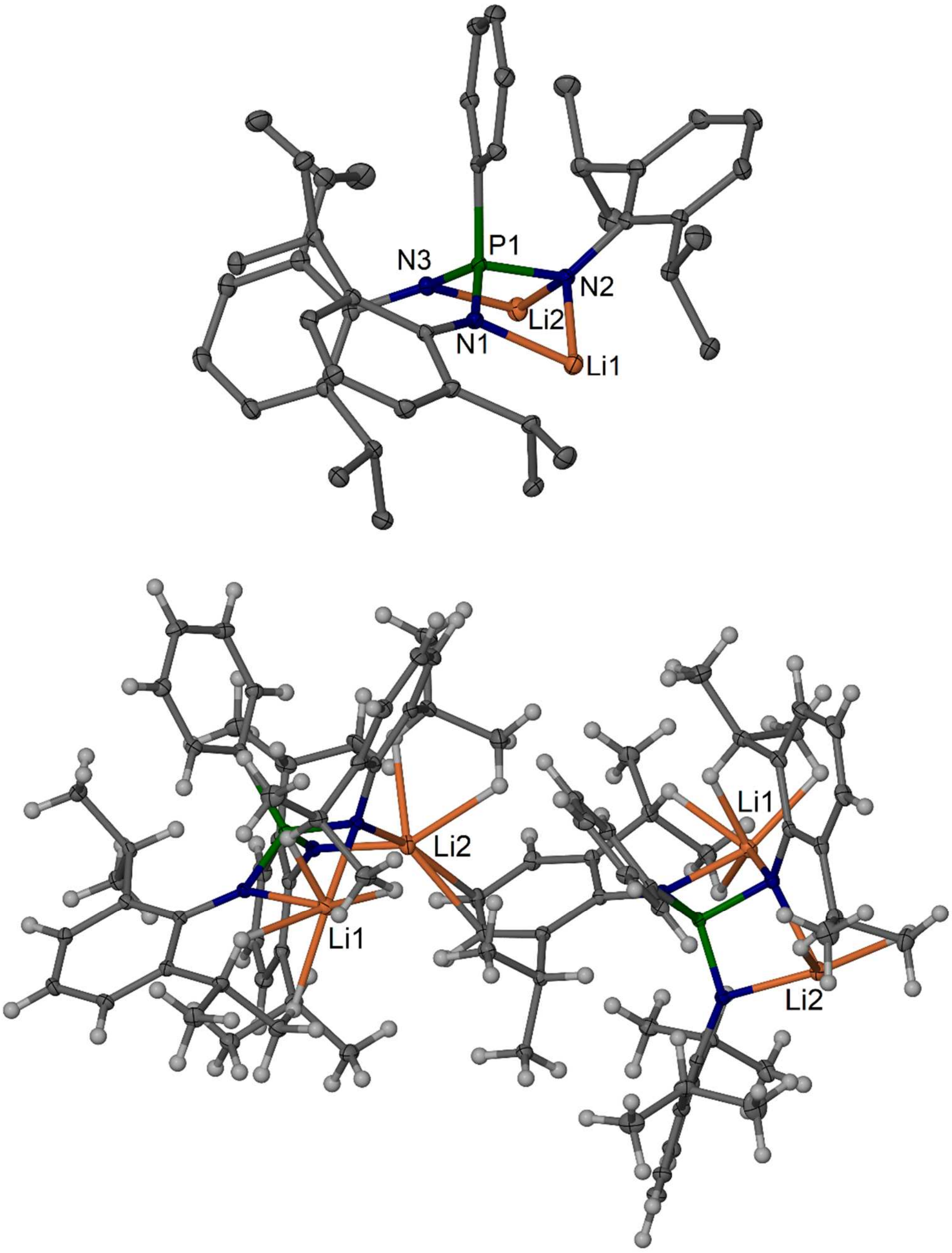

Figure S72. The molecular structures of [TipLi$_2$]·1.5 C_6H_6, **3**·1.5 C_6H_6, shown as 30% thermal ellipsoids. Solvent molecules and minor components of disorder are omitted for clarity. Top: monomeric unit, no hydrogen atoms shown. Bottom: two units shown that include Li···C and Li···H contacts. Selected bond lengths (Å) and angles (°): P1–N1 1.6286(15), P1–N2 1.6470(16), P1–N3 1.6015(15), P1–C1 1.8441(18), N1–Li1 1.966(4), Li1–N2 2.024(4), N2–Li2 2.022(4), Li2–N3 1.958(4), Li2–C10’ 2.550(4), Li2–C11’ 2.423(4); N1–P1–N2 104.66(8), N3–P1–N2 101.63(8), N3–P1–N1 117.77(8), N1–P1–C1 107.31(8), N2–P1–C1 104.82(8), C7–N1–P1 137.55(12), C7–N1–P1 137.55(12), C19–N2–P1 124.59(12), C31–N3–P1 137.67(13), N1–Li1–N2 81.05(13), N3–Li2–N2 78.47(14).

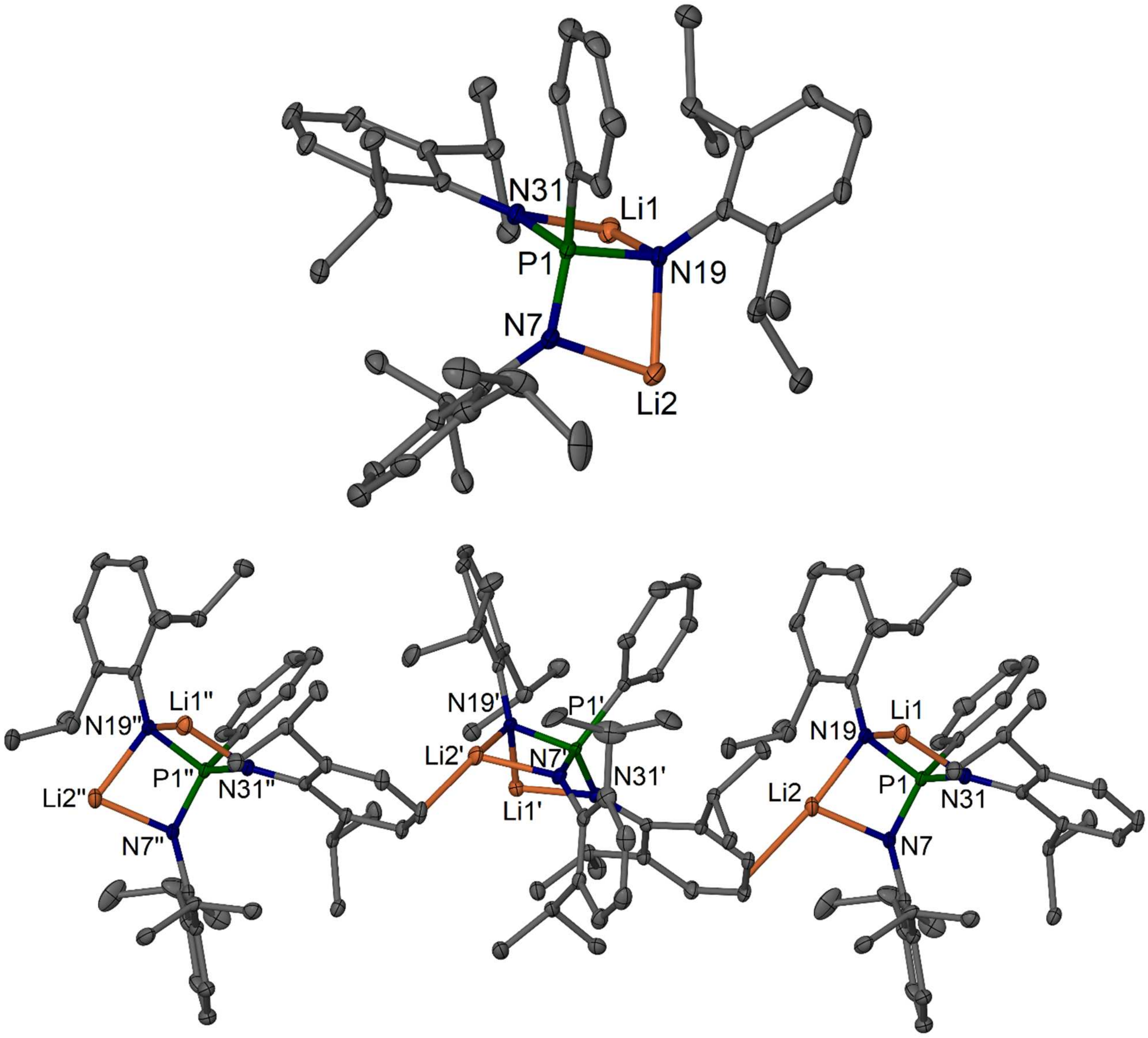

Figure S73. The molecular structures of [TipLi$_2$]·1.5 C_7H_8, **3**·1.5 C_7H_8, shown as 30% thermal ellipsoids. Hydrogen atoms, solvent molecules and minor disordered parts are omitted for clarity. Top: monomeric unit. Bottom: three units in the one-dimensional polymer shown including Li···C contacts. Selected bond lengths (Å) and angles (°): P1–N7 1.598(3), P1–N19 1.650(3), P1–N31 1.628(3), P1–C1 1.837(3), N19–Li1 2.022(7), N19–Li2 2.025(7), N7–Li2 1.957(6), N31–Li1 1.965(6), C33’–Li2 2.395(7), C34’–Li2 2.483(7); N7–P1–N19 102.04(14), N7–P1–N31 117.69(14), N31–P1–N19 104.38(15), N7–P1–C1 118.81(16), N19–P1–C1 104.57(14), N31–P1–C1 107.32(14), C7–N7–P1 137.3(2), C19–N19–P1 124.8(2), C31–N31–P1 137.5(2), N7–Li2–N19 78.7(2), N31–Li1–N19 81.0(3).

[Tip(LiTHF)$_2$]·C_6H_6 , **3**(THF)$_2$·C_6H_6

This complex crystallised with a full main molecule in the asymmetric unit. The structure shows disorder in the orientation of the benzene solvate which was modelled using two positions and refined with thermal restraints.

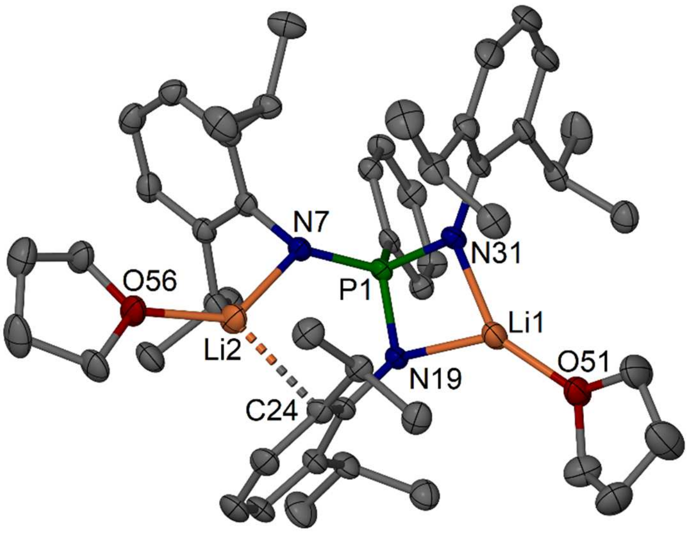

Figure S74. The molecular structure of [Tip(LiTHF)$_2$]·C_6H_6, **3**(THF)$_2$·C_6H_6, shown as 30% thermal ellipsoids. Hydrogen atoms and the solvent molecule are omitted for clarity. Selected bond lengths (Å) and angles (°): P1–N7 1.611(3), P1–N19 1.627(3), P1–N31 1.622(3), P1–C1 1.835(3), P1–Li1 2.519(8), O51–Li1 1.889(8), N19–Li1 1.961(8), N31–Li1 1.926(9), N7–Li2 1.936(8), O56–Li2 1.905(8), C24–Li2 2.382(9), C7–Li2 2.564(8), C19–Li2 2.703(9); N7–P1–N19 111.42(16), N7–P1–N31 121.61(17), N31–P1–N19 100.43(16), N7–P1–C1 107.09(16), N19–P1–C1 109.66(17), N31–P1–C1 106.15(15), N31–Li1–N19 79.9(3), O51–Li1–N19 135.4(5), O51–Li1–N31 144.6(5), O56–Li2–N7 132.1(4), O56–Li2–C24 127.7(4), N7–Li2–C24 98.9(3), P1–N7–Li2 123.4(3).

[(TipH)K]

The complex crystallised with a full formula unit in the asymmetric unit and forms a one-dimensional polymer via K····arene interactions.

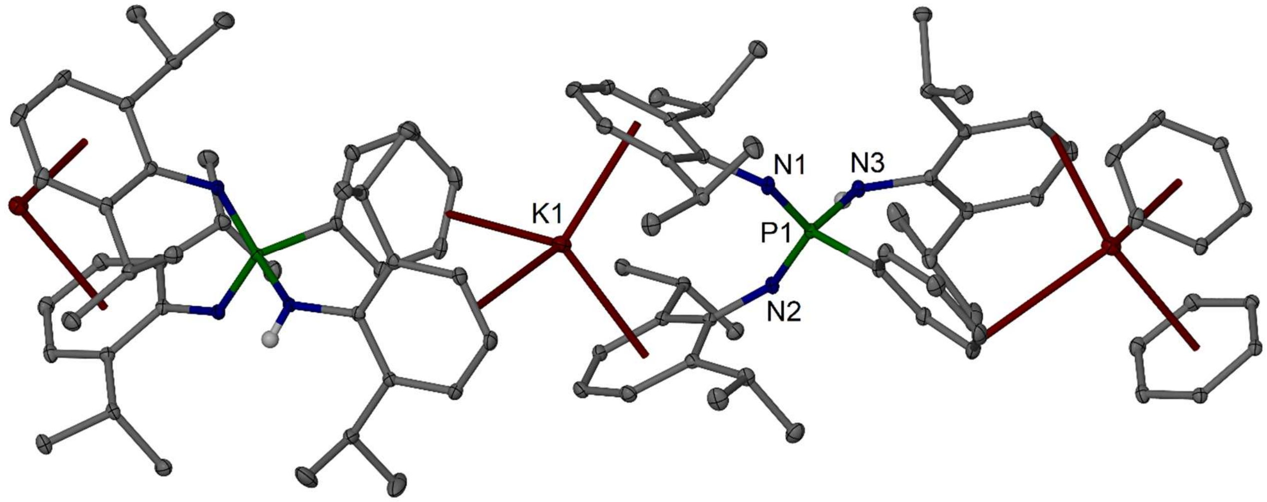

Figure S75. The molecular structure of [(TipH)K] as a section of the polymeric structure shown as 30% thermal ellipsoids. Hydrogen atoms are omitted for clarity. Only one asymmetric unit contains selected atom labels. Selected bond lengths (Å) and angles (°): P1–N1 1.5660(17), P1–N2 1.5986(17), P1–N3 1.7007(17), P1–C1 1.8266(19), K1–C7 3.279(2), K1–C8 3.193(2), K1–C9 3.141(2), K1–C10 3.150(2), K1–C11 3.181(2), K1–C12 3.256(2), K1–C21 3.173(2), K1–C22 3.058(2), K1–C23 3.186(2), K1–C33' 3.278(2), K1–C34' 3.314(2), K1–C5' 3.351(2); N1–P1–N2 119.64(9), N1–P1–N3 117.24(9), N2–P1–N3 100.14(9), N1–P1–C1 104.35(9), N2–P1–C1 113.39(9), N3–P1–C1 100.87(8).

$(TipSi)_2 \cdot C_6H_{14}$, $\mathbf{4}_2 \cdot C_6H_{14}$

Compound $(TipSi)_2 \cdot C_6H_{14}$, $\mathbf{4}_2 \cdot C_6H_{14}$, crystallised with a quarter of the main molecule (dimer) in the asymmetric unit. The structure shows positional disorder in the terminal methyl of one isopropyl group which was modelled using two positions. The hexane solvate was disordered over multiple symmetry elements and was placed with FragmentDB[23,24] using an idealised geometry and refined with an occupancy fixed at 0.25, and with strong geometric and thermal restraints.

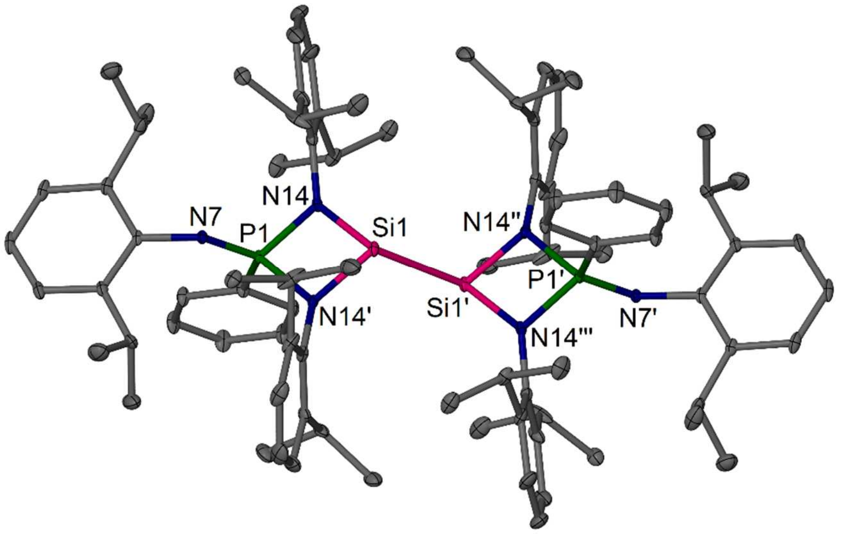

Figure S76. The molecular structure of $(TipSi)_2 \cdot C_6H_{14}$, $\mathbf{4}_2 \cdot C_6H_{14}$, shown as 30% thermal ellipsoids. Hydrogen atoms, the minor component of disorder, and solvent are omitted for clarity. Selected bond lengths (Å) and angles (°): Si1–Si1' 2.5916(9), Si1–N14 1.7510(11), Si1–N14' 1.7510(11), P1–N14 1.7060(11), P1–N14' 1.7060(11), P1–N7 1.5333(15), P1–C1 1.8054(18), N7–C7 1.409(2); N14–Si1–N14' 82.51(7), N7–P1–N14 117.62(5), N7–P1–N14' 117.62(5), N14–P1–N14' 85.20(7), N7–P1–C1 116.33(8), N14–P1–C1 107.87(6), N14'–P1–C1 107.87(5), C7–N7–P1 136.52(12), ϑ 55.48°.

$(TipSi)_2 \cdot 3\ C_6H_6$, $\mathbf{4}_2 \cdot 3\ C_6H_6$

Compound $(TipSi)_2 \cdot 3\ C_6H_6$, $\mathbf{4}_2 \cdot 3\ C_6H_6$, crystallised with a full molecule (dimer) in the asymmetric unit. The structure shows disorder in three isopropyl groups which was modelled using two positions and refined with geometric and thermal restraints. Geometric and thermal restraints were applied to the refinement of the lattice benzene molecules.

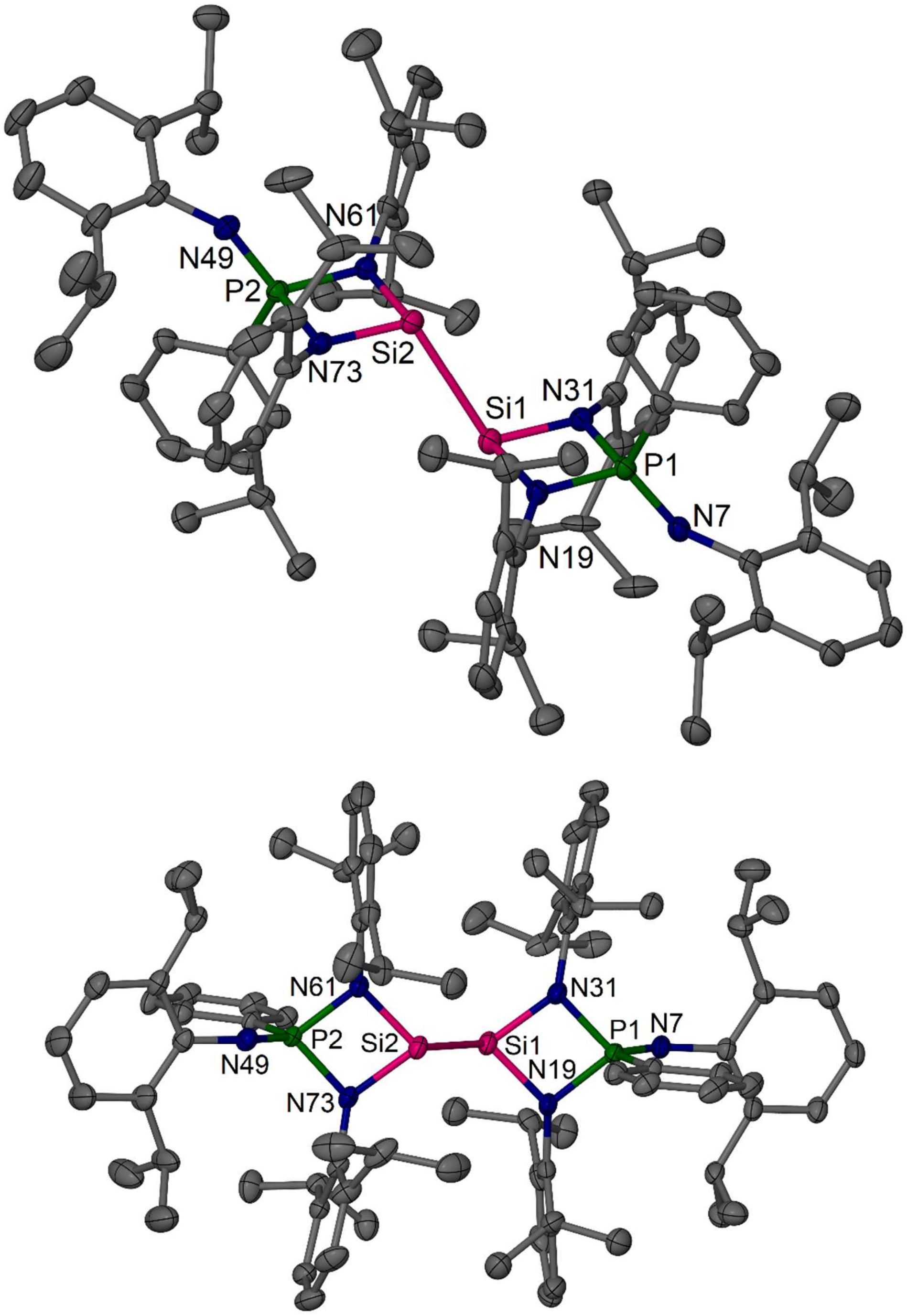

Figure S77. The molecular structure of $(TipSi)_2 \cdot 3\ C_6H_6$, $\mathbf{4}_2 \cdot 3\ C_6H_6$, shown as 30% thermal ellipsoids. Hydrogen atoms, the minor component of disorder, and solvent molecules are omitted for clarity. Selected bond lengths (Å) and angles (°): Si1–Si2 2.6879(13), Si1–N19 1.762(3), Si1–N31 1.748(3),

Si2–N61 1.751(3), Si2–N73 1.748(3), P1–N7 1.516(4), P1–N19 1.704(3), P1–N31 1.697(3), P1–C1 1.810(4), P2–N49 1.515(3), P2–N61 1.702(3), P2–N73 1.705(3), P2–C43 1.811(4), N7–C7 1.404(5); N31–Si1–N19 82.01(15), N73–Si2–N61 82.56(14), N7–P1–N19 119.54(17), N7–P1–N31 116.95(18), N31–P1–N19 85.24(16), N7–P1–C1 114.94(18), N19–P1–C1 107.78(18), N31–P1–C1 108.49(17), N49–P2–N61 116.96(17), N49–P2–N73 119.34(17), N61–P2–N73 85.33(15), N49–P2–C43 114.48(18), N61–P2–C43 109.04(17), N73–P2–C43 108.02(16), C7–N7–P1 147.0(3), C49–N49–P2 149.3(3), ϑ 56.76° (around Si1), 55.43° (around Si2), ϑ (mean) ca. 56.1°.

[(TipH)Li(IPr)]·C_6H_{14}, and [(TipH)Li(IPr)]·2 C_6H_6

Two different solvates of [(TipH)Li(IPr)] were afforded and both crystallised with one main molecule in the asymmetric unit. The hexane solvate shows significant orientational disorder in the hexane molecule which was modelled using two positions, the occupancy of each pair of positions summing to 0.5, and were refined with geometric and thermal restraints. The benzene solvate shows disorder in two isopropyl groups, and in the terminal methyl of another isopropyl group, which were modelled using two positions for all relevant atoms in each group (as well as two atoms of the adjacent phenyl ring in one case), using geometric and thermal restraints. One of the benzene solvates is disordered over a symmetry element and was placed with FragmentDB[23,24] using an idealised geometry and refined with an occupancy fixed at 0.5, and with strong geometric and thermal restraints.

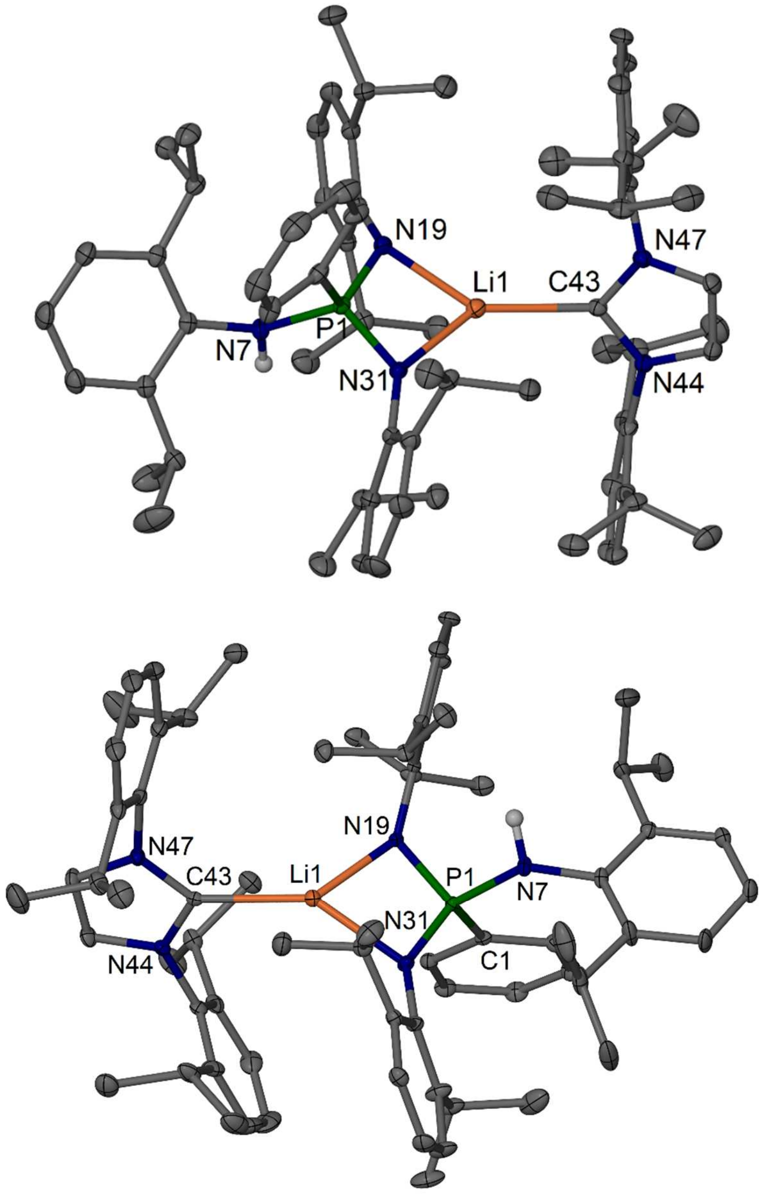

Figure S78. The molecular structures of [(TipH)Li(IPr)]·C_6H_{14} (top) and [(TipH)Li(IPr)]·2 C_6H_6 (bottom) shown as 30% thermal ellipsoids, and in different orientations. Hydrogen atoms except the NH hydrogen, the minor components of disorder, and solvent molecules are omitted for clarity. Selected bond lengths (Å) and angles (°): Top molecule: P1–N7 1.6944(15), P1–N19 1.5941(15), P1–N31 1.6034(14), P1–C1 1.8174(18), N7–C7 1.434(2), P1–Li1 2.606(3), N19–Li1 2.090(3), N31–Li1 2.022(3), C43–Li1 2.266(3); N19–P1–N7 114.52(8), N31–P1–N7 113.94(8), N19–P1–N31 103.62(7), N7–P1–C1 102.23(8), N19–P1–C1 111.52(8), N31–P1–C1 111.29(8), C7–N7–P1 134.16(12), N31–Li1–N19 75.34(12), N31–Li1–C43 137.71(16), N19–Li1–C43 145.13(16). Bottom molecule: P1–N7 1.6992(12), P1–N19 1.6035(11), P1–N31 1.5858(12), P1–C1 1.8283(14), N7–C7 1.4418(18), N19–Li1 1.971(3), N31–Li1 2.157(3), C43–Li1 2.232(3); N19–P1–N7 105.58(6), N31–P1–N7 120.56(7), N31–P1–N19 103.47(6), N7–P1–C1 107.24(6), N19–P1–C1 110.04(6), N31–P1–

C1 109.59(6), C7–N7–P1 138.61(11), N19–Li1–N31 74.52(9), N19–Li1–C43 136.47(13), N31–Li1–C43 145.40(13).

$[(TipH)Li\{N(Dip)PN(Ar')Li\}]\cdot 2\ C_6H_{14}$, Ar' = 2-*i*Pr-6($Me_2PhC$)$C_6H_3$

A crystal was obtained from an intractable mixture of products from the reaction of $TipLi_2$ and $SiBr_4$. This compound crystallised with a full main molecule in the asymmetric unit. Similar to the constitution of compound [(TipH)Li(IPr)], a TipHLi unit coordinates to a neutral molecule, here DipN=P-N(Li)Ar', where the Ar' group is derived from a Dip substituent where a central isopropyl hydrogen atom is substituted with a Ph group (that is missing on P2). The structure shows disorder in two isopropyl groups which was modelled using two positions. One of these was refined isotropically for the minor component with distance restraints. One of the *n*-pentane solvate molecules also shows disorder which was modelled using two positions and refined with geometric and thermal restraints.

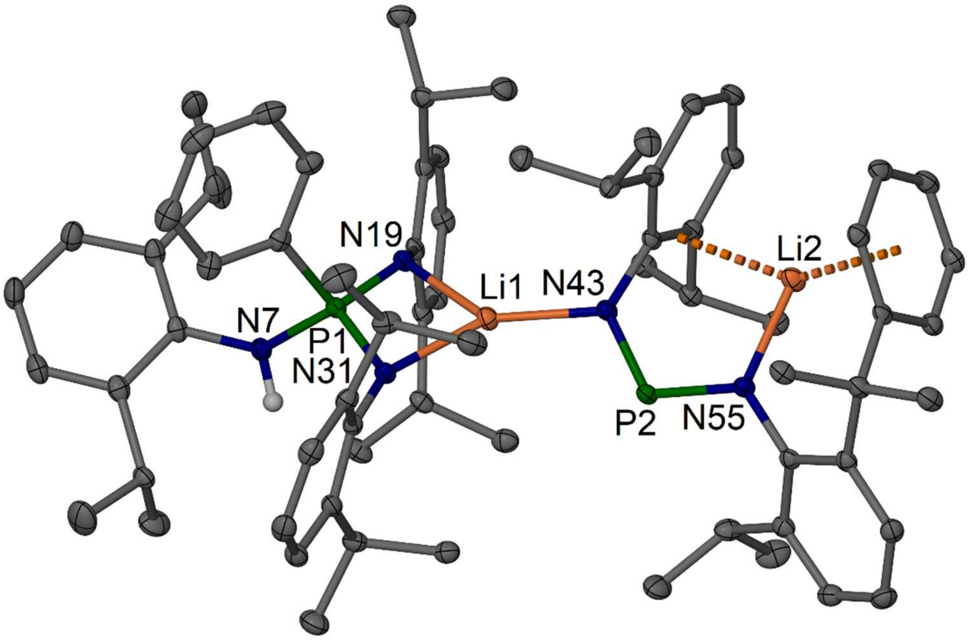

Figure S79. The molecular structure of $[(TipH)Li\{N(Dip)PN(Ar')Li\}]\cdot 2\ C_6H_{14}$ shown as 30% thermal ellipsoids. Hydrogen atoms except the NH hydrogen, the minor component of disorder, and solvent molecules are omitted for clarity. Selected bond lengths (Å) and angles (°): P1–N7 1.6989(14), P1–N19 1.5946(13), P1–N31 1.6100(13), N7–C7 1.441(2), P1–C1 1.8263(17), P1–Li1 2.543(3), P2–N43 1.5992(14), P2–N55 1.6147(13), P2–Li1 3.017(3), P2–Li2 3.117(3), C43–Li2 2.463(3), C48–Li2 2.421(3), C62–Li2 2.365(3), C63–Li2 2.444(3), C64–Li2 2.640(3), C65–Li2 2.744(3), C66–Li2 2.676(3), C67–Li2 2.499(3); N19–P1–N7 116.31(7), N31–P1–N7 112.24(7), N19–P1–N31 104.30(7), N7–P1–C1 101.81(7), N19–P1–C1 111.37(7), N31–P1–C1 111.01(7), C7–N7–P1 132.69(12), N31–Li1–N43 145.79(16), N31–Li1–N19 77.58(11), N43–Li1–N19 136.56(15), N43–P2–N55 110.15(7), N43–P2–N55 110.15(7), N43–P2–Li1 37.76(7), N55–P2–Li1 146.90(7), N43–P2–Li2 75.89(7), N55–P2–Li2 34.40(7), P2–N55–Li2 118.52(11).

TipGe **5**

Compound TipGe **5** crystallised with one monomer in the asymmetric unit and is arranged in the solid state as a weakly bonded dimer, $\mathbf{5}_2$, with highly comparable overall structure to that of the solvated structures of compound $\mathbf{4}_2$. The structure shows disorder in one isopropyl group, which was modelled using two positions and was refined using distance restraints.

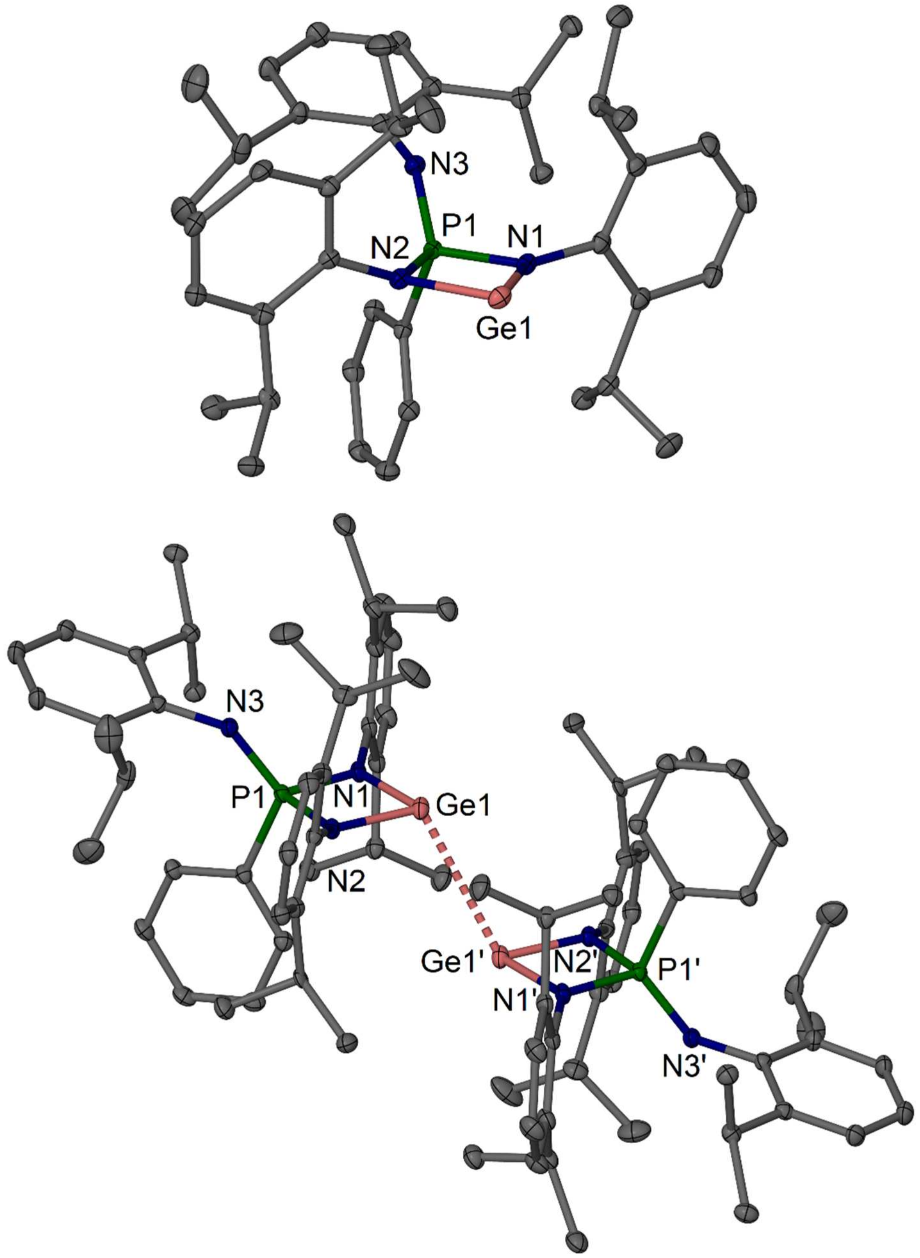

Figure S80. The molecular structure of TipGe **5** shown as 30% thermal ellipsoids. Hydrogen atoms and the minor component of disorder are omitted for clarity. Top: monomeric unit. Bottom: dimeric unit. Selected bond lengths (Å) and angles (°): Ge····Ge 3.1453(10), Ge1–N1 1.9044(19), Ge1–N2 1.8503(19), Ge1···P1 2.6080(8), P1–N1 1.6850(19), P1–N2 1.7296(19), P1–N3 1.5661(19), P1–C1 1.802(2), N3–C31 1.382(3); N2–Ge1–N1 81.63(8), N1–P1–N2 91.91(9), N3–P1–N1 113.98(10),

N3–P1–N2 119.73(10), N1–P1–C1 108.04(9), N2–P1–C1 102.43(10), N3–P1–C1 117.35(10), C31–N3–P1 144.07(16), ϑ 59.45.

TipGe·(TipH)Ge(NHDip)·C_5H_{14}, **5**·(TipH)Ge(NHDip)·C_5H_{14}

This molecular structure consists of two Ge-containing molecules plus lattice *n*-pentane. One of these is TipGe **5**, which, as in the previous structure, crystallised with one monomer in the asymmetric unit and is arranged in the solid state as a weakly bonded dimer. The other molecule is (TipH)Ge(NHDip), from the putative reaction of TipGe **5** with a DipNH$_2$ impurity. One isopropyl group in **5** and one isopropyl group in (TipH)Ge(NHDip) show disorder and both were modelled using two positions for each atom and were refined using distance restraints. Distance restraints were used in refining the lattice *n*-pentane molecule.

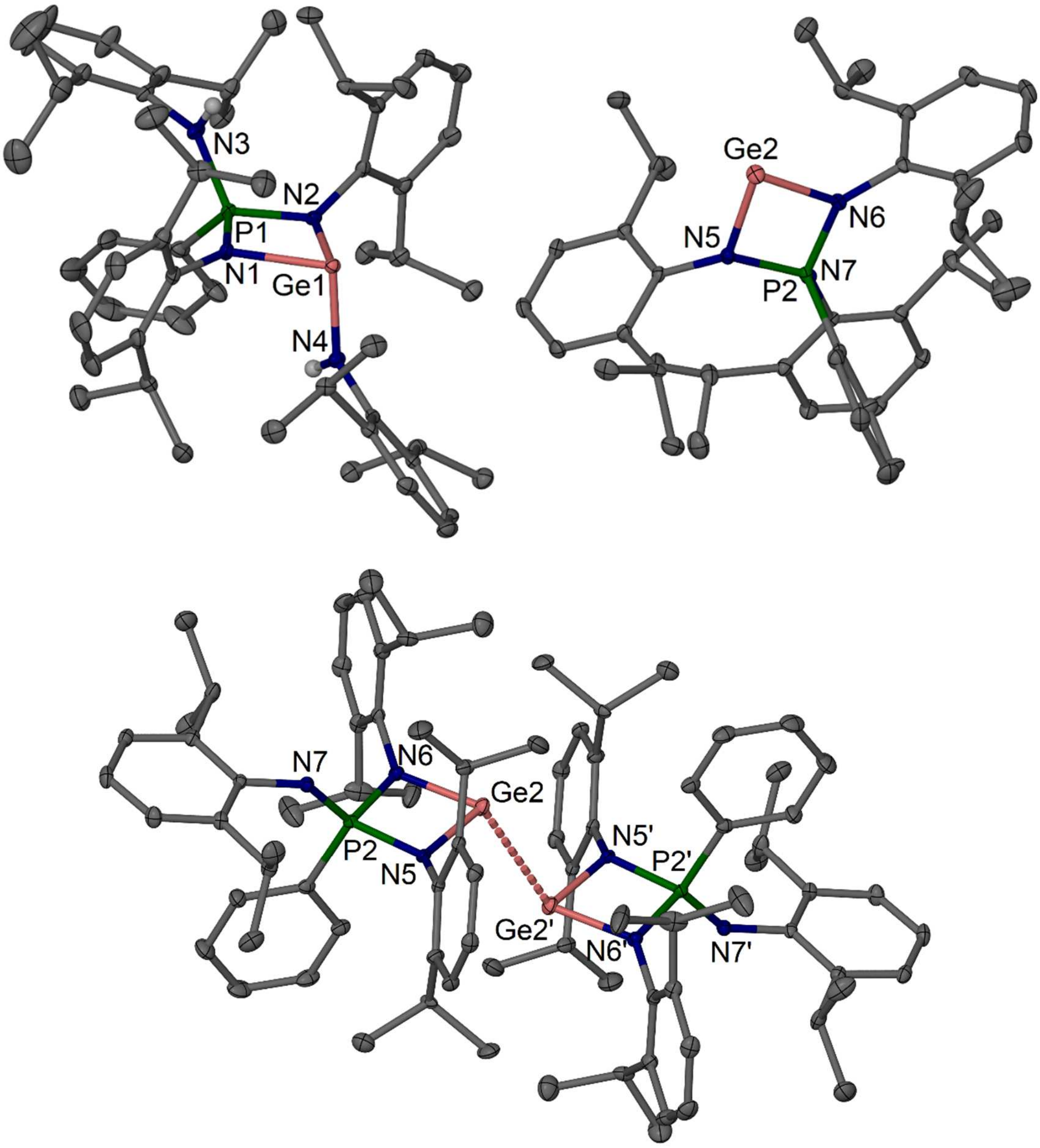

Figure S81. The molecular structure of TipGe·(TipH)Ge(NHDip)·C_5H_{14}, **5**·(TipH)Ge(NHDip)·C_5H_{14} shown as 30% thermal ellipsoids. Hydrogen atoms, except the NH hydrogen, the minor component of disorder, and solvent molecules are omitted for clarity. The two

main molecules (TipH)Ge(NHDip)· (left) and **5**· (right) are shown at the top as they pack in the lattice next to each other. The bottom image shows the dimerization in **5**$_2$. Selected bond lengths (Å) and angles (°): Top left, (TipH)Ge(NHDip): Ge1–N4 1.8992(17), Ge1–N2 2.0464(17), Ge1–N1 2.0533(17), Ge1–P1 2.7666(6), P1–N1 1.5998(18), P1–N2 1.6347(18), P1–N3 1.6493(18), P1–C1 1.812(2), N3–C31 1.448(3); N4–Ge1–N2 98.09(7), N4–Ge1–N1 97.85(7), N2–Ge1–N1 70.83(7), N1–P1–N2 94.53(9), N1–P1–N3 113.54(9), N2–P1–N3 117.10(9), N1–P1–C1 117.14(10), N2–P1–C1 113.14(10), N3–P1–C1 102.23(10), C31–N3–P1 133.64(15); Top right, [(Tip)Ge]: Ge····Ge 3.1655(8), Ge2–N5 1.8766(17), Ge2–N6 1.8768(17), Ge2–P2 2.6764(6), P2–N7 1.5293(18), P2–N5 1.6875(17), P2–N6 1.6922(18), P2–C55 1.822(2), N7–C85 1.407(3); N5–Ge2–N6 77.59(7), N7–P2–N5 117.50(9), N7–P2–N6 117.19(9), N5–P2–N6 88.19(8), N7–P2–C55 114.71(10), N5–P2–C55 106.66(9), N6–P2–C55 109.38(9), C85–N7–P2 141.03(15), ϑ ca. 60.85.

TipHGeCl

This compound crystallised with a full molecule in the asymmetric unit.

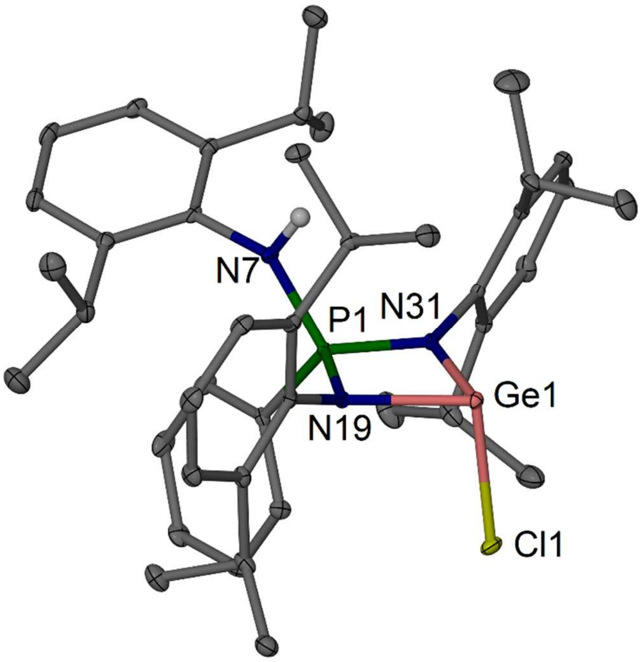

Figure S82. The molecular structure of TipHGeCl shown as 30% thermal ellipsoids. Hydrogen atoms except the NH hydrogen are omitted for clarity. Selected bond lengths (Å) and angles (°): Ge1–Cl1 2.3184(4), Ge1–N19 1.9932(11), Ge1–N31 2.0019(11), P1–N7 1.6443(12), P1–N19 1.6291(11), P1–N31 1.6317(11), P1–C1 1.8084(13), N7–C7 1.4520(17); N19–Ge1–N31 72.75(4), N19–Ge1–Cl1 97.00(3), N31–Ge1–Cl1 98.93(3), N7–P1–C1 106.97(6), N19–P1–N7 112.71(6), N19–P1–N31 93.21(6), N19–P1–C1 113.77(6), N31–P1–N7 116.07(6), N31–P1–C1 113.94(6), C7–N7–P1 134.83(9).

(TipHGe)$_2$O

This compound crystallised with half a molecule in the asymmetric unit.

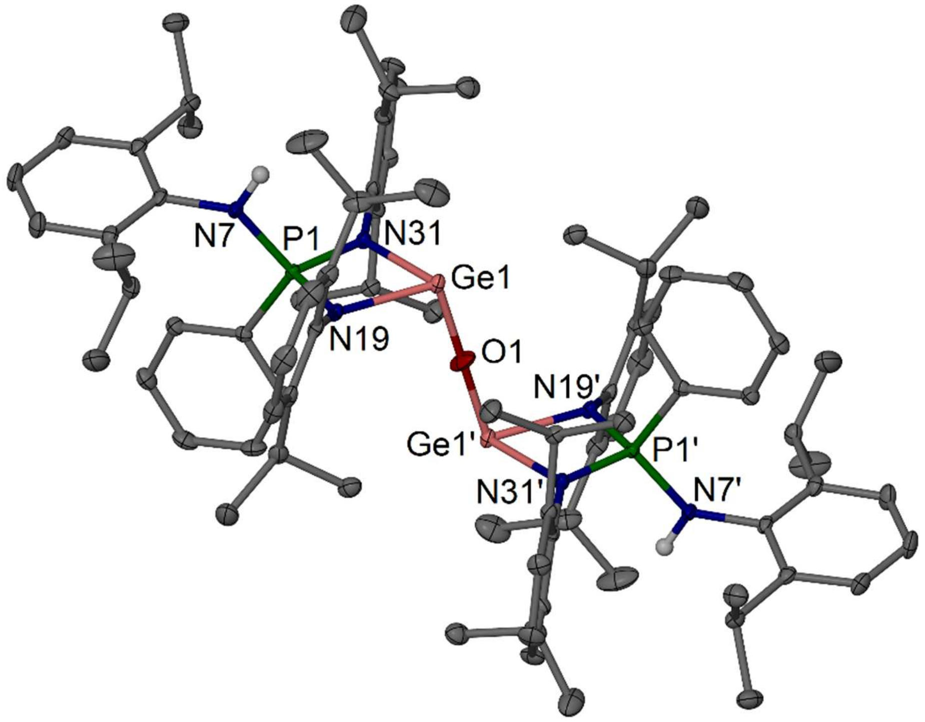

Figure S83. The molecular structure of (TipHGe)$_2$O shown as 30% thermal ellipsoids. Hydrogen atoms except the NH hydrogens are omitted for clarity. Selected bond lengths (Å) and angles (°): Ge1–O1 1.77811(19), Ge1–N19 2.0639(11), Ge1–N31 2.0671(11), Ge1–P1 2.7718(4), P1–N31 1.6154(11), P1–N19 1.6179(12), P1–N7 1.6546(12), P1–C1 1.8196(14), N7–C7 1.4511(17); Ge1–O1–Ge1' 180.0, N19–Ge1–N31 70.76(4), O1–Ge1–N19 100.48(3), O1–Ge1–N31 99.68(3), N19–P1–N7 114.58(6), N31–P1–N7 114.29(6), N31–P1–N19 95.43(6), N7–P1–C1 107.62(6), N19–P1–C1 111.94(6), N31–P1–C1 112.80(6), C7–N7–P1 134.23(10).

TipSn·1.5 C_6H_6, **6**·1.5 C_6H_6

Complex **6**·1.5 C_6H_6 crystallised with a full complex molecule in the asymmetric unit. The benzene lattice molecules were refined using geometric and thermal restraints. The molecule packs as a weakly bonded coordination polymer $(TipSn)_\infty$, $\mathbf{6}_\infty$.

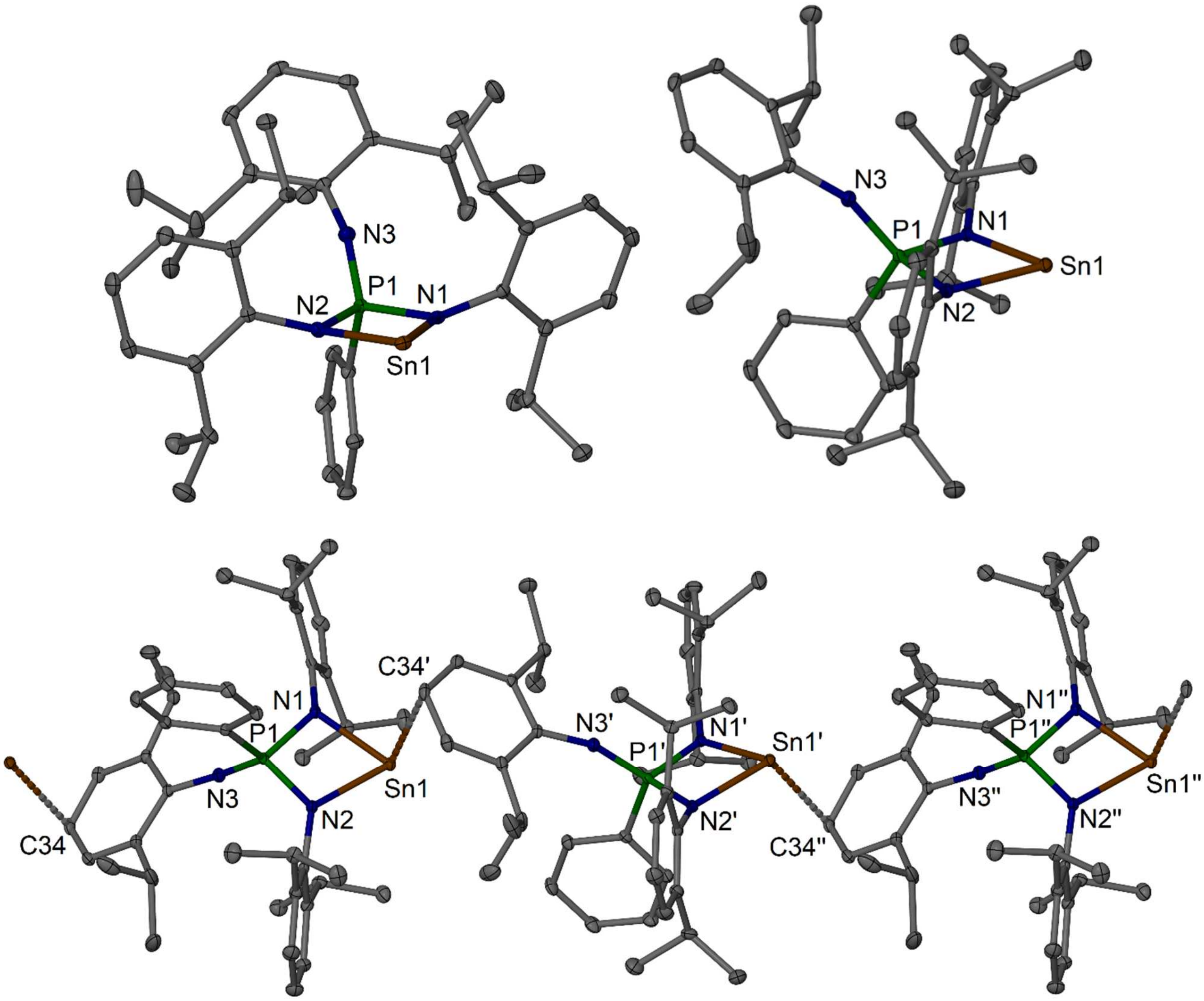

Figure S84. The molecular structure of TipSn·1.5 C_6H_6, **6**·1.5 C_6H_6, shown as 30% thermal ellipsoids. Hydrogen atoms and solvent molecule are omitted for clarity. Top: monomeric unit (in two views); bottom: one-dimensional polymer. Selected bond lengths (Å) and angles (°): Sn1–N1 2.1380(18), Sn1–N2 2.136(2), Sn1–P1 2.9100(8), need Sn1···C34' 2.793(2), P1–N1 1.6646(18), P1–N2 1.662(2), P1–N3 1.5346(19), P1–C1 1.822(2), N3–C31 1.356(3), C31–C36 1.435(3), C31–C32 1.437(3), C32–C33 1.398(3), C33–C34 1.391(3), C34–C35 1.397(3), C35–C36 1.386(3); N2–Sn1–N1 68.60(7), N2–P1–N1 92.75(9), N3–P1–N1 116.90(9), N3–P1–N2 119.65(9), N1–P1–C1 108.94(9), N2–P1–C1 108.30(9), N3–P1–C1 109.07(10), C31–N3–P1 161.27(16), C36–C31–C32

117.71(18), C33–C32–C31 119.5(2), C34–C33–C32 122.0(2), C33–C34–C35 118.5(2), C36–C35–C34 121.9(2), C35–C36–C31 120.04(19).

TipPb·C_6H_6, **7**·C_6H_6

Complex **7**·C_6H_6 crystallised with a full complex molecule in the asymmetric unit. The molecule packs as a weakly bonded coordination polymer $(TipPb)_\infty$, $\mathbf{7}_\infty$.

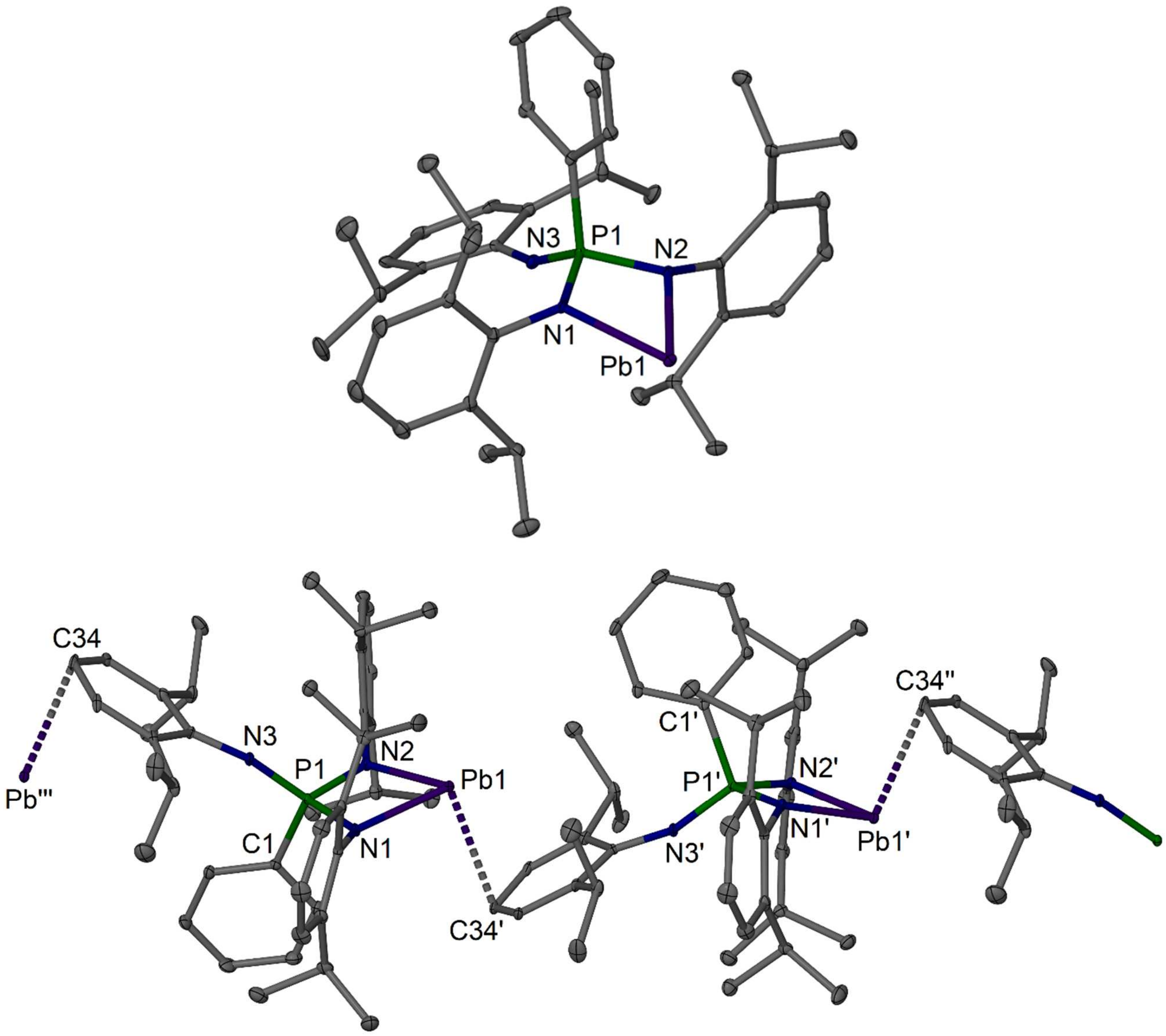

Figure S85. The molecular structure of TipPb·C_6H_6, **7**·C_6H_6, shown as 30% thermal ellipsoids. Hydrogen atoms and solvent molecule are omitted for clarity. Top: monomeric unit; bottom: one-dimensional polymer. Selected bond lengths (Å) and angles (°): Pb1–N1 2.230(3), Pb1–N2 2.257(3), Pb1···C34' 2.826(4), P1–N3 1.553(3), P1–N2 1.657(3), P1–N1 1.658(3), P1–C1 1.834(4), N3–C31 1.343(4), C31–C32 1.438(5), C31–C36 1.442(5), C32–C33 1.387(5), C33–C34 1.393(6), C34–C35 1.387(6), C35–C36 1.395(5); N1–Pb1–N2 65.82(11), N1–Pb1–C34' 90.70(11), N2–Pb1–C34' 104.04(11), N2–P1–N1 94.66(16), N3–P1–N2 120.67(16), N3–P1–N1 116.20(16), N1–P1–C1 108.61(16), N2–P1–C1 107.66(15), N3–P1–C1 108.02(17), C7–N1–P1 127.1(2), C31–N3–P1

160.4(3), C32–C31–C36 117.1(3), C33–C32–C31 119.9(4), C32–C33–C34 122.3(4), C35–C34–C33 118.3(3), C34–C35–C36 122.3(4), C35–C36–C31 119.6(3).

TipSi(DMAP)·C_6H_6·0.25 C_6H_{14}, **9**·C_6H_6·0.25 C_6H_{14}

Compound **9**·C_6H_6·0.25 C_6H_{14} crystallised with two full main molecules in the asymmetric unit. The structure shows rotational disorder in one benzene solvate which was modelled using two positions and refined with geometric and thermal restraints. Furthermore, diffuse electron density from disordered solvent in void spaces has been removed using the SQUEEZE[19] routine.

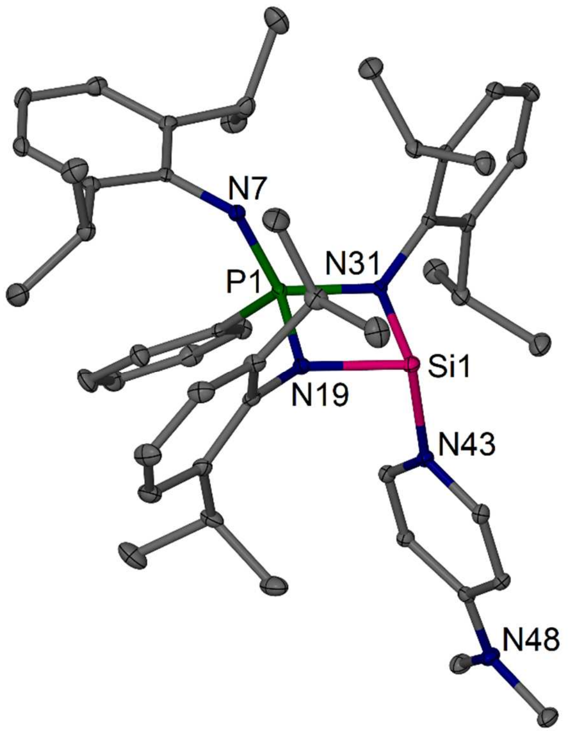

Figure S86. The molecular structure of TipSi(DMAP)·C_6H_6·0.25 C_6H_{14}, **9**·C_6H_6·0.25 C_6H_{14}, shown as 30% thermal ellipsoids. Only one of two independent molecules is shown but bond distances and angles are given for both molecules. Hydrogen atoms, the minor component of disorder, and solvent molecules are omitted for clarity. Selected bond lengths (Å) and angles (°): shown molecule: Si1–N19 1.8126(16), Si1–N31 1.8338(17), Si1–N43 1.9499(16), P1–N7 1.5522(16), P1–N19 1.6711(17), P1–N31 1.6824(16), P1–C1 1.819(2), N7–C7 1.414(2); from second molecule: Si2–N68 1.8278(16), Si2–N80 1.8139(16), Si2–N92 1.9706(16), P2–N68 1.6754(15), P2–N56 1.5524(16), P2–N80 1.6749(16), P2–C50 1.819(2), N56–C56 1.413(2); shown molecule: N19–Si1–N31 78.54(7), N19–Si1–N43 99.66(7), N31–Si1–N43 100.15(7), N7–P1–N19 118.71(9), N7–P1–N31 119.56(8), N7–P1–C1 107.88(9), N19–P1–N31 86.98(8), N19–P1–C1 109.97(8), N31–P1–C1 112.49(9), C7–N7–P1 127.80(13); from second molecule: N80–Si2–N68 78.12(7), N68–Si2–N92 99.38(7), N80–Si2–N92 100.94(7), N56–P2–N68 119.31(8), N56–P2–N80 118.93(9), N68–P2–C50 113.65(9), N56–P2–C50 107.77(9), N80–P2–N68 86.46(8), N80–P2–C50 109.44(8), C56–N56–P2 126.95(14).

TipSi(CPh)$_2$ **10**

Compound **10** crystallised with two full molecules in the asymmetric unit. The structure shows overall poor ordering including disorder in various phenyl and diisopropylphenyl groups, which were modelled using two positions and refined with strong geometric and thermal restraints. Diffuse electron density in void spaces has been removed using the SQUEEZE[19] routine. Much of the rest of the structure showed signs of apparent disorder, possibly dynamic and associated with the void-spaces, or associated with unaccounted-for additional twinning. Thus, bond distances and angles should not be overinterpreted.

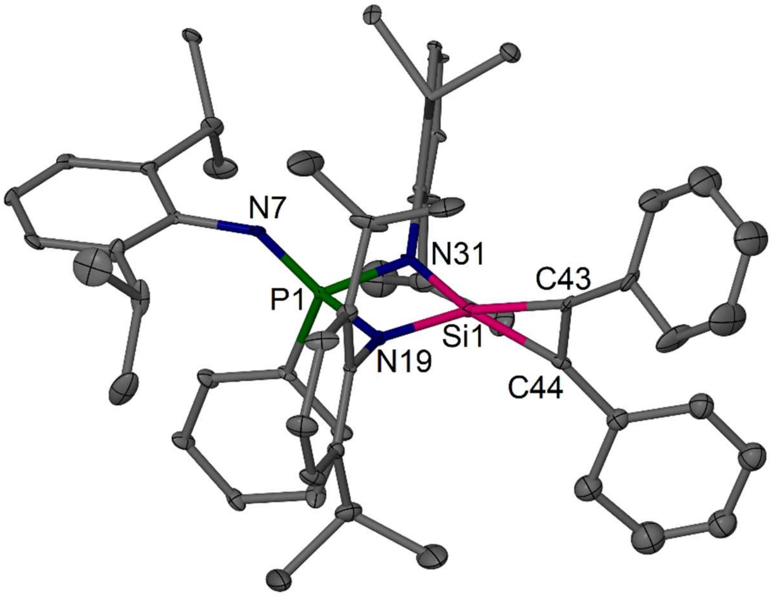

Figure S87. The molecular structure of TipSi(CPh)$_2$ **10** shown as 30% thermal ellipsoids. Only one of two independent molecules is shown. Hydrogen atoms and minor components of disorder are omitted for clarity. Due to more severe disorder in the second molecule, only metric data for the shown molecule are given. Selected bond lengths (Å) and angles (°): Si1–N19 1.681(6), Si1–N31 1.742(5), Si1–C43 1.800(7), Si1–C44 1.762(8), P1–N7 1.519(6), P1–N19 1.695(5), P1–N31 1.694(6), P1–C1 1.801(7), N7–C7 1.415(8); N19–Si1–N31 83.2(3), N19–Si1–C43 131.6(3), N19–Si1–C44 132.6(3), N31–Si1–C43 139.7(3), N31–Si1–C44 129.8(3), C44–Si1–C43 45.5(3), C7–N7–P1 137.5(5), C44–C43–Si1 65.8(4), C45–C43–Si1 159.1(6), C43–C44–Si1 68.7(4), C51–C44–Si1 157.5(6), C44–C43–C45 134.7(6), C43–C44–C51 133.7(7).

TipSi{$CH_2C(Me)$}$_2$ **11**

Compound **11** crystallised with a full molecule in the asymmetric unit. The ligand shows poor overall ordering, but the organosilicon unit shows much better ordering. The structure shows disorder in all three N-diisopropylphenyl groups and the P-phenyl, and where possible these were split into two parts and refined with strong geometric and thermal restraints. The parts that did not allow stable refinement with split positions were refined with some restraints. This behaviour suggests the possibility that the disorder in this structure is dynamic, rather than static.

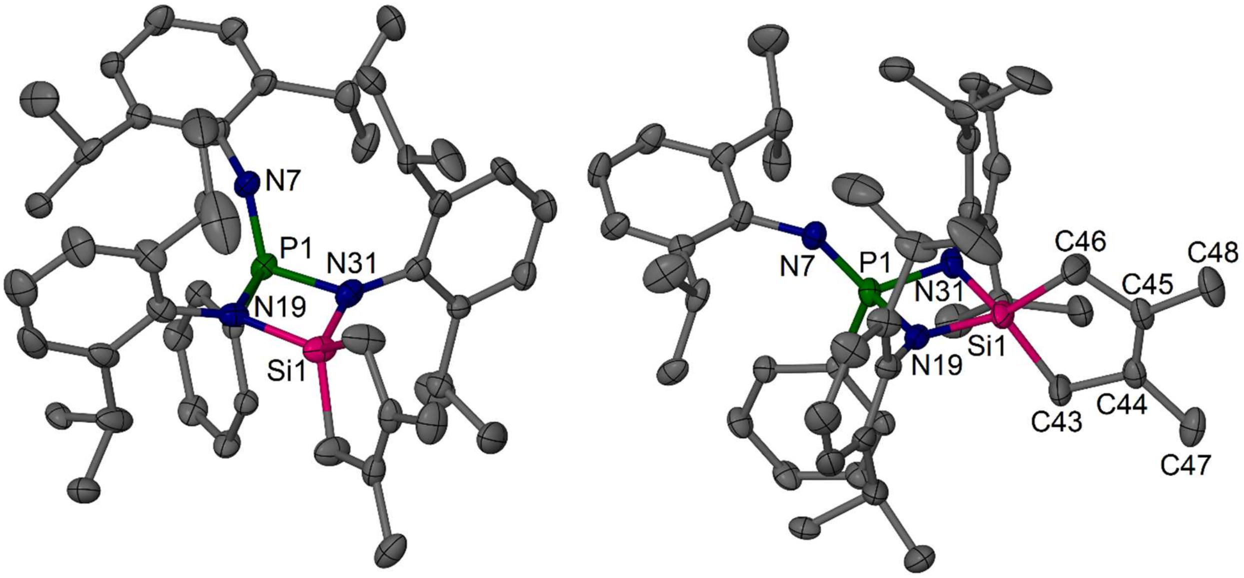

Figure S88. The molecular structure of TipSi{$CH_2C(Me)$}$_2$ **11** shown as 30% thermal ellipsoids in two views. Hydrogen atoms and minor components of disorder are omitted for clarity. Selected bond lengths (Å) and angles (°): Si1–N19 1.738(2), Si1–N31 1.743(2), Si1–C43 1.856(3), Si1–C46 1.861(3), P1–N7 1.5259(18), P1–N19 1.6975(18), P1–N31 1.6950(19), P1–C1A 1.874(9), P1–C1B 1.778(11), N7–C7 1.413(3), C43–C44 1.494(3), C44–C45 1.330(4), C44–C47 1.497(4), C45–C46 1.502(4), C45–C48 1.507(3); N19–Si1–N31 82.74(9), N19–Si1–C43 117.11(11), N19–Si1–C46 125.31(14), N31–Si1–C43 120.57(13), N31–Si1–C46 119.11(13), C43–Si1–C46 94.82(12), C44–C43–Si1 104.85(18), C45–C46–Si1 104.15(18), N7–P1–N19 118.43(9), N7–P1–N31 118.46(10), N7–P1–C1A 117.7(4), N7–P1–C1B 109.6(5), N19–P1–C1A 102.4(3), N19–P1–C1B 116.5(5), N31–P1–N19 85.40(9), N31–P1–C1A 109.2(4), N31–P1–C1B 106.2(5), C7–N7–P1 140.43(18), C43–C44–C47 116.0(2), C45–C44–C43 117.7(2), C45–C44–C47 126.2(2), C44–C45–C46 118.2(2), C44–C45–C48 126.4(3), C46–C45–C48 115.4(2).

Table S3. Selected bond lengths and angle in the P=N–C tail of TipE compounds **4-7**.

Structure	Tail P=N [Å]	Tail N–C [Å]	Tail P=N–C [°]
**4**$_2$·C_6H_{14}	P1–N7 1.5333(15)	N7–C7 1.409(2)	C7–N7–P1 136.52(12)
**4**$_2$·3 C_6H_6	P1–N7 1.516(4),	N7–C7 1.404(5),	C7–N7–P1 147.0(3),
	P2–N49 1.515(3)	N49–C49 1.389(5)	C49–N49–P2 149.3(3)
**5**	P1–N3 1.5661(19)	N3–C31 1.382(3)	C31–N3–P1 144.07(16)
**5** in **5**·(TipH)Ge(NHDip)·C_5H_{14}	P2–N7 1.5293(18)	N7–C85 1.407(3)	C85–N7–P2 141.03(15)
**6**$_\infty$, in **6**·1.5 C_6H_6	P1–N3 1.5346(19)	N3–C31 1.356(3)	C31–N3–P1 161.27(16)
**7**$_\infty$, in **7**·C_6H_6	P1–N3 1.553(3)	N3–C31 1.343(4)	C31–N3–P1 160.4(3)

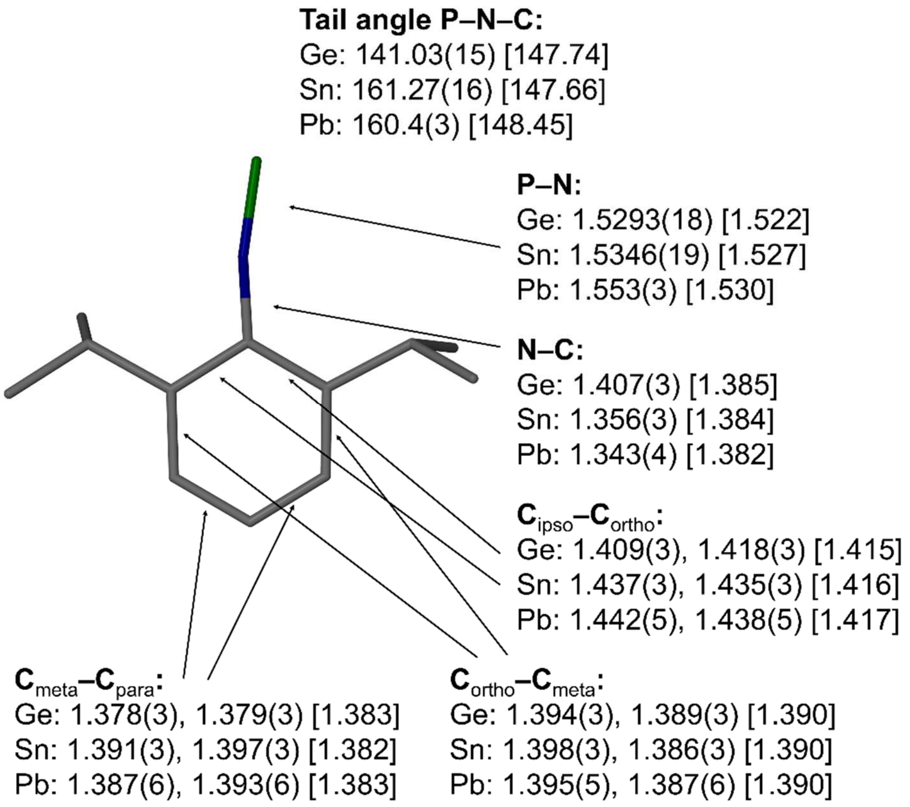

Figure S89. Comparison of tail PNDip geometry (angle in [°], distances in [Å]) for **5** (**5**·(TipH)Ge(NHDip)·C_5H_{14}), **6**$_\infty$ (**6**·1.5 C_6H_6) and **7**$_\infty$ (**7**·C_6H_6) as determined by X-ray diffraction. For **5**, the molecular structure of **5**·(TipH)Ge(NHDip)·C_5H_{14} was chosen due to the longer Ge–Ge distance and the more even C–C bond lengths of the two halves of the DipN tail compared with that in TipGe **5**. The values are compared to DFT-optimised data [in square brackets], *vide infra*, for the monomers TipE **5**, **6** and **7** to allow comparison in the PNDip tail between the coordination polymer structures **6**$_\infty$ and **7**$_\infty$ and the molecular entities of **5**-**7**.

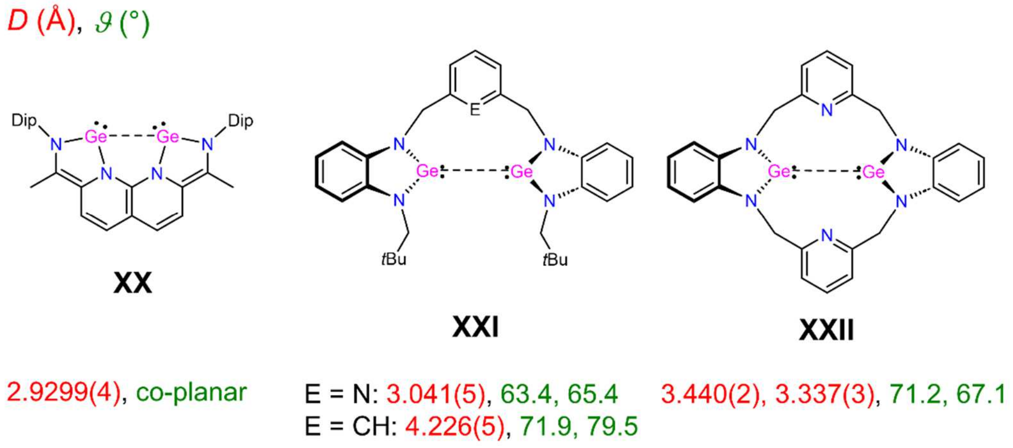

Figure S90. Structurally characterised N-heterocyclic germylene examples with relatively close Ge···Ge interactions and selected metrical parameters; **XX**,[25] **XXI**,[26] **XXII**.[27]

4 DFT Computational Studies

4.1 General considerations

Compounds were optimised from the starting geometries obtained by X-ray diffraction, or derived from those, using the M06-L[28] density functional coupled with the def2-TZVP[29,30] basis set augmented with a D3 dispersion term,[31] followed by single point calculations with the M06 functional[32] with a D3 term and the def2-TZVP basis set in the gas phase. If not stated otherwise, analysis and graphical material is based on results at this level of theory (M06-D3/def2-TZVP//M06-L-D3/def2-TZVP level – in some text and tables abbreviated as M06D3). In addition, some calculations on a cut-back model (e.g. on **4'**$_2$, **5'**$_2$), plus one on **4**$_2$, were performed at the B3LYP[33,34]/def2-TZVP level without dispersion (in some text and tables abbreviated as B3LYP). Counterpoise correction was added to eliminate Basis Set Superposition Error (BSSE).[35] Natural Population Analysis (NPA) charges (e.g. in Table S4, Figure S103) and Wiberg bond indices (WBIs) (e.g. in Table S7) were calculated using Natural Bond Orbital (NBO) analysis,[36] at the respective level. Quantum Theory of Atoms in Molecules (QTAIM) analysis was performed at the M06-D3/def2-TZVP level and the B3LYP/def2-TZVP level using AIMALL.[37] The calculations were performed using Gaussian 16.[38] Non-covalent interactions were studied using NCIPLOT.[39]

Gibbs free energies ΔG_{298} (in kcal mol^{-1}) for the reaction of two monomers to one dimer were determined by calculations at the M06-D3/def2-TZVP//M06-L-D3/def2-TZVP level (Table S5). The singlet-triplet vertical gap, $\Delta E_{s\text{-}t}$ (in kcal mol^{-1}, eV) was determined at the M06-D3/def2-TZVP//M06-L-D3/def2-TZVP level for the Si/Ge monomers and dimers and the singlet-triplet adiabatic gap only for the monomers (Table S6); all other species were calculated as closed-shell singlet-state molecules.

4.2 Silicon compounds

4.2.1 TipSi 4 (monomer)

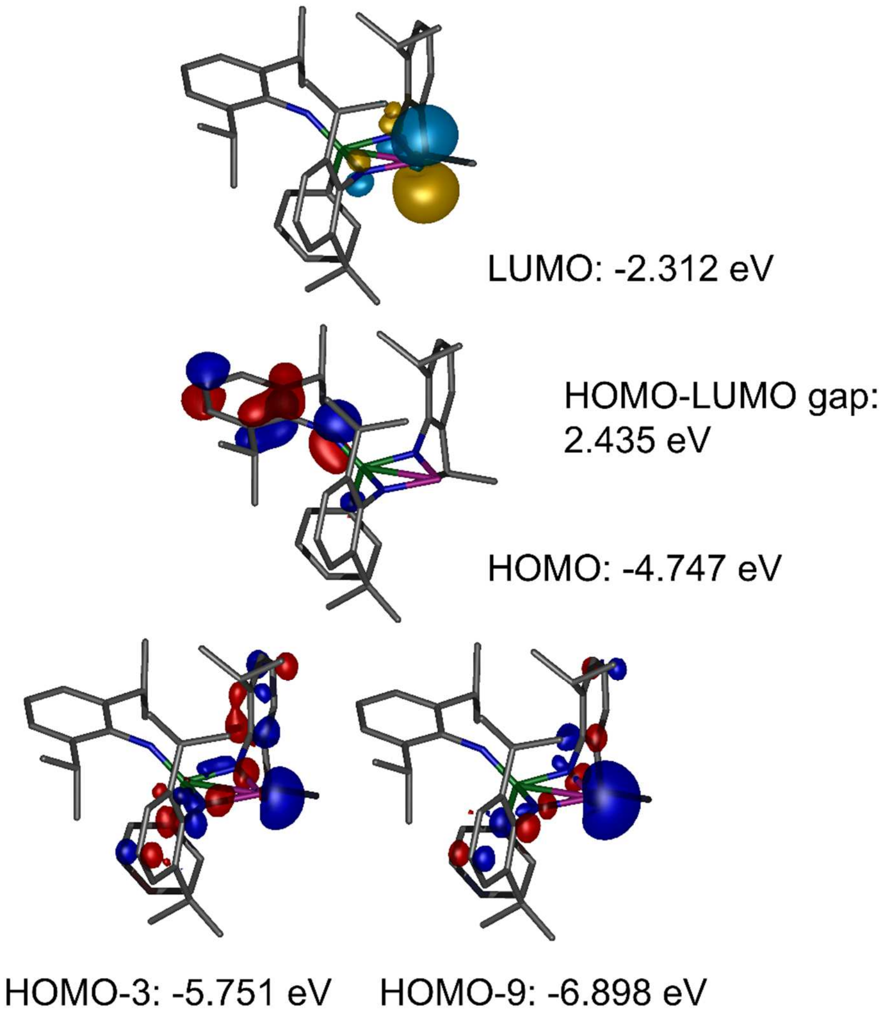

Figure S91. Selected molecular orbitals at the M06-D3/def2-TZVP//M06-L-D3/def2-TZVP level (isovalue 0.06) of TipSi **4**.

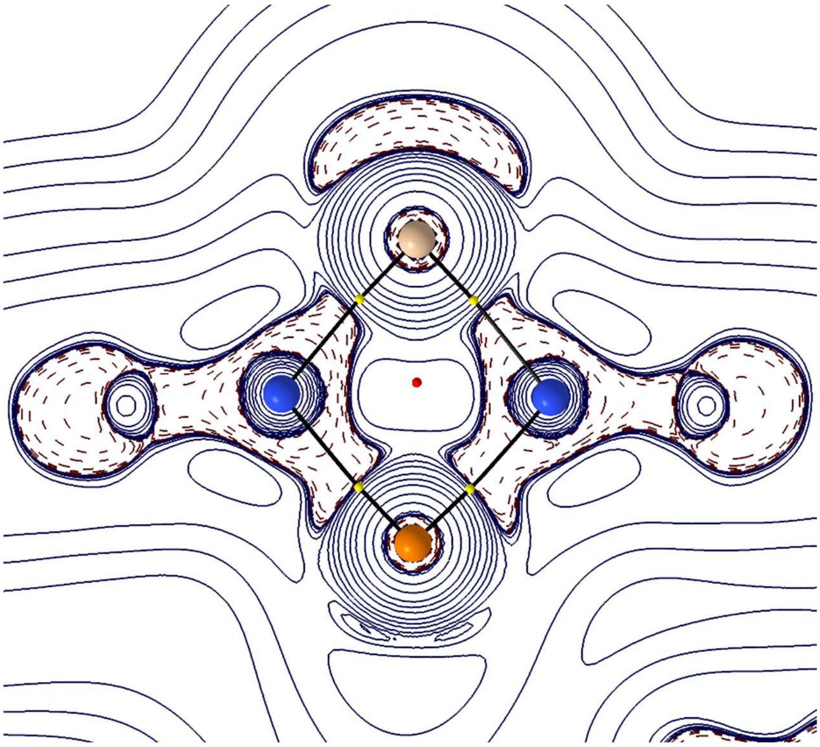

Figure S92. QTAIM contour plots showing the Laplacian of the electron density (solid lines: positive, dashed lines: negative) for TipSi **4** through the PNSi plane. Si at the top, N blue, P orange.

4.2.2 (TipSi)$_2$ **4**$_2$ (dimer)

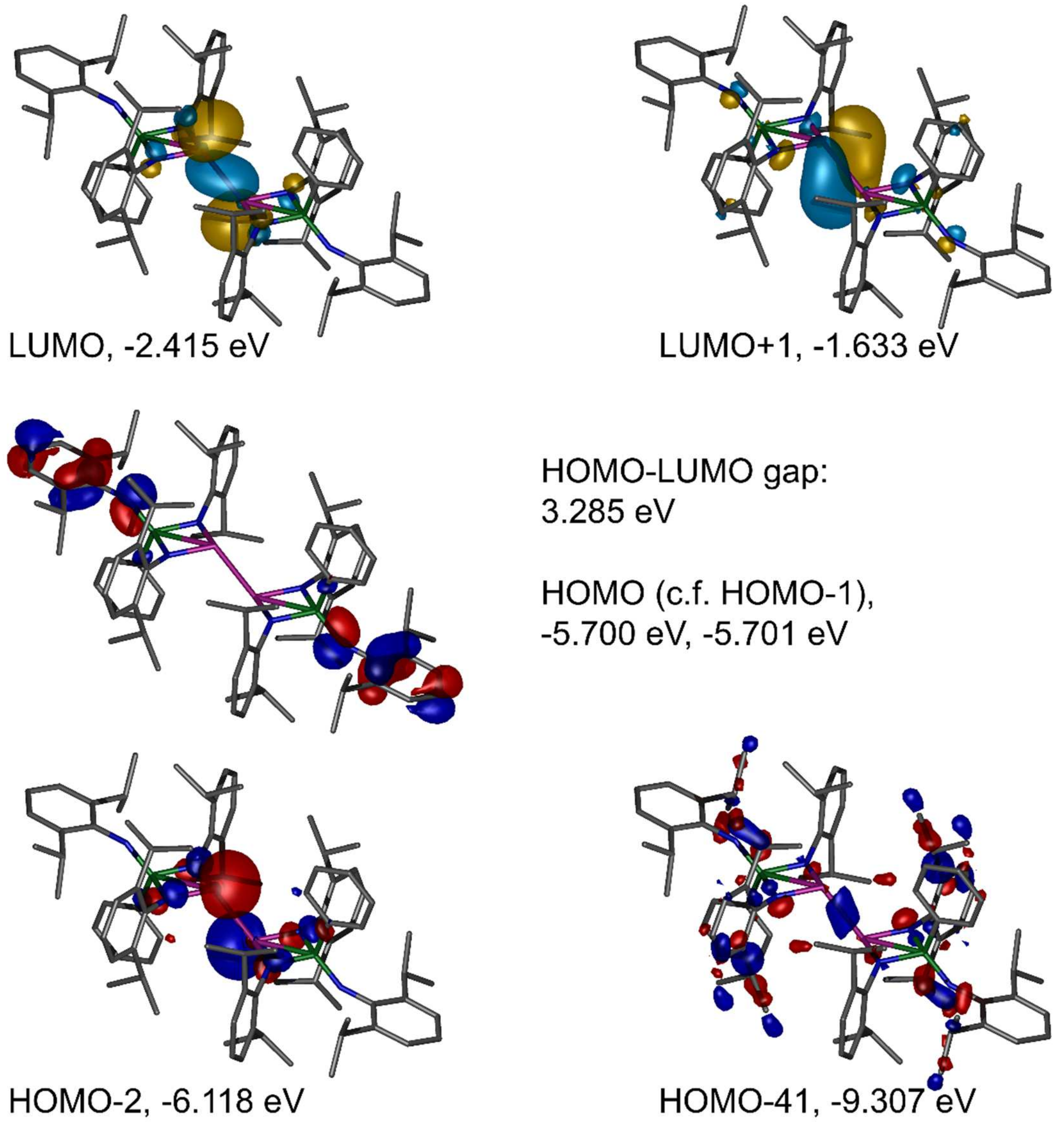

Figure S93. Selected molecular orbitals at the M06-D3/def2-TZVP//M06-L-D3/def2-TZVP level (isovalue 0.04) of (TipSi)$_2$ **4**$_2$.

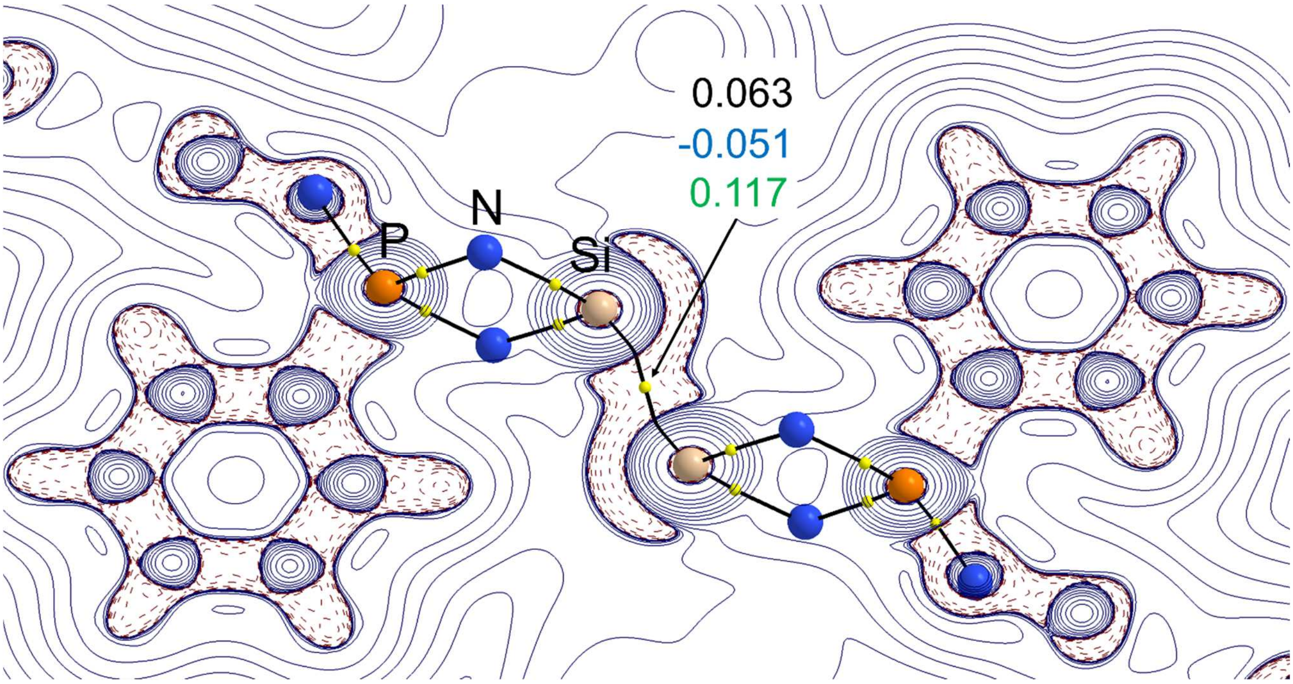

Figure S94. QTAIM contour plots showing the Laplacian of the electron density (solid lines: positive, dashed lines: negative) for **4**$_2$ (through the P-SiSi-plane but angling the N_2Si unit to show bond paths); bond critical points (bcps): yellow, bond paths: black lines, values for the electron density ρ [e bohr^{-3}], (black), Laplacian, $\nabla^2\rho$ [e bohr^{-5}], (blue), and bond ellipticity ε, (green) are given for the bcp between both Si atoms.

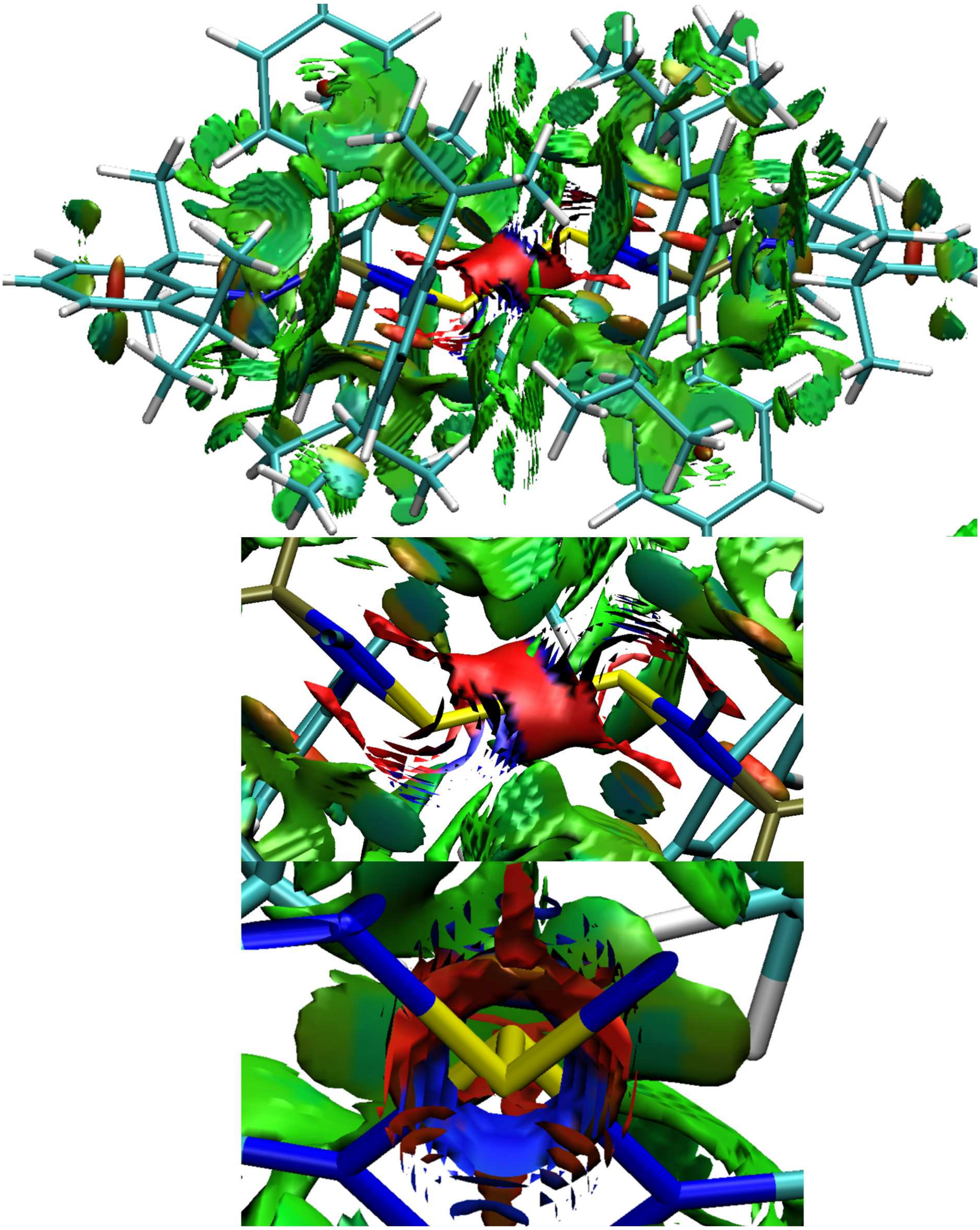

Figure S95. NCIPLOT analysis of the $(TipSi)_2$ $\mathbf{4}_2$ in different views showing the isosurface for s = 0.5 au and colour scale −0.05 < ρ < 0.05 au (green: van der Waals interactions, red: repulsive, blue: attractive interactions).

4.2.3 (Tip’Si)$_2$ 4’$_2$

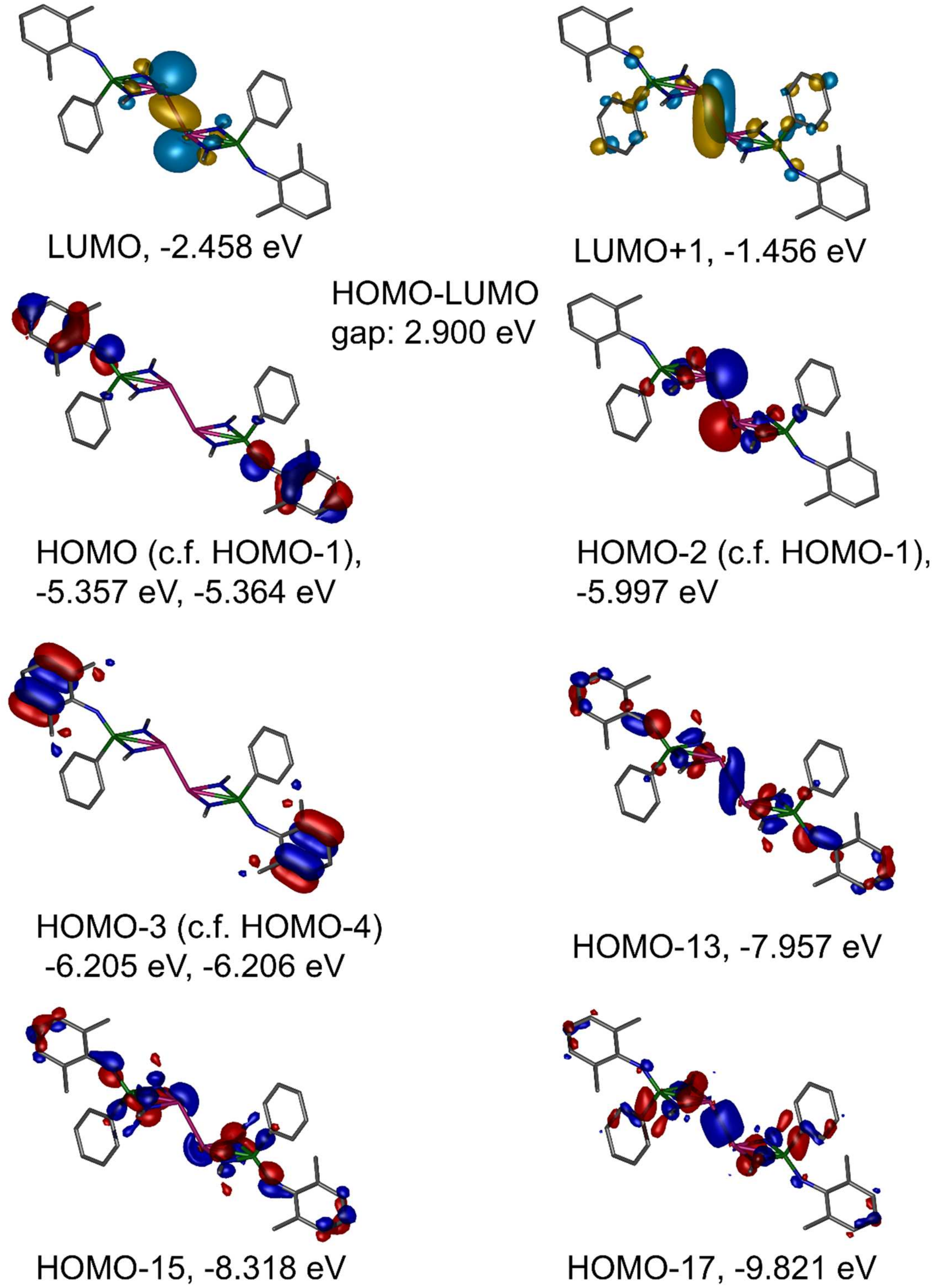

Figure S96. Selected molecular orbitals at the B3LYP/def2-TZVP level (isovalue 0.04) of (Tip’Si)$_2$ **4’**$_2$.

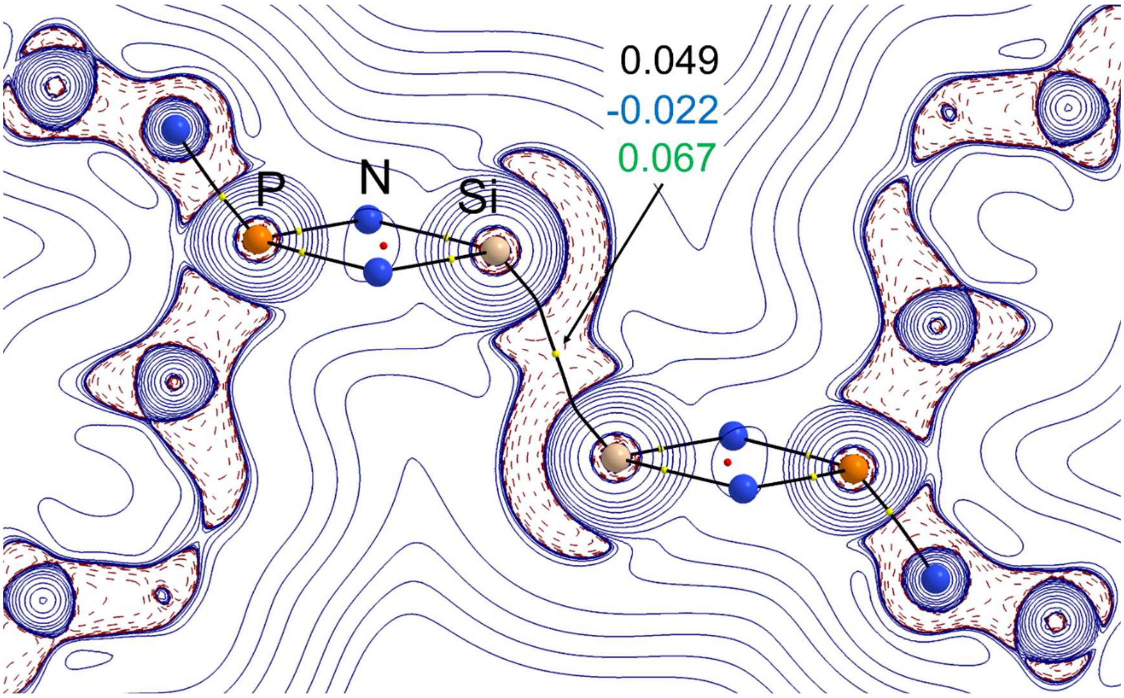

Figure S97. QTAIM contour plots showing the Laplacian of the electron density (solid lines: positive, dashed lines: negative) for **4’**$_2$ (through the P-SiSi-plane but angling the N_2Si unit to show bond paths); bond critical points (bcps): yellow, bond paths: black lines, values for the electron density ρ [e bohr^{-3}], (black), Laplacian, $\nabla^2\rho$ [e bohr^{-5}], (blue), and bond ellipticity ε, (green) are given for the bcp between both Si atoms.

Table S4. Comparison of mean calculated NPA and QTAIM charges for **4**$_2$ and **4’**$_2$.

	**4**$_2$ NPA	**4**$_2$ QTAIM	**4’**$_2$ NPA	**4’**$_2$ QTAIM
Si	+1.253	+1.534	+1.132	+1.508
P	+2.215	+3.298	+2.215	+3.314
N(Si)	-1.280	-1.868	-1.183	-1.849
N(Si)	-1.272	-1.844	-1.174	-1.838
N(P)	-1.086	-1.796	-1.052	-1.797

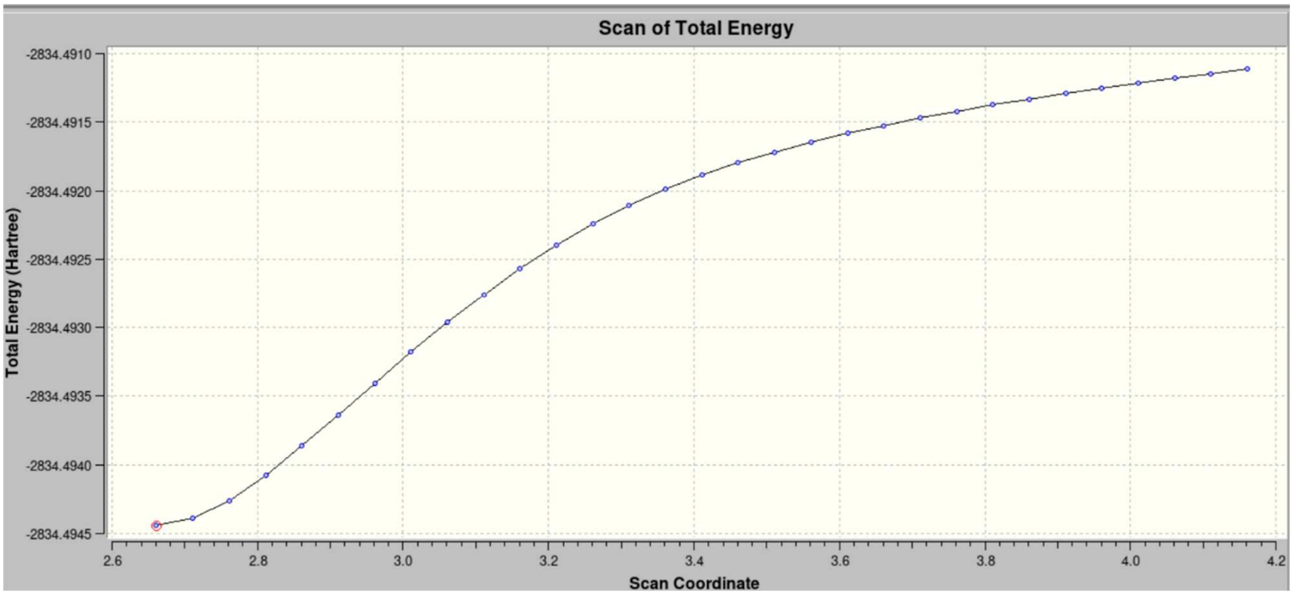

Figure S98. Energy scan (restricted DFT) along the Si····Si interaction in **4'**$_2$ at 0.05 Å intervals (from the bonding distance to approximately 2 Si van der Waals radii), total energy in Hartree versus distance in Å, with geometry optimisation at each step. The difference in energy between minimum and maximum of the shown part of the curve is ca. 2.1 kcal mol^{-1}. (This should only be used as a guide. The scan shows that between the Si–Si bonding distance in **4'**$_2$ and stretching to 2 monomers of **4**, no energy maximum, or barrier, is found.)

4.2.4 {tBuC$_6$H$_4$C(NDip)$_2$Si}$_2$ 8

For comparison to the Si^{II}–Si^{II} bonding in the **4'**$_2$ system, we reoptimized {tBuC$_6$H$_4$C(NDip)$_2$Si}$_2$ **8** at the M06-D3/def2-TZVP//M06-L-D3/def2-TZVP level because a molecular structure from X-ray diffraction (Si–Si: 2.4885(15) Å) and DFT calculations (RI-BP86/def2-TZVPP: 2.560 Å) are known and described.[40] The reoptimized structure afforded an Si^{I}–Si^{I} bond distance (2.482 Å) that is in very good agreement with that obtained from X-ray diffraction.[40] The orbital description is analogous to that in the original publication and the QTAIM contour plot is shown in Figure S90. E–E comparison data is furthermore collected in Table S7.

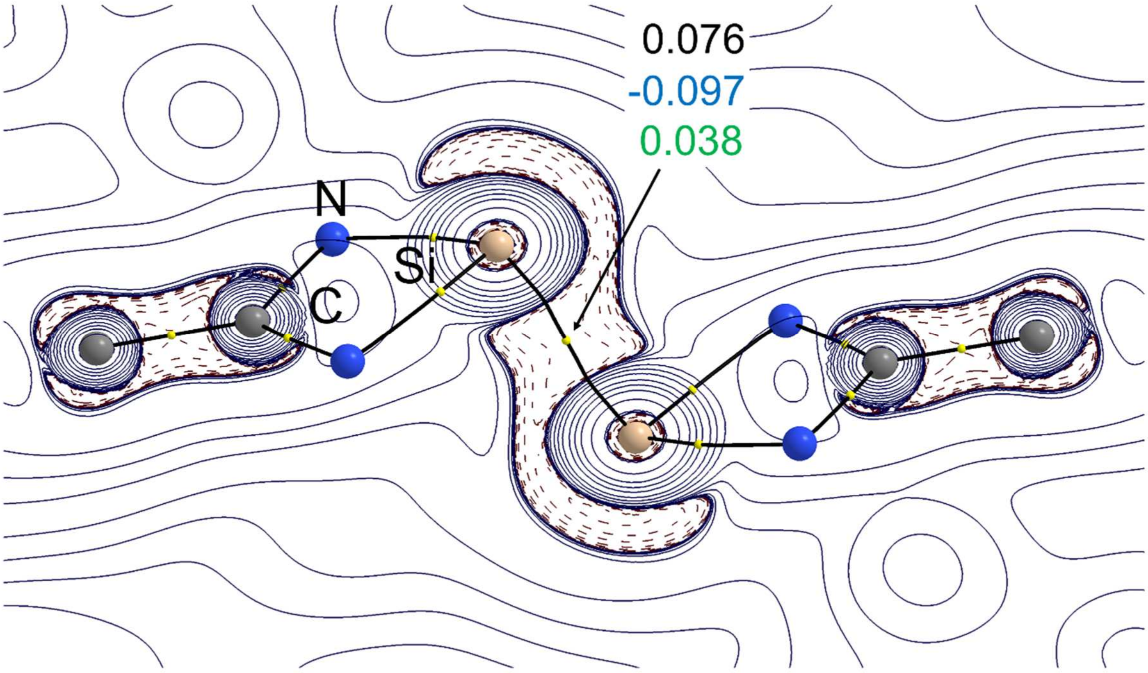

Figure S99. QTAIM contour plots showing the Laplacian of the electron density (solid lines: positive, dashed lines: negative) for **8** (through the C-Si-Si-plane but angling the N$_2$Si unit to show bond paths); bond critical points (bcps): yellow, bond paths: black lines, values for the electron density ρ [e bohr^{-3}], (black), Laplacian, $\nabla^2\rho$ [e bohr^{-5}], (blue), and bond ellipticity ε, (green) are given for the bcp between both Si atoms.

4.3 Germanium compounds

4.3.1 TipGe 5 (monomer)

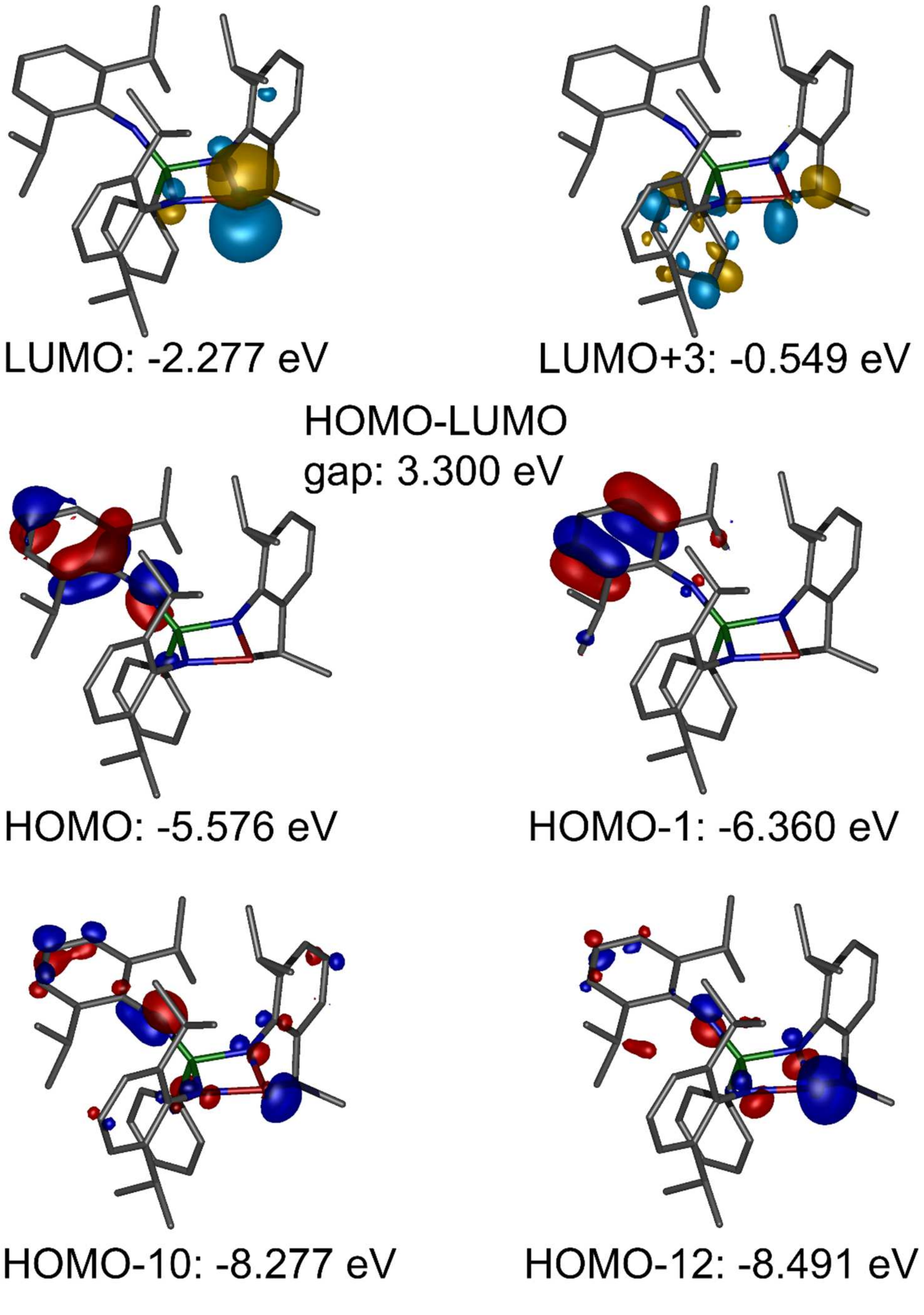

Figure S100. Selected molecular orbitals at the M06-D3/def2-TZVP//M06-L-D3/def2-TZVP level (isovalue 0.06) of TipGe **5**.

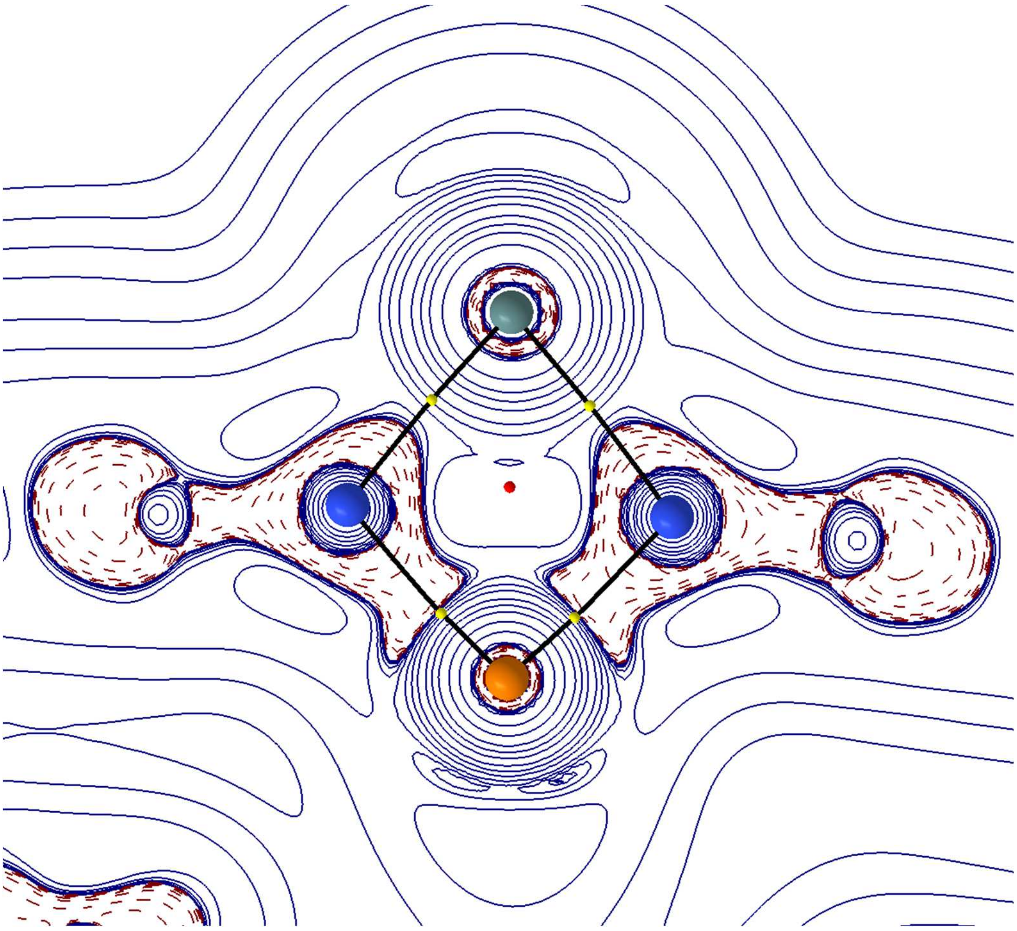

Figure S101. QTAIM contour plots showing the Laplacian of the electron density (solid lines: positive, dashed lines: negative) for TipGe **5** through the PNGe plane. Ge at the top, N blue, P orange.

4.3.2 (TipGe)$_2$ 5$_2$ (dimer)

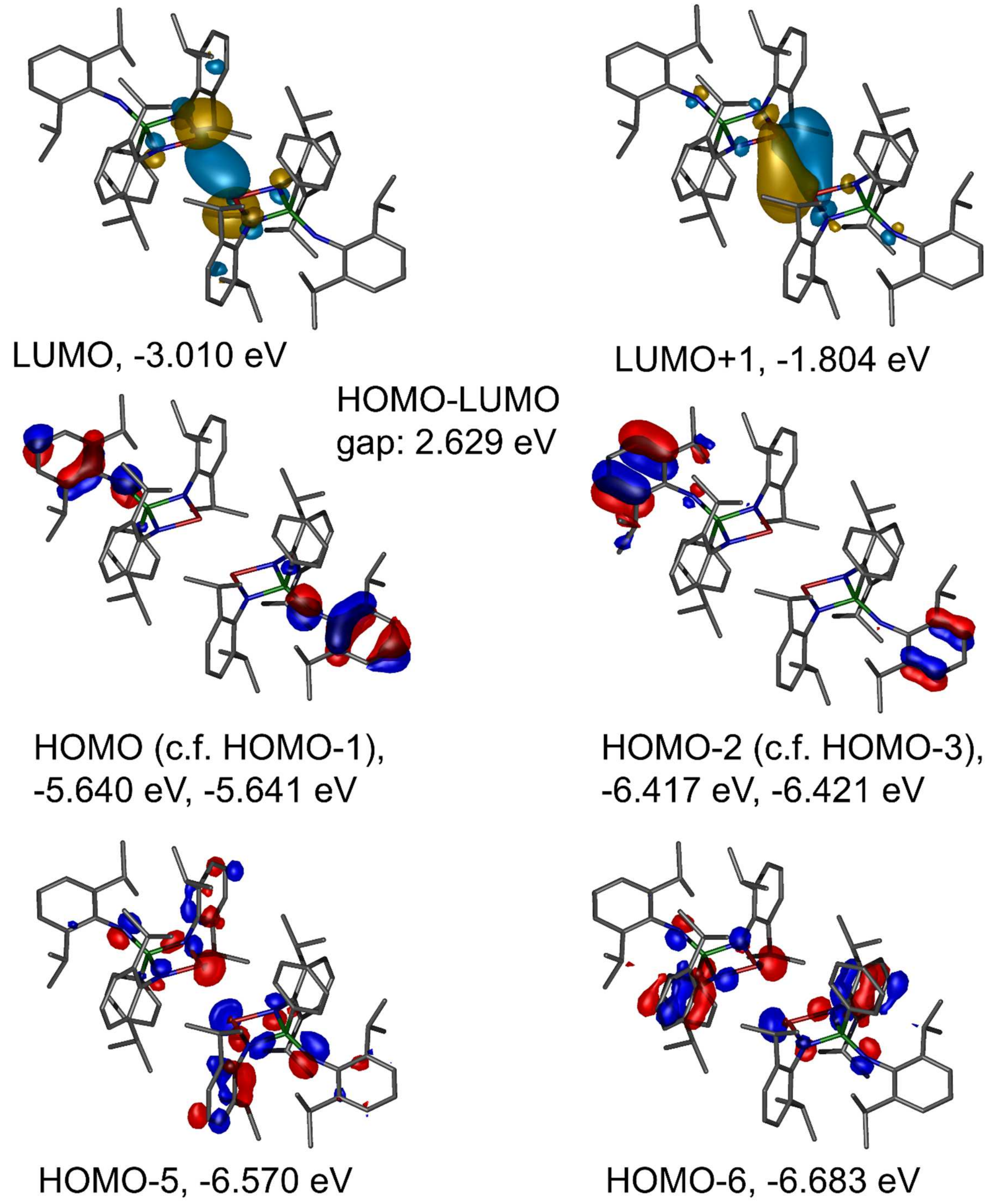

Figure S102. Selected molecular orbitals at the M06-D3/def2-TZVP//M06-L-D3/def2-TZVP level (isovalue 0.04) of (TipGe)$_2$ **5**$_2$.

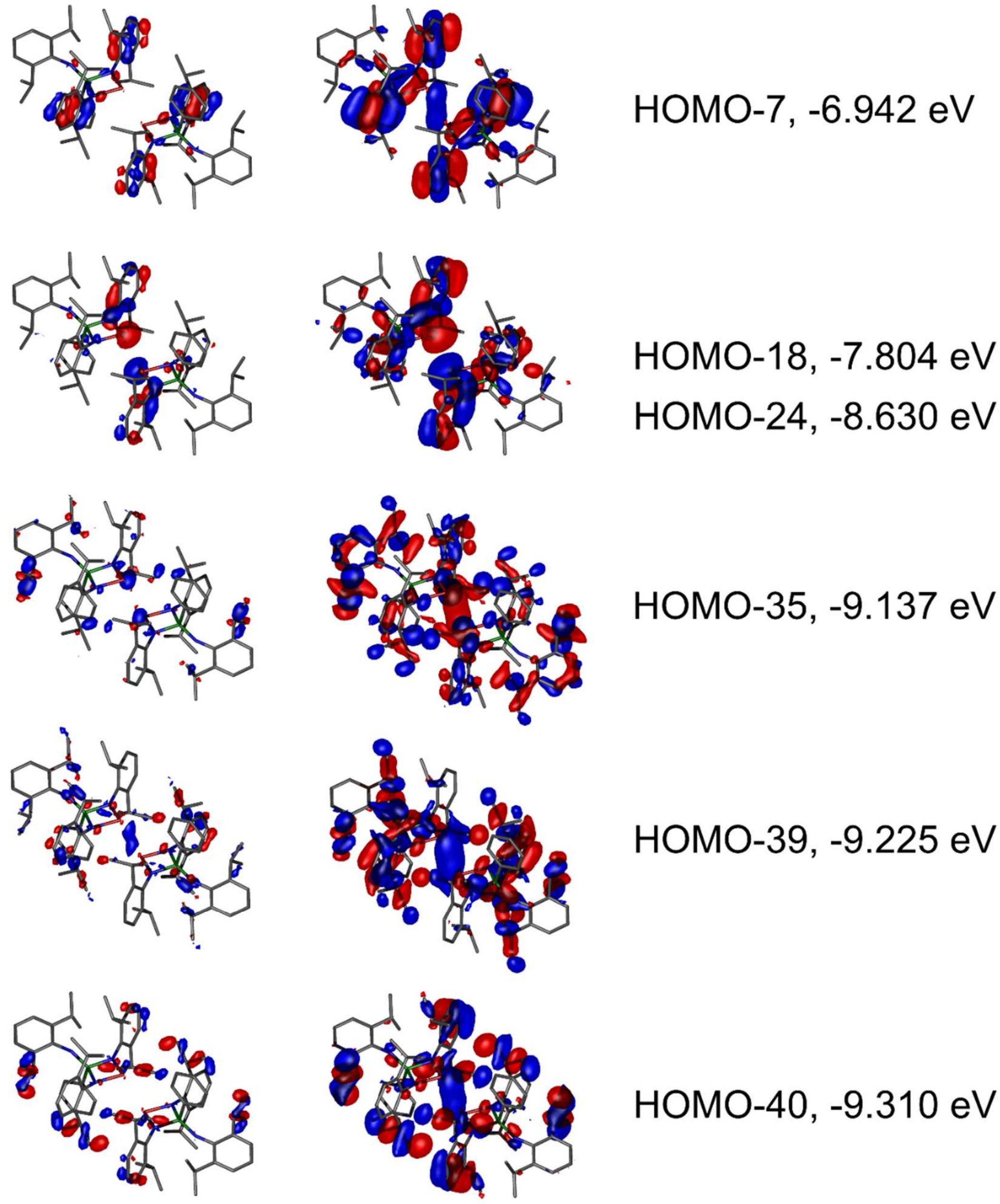

Figure S103. Selected (low-lying) molecular orbitals at the M06-D3/def2-TZVP//M06-L-D3/def2-TZVP level (isovalue 0.04 left, isovalue 0.02 right) of $(TipGe)_2$ $\mathbf{5}_2$.

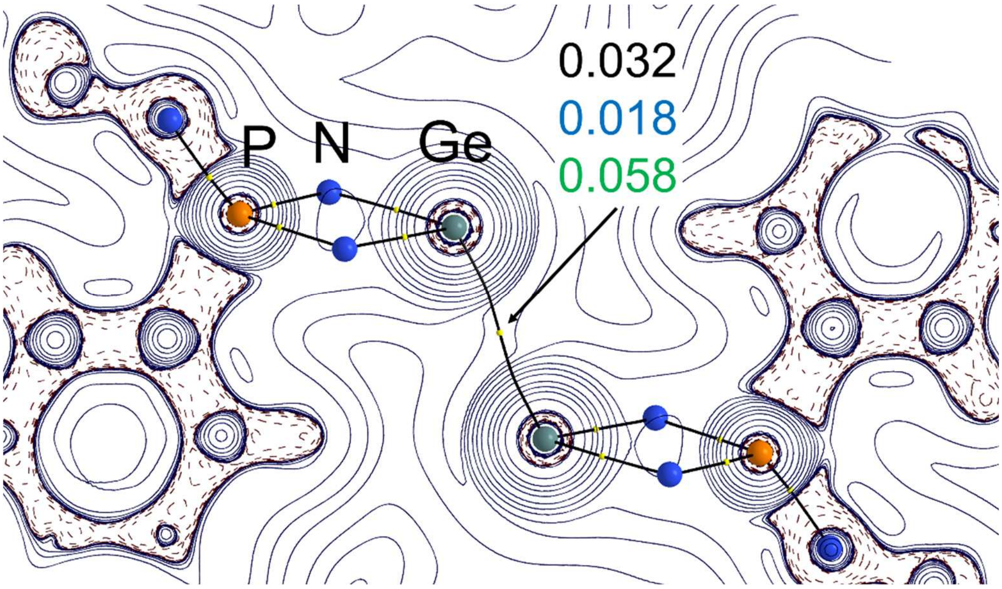

Figure S104. QTAIM contour plots showing the Laplacian of the electron density (solid lines: positive, dashed lines: negative) for **5**$_2$ (through the P-GeGe-plane but angling the N_2Si unit to show bond paths); bond critical points (bcps): yellow, bond paths: black lines, values for the electron density ρ [e bohr^{-3}], (black), Laplacian, $\nabla^2\rho$ [e bohr^{-5}], (blue), and bond ellipticity ε, (green) are given for the bcp between the two Ge atoms.

4.3.3 (Tip’Ge)$_2$ 5’$_2$ (dimer)

This dimer optimised in a Ge-Ge bonded geometry without contributions from dispersion (B3LYP/def2-TZVP level), but with a very large Ge···Ge distance (3.614 Å, *trans*-bending angle = 58.5°). This is ca. 1 Å (0.953 Å) longer than the same bond for the respective Si model (2.661 Å, *trans*-bending angle = 57.2°) at the same level of theory. For comparison of E–E bonding data, see Table S7.

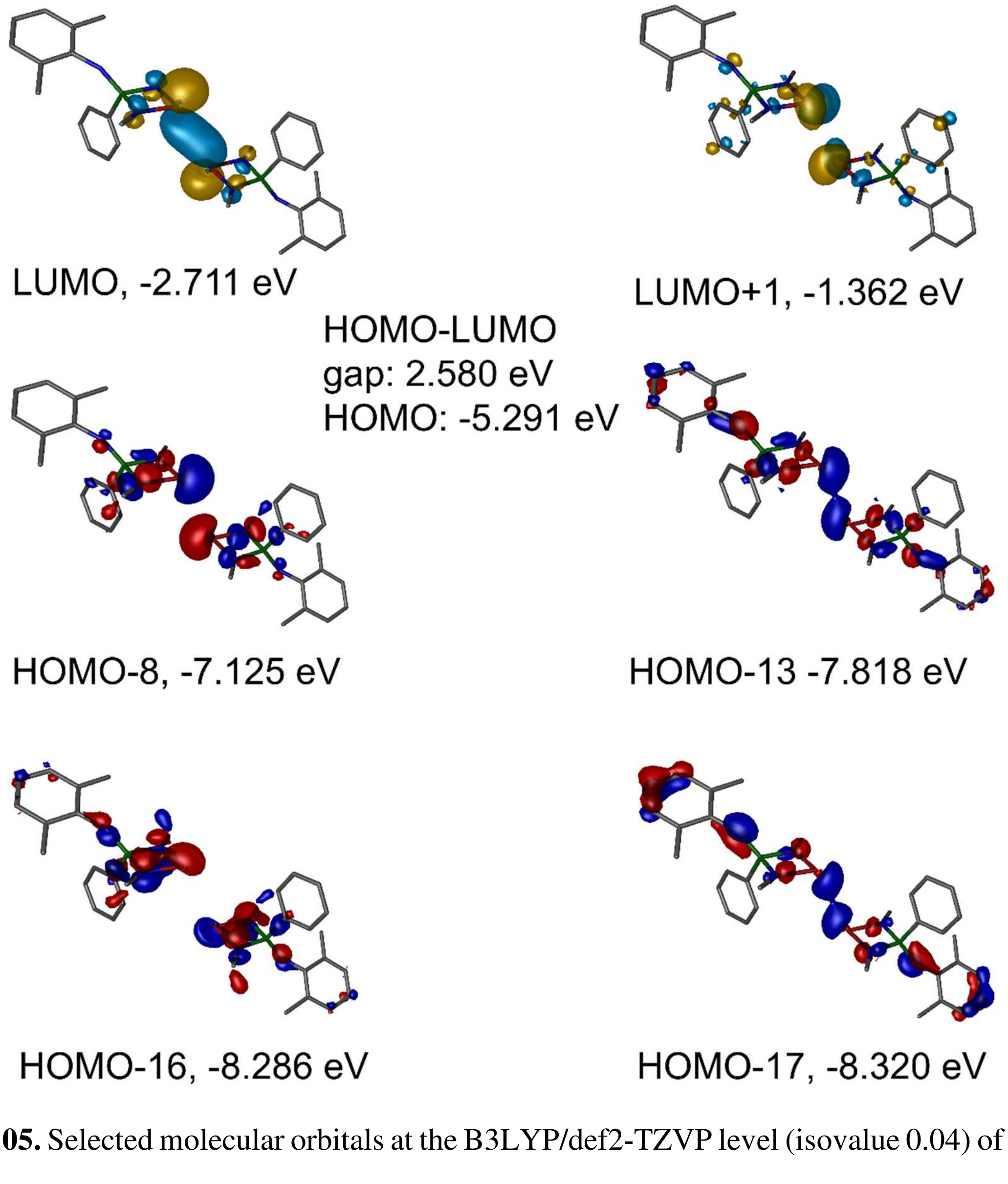

Figure S105. Selected molecular orbitals at the B3LYP/def2-TZVP level (isovalue 0.04) of (TipGe’)$_2$ **5’$_2$**.

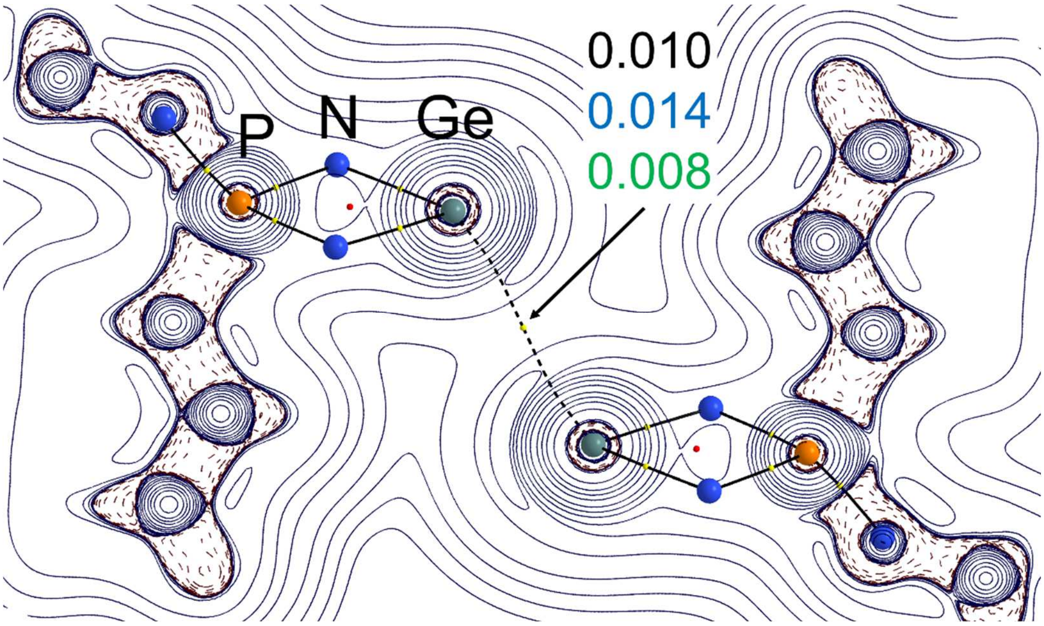

Figure S106. QTAIM contour plots showing the Laplacian of the electron density (solid lines: positive, dashed lines: negative) for **5'**$_2$ (through the P-Ge-plane but angling the N_2Ge unit to show bond paths); bond critical points (bcps): yellow, bond paths: black lines, values for the electron density ρ [e bohr^{-3}], (black), Laplacian, $\nabla^2\rho$ [e bohr^{-5}], (blue), and bond ellipticity ε, (green) are given for the bcp between the two Ge atoms. When compared to the Si–Si-bonded examples (Figure S88), there is no area of negative Laplacian at the midpoint of the E–E bond and the electron density at the bcp is also much lower.

4.4 Tin and lead compounds

4.4.1 TipSn 6

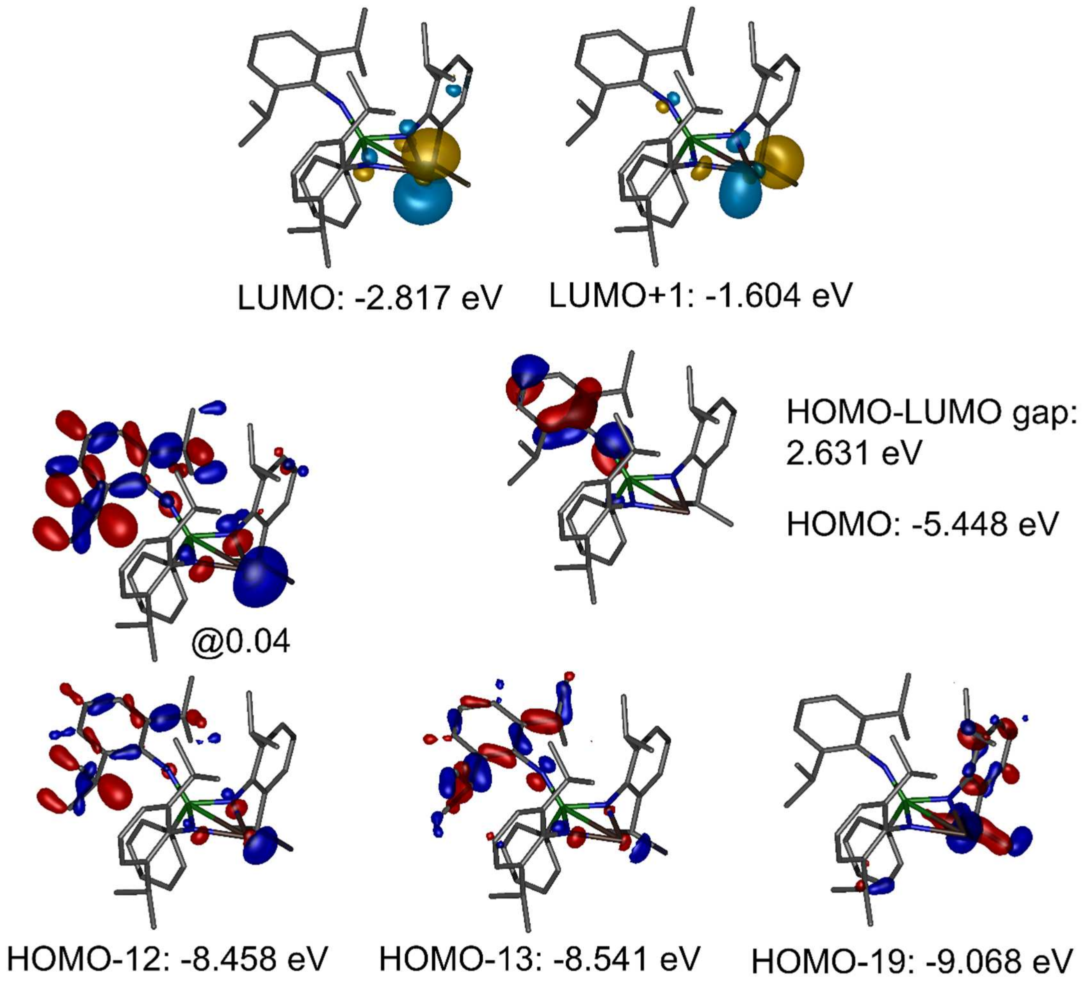

Figure S107. Selected molecular orbitals at the M06-D3/def2-TZVP//M06-L-D3/def2-TZVP level (isovalue 0.06) of TipSn **6**. The Sn lone pair orbital appears to be mixed in with several other contributions in low-lying orbitals and an image of the HOMO-12 is also provided at an isovalue of 0.04.

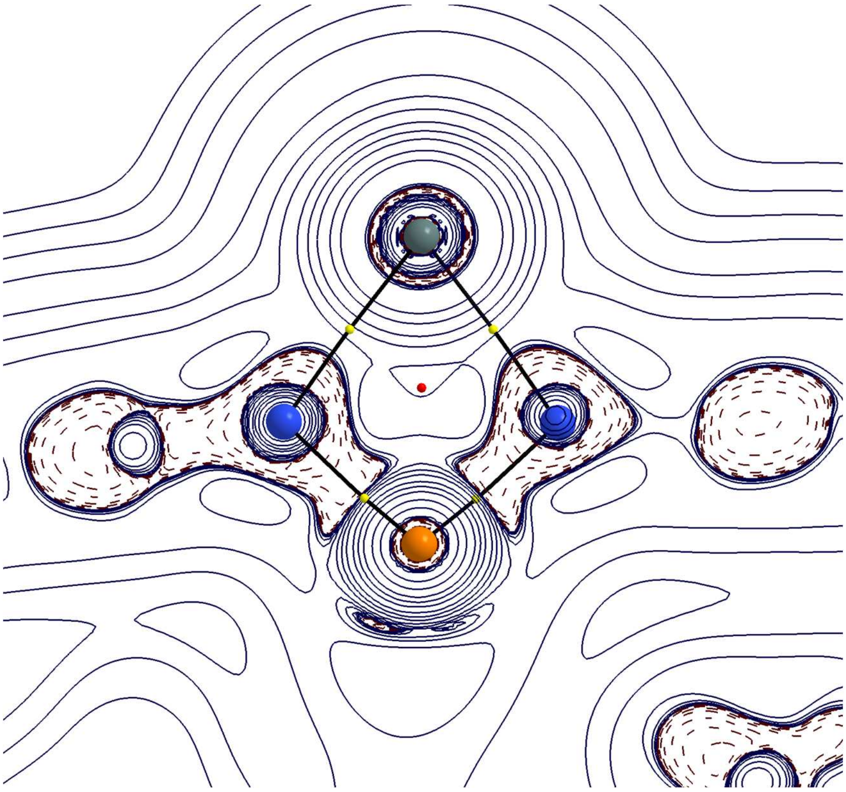

Figure S108. QTAIM contour plots showing the Laplacian of the electron density (solid lines: positive, dashed lines: negative) for TipSn **6** through the PNSn plane. Sn at the top, N blue, P orange.

4.4.2 TipPb 7

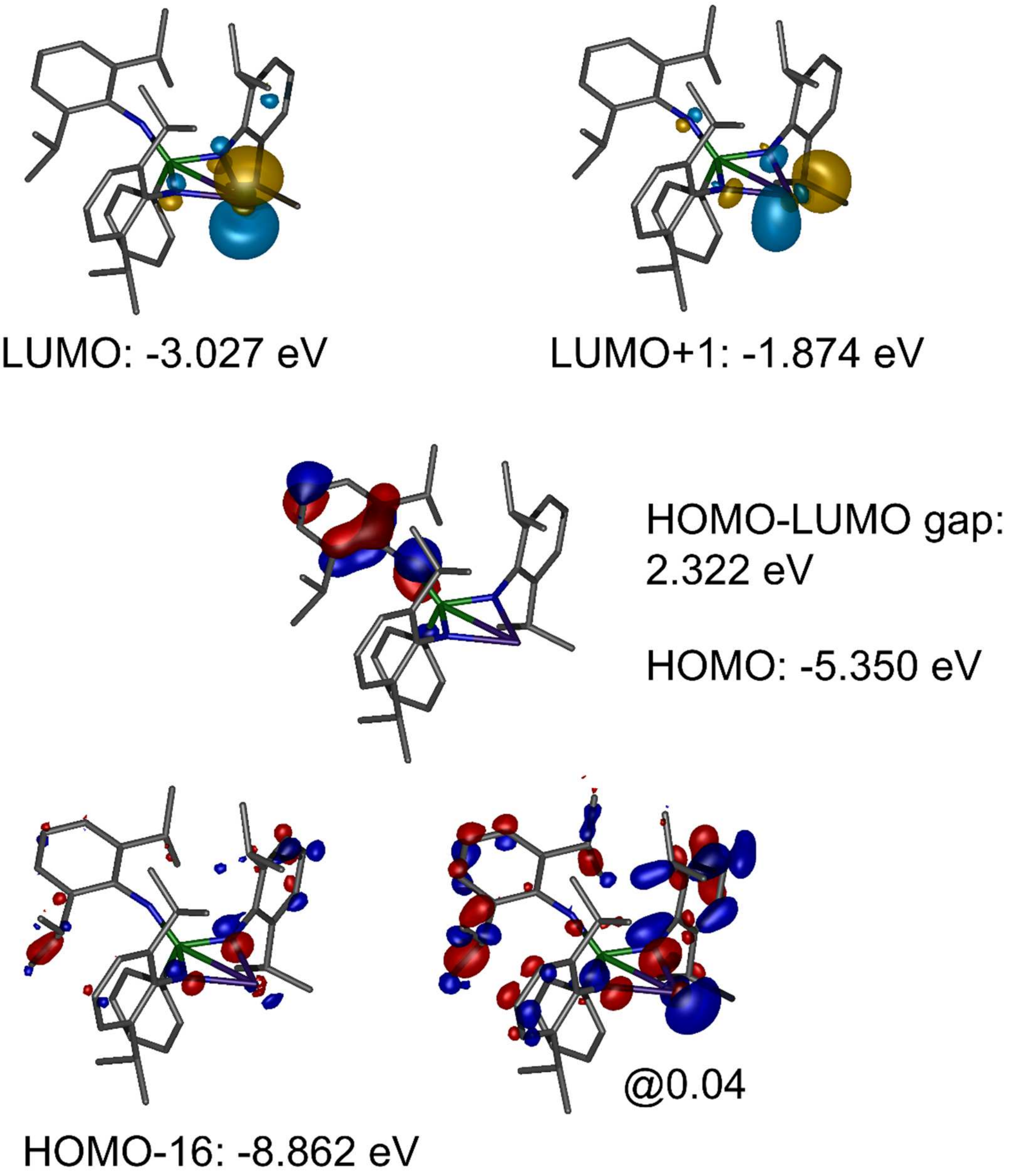

Figure S109. Selected molecular orbitals at the M06-D3/def2-TZVP//M06-L-D3/def2-TZVP level (isovalue 0.06) of TipPb **7**. The Pb lone pair orbital appears to be mixed in with other contributions in low-lying orbitals and an image of the HOMO-16 is also provided at an isovalue of 0.04.

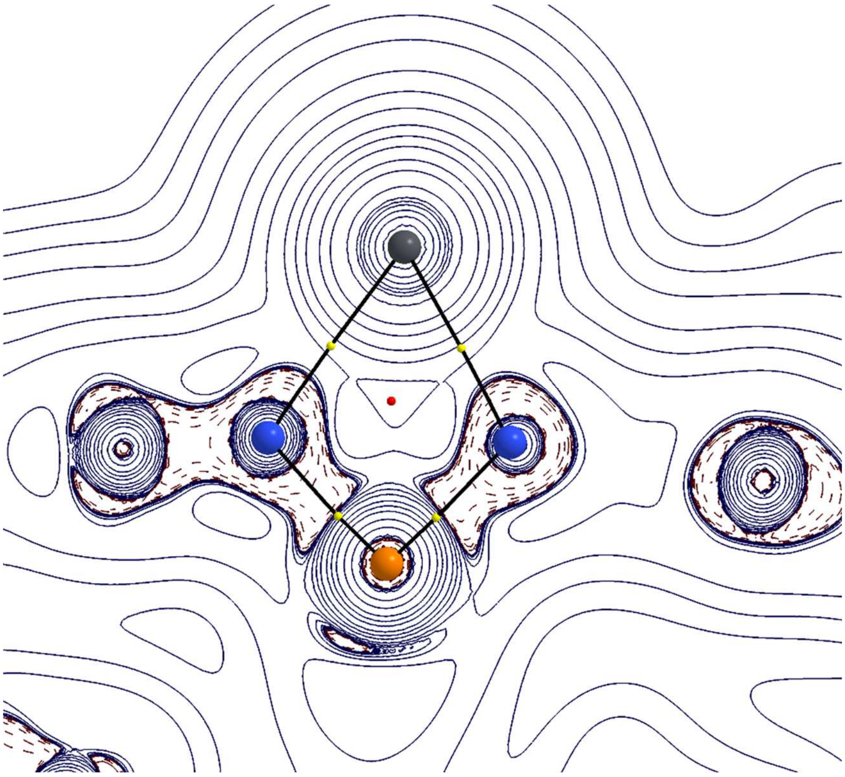

Figure S110. QTAIM contour plots showing the Laplacian of the electron density (solid lines: positive, dashed lines: negative) for TipPb **7** through the PNPb plane. Pb at the top, N blue, P orange.

4.5 Comparison of molecules

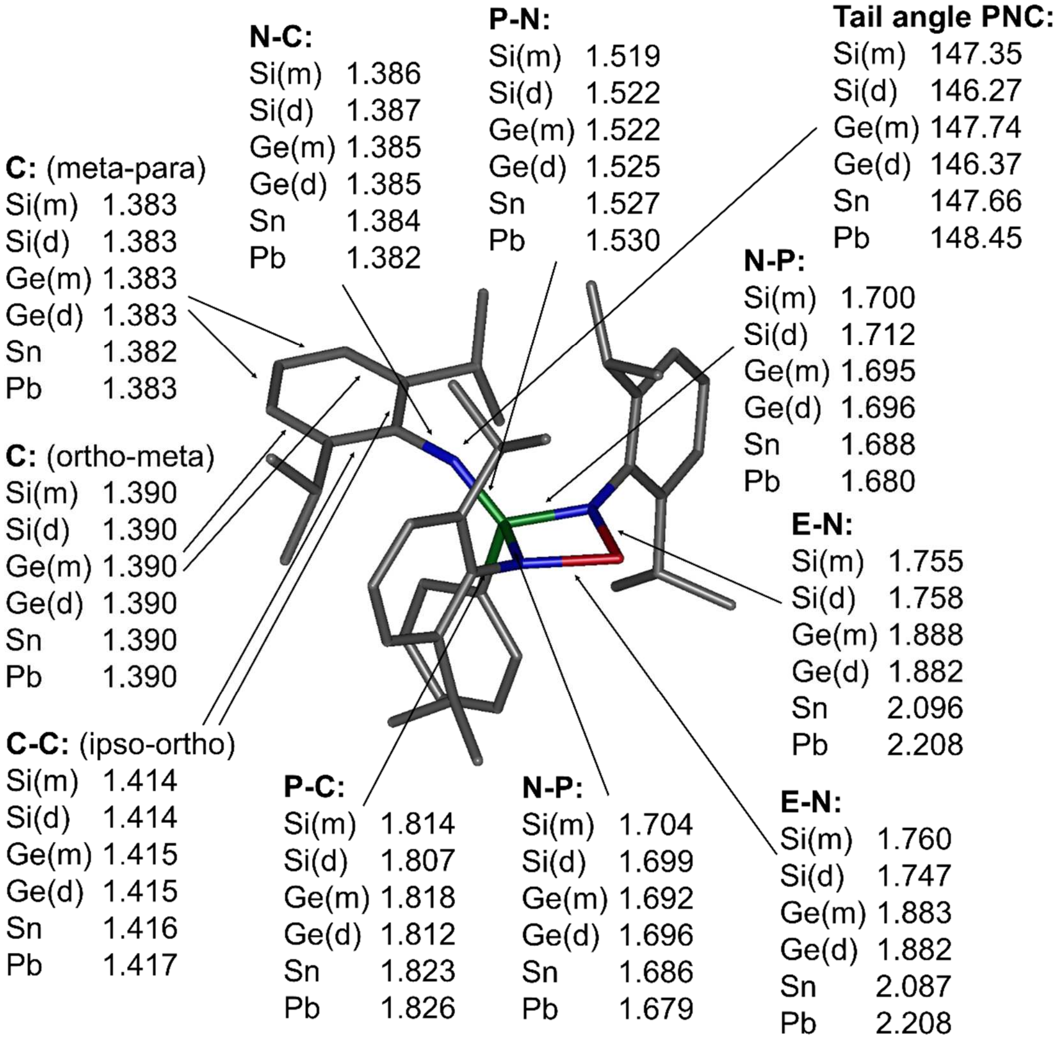

Figure S111. Comparison of bond lengths (Å) and tail angle (°) of DFT-optimised (M06-D3/def2-TZVP//M06-L-D3/def2-TZVP) monomeric compounds TipE, E = Si **4**, Ge **5**, Sn **6**, Pb **4**, and dimeric compounds $(TipE)_2$, E = Si $\mathbf{4}_2$, Ge $\mathbf{5}_2$. For Si and Ge, (m) refers to the monomer and (d) refers to the dimer. Values for C–C bonds are averages. For dimers (d), only values for one half are given, but the values for the second half are essentially identical.

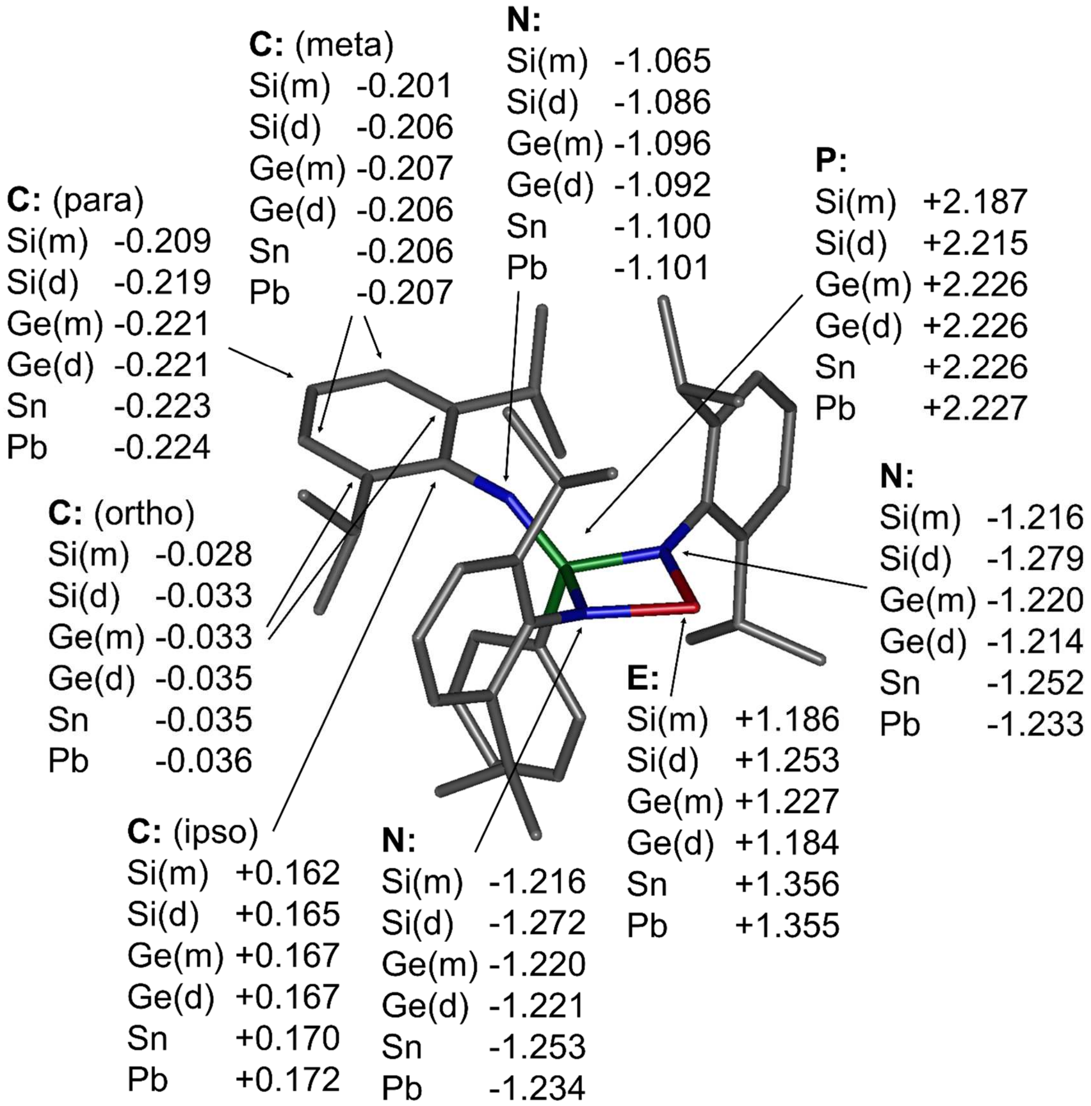

Figure S112. Comparison of NPA atomic charges of DFT-optimised (M06-D3/def2-TZVP//M06-L-D3/def2-TZVP) monomeric compounds TipE, E = Si **4**, Ge **5**, Sn **6**, Pb **4**, and dimeric compounds $(TipE)_2$, E = Si $\mathbf{4}_2$, Ge $\mathbf{5}_2$. For Si and Ge, (m) refers to the monomer and (d) refers to the dimer. Values for carbon atoms are averages for ortho and meta carbon atoms. For dimers (d), only values for one half are given, but the charges in the second half are essentially identical.

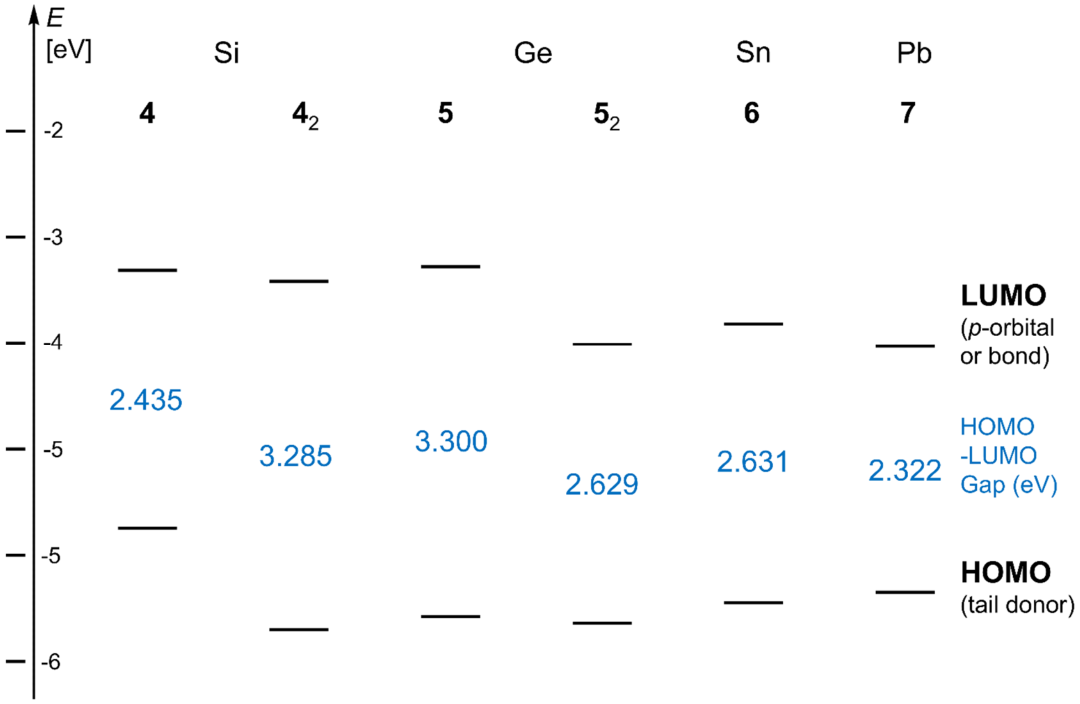

Figure S113. HOMO and LUMO orbital energies for DFT-calculated (M06-D3/def2-TZVP//M06-L-D3/def2-TZVP) TipE species **4-7** and for the dimers $(TipSi)_2$ $\mathbf{4}_2$ and $(TipGe)_2$ $\mathbf{5}_2$.

Table S5. Selected energies (in kcal mol^{-1}) for the dimerization reaction (gas phase) of two monomers to one dimer at the respective DFT level with BSSE correction; the latter was found to be small.

Species (method)	ΔG [kcal mol^{-1}]	ΔH [kcal mol^{-1}]	ΔE_0 [kcal mol^{-1}]
2 **4** to $\mathbf{4}_2$ (M06D3)	-17.2	-34.9	-34.7
2 **4’** to $\mathbf{4'}_2$ (B3LYP)	+9.8	-1.1	-1.9
2 **5** to $\mathbf{5}_2$ (M06D3)	-12.1	-28.9	-29.0
2 **5’** to $\mathbf{5'}_2$ (B3LYP)	+7.1	+1.5	+0.4

ΔE_0 = ZPE-corrected energy

Table S6. Singlet triplet gaps ($\Delta E_{s\text{-}t}$) of **4**, $\mathbf{4}_2$, **5** and $\mathbf{5}_2$ (M06D3).

Species (change formula)	Vertical $\Delta E_{s\text{-}t}$ [kcal mol^{-1} (eV)]	Adiabatic $\Delta E_{s\text{-}t}$ [kcal mol^{-1} (eV)]
**4**, Si monomer (full)	70.7 (3.06)	58.4 (2.53)
$\mathbf{4}_2$, Si dimer (full)	36.4 (1.58)	-
**5**, Ge monomer (full)	66.9 (2.90)	45.9 (1.99)
$\mathbf{5}_2$, Ge dimer (full)	47.4 (2.06)	-

Table S7. Comparison of DFT-optimised E–E-bonded species.

compound	E–E [Å]	WBI	ϑ [°]	ρ [e bohr^{-3}]	$\nabla^2\rho$ [e bohr^{-5}]	ε
**4**$_2$ (M06D3)	2.479	1.03	56.3	0.063	-0.051	0.117
**4'**$_2$ (B3LYP)	2.661	0.752	57.2	0.049	-0.022	0.067
**8** (M06D3)	2.482	0.803	77.1	0.076	-0.097	0.038
**5**$_2$ (M06D3)	2.892	0.502	62.6	0.032	+0.018	0.058
**5'**$_2$ (B3LYP)	3.614	0.118	58.5	0.010	+0.014	0.008

Bond distance E–E and *trans*-bending angle (ϑ, as defined in Figure 1; mean, if two slightly different values were obtained) are taken from the optimised geometry; WBI from NBO analysis. Values for ρ, $\nabla^2\rho$ and ε are given for the respective (3, -1) bcp at the E–E bond as obtained from QTAIM analysis.

4.6 Atomic coordinates (XYZ) (in Å)

M06-D3/def2-TZVP//M06-L-D3/def2-TZVP

TipSi **4**
103
-2429.019694
P -0.01891000 -0.38115100 0.02609600
Si -0.19341400 -1.51459400 2.33038400
N 1.04569200 -0.94961100 1.22299300
N 0.10277300 1.06362500 -0.42746400
N -1.25526800 -0.80765500 1.11800900
C 2.41101600 -0.58411600 1.37248100
C 0.02145400 -1.60745900 -1.31058400
C 0.19444300 1.94938400 -1.48893900
C -2.65320100 -0.53014200 1.08016100
C 2.71775100 0.63413200 2.01171600
C 3.43130700 -1.41144900 0.87691300
C 1.45375900 2.33502500 -1.99870900
C -3.56522900 -1.47411500 0.58550000
C -0.98173800 2.51668500 -2.03416500
C 0.06685600 -1.17847900 -2.63765500
H 0.05865000 -0.11862000 -2.86050500
C 0.03675400 -2.97752300 -1.05004300
H 0.00956500 -3.32974200 -0.02345200
C -0.87474000 3.39161000 -3.10683400
H -1.77690900 3.81711000 -3.52906700
C 0.09144100 -3.89743600 -2.08114100
H 0.10239900 -4.95717500 -1.85941500
C -3.10325000 0.70959600 1.58522000
C -2.33089500 2.15710300 -1.46213800
H -2.16945500 1.97313800 -0.39807100
C 4.74601000 -0.96115100 0.95269600
H 5.53806300 -1.58694600 0.55728800
C 1.50170200 3.21417700 -3.07543300
H 2.46962900 3.49277300 -3.47656900
C 3.50406900 -2.88583400 -1.16550000
H 4.55803200 -2.66349300 -1.34222000
H 2.91595500 -2.18853400 -1.76124900
H 3.31146000 -3.89074700 -1.54276400
C 0.35472300 3.73667800 -3.64167600
H 0.41667400 4.41727200 -4.48084800
C -5.36819400 0.07897500 1.01399700
H -6.42258000 0.32140100 0.97730500
C 3.15970100 -2.78779400 0.31519000
H 2.09203000 -2.97629600 0.42100500
C -4.46281100 0.98967300 1.53283200
H -4.82106500 1.94039200 1.90564000
C -4.91815600 -1.14207700 0.55720000
H -5.62588300 -1.86302200 0.16465500
C -3.14739800 -2.83806100 0.09062000
H -2.07863800 -2.93934700 0.27168300
C 5.05768600 0.25617700 1.52698500
H 6.08572500 0.59298500 1.56853900
C 2.74173800 1.78404800 -1.43381400
H 2.50279300 1.30932100 -0.48170300
C -3.84424400 -3.95159200 0.86482500
H -4.92355500 -3.93592600 0.70883600
H -3.66619300 -3.86556000 1.93668300
H -3.48401900 -4.92846700 0.54090700
C 0.13564000 -3.45796000 -3.39754600
H 0.18047100 -4.17586500 -4.20640400
C 0.12410900 -2.09985700 -3.67305700
H 0.16034900 -1.75128600 -4.69691700
C 4.04916700 1.03093200 2.06965900
H 4.29549800 1.97267200 2.54678300
C 3.77572800 2.86752400 -1.15425400
H 4.65440700 2.43832500 -0.67177700
H 3.37816000 3.64251600 -0.49912200
H 4.11406400 3.35388600 -2.07037900
C -2.14465300 1.69935500 2.21666700
H -1.20149300 1.63708200 1.66625000
C -3.36933500 3.26112300 -1.58846900
H -3.69228100 3.40286700 -2.62097600
H -2.99296100 4.21857300 -1.22752600
H -4.25879000 3.00582600 -1.01027600
C 1.67383000 1.51212200 2.66802600

H 0.69019700 1.07225500 2.48833000
C -2.87394500 0.86188400 -2.05756000
H -3.83203800 0.59369400 -1.60803500
H -2.19290500 0.02661500 -1.89580600
H -3.01990300 0.96073300 -3.13554900
C -3.37955900 -2.98347200 -1.40933000
H -3.06685200 -3.96831900 -1.75788900
H -2.82257000 -2.23799500 -1.97756600
H -4.43593300 -2.86481100 -1.65652300
C 3.89245700 -3.85849300 1.11774000
H 3.63563700 -4.85422400 0.75515000
H 3.63893600 -3.80962100 2.17656900
H 4.97475700 -3.75135600 1.03432000
C -1.87599300 1.32993300 3.67337800
H -2.79689500 1.36788700 4.25791200
H -1.16492400 2.02368200 4.12384600
H -1.47010800 0.32155400 3.78740900
C -2.60948100 3.14690300 2.14147300
H -2.88053500 3.43982400 1.12837300
H -1.81364000 3.81097000 2.47720700
H -3.47072100 3.33402900 2.78469000
C 3.32453200 0.71212800 -2.34950400
H 3.60728300 1.13564200 -3.31549900
H 2.60365000 -0.08312000 -2.54275800
H 4.21334000 0.25788800 -1.90821700
C 1.63208200 2.91617300 2.07838100
H 0.85195900 3.50610500 2.56187900
H 1.41700600 2.88973800 1.01055200
H 2.57699200 3.44236700 2.22379600
C 1.89135500 1.55237300 4.17830500
H 2.86752900 1.97178600 4.42536700
H 1.84265800 0.55539100 4.61770600
H 1.13712900 2.17102500 4.66511700

(TipSi)$_2$ **4**$_2$
206
-4858.063721
P -3.38462200 -0.03054400 0.38053900
P 3.38462000 0.03054800 -0.38053900
Si -1.04670600 -0.10820700 -0.65473100
Si 1.04671000 0.10821300 0.65474600
N 2.22234000 -1.08548500 0.15844100
N -2.26753900 -1.23948700 -0.08917500
N -4.75124000 -0.00166200 -0.28898900
N 4.75124200 0.00167000 0.28898100
N -2.22234000 1.08548900 -0.15843300
N 2.26754000 1.23949100 0.08918200
C 2.46897300 -2.41198100 0.60523400
C 3.41399400 -0.06895500 -2.18427200
C 6.12414300 -0.06196000 0.10539900
C 2.48158100 2.64398100 0.25378100
C -2.22638100 0.15151900 2.91493000
H -1.27395400 0.13459100 2.39689000
C -2.28052400 3.49502100 0.26957300
C -3.27375500 -2.19409500 -2.64896700
H -3.59541000 -1.23709300 -2.23200800
C 2.91510500 -2.62024300 1.92551000
C -2.48157300 -2.64397700 -0.25377500
C 2.28051600 -3.49501900 -0.26955600
C 6.78391900 -1.29783100 -0.07252100
C 2.16780100 3.54562700 -0.77714700
C -6.88665600 -1.13007900 -0.15046800
C -3.41400300 0.06895800 2.18427200
C -4.63031700 0.09689100 2.86645200
H -5.56021200 0.03464600 2.31460500
C 6.88666800 1.13006200 0.15046300
C 4.63030700 -0.09688800 -2.86645400
H 5.56020300 -0.03464300 -2.31460800
C -3.00057200 -3.11894400 -1.48127500
C -2.63135000 4.76890600 -0.16910800
H -2.50847800 5.60883400 0.50415400
C -2.16779600 -3.54562300 0.77715400
C -3.23012300 -4.48141200 -1.62527500
H -3.63151500 -4.85218200 -2.55923300
C -6.12414300 0.06195100 -0.10541400
C -2.43323800 -4.90070700 0.58189800
H -2.21110600 -5.59692800 1.38052200
C 2.22637100 -0.15151500 -2.91492700
H 1.27394500 -0.13458500 -2.39688500

C	-4.65383200	0.20591700	4.24960300
H	-5.60369500	0.22829800	4.76792800
C	-2.25211400	0.25764300	4.29339900
H	-1.32161900	0.31860800	4.84551200
C	-2.96482200	-5.37090600	-0.59959200
H	-3.16416600	-6.42715100	-0.72793100
C	8.26412600	1.06346600	-0.00176600
H	8.84066000	1.97987000	0.02927900
C	-1.54079100	-3.13145800	2.08875500
H	-1.18401300	-2.10140900	1.97954500
C	2.25210200	-0.25763900	-4.29339600
H	1.32160600	-0.31860400	-4.84550800
C	3.00058700	3.11894700	1.48127800
C	-6.21222100	3.29065000	1.48862500
H	-5.61433300	4.20183700	1.54272100
H	-5.90234700	2.63750000	2.30530600
H	-7.25250800	3.56686400	1.66959200
C	6.19129000	2.45717800	0.31417100
H	5.31888100	2.26996800	0.94336100
C	2.63133900	-4.76890400	0.16912900
H	2.50846300	-5.60883400	-0.50412900
C	-3.11213500	4.98277600	-1.44727300
H	-3.38151200	5.98092200	-1.76837600
C	8.16791400	-1.30559900	-0.21983200
H	8.66754300	-2.25768200	-0.36278300
C	-1.68735700	3.32770500	1.64989000
H	-1.30241200	2.30524400	1.71655300
C	-6.19126700	-2.45719000	-0.31417000
H	-5.31885600	-2.26997500	-0.94335500
C	-3.46840300	0.28630900	4.96362500
H	-3.49056200	0.37152000	6.04257500
C	2.73731600	-3.51005300	-2.73904500
H	3.14545500	-4.52243100	-2.71736600
H	3.56970300	-2.81724400	-2.62084500
H	2.30474200	-3.34804500	-3.72702900
C	-2.46897700	2.41198600	-0.60522100
C	8.91438700	-0.14445000	-0.18950600
H	9.98977800	-0.17784000	-0.30610600
C	2.96484100	5.37090900	0.59959300
H	3.16419000	6.42715400	0.72793000
C	-2.55207200	-3.17392300	3.23042600
H	-2.92730400	-4.18994800	3.36844100
H	-3.41032100	-2.52872900	3.05052700
H	-2.08943000	-2.86061100	4.16734600
C	1.68735300	-3.32770400	-1.64987500
H	1.30240200	-2.30524500	-1.71653600
C	-3.22863600	3.91669200	-2.31906500
H	-3.57844400	4.08833900	-3.33073300
C	-6.78393200	1.29781800	0.07248500
C	3.23014400	4.48141500	1.62527600
H	3.63154000	4.85218500	2.55923100
C	2.43324900	4.90071000	-0.58189400
H	2.21111700	5.59693100	-1.38051700
C	1.54078500	3.13146400	-2.08874300
H	1.18400400	2.10141500	-1.97953000
C	3.11212400	-4.98277000	1.44729400
H	3.38149800	-5.98091600	1.76840100
C	6.04881100	-2.61699700	-0.12955300
H	4.98615100	-2.41798500	0.01831600
C	0.34022400	4.01032100	-2.42345500
H	0.64092100	5.03795100	-2.62868100
H	-0.38273700	4.03646200	-1.60896600
H	-0.16916600	3.64825000	-3.31450800
C	-6.04883500	2.61699100	0.12949300
H	-4.98617800	2.41798600	-0.01840100
C	-5.67047400	-2.96703500	1.02600000
H	-6.49540000	-3.14498600	1.71935400
H	-5.00107700	-2.24504400	1.49459100
H	-5.11752300	-3.90053900	0.90823200
C	-0.51528100	4.27767000	1.86879800
H	-0.83456700	5.32063500	1.86366300
H	0.24817700	4.16431800	1.09872200
H	-0.04686000	4.09154000	2.83493100
C	3.46838900	-0.28630700	-4.96362400
H	3.49054700	-0.37151800	-6.04257500
C	4.65382000	-0.20591400	-4.24960500
H	5.60368100	-0.22829700	-4.76793100
C	-8.26411500	-1.06349600	0.00176200
H	-8.84063900	-1.97990600	-0.02927400

C	-2.91510800	2.62025100	-1.92549800
C	-2.00668900	-1.97582200	-3.47437500
H	-1.16589100	-1.60776100	-2.87877800
H	-2.18430900	-1.25751400	-4.27575400
H	-1.68658700	-2.91294200	-3.93379500
C	-7.03379900	-3.52656400	-0.99036000
H	-6.41916100	-4.39879500	-1.21851100
H	-7.47537800	-3.17029300	-1.92150700
H	-7.84835900	-3.87072100	-0.35119700
C	3.22862900	-3.91668400	2.31908200
H	3.57843700	-4.08832800	3.33075100
C	-2.73731100	3.51006600	2.73906600
H	-2.30473000	3.34806000	3.72704800
H	-3.56970300	2.81726100	2.62087800
H	-3.14544500	4.52244500	2.71738700
C	6.50084500	-3.56172800	0.97983700
H	5.95065800	-4.50186500	0.93547700
H	6.34314500	-3.12921000	1.96730900
H	7.56310300	-3.79503200	0.88888900
C	3.27377200	2.19410000	2.64897200
H	3.59542400	1.23709600	2.23201300
C	7.03383200	3.52654600	0.99035600
H	7.84839200	3.87069700	0.35118800
H	7.47541500	3.17027400	1.92150000
H	6.41920200	4.39878200	1.21851000
C	3.03078900	-1.50909000	2.94673500
H	2.82242200	-0.56203900	2.44617300
C	5.67049000	2.96702600	-1.02599600
H	5.11754500	3.90053200	-0.90822500
H	5.00108600	2.24503800	-1.49458200
H	6.49541300	3.14497300	-1.71935600
C	2.55206000	3.17392400	-3.23042000
H	2.08941100	2.86061100	-4.16733700
H	3.41030700	2.52872800	-3.05052500
H	2.92729300	4.18994800	-3.36843900
C	-4.38827500	-2.66931300	-3.57221300
H	-4.09784600	-3.55284100	-4.14240900
H	-4.62701400	-1.89191000	-4.29713900
H	-5.30122300	-2.90781700	-3.02963600
C	-8.91438800	0.14441500	0.18949200
H	-9.98977800	0.17779500	0.30609600
C	-8.16792700	1.30557300	0.21980000
H	-8.66756600	2.25765200	0.36274000
C	0.51528400	-4.27767400	-1.86879400
H	0.04686600	-4.09154100	-2.83492700
H	-0.24817800	-4.16433100	-1.09872000
H	0.83457500	-5.32063700	-1.86366500
C	-0.34023000	-4.01031300	2.42347400
H	0.16915400	-3.64824100	3.31453000
H	0.38273600	-4.03645100	1.60898800
H	-0.64092600	-5.03794400	2.62869700
C	2.00670800	1.97583100	3.47438300
H	1.68661000	2.91295300	3.93380300
H	2.18433000	1.25752500	4.27576400
H	1.16590700	1.60777100	2.87879000
C	4.38829600	2.66931700	3.57221400
H	5.30124300	2.90782000	3.02963400
H	4.62703600	1.89191200	4.29713900
H	4.09786900	3.55284400	4.14241200
C	6.21222400	-3.29065500	-1.48868200
H	7.25251200	-3.56687800	-1.66962600
H	5.90237400	-2.63750200	-2.30537100
H	5.61433000	-4.20183700	-1.54279200
C	-6.50090200	3.56171400	-0.97989000
H	-7.56315800	3.79501800	-0.88891600
H	-6.34322800	3.12919100	-1.96736400
H	-5.95071600	4.50185300	-0.93555000
C	4.43659200	-1.38749100	3.51890200
H	4.48312800	-0.57107800	4.24072300
H	5.16165100	-1.18096000	2.73197200
H	4.74353100	-2.29740100	4.03699900
C	1.98891800	-1.67279300	4.05085000
H	2.06646400	-2.64886500	4.53253800
H	0.97309900	-1.57579400	3.65856200
H	2.11900800	-0.91570500	4.82468200
C	-3.03079100	1.50910200	-2.94672600
H	-2.82242300	0.56204800	-2.44616800
C	-4.43659500	1.38750400	-3.51889300
H	-5.16165300	1.18096700	-2.73196300

H -4.48313000 0.57109600 -4.24071900
H -4.74353600 2.29741700 -4.03698300
C -1.98892100 1.67281100 -4.05084100
H -2.06647100 2.64888400 -4.53252600
H -2.11900900 0.91572400 -4.82467500
H -0.97310200 1.57581500 -3.65855300

Adiabatic gap

TipSi **4** (triplet)
103
P -0.01189700 0.49586400 -0.03614100
Si -0.08885400 1.59344300 -2.40286000
N 1.11555600 0.93070500 -1.14811900
N 0.05914700 -0.86418600 0.72698600
N -1.20510300 0.77144800 -1.13187500
C 2.46107400 0.52861400 -1.27927300
C 0.01247200 1.71279400 1.31557500
C 0.10248500 -1.93010800 1.50985900
C -2.59432600 0.51251300 -1.11244800
C 2.75339300 -0.69436800 -1.92015600
C 3.49912600 1.34704700 -0.79890800
C 1.37172400 -2.48247800 1.91885500
C -3.50980900 1.49268300 -0.69036300
C -1.11775200 -2.52926000 1.99862400
C 0.01509800 1.36260200 2.66454600
H -0.02026900 0.31947100 2.95329200
C 0.06783600 3.06616800 0.97243600
H 0.07554000 3.34867200 -0.07706000
C -1.02789800 -3.57225100 2.89568200
H -1.93546900 -4.02193100 3.27644200
C 0.11246200 4.03966900 1.95467900
H 0.15216200 5.08391400 1.67095100
C -3.05821200 -0.74189600 -1.57704900
C -2.45026400 -2.01613200 1.52560900
H -2.32341800 -1.77778600 0.46884600
C 4.80732200 0.87668100 -0.87221700
H 5.61074900 1.49332700 -0.48528000
C 1.38028100 -3.53060000 2.81592000
H 2.32897700 -3.93907800 3.14002500
C 3.49896500 2.81412500 1.24903300
H 4.53367400 2.55652100 1.48507300
H 2.85323100 2.13761200 1.80859000
H 3.31781800 3.82400800 1.61898800
C 0.20167100 -4.07171400 3.31280500
H 0.23883500 -4.89460300 4.01544900
C -5.32679300 -0.04883200 -1.09970400
H -6.38643700 -0.27087700 -1.08619500
C 3.23811800 2.73048100 -0.25012300
H 2.18377500 2.94760000 -0.42229000
C -4.42311700 -0.99761500 -1.55036000
H -4.78859100 -1.95805700 -1.89089500
C -4.86787200 1.18495900 -0.68481400
H -5.57402600 1.93587500 -0.34921400
C -3.07659300 2.87219700 -0.25646600
H -2.00349500 2.94597000 -0.42776500
C 5.09940500 -0.34846400 -1.44193900
H 6.12302900 -0.69886600 -1.48711400
C 2.65699400 -1.87613300 1.42742600
H 2.44965100 -1.41886700 0.45988900
C -3.73623200 3.95511800 -1.10288500
H -4.81985600 3.96516100 -0.97604600
H -3.52606400 3.81158700 -2.16233900
H -3.36563800 4.94053700 -0.81940100
C 0.11174100 3.67924700 3.29498100
H 0.15041700 4.44157200 4.06252800
C 0.06546700 2.33977500 3.64749100
H 0.06939900 2.05025000 4.69037600
C 4.07774200 -1.11174200 -1.98056300
H 4.31062900 -2.05704400 -2.45830400
C 3.77242600 -2.89130500 1.22995900
H 4.62868800 -2.41142200 0.75807200
H 3.45910800 -3.71778600 0.59213000
H 4.11952400 -3.31071900 2.17542500
C -2.10224200 -1.78520000 -2.11750800
H -1.16567600 -1.69241300 -1.55603700
C -3.57550300 -3.03191800 1.64798700
H -3.87561300 -3.18132000 2.68660700
H -3.30282700 -4.00501600 1.23949400

H -4.45269200 -2.67425700 1.10899400
C 1.68500900 -1.55139500 -2.56414800
H 0.71794600 -1.07772600 -2.38564500
C -2.85858400 -0.71549100 2.21548700
H -3.82049900 -0.37438300 1.83069800
H -2.13901600 0.08158300 2.04321600
H -2.95447700 -0.85601200 3.29391400
C -3.34059800 3.09754200 1.22817800
H -3.01876600 4.09362800 1.53395800
H -2.80782900 2.37404000 1.84652400
H -4.40427400 3.00919700 1.45812400
C 4.04661600 3.78351500 -1.00059000
H 3.78527400 4.78391500 -0.65474800
H 3.86016800 3.74018400 -2.07313900
H 5.11907700 3.65659600 -0.84556100
C -1.79275600 -1.51997800 -3.58916400
H -2.70938300 -1.55295600 -4.18102600
H -1.11712800 -2.28198900 -3.98080000
H -1.32182700 -0.54670200 -3.74339100
C -2.58990600 -3.21817300 -1.95263400
H -2.90278500 -3.43962300 -0.93331500
H -1.79393300 -3.91441400 -2.21574800
H -3.43122600 -3.43871900 -2.61125500
C 3.10601800 -0.75619500 2.36449000
H 3.34217000 -1.14779000 3.35588200
H 2.33129700 0.00150100 2.48167900
H 3.99602200 -0.26406600 1.97127000
C 1.61970200 -2.95844100 -1.98149200
H 0.84403400 -3.53977300 -2.48233400
H 1.38417700 -2.94631000 -0.91618000
H 2.56084300 -3.49680800 -2.10691200
C 1.89015300 -1.59048200 -4.07569700
H 2.86543600 -2.00622500 -4.33452700
H 1.82546000 -0.58824500 -4.50045800
H 1.13026200 -2.20621500 -4.55696800

B3LYP/def2-TZVP

Tip'Si **4'**
41
-1416.967363

P 0.80210100 -0.66628400 0.09616500
N 1.78448100 -1.31557100 1.31212500
N -0.69762100 -0.90242600 0.18418800
N 1.73140300 -1.62358500 -0.95718000
C 1.29330900 1.06835700 -0.18805100
C -1.99108900 -0.42481400 0.13603500
C -2.55784700 0.26030600 1.23537000
C -2.78657800 -0.67978500 -1.00470300
C 0.33433300 1.98904100 -0.61914500
H -0.69060200 1.67354200 -0.75962200
C 2.60987500 1.50150900 -0.00288300
H 3.36622600 0.80384200 0.33541000
C -4.10392800 -0.23124900 -1.03469200
H -4.70443700 -0.43206700 -1.91483500
C 2.95961800 2.82357300 -0.24080700
H 3.98211600 3.14610100 -0.08897800
C -3.88016700 0.69103500 1.16107200
H -4.30555700 1.21684600 2.00848700
C -4.65797000 0.45681900 0.03582300
H -5.68490100 0.79725100 -0.00216800
C 1.99753700 3.73196600 -0.67244500
H 2.27043500 4.76319000 -0.85832200
C 0.68675900 3.31245700 -0.86179400
H -0.06648500 4.01460200 -1.19609400
C 1.67877900 -1.13648800 2.74559200
H 2.45520000 -1.72286000 3.24019700
H 1.81309200 -0.09094700 3.03616000
H 0.70987400 -1.47326800 3.12014500
C 1.67651100 -1.67809400 -2.40381000
H 1.81597900 -0.69035300 -2.85314700
H 2.47381800 -2.32590800 -2.77250200
H 0.72425300 -2.08178100 -2.75262300
C -2.21286600 -1.43243900 -2.17137700
H -1.42318400 -0.86242500 -2.67007800
H -1.76410700 -2.37501500 -1.84915800
H -2.98434400 -1.64888100 -2.91090800

C -1.75482500 0.50755600 2.48144700
H -1.41514900 -0.43195700 2.92445500
H -0.86190800 1.10593100 2.28286500
H -2.35139700 1.03617300 3.22533800
Si 2.79521500 -2.29251300 0.26179900

(Tip'Si)$_2$ **4'**$_2$
82
-2833.919777
P -3.47001700 0.11139700 0.06903600
P 3.47001100 -0.11137000 -0.06899300
Si -1.01290000 0.85192600 0.13576000
Si 1.01288100 -0.85186100 -0.13565900
N 2.26408400 -0.30208100 -1.23785400
N -2.28398400 0.61984100 -1.04031700
N -4.73199000 0.95346400 0.23372500
N 4.73196400 -0.95346300 -0.23369100
N -2.26412400 0.30212800 1.23792600
N 2.28399500 -0.61979100 1.04038700
C 3.76339000 1.65812800 0.23611100
C 6.11479400 -0.96328900 -0.20732900
C -2.87130300 -2.64063300 0.20571000
H -1.98470300 -2.35351900 0.75452700
C 6.87816700 -0.38123500 -1.24515500
C -6.77748200 1.63591000 -0.84505900
C -3.76335700 -1.65810400 -0.23608400
C -4.90477800 -2.04726300 -0.94507600
H -5.61535900 -1.30195400 -1.27715500
C 6.77748800 -1.63595300 0.84499200
C 4.90486100 2.04727400 0.94502900
H 5.61545600 1.30195700 1.27705900
C -6.11482000 0.96325200 0.20728700
C 2.87131500 2.64066700 -0.20562000
H 1.98467500 2.35356400 -0.75437900
C -5.14432500 -3.39040100 -1.20957500
H -6.03268100 -3.67896800 -1.75701800
C -3.11708600 -3.98289400 -0.05513500
H -2.42135400 -4.73495500 0.29538500
C 8.16840500 -1.68896200 0.85633400
H 8.66564000 -2.20748200 1.66844200
C 3.11712600 3.98292500 0.05521600
H 2.42137800 4.73499400 -0.29525500
C 8.26798300 -0.45817700 -1.19227000
H 8.84561000 -0.00767000 -1.99156900
C -4.25249800 -4.35967300 -0.76453900
H -4.44337200 -5.40622600 -0.96637000
C 8.92134200 -1.10064600 -0.15014700
H 10.00249600 -1.15205900 -0.12905100
C -6.87822800 0.38116400 1.24506900
C 4.25258900 4.35969000 0.76454600
H 4.44348500 5.40624100 0.96637000
C 5.14443700 3.39040800 1.20951900
H 6.03283100 3.67896500 1.75690500
C -8.16840000 1.68887900 -0.85646800
H -8.66560900 2.20739500 -1.66859400
C -8.92136900 1.10052900 0.14996900
H -10.00252300 1.15191200 0.12882000
C -8.26804400 0.45806600 1.19211600
H -8.84569800 0.00753300 1.99138200
C -2.42941100 0.34370400 2.67799800
H -2.63567800 -0.64675300 3.09276700
H -1.51242100 0.71602800 3.13798900
H -3.24454700 1.01172400 2.96394800
C 2.42932500 -0.34363400 -2.67793100
H 1.51230900 -0.71591900 -3.13790100
H 2.63561000 0.64682500 -3.09268700
H 3.24443000 -1.01167400 -2.96392200
C 2.35721100 -0.62014900 2.48648000
H 2.46182200 0.39151500 2.89178400
H 1.44417400 -1.05286000 2.89897800
H 3.19732900 -1.22106500 2.83921300
C 5.98369300 -2.29073200 1.94013500
H 5.49589900 -1.55045000 2.58223800
H 5.19088900 -2.91984100 1.53011700
H 6.62688100 -2.90308600 2.57289300
C 6.21032400 0.29336400 -2.41141400
H 5.55114800 -0.39749500 -2.94277800
H 5.59862900 1.14398200 -2.10161600
H 6.95416600 0.66030600 -3.11906400

C -6.21042200 -0.29343000 2.41135200
H -5.59869900 -1.14403600 2.10157300
H -5.55127800 0.39743800 2.94274600
H -6.95428600 -0.66038900 3.11897000
C -5.98365100 2.29072700 -1.94015300
H -5.19087400 2.91983900 -1.53008700
H -5.49581900 1.55046700 -2.58225400
H -6.62682300 2.90308500 -2.57292300
C -2.35715400 0.62017800 -2.48641200
H -3.19728100 1.22106100 -2.83917800
H -1.44411900 1.05291600 -2.89888500
H -2.46171800 -0.39149500 -2.89170700

M06-D3/def2-TZVP//M06-L-D3/def2-TZVP

TipGe **5**
103
-4216.483627
Ge -0.23102700 -1.96249200 -1.97474800
P -0.01244600 -0.29653600 0.12737000
N -1.28193100 -0.90001200 -0.82021600
C 0.03220400 -1.22787300 1.68791300
N 1.05813400 -1.09141600 -0.91400200
C 0.03711900 -2.62250000 1.71298100
N 0.12747100 1.20978200 0.29481200
C 0.09742100 -3.31545900 2.90819200
C 0.15852300 -2.61910500 4.10824400
C 0.15791300 -1.23364200 4.10162700
C 0.09462800 -0.54099400 2.90093100
C -2.67067900 -0.59670000 -0.81295300
C -3.11834000 0.51817700 -1.55761400
C -4.47338000 0.82231900 -1.54718500
C -5.38112700 0.04990600 -0.84146100
C -4.93727200 -1.05780600 -0.14971700
C -3.58858100 -1.40637500 -0.12496200
C -2.15664300 1.34164800 -2.39099500
C -1.91420500 0.67592000 -3.74304900
C -2.60186700 2.78026500 -2.61215200
C -3.17785800 -2.64617500 0.63293100
C -3.89246200 -3.88607300 0.10638800
C -3.39419300 -2.48446800 2.13343600
C 2.41637000 -0.75630400 -1.14129100
C 2.71879400 0.30450000 -2.02044100
C 4.04887500 0.67989700 -2.17150100
C 5.06334400 0.03326100 -1.48952500
C 4.75795900 -1.03851800 -0.67268000
C 3.44475000 -1.46337100 -0.49506100
C 1.66629800 1.03016800 -2.83085100
C 1.86603000 0.76231300 -4.32033100
C 1.62901100 2.52477200 -2.53943400
C 3.17761700 -2.69279800 0.34260100
C 0.24097500 2.28635000 1.15818800
C -0.92187400 2.97136600 1.58692600
C -0.79478400 4.04153200 2.46192400
C 0.44294000 4.46898100 2.91115800
C 1.57706200 3.82466600 2.45614200
C 1.50871700 2.74832600 1.57768800
C -2.28172300 2.52511700 1.11069500
C -3.30421900 3.64724000 1.01837600
C -2.83382400 1.38693500 1.96288700
C 2.78821600 2.07954000 1.13423500
C 3.81816400 3.07135600 0.60723200
C 3.38290000 1.24018400 2.26053700
C 3.50930300 -2.47565800 1.81359600
C 3.92186900 -3.90420000 -0.21052700
H -0.00169700 -3.17653500 0.77961000
H 0.10022300 -4.39825300 2.90550400
H 0.20824000 -3.15863300 5.04529600
H 0.20747700 -0.68435100 5.03280700
H 0.09557600 0.54198500 2.90564400
H -4.82665400 1.68155600 -2.10246500
H -6.43213100 0.30919400 -0.84204100
H -5.64699300 -1.67307800 0.39138500
H -1.20657000 1.37843900 -1.85014600
H -1.53428300 -0.34523700 -3.65116800
H -1.19238100 1.24262100 -4.33265700
H -2.84061400 0.61353200 -4.31670100
H -1.80617600 3.34729800 -3.09417700

H -2.84652300 3.28180200 -1.67715500
H -3.47515900 2.84339500 -3.26325000
H -2.11139700 -2.79197600 0.46873100
H -3.53714500 -4.78146600 0.61745200
H -3.72731500 -4.02065100 -0.96281600
H -4.96983300 -3.82787900 0.26579900
H -4.44706400 -2.31048100 2.36228600
H -2.82678700 -1.64405900 2.53362900
H -3.08224500 -3.38105200 2.67020100
H 4.28976200 1.50399600 -2.83359200
H 6.09046300 0.35420700 -1.60812200
H 5.55456800 -1.56779700 -0.16207000
H 0.68592300 0.63720000 -2.55104400
H 1.10421300 1.26756900 -4.91461500
H 1.81799600 -0.30346100 -4.54865400
H 2.83775300 1.12356000 -4.65959500
H 2.56980500 3.01027200 -2.80424200
H 1.43151100 2.71499400 -1.48495600
H 0.83920200 3.00430900 -3.11968400
H 2.11119300 -2.90488200 0.27232100
H -1.68829900 4.55558900 2.79465500
H 0.52094000 5.30295100 3.59648800
H 2.55222300 4.16151300 2.78981300
H -2.13471600 2.12584000 0.10552500
H -4.20237600 3.29371500 0.50934900
H -2.91855700 4.50673800 0.46946800
H -3.61566200 3.99921200 2.00309100
H -2.16493700 0.52650500 1.96650900
H -3.80153300 1.04776000 1.58811200
H -2.96323000 1.70560000 2.99967500
H 2.53695300 1.40411100 0.31624000
H 4.15714100 3.75655400 1.38565900
H 3.41584500 3.67376200 -0.20706800
H 4.69680600 2.54525700 0.23267400
H 4.26244800 0.69068300 1.92053800
H 2.66211900 0.51558300 2.64104000
H 3.68378600 1.87016100 3.10022500
H 4.55835000 -2.20481300 1.94682900
H 3.32704000 -3.38347700 2.38985700
H 2.90545300 -1.67971100 2.24835000
H 3.68132400 -4.07881300 -1.25929100
H 3.66291800 -4.80355500 0.34903300
H 5.00302800 -3.77832200 -0.13964400

$(TipGe)_2$ **5**$_2$
206
-8432.980673
Ge -1.16731700 0.20928800 0.83296700
P -3.57056700 0.00794300 -0.35113000
N -2.55487700 1.27958800 0.13927500
C -3.54461600 -0.11681700 -2.15913300
N -2.39812900 -1.07022300 0.21789400
C -2.34041700 -0.18939000 -2.86209000
H -1.40286700 -0.14577200 -2.31968300
N -4.96347100 -0.07050000 0.26372100
C -2.32655000 -0.32504600 -4.23800600
H -1.38145600 -0.38156900 -4.76519600
C -3.52428000 -0.39390100 -4.93805900
H -3.51717100 -0.50323700 -6.01497200
C -4.72767100 -0.32431000 -4.25435100
H -5.66410700 -0.37915900 -4.79431400
C -4.74083100 -0.18642700 -2.87347900
H -5.68629900 -0.13589700 -2.34816100
C -2.79962000 2.67693300 0.23990600
C -3.36664100 3.18701200 1.43111800
C -3.59156300 4.55437600 1.52730200
H -4.03192200 4.95465200 2.43140800
C -3.26829500 5.41399000 0.49124200
H -3.46124400 6.47508100 0.58413900
C -2.68892400 4.90904300 -0.65410600
H -2.42152900 5.58181700 -1.46006400
C -2.43277800 3.54734300 -0.80109600
C -3.68335500 2.28841700 2.61010000
H -3.97711300 1.31522800 2.20694000
C -2.45254500 2.10213900 3.49523900
H -1.58417400 1.71529000 2.95323600
H -2.66310500 1.41356300 4.31465900
H -2.14645500 3.05536200 3.93048400
C -4.83640300 2.78013500 3.47543900

H	-4.57237200	3.67903300	4.03447300
H	-5.10223800	2.01916400	4.20854100
H	-5.72646600	3.00179900	2.89016500
C	-1.75844600	3.07742900	-2.06940500
H	-1.49321100	2.02680700	-1.93235800
C	-0.46704500	3.84364500	-2.33400700
H	-0.65098300	4.90593300	-2.49896400
H	0.03158800	3.46519300	-3.22620800
H	0.23193000	3.76022400	-1.50129100
C	-2.69826500	3.17296700	-3.26670600
H	-3.59972300	2.57708000	-3.12660800
H	-2.20571200	2.82321900	-4.17474900
H	-3.00831100	4.20620500	-3.43385900
C	-2.60326100	-2.39789300	0.66677000
C	-3.06798900	-2.61781800	1.97944100
C	-3.30124800	-3.92627400	2.38840100
H	-3.66271800	-4.10621900	3.39464400
C	-3.08894200	-4.99599100	1.53852100
H	-3.29468300	-6.00517500	1.87179400
C	-2.59585100	-4.76910700	0.26722000
H	-2.39893800	-5.60892100	-0.38889800
C	-2.32435300	-3.48125900	-0.18484900
C	-3.26174400	-1.50003300	2.98209300
H	-3.11395700	-0.54738200	2.46773200
C	-2.21243500	-1.59343100	4.08748500
H	-2.26415200	-2.55263700	4.60494500
H	-2.35186500	-0.80904800	4.83181300
H	-1.20032000	-1.49687200	3.68510700
C	-4.67360100	-1.44849500	3.54841100
H	-4.93532600	-2.36777400	4.07467100
H	-5.40373200	-1.28806000	2.75553400
H	-4.76681700	-0.62663800	4.25972500
C	-6.32255300	-0.18701100	0.02498500
C	-7.13081600	0.97653200	0.02510700
C	-8.49873500	0.85509300	-0.17254700
H	-9.11091700	1.74857000	-0.17392500
C	-9.09648600	-0.37917300	-0.36469400
H	-10.16565900	-0.45499100	-0.51405700
C	-8.30551100	-1.51090400	-0.36029700
H	-8.76357600	-2.48251400	-0.51146200
C	-6.92833200	-1.44811000	-0.16993100
C	-6.49097200	2.33169200	0.19352200
H	-5.64368700	2.18742300	0.86693000
C	-7.40217500	3.38306600	0.80596300
H	-8.19754500	3.68329500	0.12204500
H	-6.83085200	4.28264000	1.04103200
H	-7.87376600	3.03106900	1.72393300
C	-5.92341200	2.83940500	-1.12878400
H	-5.20441900	2.13743900	-1.55160900
H	-5.41163600	3.79502500	-0.99948200
H	-6.71880800	2.97604800	-1.86475200
C	-6.13848600	-2.73627100	-0.20666800
H	-5.08581300	-2.49040300	-0.05591000
C	-6.55546200	-3.68745600	0.91073200
H	-6.41422500	-3.24154600	1.89466800
H	-5.97111700	-4.60727000	0.87487200
H	-7.60848300	-3.96029000	0.82115100
C	-6.26684300	-3.43015400	-1.55952300
H	-7.29477700	-3.74578900	-1.74587700
H	-5.63755700	-4.32058200	-1.60024900
H	-5.97419900	-2.77445700	-2.38059900
C	-2.71018100	-3.59431700	-2.66329600
H	-3.04230200	-4.63334700	-2.61874800
H	-2.26344500	-3.43062400	-3.64461200
H	-3.59473000	-2.96164400	-2.58994000
C	-0.44095600	-4.11942200	-1.71714200
H	0.30404300	-3.87206700	-0.95893900
H	0.00883600	-3.94346600	-2.69451100
H	-0.63739200	-5.18980400	-1.64179300
C	-1.71114300	-3.29217100	-1.55354700
H	-1.42812600	-2.24017700	-1.63661700
Ge	1.16533100	-0.19088500	-0.82844000
P	3.57222700	-0.01047100	0.34962900
N	2.54538900	-1.27441900	-0.13920000
C	3.55552600	0.10368700	2.15824600
N	2.40647700	1.07702400	-0.21391700
C	2.35625900	0.17827500	2.86939800
H	1.41466500	0.14170500	2.33321000
N	4.96263600	0.06397800	-0.27014000

C	2.35239100	0.30603200	4.24613900
H	1.41154900	0.36564900	4.78034400
C	3.55498100	0.36299500	4.93890200
H	3.55529700	0.46614700	6.01644500
C	4.75346100	0.28849300	4.24731000
H	5.69372500	0.33285500	4.78157600
C	4.75666400	0.15951800	2.86552700
H	5.69832100	0.10523000	2.33376100
C	2.78629100	-2.67222600	-0.24637800
C	3.33648200	-3.18059700	-1.44631800
C	3.56567700	-4.54680800	-1.54674300
H	3.99392000	-4.94471900	-2.45772200
C	3.26057100	-5.40816300	-0.50660200
H	3.45658100	-6.46842300	-0.60246500
C	2.69377000	-4.90591900	0.64613600
H	2.43860400	-5.58020400	1.45481700
C	2.43493100	-3.54513900	0.79781600
C	3.62233100	-2.28092200	-2.63162700
H	3.91566700	-1.30519900	-2.23456500
C	2.36845500	-2.10909900	-3.48774000
H	1.50339800	-1.75411000	-2.91949800
H	2.54199900	-1.40401800	-4.30159700
H	2.07521400	-3.06325200	-3.92987500
C	4.76095200	-2.76125200	-3.52154100
H	4.49681300	-3.66733400	-4.06871000
H	4.99760600	-2.00110700	-4.26543300
H	5.66799400	-2.96596300	-2.95636500
C	1.77295300	-3.08015600	2.07430600
H	1.50800400	-2.02870300	1.94451500
C	0.48239800	-3.84553000	2.34605800
H	0.66486900	-4.91022900	2.49647900
H	-0.00406500	-3.47562400	3.24852200
H	-0.22651700	-3.75014000	1.52300600
C	2.72334200	-3.18346600	3.26247800
H	3.62636100	-2.59158000	3.11523900
H	2.24112800	-2.83388600	4.17610100
H	3.03005500	-4.21856800	3.42404800
C	2.61054600	2.40978800	-0.64856500
C	3.08028800	2.64559700	-1.95663300
C	3.30261900	3.96019000	-2.35255400
H	3.66741700	4.15237800	-3.35533900
C	3.07528700	5.02050500	-1.49500900
H	3.27162400	6.03454100	-1.81907500
C	2.58050100	4.77754600	-0.22733900
H	2.37329300	5.60935100	0.43581400
C	2.32054600	3.48317800	0.21220100
C	3.29634900	1.53914500	-2.96762600
H	3.14453300	0.58096000	-2.46524100
C	2.26837000	1.63645700	-4.09251200
H	2.31857200	2.60326500	-4.59575500
H	2.43450600	0.86497800	-4.84490500
H	1.24924500	1.52034800	-3.71399000
C	4.71900200	1.50501000	-3.50861600
H	4.98363000	2.43499300	-4.01433900
H	5.43583200	1.33633700	-2.70562500
H	4.83304100	0.69700800	-4.23255900
C	6.32331000	0.17726200	-0.04228000
C	7.13083600	-0.98611100	-0.07784100
C	8.50147300	-0.86941900	0.10199900
H	9.11333100	-1.76272300	0.07510500
C	9.10211500	0.36057600	0.31241500
H	10.17346100	0.43337000	0.44698400
C	8.31122300	1.49186800	0.34733100
H	8.77164800	2.45959900	0.51527200
C	6.93126000	1.43372000	0.17594100
C	6.48477600	-2.33637900	-0.25990800
H	5.62990700	-2.17972500	-0.92129300
C	7.38487800	-3.38216700	-0.89736300
H	8.18861800	-3.69268500	-0.22797000
H	6.80814600	-4.27737300	-1.13587000
H	7.84491800	-3.01996600	-1.81724800
C	5.93103600	-2.85939600	1.06254600
H	5.22193700	-2.15889300	1.50454900
H	5.41167700	-3.80996500	0.92679700
H	6.73531000	-3.01111900	1.78576400
C	6.14257100	2.72081400	0.25745300
H	5.08717900	2.47936500	0.11732700
C	6.54231600	3.70089700	-0.84119900
H	6.39413700	3.27831900	-1.83417300

H 5.95275100 4.61587000 -0.77708900
H 7.59453200 3.97801000 -0.75515800
C 6.29286000 3.38204900 1.62451000
H 7.32573700 3.68428100 1.80548200
H 5.67297900 4.27732800 1.69269600
H 6.00332600 2.71130900 2.43428400
C 2.70659000 3.58057900 2.69004900
H 3.02940700 4.62276700 2.65151300
H 2.26231600 3.40683000 3.67073000
H 3.59678000 2.95609700 2.61224200
C 0.42869500 4.08449300 1.75027700
H -0.31236000 3.83514200 0.98866900
H -0.01946300 3.89168400 2.72531100
H 0.61027300 5.15826000 1.68620600
C 1.70965500 3.27578300 1.57900500
H 1.43980000 2.22010400 1.65349000

Adiabatic gap

TipGe **5** (triplet)
103
Ge 0.25089700 -1.66391300 -2.10374100
P 0.07147800 -0.30960600 0.11806400
N -1.07141700 -1.03051500 -0.88828800
C 0.27496400 -1.35880600 1.56994000
N 1.37530200 -0.82412900 -0.89765400
C 0.61389800 -2.75795700 1.51232800
N -0.16750000 1.21881200 0.36164600
C 0.74246400 -3.55384800 2.62515200
C 0.52203500 -2.95225100 3.81730900
C 0.18982100 -1.56407700 3.89070800
C 0.07282900 -0.76190800 2.77632900
C -2.57920300 -0.93321500 -0.90143900
C -3.29222500 0.16722900 -1.57891000
C -4.75393600 0.25302500 -1.57054300
C -5.51155800 -0.71299600 -0.93196400
C -4.81196500 -1.79603000 -0.31108400
C -3.34538200 -1.94545200 -0.29257400
C -2.49789900 1.21131800 -2.33643900
C -2.17321900 0.70153300 -3.69882900
C -3.28045200 2.62335600 -2.44451200
C -2.65658100 -3.19497300 0.38787500
C -3.19515400 -4.51762600 -0.13635200
C -2.89891500 -3.13854700 1.83894700
C 2.71060900 -0.26496300 -1.11370800
C 2.79168400 0.84150500 -1.97843800
C 4.11993500 1.31505900 -2.23757500
C 5.31937100 0.73049100 -1.65808600
C 5.22346000 -0.34551400 -0.80222700
C 3.92609400 -0.87194600 -0.52308200
C 1.52376100 1.52106300 -2.67250700
C 1.67874500 1.40675800 -4.13472400
C 1.24164300 3.00616100 -2.26400300
C 3.86288100 -2.11746400 0.34054900
C -0.28154300 2.19172000 1.33499400
C -1.63602900 2.68759500 1.73998500
C -1.74412600 3.67987300 2.69171800
C -0.56753000 4.18802400 3.24202100
C 0.74589500 3.72407600 2.82854300
C 0.92181000 2.73457700 1.87335300
C -2.94161200 2.10700600 1.18583900
C -4.22179000 3.12950500 1.11094900
C -3.34560700 0.85303400 1.94995600
C 2.40083400 2.27399000 1.47148000
C 3.20558700 3.47784200 0.95152400
C 3.22145300 1.57097800 2.57655700
C 4.60944600 -1.97871600 1.62078900
C 4.49300100 -3.36640400 -0.26820100
H 0.75612500 -3.15135700 0.65937200
H 0.97278500 -4.47330200 2.56136300
H 0.58912100 -3.46026000 4.61727900
H 0.04312600 -1.17714400 4.74583900
H -0.13837400 0.16164100 2.84661900
H -5.18575400 0.97790400 -2.00711300
H -6.45968900 -0.65926000 -0.90674600
H -5.32930200 -2.46628500 0.11976200
H -1.63932500 1.36628800 -1.84776900
H -1.67713700 -0.14022400 -3.62300100
H -1.62571000 1.36183400 -4.17279500

H -3.00333400 0.54779600 -4.19651300
H -2.68074400 3.30125800 -2.82026400
H -3.57251100 2.90412300 -1.55217200
H -4.06207500 2.51995900 -3.02655700
H -1.67150700 -3.15514500 0.22040100
H -2.72030800 -5.25800200 0.29583600
H -3.05969800 -4.56429500 -1.10582300
H -4.15241700 -4.58268700 0.06324400
H -3.86359800 -3.10277600 2.00790200
H -2.47229700 -2.33834100 2.21052100
H -2.52218500 -3.93699400 2.26435000
H 4.22304200 2.05575100 -2.82346200
H 6.17195600 1.09178100 -1.87035800
H 5.99962700 -0.72657700 -0.40870400
H 0.72032200 0.98396500 -2.41551700
H 0.90168300 1.80702800 -4.57785900
H 1.74536600 0.46115000 -4.38323700
H 2.49218700 1.87618900 -4.41467500
H 2.00659800 3.56494600 -2.51500200
H 1.10327700 3.05783000 -1.29519400
H 0.43848200 3.32515000 -2.72615400
H 2.90463800 -2.31278300 0.54938500
H -2.59342600 4.00704200 2.96396200
H -0.62692900 4.86065600 3.91023700
H 1.51433800 4.11407300 3.22841900
H -2.75046700 1.81994800 0.24719900
H -4.95728200 2.69730200 0.62863100
H -3.95898300 3.94804000 0.64048400
H -4.51400800 3.35413800 2.01900100
H -2.60569800 0.21055600 1.93785000
H -4.13331300 0.45215000 1.52665300
H -3.55773900 1.08955000 2.87701600
H 2.31294700 1.62809100 0.71313400
H 3.34826900 4.11544700 1.68195800
H 2.70898900 3.91506600 0.22860500
H 4.07208500 3.16983000 0.61284100
H 4.09855100 1.31719000 2.22066400
H 2.74571200 0.76824700 2.87604300
H 3.34039100 2.18176000 3.33366700
H 5.57004900 -1.92437200 1.43453000
H 4.43041600 -2.75660500 2.18930700
H 4.31939700 -1.16378600 2.08141200
H 4.05763500 -3.56867500 -1.12256000
H 4.37723900 -4.12247500 0.34447900
H 5.44891000 -3.21036600 -0.41755200

B3LYP/def2-TZVP

Tip’Ge **5’**

41
-3204.499821
P 0.61725800 -0.32447600 0.07212700
N 1.73527000 -0.72968000 1.26238300
N -0.78745200 -0.90013100 0.22416500
N 1.67452200 -1.01367700 -1.06052600
C 0.67061900 1.47882500 -0.21458300
C -2.15127400 -0.69179300 0.18456600
C -2.82332200 -0.00840100 1.22487500
C -2.90409200 -1.23464100 -0.88279400
C -0.41757400 2.10212500 -0.83304200
H -1.28871500 1.52117100 -1.10524400
C 1.77485200 2.25599900 0.14775700
H 2.62230500 1.79131500 0.63606300
C -4.28528400 -1.06356000 -0.90535600
H -4.85095100 -1.48437000 -1.72921100
C 1.79156700 3.62224100 -0.10082300
H 2.65105500 4.21248200 0.19197700
C -4.20674700 0.13948800 1.16031200
H -4.71235400 0.66657400 1.96170600
C -4.94465200 -0.37581000 0.10384600
H -6.01982000 -0.25272900 0.07353700
C 0.70578000 4.23136900 -0.72206600
H 0.71884900 5.29657200 -0.91605800
C -0.39685700 3.46900000 -1.08804900
H -1.24682500 3.93734100 -1.56815900
C 1.53421400 -0.70673000 2.69699300
H 2.40086100 -1.14819000 3.19275200

H 1.41747500 0.31228000 3.07752000
H 0.65050600 -1.28102200 2.98637900
C 1.57906500 -0.95314000 -2.50480000
H 1.49075100 0.07598400 -2.86836600
H 2.47768200 -1.38411500 -2.95016300
H 0.71982400 -1.51906000 -2.87195900
C -2.21470100 -1.99274500 -1.98167100
H -1.60457300 -1.33199700 -2.60525000
H -1.54001500 -2.74914500 -1.57500800
H -2.94141400 -2.48223400 -2.63091600
C -2.06621600 0.53580300 2.40444000
H -1.53432400 -0.25899500 2.93307500
H -1.31917800 1.27615100 2.10859600
H -2.74685000 1.01347500 3.10977200
Ge 3.03650600 -1.49025400 0.13585300

(Tip'Ge)$_2$ **5'$_2$**
82
-6408.988317
P 3.93741800 0.18130800 -0.04244900
P -3.93742300 -0.18133700 0.04241800
N -2.76068500 -0.47652700 1.21036100
N 2.83264600 0.74911400 1.11489600
N 5.26693700 0.90860500 -0.21717400
N -5.26696100 -0.90858700 0.21719800
N 2.76072100 0.47645400 -1.21044500
N -2.83272300 -0.74918100 -1.11497700
C -4.08454800 1.61702800 -0.23117900
C -6.64646100 -0.84988000 0.19951200
C 3.06361600 -2.50636000 -0.11809200
H 2.16088000 -2.13258000 -0.58389900
C -7.37174200 -0.24639900 1.25319300
C 7.35232300 1.46945800 0.85799300
C 4.08459400 -1.61705100 0.23115700
C 5.24545400 -2.12217900 0.82499000
H 6.05342800 -1.45187500 1.08639800
C -7.35242300 -1.46934600 -0.85786800
C -5.24540700 2.12219500 -0.82498100
H -6.05340900 1.45191800 -1.08637000
C 6.64643800 0.84996100 -0.19942000
C -3.06353300 2.50630400 0.11804800
H -2.16079600 2.13249300 0.58382900
C 5.37794600 -3.48487100 1.06777300
H 6.28272300 -3.86166600 1.52781100
C 3.20061600 -3.86805600 0.11854700
H 2.40483700 -4.54633000 -0.16327300
C -8.74425800 -1.45000900 -0.85782300
H -9.27392200 -1.92831800 -1.67416100
C -3.20049500 3.86800500 -0.11858300
H -2.40468800 4.54625300 0.16322000
C -8.76390400 -0.25001600 1.21100900
H -9.31097700 0.21684800 2.02239600
C 4.35779500 -4.35982700 0.71425500
H 4.46429500 -5.42153200 0.89831100
C -9.45813100 -0.84043300 0.16434800
H -10.54063100 -0.83527500 0.15157900
C 7.37179600 0.24651200 -1.25306800
C -4.35767400 4.35981500 -0.71425900
H -4.46414500 5.42152400 -0.89830900
C -5.37786200 3.48489200 -1.06775500
H -6.28263800 3.86171700 -1.52776800
C 8.74415900 1.45018300 0.85801500
H 9.27376300 1.92851500 1.67437700
C 9.45810800 0.84063900 -0.16412300
H 10.54060700 0.83552900 -0.15130300
C 8.76395700 0.25019200 -1.21081700
H 9.31108900 -0.21664700 -2.02217900
C 2.94528500 0.50216700 -2.64756800
H 3.14584500 -0.49469900 -3.05098700
H 2.03776800 0.87678300 -3.12474300
H 3.77187400 1.15762600 -2.93278900
C -2.94518800 -0.50224400 2.64749200
H -2.03766400 -0.87689800 3.12462500
H -3.14569200 0.49462700 3.05092700
H -3.77179000 -1.15767500 2.93274400
C -2.95633900 -0.68381600 -2.55683000
H -3.11862900 0.34003400 -2.90973500
H -2.03955000 -1.05199200 -3.02102900

H -3.78222000 -1.30278000 -2.91437800
C -6.60284200 -2.14489100 -1.97111000
H -6.08553600 -1.41903400 -2.60620000
H -5.83810700 -2.81873400 -1.57902400
H -7.28160000 -2.71480600 -2.60642200
C -6.65993800 0.37372200 2.42355100
H -6.03859600 -0.36056500 2.94204400
H -6.00087300 1.19044400 2.11987700
H -7.37740300 0.77432800 3.14020800
C 6.66007600 -0.37364100 -2.42345900
H 6.00103600 -1.19039400 -2.11981700
H 6.03872100 0.36061800 -2.94197800
H 7.37759200 -0.77420900 -3.14008500
C 6.60265900 2.14496900 1.97120000
H 5.83791100 2.81877700 1.57907800
H 6.08535800 1.41908800 2.60626700
H 7.28136200 2.71491600 2.60654300
C 2.95619800 0.68375500 2.55675400
H 3.78204300 1.30274500 2.91433800
H 2.03937700 1.05190200 3.02091200
H 3.11850500 -0.34009000 2.90966800
Ge 1.42075600 1.11465900 -0.05795100
Ge -1.42079600 -1.11478300 0.05780500

M06-D3/def2-TZVP//M06-L-D3/def2-TZVP

TipSn **6**
103
-2354.004374
Sn -0.30811400 -2.54853000 -1.34318100
P -0.00190000 -0.15575900 0.23551500
N -1.32649600 -0.95712900 -0.43640800
C 0.09620400 -0.60884100 1.99884400
N 1.06879100 -1.19666000 -0.54769100
C 0.10610400 -1.93973700 2.41614000
H 0.03699200 -2.73499000 1.67765000
N 0.14704100 1.34469900 -0.00874300
C 0.20979300 -2.27391000 3.75375600
H 0.21481400 -3.31455600 4.05325400
C 0.31266400 -1.27067400 4.70908500
H 0.39746700 -1.52728400 5.75721500
C 0.30862300 0.05683100 4.31420000
H 0.39076900 0.84456200 5.05206500
C 0.20040000 0.38722300 2.97004300
H 0.19974500 1.42852100 2.67343500
C -2.70060500 -0.60707400 -0.45314500
C -3.60466200 -1.14836700 0.47768200
C -4.94743900 -0.78534800 0.40047000
H -5.64200900 -1.19029400 1.12770400
C -5.40522500 0.07826700 -0.57296300
H -6.45082800 0.35584700 -0.61087000
C -4.51697000 0.57785300 -1.51123000
H -4.88092500 1.24183300 -2.28480700
C -3.16916200 0.24494300 -1.48113700
C -3.18150000 -2.11590900 1.55771600
H -2.13129200 -2.35355800 1.39102900
C -3.96343700 -3.42287900 1.48144700
H -3.59530500 -4.13575400 2.22014400
H -3.88144500 -3.88498800 0.49704700
H -5.02475700 -3.27011500 1.68073000
C -3.29386400 -1.49298100 2.94481200
H -2.66817200 -0.60544600 3.03798300
H -2.98421800 -2.19965400 3.71558800
H -4.32225000 -1.19662400 3.15917000
C -2.23332500 0.75130800 -2.56092900
H -1.26088900 0.92432600 -2.09123300
C -2.66746800 2.06488400 -3.19570600
H -3.56838500 1.94986200 -3.80051500
H -1.88669900 2.43696200 -3.85830400
H -2.86326300 2.83324500 -2.44982300
C -2.05874000 -0.29610000 -3.65672100
H -1.70626300 -1.25836000 -3.27189800
H -1.34176400 0.03689500 -4.40830200
H -3.00613900 -0.49625700 -4.16023700
C 2.40778600 -0.90664000 -0.89699100
C 3.47407300 -1.39753800 -0.12099500
C 4.77353600 -1.04185900 -0.46853500
H 5.59688400 -1.40206400 0.13794900
C 5.03307700 -0.24965600 -1.57095900

H 6.05056100 0.02274900 -1.82089500
C 3.98274100 0.18044200 -2.36061000
H 4.18634000 0.78249400 -3.23926700
C 2.66489000 -0.13553100 -2.05101400
C 3.25166600 -2.30959800 1.06351400
H 2.19405100 -2.57530300 1.07026600
C 4.05088100 -3.60176700 0.93801300
H 3.83622000 -4.11966700 0.00292400
H 3.81765400 -4.27946400 1.75989100
H 5.12521200 -3.41617300 0.96847100
C 3.55444300 -1.60787000 2.38162800
H 3.37168400 -2.27089900 3.22815100
H 2.93426500 -0.72151700 2.51290000
H 4.59799200 -1.29048600 2.42756700
C 1.57155900 0.30926000 -2.99913000
H 0.61025100 0.00745500 -2.57569900
C 1.72699200 -0.38934500 -4.34795400
H 2.67819800 -0.13566900 -4.81768200
H 0.93414600 -0.09642900 -5.03708800
H 1.69941700 -1.47610000 -4.24646700
C 1.50944600 1.82126900 -3.16580500
H 1.32358200 2.31231100 -2.21121000
H 0.70202200 2.09556500 -3.84660500
H 2.43609400 2.21960500 -3.58274400
C 0.30828500 2.60783100 0.53451800
C 1.58662900 3.11223000 0.86720200
C 1.69443600 4.40290500 1.37679300
H 2.68008600 4.77636800 1.63318800
C 0.59224400 5.21206500 1.56526400
H 0.70295500 6.21254500 1.96280100
C -0.65802500 4.72003600 1.23190900
H -1.53066600 5.34610700 1.37224300
C -0.82405700 3.44176700 0.71866700
C 2.85185700 2.30008700 0.71876000
H 2.58141100 1.32836600 0.30398700
C 3.52244300 2.06195700 2.06839800
H 3.86784500 2.99915600 2.50860000
H 4.38976600 1.40877800 1.95956400
H 2.84087200 1.59897400 2.78318400
C 3.83483100 2.96069700 -0.24288900
H 3.40839600 3.07607200 -1.23891100
H 4.74446000 2.36723800 -0.33599400
H 4.12176000 3.95308000 0.10941100
C -2.20148400 2.91826400 0.40312700
H -2.08321600 2.23934900 -0.44253700
C -2.75087300 2.09364700 1.56303100
H -2.85346800 2.70909200 2.45964300
H -2.08975900 1.26251400 1.80957100
H -3.73012300 1.67513400 1.32318900
C -3.20473600 3.98901000 0.00519200
H -3.48519400 4.62132600 0.84907500
H -4.12213400 3.52435200 -0.36019500
H -2.81808000 4.64059300 -0.77903400

TipPb **7**
103
-2332.533021
Pb -0.40250700 -2.65663600 -0.69479500
P 0.03046600 0.11517800 0.31566800
N -1.34443800 -0.74270800 -0.12651900
C 0.16024100 0.06072400 2.13589400
N 1.06503000 -1.08792600 -0.23169800
C 0.15047600 -1.14254100 2.84191300
H 0.04532100 -2.08084500 2.30141400
N 0.21685900 1.52646200 -0.24437600
C 0.27810800 -1.17256500 4.21832800
H 0.26746900 -2.11976000 4.74328000
C 0.42540800 0.01566400 4.92316200
H 0.52983500 -0.00254700 6.00037500
C 0.43978600 1.22059000 4.24072200
H 0.55623300 2.15111200 4.78157600
C 0.30699100 1.24451000 2.85876200
H 0.32288000 2.19268200 2.33642400
C -2.69967700 -0.34635300 -0.19163900
C -3.59472600 -0.61649000 0.86026900
C -4.92553800 -0.22621700 0.73222100
H -5.60937400 -0.42052300 1.55086600
C -5.38493600 0.40539700 -0.40515400
H -6.42035700 0.71160100 -0.48178500

C	-4.51087800	0.63180300	-1.45611000
H	-4.87685800	1.10980700	-2.35588100
C	-3.17603700	0.25711300	-1.38086300
C	-3.16913200	-1.32452600	2.12433300
H	-2.13219000	-1.63134600	1.99084900
C	-3.99238500	-2.58466000	2.36912900
H	-5.04257700	-2.35202100	2.54945800
H	-3.62539300	-3.12169100	3.24471000
H	-3.95388500	-3.26384600	1.51632300
C	-3.21837400	-0.39677600	3.33287400
H	-4.23106000	-0.02613200	3.50171200
H	-2.56810100	0.46772400	3.20024000
H	-2.89936800	-0.91640700	4.23710200
C	-2.26115600	0.44053800	-2.57497400
H	-1.26614700	0.67561000	-2.18647500
C	-2.17264600	-0.85289600	-3.38020500
H	-1.86288000	-1.70841700	-2.77135600
H	-1.46236000	-0.76184200	-4.20273900
H	-3.14410200	-1.11691900	-3.80187800
C	-2.66396200	1.57581100	-3.50494600
H	-3.59635800	1.36433500	-4.03087400
H	-1.89865500	1.72727400	-4.26559400
H	-2.78945700	2.51514900	-2.96930500
C	2.39632700	-0.93124100	-0.66842200
C	3.47113900	-1.31270800	0.15937600
C	4.77110500	-1.11988000	-0.29598000
H	5.59968400	-1.39518600	0.34675700
C	5.02532600	-0.59224300	-1.54831500
H	6.04414900	-0.44292700	-1.88234900
C	3.96568200	-0.26733100	-2.37465000
H	4.16240900	0.12749600	-3.36545500
C	2.64732600	-0.42999100	-1.96439300
C	3.24970700	-1.92653000	1.52261100
H	2.18417300	-2.14438200	1.60646500
C	4.00469400	-3.24021100	1.68776400
H	3.75746800	-3.95014700	0.89783300
H	3.76456600	-3.70407400	2.64509500
H	5.08497800	-3.09217900	1.66492800
C	3.60230500	-0.95085000	2.63877900
H	4.65665000	-0.67082800	2.59562400
H	3.41511400	-1.39185200	3.61869400
H	3.01423900	-0.03599000	2.56773800
C	1.53876700	-0.12887800	-2.95046800
H	0.58395100	-0.28549900	-2.44290500
C	1.60537400	-1.09653800	-4.12980800
H	2.55076300	-1.00473900	-4.66612600
H	0.80495400	-0.90310800	-4.84495600
H	1.52248900	-2.13647100	-3.80573300
C	1.54483900	1.31798100	-3.42384300
H	1.41656800	2.00284000	-2.58652600
H	0.72945500	1.49426300	-4.12709100
H	2.47542300	1.57088900	-3.93523400
C	0.44173100	2.86894500	-0.00399400
C	-0.64780500	3.77733300	-0.04439300
C	-0.42091400	5.13317100	0.13992600
H	-1.26198100	5.81493500	0.10778600
C	0.85121300	5.63237700	0.36216000
H	1.01130400	6.69393300	0.49903700
C	1.91018100	4.74846500	0.40765700
H	2.91128800	5.12574400	0.58721100
C	1.74022600	3.37786500	0.23316500
C	-2.04833900	3.25372000	-0.22839700
H	-1.96667400	2.38902600	-0.88893200
C	-2.61340900	2.75203800	1.09734000
H	-1.97797200	1.98105700	1.53451800
H	-3.60955600	2.32460100	0.96866400
H	-2.68371100	3.56892800	1.81908900
C	-3.01295700	4.24061900	-0.86529600
H	-3.25363000	5.06812600	-0.19592100
H	-3.95452100	3.74262000	-1.10336700
H	-2.61267600	4.66838400	-1.78510700
C	2.96400500	2.49648600	0.32823700
H	2.64713100	1.46098200	0.19230000
C	3.62804100	2.60744100	1.69767700
H	3.99793400	3.61848200	1.87634000
H	4.47989200	1.92964900	1.77109000
H	2.93627800	2.36613800	2.50571700
C	3.97611500	2.82311800	-0.76568300
H	3.54866200	2.70310800	-1.76048600

H	4.84709900	2.17119900	-0.69631400
H	4.32449100	3.85400300	-0.67906800

{tBuC$_6$H$_4$C(NDip)$_2$Si}$_2$ **8**

Si	0.73420500	0.00064300	0.98606400
Si	-0.73659300	-0.01229700	-1.01341500
N	2.11105600	-1.08797500	0.34925500
N	2.12205000	1.04943900	0.28813600
N	-2.10576900	1.08046100	-0.37017700
N	-2.12444300	-1.05612000	-0.30465000
C	2.90105100	-0.03635700	0.06534400
C	2.38037100	-2.47468400	0.26469600
C	2.57862600	2.35067300	0.61673500
C	-2.89727300	0.03263900	-0.07850700
C	-2.37544900	2.46676400	-0.29342000
C	-2.58893100	-2.35894400	-0.61343700
C	4.30419400	-0.03099000	-0.31182200
C	2.81827700	-3.15473900	1.41934300
C	2.18192600	-3.15253300	-0.94637900
C	3.40962400	2.55391100	1.73235200
C	2.12022300	3.43579300	-0.15303000
C	-4.29718100	0.03488200	0.31146400
C	-2.79920300	3.14161400	-1.45594700
C	-2.19465000	3.14832400	0.91839000
C	-3.43847800	-2.57059400	-1.71327500
C	-2.12261300	-3.43755700	0.16092600
C	5.15574200	-1.10408900	-0.02920000
C	4.86340700	1.07997400	-0.96209500
C	3.01813500	-4.52732700	1.33698100
C	3.12147800	-2.41501900	2.70608000
C	2.40258800	-4.52752900	-0.97549700
C	1.78893500	-2.42719800	-2.21174600
C	3.80848900	3.85320400	2.03239200
C	3.85217000	1.43319400	2.64488200
C	2.54363100	4.71363000	0.19546600
C	1.19224200	3.21570400	-1.33105600
C	-5.14883900	1.10599600	0.02217600
C	-4.85200000	-1.06580200	0.98227300
C	-3.00590000	4.51370700	-1.37976800
C	-3.08040400	2.40045200	-2.74720900
C	-2.42213900	4.52223100	0.94105700
C	-1.80979800	2.42898900	2.18996700
C	-3.84557500	-3.87184600	-1.99356900
C	-3.89291600	-1.45897300	-2.63157200
C	-2.55474000	-4.71759700	-0.16795500
C	-1.17845300	-3.20782800	1.32410500
H	4.77387700	-1.98349500	0.46605700
C	6.49939000	-1.05781600	-0.35805500
C	6.20251700	1.11067500	-1.28654500
H	4.24037100	1.92541300	-1.21476400
H	3.35196600	-5.06603400	2.21583700
C	2.80812900	-5.21442800	0.15322200
H	3.22242600	-1.35861100	2.45056200
C	1.97970500	-2.51650700	3.70980700
C	4.43552900	-2.85104700	3.34293400
H	2.25636300	-5.06281900	-1.90645200
H	1.54613700	-1.39574300	-1.94618000
C	0.54646400	-3.02979800	-2.85450100
C	2.95512100	-2.38050900	-3.19486400
H	4.44980900	4.01929000	2.89054700
C	3.38895000	4.92556200	1.27183900
H	3.48521200	0.49580200	2.23213000
C	3.22337400	1.56274600	4.02841100
C	5.37095300	1.32846900	2.73790500
H	2.20348800	5.56290300	-0.38010600
H	0.39766000	2.53418200	-0.99201600
C	0.51909700	4.49359600	-1.80126600
C	1.89997400	2.53467000	-2.50101600
H	-4.76977500	1.97698800	-0.49013100
C	-6.48898400	1.06796000	0.36616600
C	-6.18756800	-1.08820000	1.32192400
H	-4.22822200	-1.90937500	1.23962000
H	-3.32874800	5.04887200	-2.26500900
C	-2.81621700	5.20440900	-0.19478800
H	-3.14685200	1.33891400	-2.50135700
C	-1.94244500	2.54608400	-3.74961600
C	-4.40804200	2.80383100	-3.37810600
H	-2.28994400	5.06080100	1.87226900

H	-1.57197000	1.39419700	1.93253500
C	-0.56494400	3.02997000	2.82992100
C	-2.97743000	2.39707800	3.17188800
H	-4.50098600	-4.04485000	-2.83958700
C	-3.41700900	-4.93776400	-1.22911200
H	-3.52579300	-0.51549900	-2.23219800
C	-3.27708800	-1.60390200	-4.01945300
C	-5.41283900	-1.35937400	-2.71078800
H	-2.20863800	-5.56196200	0.41126000
H	-0.38543300	-2.53398800	0.96660400
C	-0.50412500	-4.48258100	1.80093000
C	-1.86828200	-2.51011900	2.49499500
H	7.11578500	-1.90979800	-0.10449500
C	7.06190700	0.05011600	-0.98639600
H	6.59206700	1.99071400	-1.78474700
H	2.97000200	-6.28401300	0.11042800
H	1.04863600	-2.14007000	3.28471500
H	2.19593700	-1.92673700	4.60160900
H	1.82446700	-3.55074400	4.02456200
H	4.39147500	-3.87659400	3.71118100
H	4.66750300	-2.21397600	4.19712400
H	5.27027800	-2.78583900	2.64425900
H	0.71648500	-4.05609300	-3.18443500
H	0.24949700	-2.44790700	-3.72691700
H	-0.29863000	-3.04399500	-2.16280800
H	3.83486600	-1.91220300	-2.75197000
H	2.68494500	-1.81225000	-4.08547700
H	3.24210200	-3.38344900	-3.51654600
H	3.70665500	5.92981000	1.52160800
H	3.53982700	2.47987800	4.52808200
H	3.51655400	0.72266000	4.66085400
H	2.13548900	1.56962100	3.96470100
H	5.83391600	1.26565700	1.75296200
H	5.65978100	0.43954800	3.30114100
H	5.79798500	2.19231800	3.24994200
H	0.00370600	5.00614800	-0.98938700
H	-0.21699100	4.27088700	-2.57006300
H	1.23951700	5.18754000	-2.23815900
H	2.75403000	3.12884300	-2.83327300
H	1.22341500	2.42917400	-3.34943700
H	2.25306800	1.53677900	-2.24483000
H	-7.10609500	1.91773500	0.10710300
C	-7.04720100	-0.02929800	1.01675500
H	-6.57401600	-1.96016100	1.83644700
H	-2.98372400	6.27330400	-0.15678700
H	-1.00374200	2.18081900	-3.33159000
H	-2.14602900	1.97028100	-4.65334700
H	-1.80835900	3.58922800	-4.04402100
H	-4.39335200	3.83352700	-3.73716000
H	-4.62503500	2.16840700	-4.23743700
H	-5.23893200	2.71028100	-2.67813600
H	-0.72826500	4.06206700	3.14491700
H	-0.27611900	2.45785000	3.71150400
H	0.28282700	3.02797800	2.14151400
H	-3.86035700	1.93276400	2.73121400
H	-2.71238200	1.83241400	4.06631000
H	-3.25709600	3.40402900	3.48737300
H	-3.74142800	-5.94347800	-1.46390700
H	-3.58881000	-2.53265600	-4.50021100
H	-3.58613400	-0.77845300	-4.66308700
H	-2.18857500	-1.59847800	-3.96717600
H	-5.86574600	-1.28614100	-1.72184400
H	-5.70892200	-0.47757300	-3.28117100
H	-5.84313200	-2.22994900	-3.20842700
H	-0.00049700	-5.00659900	0.98900600
H	0.24209300	-4.25328900	2.55794900
H	-1.22153400	-5.16862100	2.25492400
H	-2.71996800	-3.09723200	2.84545700
H	-1.18011800	-2.39675600	3.33298800
H	-2.22101100	-1.51390300	2.23157400
C	8.54161400	0.15373800	-1.32954200
C	-8.52307600	-0.12345000	1.37870800
C	8.70272200	0.38585200	-2.83541400
C	9.31140100	-1.10605900	-0.94856300
C	9.14254000	1.34116500	-0.56829200
C	-8.66682200	-0.33274200	2.88967500
C	-9.29377600	1.13289300	0.98827400
C	-9.13641300	-1.32011700	0.64225500
H	8.20475600	1.29852900	-3.16074100

H	8.28490600	-0.44217000	-3.40839300
H	9.75827800	0.47551000	-3.09567000
H	8.93363500	-1.98644400	-1.46935900
H	9.26520300	-1.30272300	0.12293100
H	10.36216200	-0.99314500	-1.21496300
H	10.20555900	1.44088100	-0.79254200
H	9.03654000	1.21071400	0.50932300
H	8.65778700	2.27935500	-0.83666200
H	-8.16824400	-1.24238200	3.22247300
H	-8.23908600	0.50208500	3.44520500
H	-9.71946800	-0.41454600	3.16391300
H	-8.90784300	2.01964300	1.49200500
H	-9.25918300	1.31393100	-0.08640600
H	-10.34173900	1.02677000	1.26813700
H	-10.19680700	-1.41366800	0.88108700
H	-9.04351100	-1.20552400	-0.43836700
H	-8.65078600	-2.25567200	0.91812300

5 References

[1] L. P. Spencer, R. Altwer, P. Wei, L. Gelmini, J. Gauld, D. W. Stephan, *Organometallics* **2003**, *22*, 3841–3854.

[2] M. Schlosser, J. Hartmann, *Angew. Chem. Int. Ed. Engl.* **1973**, *12*, 508−509.

[3] A. C. Filippou, O. Chernov, G. Schnakenburg, *Chem. Eur. J.* **2011**, *17*, 13574−13583.

[4] R. S. Ghadwal, H. W. Roesky, S. Merkel, J. Henn, D. Stalke, *Angew. Chem. Int. Ed.* **2009**, *48*, 5683–5686.

[5] M. C. Copsey, T. Chivers, *Acta Cryst. Sect. E, Struct. Rep.* **2006**, *E62*, m1560–m1561.

[6] J. Vrána, S. Ketkov, R. Jambor, A. Růžička, A. Lyčka, L. Dostál, *Dalton Trans.* **2016**, *45*, 10343–10354.

[7] R. Evans, Z. Deng, A. K. Rogerson, A. S. McLachlan, J. J. Richards, M. Nilsson, G. A. Morris, *Angew. Chem. Int. Ed. Engl.* **2013**, *52*, 3199–3202.

[8] R. Evans, G. Dal Poggetto, M. Nilsson, G. A. Morris, *Anal. Chem.* **2018**, *90*, 3987–3994.

[9] *CrystalClear-SM Exper*t v2.1. Rigaku Americas, *The Woodlands, Texas, USA*, and Rigaku Corporation, *Tokyo, Japan*, 2015.

[10] *CrysAlisPro* v1.171.42.49, .42.74a, 42.82a, 42.93a, and 42.96a, Rigaku Oxford Diffraction, Rigaku Corporation, *Tokyo,* Japan, 2023.

[11] N. P. Cowieson, D. Aragao, M. Clift, D. J. Ericsson, C. Gee, S. J. Harrop, N. Mudie, S. Panjikar, J. R. Price, A. Riboldi-Tunnicliffe, R. Williamson, T. Caradoc-Davies, *J. Synchrotron Radiat.* **2015**, *22*, 187–190.

[12] D. Aragao, J. Aishima, H. Cherukuvada, R. Clarken, M. Clift, N. P. Cowieson, D. J. Ericsson, C. L. Gee, S. Macedo, N. Mudie, S. Panjikar, J. R. Price, A. Riboldi-Tunnicliffe, R. Rostan, R. Williamson, T. T. Caradoc-Davies, *J. Synchrotron Radiat.* **2018**, *25*, 885–891.

[13] T. M. McPhillips, S. E. McPhillips, H.-J. Chiu, A. E. Cohen, A. M. Deacon, P. J. Ellis, E. Garman, A. Gonzalez, N. K. Sauter, R. P. Phizackerley, S. M. Soltisa, P. Kuhn, *J. Synchrotron Radiat.* **2002**, *9*, 401–406.

[14] W. Kabsch, *Acta Crystallogr., Sect. D: Biol. Crystallogr.* **2010**, *66*, 125–132.

[15] G. M. Sheldrick, SADABS, Version 2008/1. Bruker AXS Inc., Germany.

[16] G. M. Sheldrick, *Acta Crystallogr., Sect. A: Found. Adv*. **2015**, *71*, 3−8.

[17] M. C. Burla, R. Caliandro, M. Camalli, B. Carrozzini, G. L. Cascarano, C. Giacovazzo, M. Mallamo, A. Mazzone, G. Polidori, R. Spagna, *J. Appl. Crystallogr.* **2012**, *45*, 357−361.

[18] G. M. Sheldrick, *Acta Crystallogr., Sect. C: Struct. Chem.* **2015**, *71*, 3−8.

[19] A. L. Spek, *Acta Crystallogr. Sect C: Struct. Chem.* **2015**, *71*, 9−18.

[20] A. L. Spek, *Acta Crystallogr. Sect D: Biol. Crystallogr.* **2009**, *65*, 148−155.

[21] *CrystalStructure* v4.3.0. Rigaku Americas, *The Woodlands, Texas, USA*, and Rigaku Corporation, *Tokyo, Japan*, 2018.

[22] O. V. Dolomanov, L. J. Bourhis, R. J. Gildea, J. A. K. Howard, H. Puschmann, *J. Appl. Crystallogr*. **2009**, *42*, 339–341.

[23] D. Kratzert, J. J. Holstein, I. Krossing, *J. Appl. Cryst*. **2015**, *48*, 933–938.

[24] D. Kratzert, I. Krossing, *J. Appl. Crystallogr.* **2018**, *51*, 928–934.

[25] J. Cui, J. Weiser, F. Fantuzzi, M. Dietz, Y. Yatsenko, A. Häfner, S. Nees, I. Krummenacher, M. Zhang, K. Hammond, P. Roth, W. Lu, R. D. Dewhurst, B. Engels, H. Braunschweig, *Chem. Commun.* **2022**, *58*, 13357–13360.

[26] F. E. Hahn, A. V. Zabula, T. Pape, A. Hepp, *Eur. J. Inorg. Chem.* **2007**, 2405–2408.

[27] F. E. Hahn, A. V. Zabula, T. Pape, F. Hupka, *Z. Anorg. Allg. Chem.* **2009**, *635*, 1341–1344.

[28] Y. Zhao, D. G. Truhlar, *J. Chem. Phys.* **2006**, *125*, 194101.

[29] F. Weigend, R. Ahlrichs, *Phys. Chem. Chem. Phys.* **2005**, *7*, 3297–3305.

[30] K. L. Schuchardt, B. T. Didier, T. Elsethagen, L. Sun, V. Gurumoorthi, J. Chase, J. Li, T. L. Windus, *J. Chem. Inf. Model.* **2007**, *47*, 1045–1052.

[31] S. Grimme, J. Antony, S. Ehrlich, H. Krieg, *J. Chem. Phys.* **2010**, *132*, 154104.

[32] Y. Zhao, D. G. Truhlar, *Theor. Chem. Acc.* **2008**, *120*, 215–241.

[33] A. D. Becke, *J. Chem. Phys.* **1993**, *98*, 5648–5652.

[34] C. Lee, W. Yang, R. G. Parr, *Phys. Rev. B* **1988**, *37*, 785–789.

[35] S. F. Boys, F. J. M. P. Bernardi, *Mol. Phys.* **1970**, *19*, 553–566.

[36] NBO 7.0: E. D. Glendening, J. K. Badenhoop, A. E. Reed, J. E. Carpenter, J. A. Bohmann, C. M. Morales, C. R. Landis, F. Weinhold, Theoretical Chemistry Institute, University of Wisconsin, Madison, 2018; https://nbo7.chem.wisc.edu/.

[37] AIMAll (Version 19.10.12), Todd A. Keith, TK Gristmill Software, Overland Park KS, USA, 2019 (aim.tkgristmill.com)

[38] Gaussian 16, Revision C.01, M. J. Frisch, G. W. Trucks, H. B. Schlegel, G. E. Scuseria, M. A. Robb, J. R. Cheeseman, G. Scalmani, V. Barone, G. A. Petersson, H. Nakatsuji, X. Li, M. Caricato, A. V. Marenich, J. Bloino, B. G. Janesko, R. Gomperts, B. Mennucci, H. P. Hratchian, J. V. Ortiz, A. F. Izmaylov, J. L. Sonnenberg, D. Williams-Young, F. Ding, F. Lipparini, F. Egidi, J. Goings, B. Peng, A. Petrone, T. Henderson, D. Ranasinghe, V. G. Zakrzewski, J. Gao, N. Rega, G. Zheng, W. Liang, M. Hada, M. Ehara, K. Toyota, R. Fukuda, J. Hasegawa, M. Ishida, T. Nakajima, Y. Honda, O. Kitao, H. Nakai, T. Vreven, K. Throssell, J. A. Montgomery, Jr., J. E. Peralta, F. Ogliaro, M. J. Bearpark, J. J. Heyd, E. N. Brothers, K. N. Kudin, V. N. Staroverov, T. A. Keith, R. Kobayashi, J. Normand, K. Raghavachari, A. P. Rendell, J. C. Burant, S. S. Iyengar, J. Tomasi, M. Cossi, J. M.

Millam, M. Klene, C. Adamo, R. Cammi, J. W. Ochterski, R. L. Martin, K. Morokuma, O. Farkas, J. B. Foresman, D. J. Fox, Gaussian, Inc., Wallingford CT, 2016.

[39] J. Contreras-García, E. R. Johnson, S. Keinan, R. Chaudret, J. P. Piquemal, D. N. Beratan, W. Yang, *J. Chem. Theory Comput.* **2011**, *7*, 625–632.

[40] C. Jones, S. J. Bonyhady, N. Holzmann, G. Frenking, A. Stasch, *Inorg. Chem.* **2011**, *50*, 12315–12325.
